# Distributed control circuits across a brain-and-cord connectome

**DOI:** 10.1038/s41586-026-10735-w

**Published:** 2026-06-08

**Authors:** Alexander S. Bates, Jasper S. Phelps, Minsu Kim, Helen H. Yang, Arie Matsliah, Zaki Ajabi, Eric Perlman, Kevin M. Delgado, Mohammed Abdal Monium Osman, Christopher K. Salmon, Jay Gager, Benjamin Silverman, Sophia Renauld, Farzaan Salman, Janki Patel, Matthew F. Collie, Jingxuan Fan, Diego A. Pacheco, Yunzhi Zhao, Wenyi Zhang, Laia Serratosa Capdevila, Ruairí J. V. Roberts, Eva J. Munnelly, Nina Griggs, Helen Langley, Borja Moya-Llamas, Zuoyu Zhang, Ryan T. Maloney, Szi-chieh Yu, Amy R. Sterling, Marissa Sorek, Krzysztof Kruk, Nikitas Serafetinidis, Serene Dhawan, Finja Klemm, Paul Brooks, Ellen Lesser, Jessica M. Jones, Sara E. Pierce-Lundgren, Su-Yee Lee, Yichen Luo, Andrew P. Cook, Theresa H. McKim, Dimitrios Stasi Giakoumas, Benjamin Gorko, Justin Ellis-Joyce, Jiayi Zhang, Emily C. Kophs, Tjalda Falt, Alexa M. Negron-Morales, Austin Burke, James Hebditch, Kyle P. Willie, Ryan Willie, Sergiy Popovych, Nico Kemnitz, Dodam Ih, Kisuk Lee, Ran Lu, Akhilesh Halageri, J. Alexander Bae, Ben Jourdan, Gregory Schwartzman, Damian D. Demarest, Emily Behnke, Doug Bland, Anne Kristiansen, Jaime Skelton, Tom Stocks, Dustin Garner, Anthony Hernandez, Sandeep Kumar, The BANC-FlyWire Consortium, Alexander S. Bates, Helen H. Yang, Arie Matsliah, Zaki Ajabi, Eric Perlman, Kevin M. Delgado, Mohammed Abdal Monium Osman, Christopher K. Salmon, Jay Gager, Benjamin Silverman, Sophia Renauld, Janki Patel, Matthew F. Collie, Jingxuan Fan, Diego A. Pacheco, Yunzhi Zhao, Wenyi Zhang, Laia Serratosa Capdevila, Ruairí J. V. Roberts, Eva J. Munnelly, Nina Griggs, Helen Langley, Borja Moya-Llamas, Zuoyu Zhang, Ryan T. Maloney, Szi-chieh Yu, Amy R. Sterling, Marissa Sorek, Krzysztof Kruk, Nikitas Serafetinidis, Serene Dhawan, Finja Klemm, Paul Brooks, Ellen Lesser, Jessica M. Jones, Sara E. Pierce-Lundgren, Su-Yee Lee, Yichen Luo, Andrew P. Cook, Theresa H. McKim, Benjamin Gorko, Justin Ellis-Joyce, Jiayi Zhang, Emily C. Kophs, Tjalda Falt, Alexa M. Negron-Morales, Austin Burke, James Hebditch, Kyle P. Willie, Ryan Willie, Sergiy Popovych, Nico Kemnitz, Dodam Ih, Kisuk Lee, Ran Lu, Akhilesh Halageri, J. Alexander Bae, Ben Jourdan, Gregory Schwartzman, Damian D. Demarest, Emily Behnke, Doug Bland, Anne Kristiansen, Jaime Skelton, Tom Stocks, Dustin Garner, Anthony Hernandez, Sandeep Kumar, Jasper S. Phelps, Minsu Kim, Farzaan Salman, Dimitrios Stasi Giakoumas, Benjamin L. de Bivort, Anna Verbe, Gabriel A. Nieves-Sanabria, Devon Jones, Zijin Huang, Sofia Pinto, Celia David, Omaris Y. De Pablo-Crespo, Emily Ye, Wolf Huetteroth, Zequan Liu, Fernando J. Figueroa Santiago, Kevin C. Daly, Sven Dorkenwald, Forrest Collman, Marie P. Suver, Lisa M. Fenk, Michael J. Pankratz, Zepeng Yao, Fei Wang, Stephen J. Huston, Tomke Stürner, Gregory S. X. E. Jefferis, Katharina Eichler, Andrew M. Seeds, Stefanie Hampel, Sweta Agrawal, Tatsuo S. Okubo, Meet Zandawala, Thomas Macrina, Diane-Yayra Adjavon, Jan Funke, John C. Tuthill, Anthony Azevedo, H. Sebastian Seung, Mala Murthy, Jan Drugowitsch, Rachel I. Wilson, Wei-Chung Allen Lee, Kevin C. Daly, Sven Dorkenwald, Forrest Collman, Marie P. Suver, Lisa M. Fenk, Michael J. Pankratz, Zepeng Yao, Fei Wang, Stephen J. Huston, Tomke Stürner, Gregory S. X. E. Jefferis, Katharina Eichler, Andrew M. Seeds, Stefanie Hampel, Sweta Agrawal, Tatsuo S. Okubo, Meet Zandawala, Thomas Macrina, Diane-Yayra Adjavon, Jan Funke, John C. Tuthill, Anthony Azevedo, H. Sebastian Seung, Benjamin L. de Bivort, Mala Murthy, Jan Drugowitsch, Rachel I. Wilson, Wei-Chung Allen Lee

**Affiliations:** 1 Department of Neurobiology, Harvard Medical School, Boston, MA USA; 2 Centre for Neural Circuit and Behaviour, University of Oxford, Oxford, UK; 3 Princeton Neuroscience Institute, Princeton University, Princeton, NJ USA; 4 Yikes, Baltimore, MD USA; 5 Department of Biology, West Virginia University, Morgantown, WV USA; 6 Aelysia, Bristol, UK; 7 Beijing Institute for Brain Research, Chinese Academy of Medical Sciences and Peking Union Medical College, Beijing, China; 8 Chinese Institute for Brain Research, Beijing, Beijing, China; 9 Department of Organismic and Evolutionary Biology, Harvard University, Cambridge, MA USA; 10 Center for Brain Science, Harvard University, Cambridge, MA USA; 11 Eyewire, Boston, MA USA; 12 Genetics Department, Leipzig University, Leipzig, Germany; 13 Zoology Department, University of Cambridge, Cambridge, UK; 14 Department of Molecular Genetics and Cell Biology, The University of Chicago, Chicago, IL USA; 15 Department of Neurobiology and Biophysics, University of Washington, Seattle, WA USA; 16 School of Neuroscience, Virginia Tech, Blacksburg, VA USA; 17 Department of Biology, University of Nevada Reno, Reno, NV USA; 18 Department of Biology, University of Florida, Gainesville, FL USA; 19 Molecular, Cellular, and Developmental Biology, University of California Santa Barbara, Santa Barbara, CA USA; 20 HHMI Janelia, Ashburn, VA USA; 21 Department of Neuroscience, Johns Hopkins University, Baltimore, MD USA; 22 Institute of Neuroscience, Center for Excellence in Brain Science and Intelligence Technology, Chinese Academy of Sciences, Shanghai, China; 23 Department of Biological Sciences, Vanderbilt University, Nashville, TN USA; 24 Max Planck Institute for Biological Intelligence, Martinsried, Germany; 25 Institute of Neurobiology, University of Puerto Rico Medical Sciences Campus, San Juan, Puerto Rico; 26 Zetta AI, Sherrill, NY USA; 27 School of Informatics, University of Edinburgh, Edinburgh, UK; 28 Japan Advanced Institute of Science and Technology (JAIST), Nomi, Japan; 29 Molecular Brain Physiology and Behavior, LIMES Institute, University of Bonn, Bonn, Germany; 30 Department of Neuroscience, West Virginia University, Morgantown, WV USA; 31 Allen Institute for Brain Science, Seattle, WA USA; 32 Florida Chemical Senses Institute, University of Florida, Gainesville, FL USA; 33 McKnight Brain Institute, University of Florida, Gainesville, FL USA; 34 Genetics Institute, University of Florida, Gainesville, FL USA; 35 Department of Neuroscience, Zuckerman Mind Brain Behavior Institute, Columbia University, New York, NY USA; 36 Neurobiology Division, MRC Laboratory of Molecular Biology, Cambridge, UK; 37 Department of Biochemistry and Molecular Biology, University of Nevada Reno, Reno, NV USA; 38 Neurobiology and Genetics, Theodor-Boveri-Institute, Biocenter, Julius-Maximilians-University of Würzburg, Am Hubland, Würzburg, Germany; 39 Computer Science Department, Princeton University, Princeton, NJ USA; 40 F. M. Kirby Neurobiology Center, Boston Children’s Hospital, Harvard Medical School, Boston, MA USA; 41 Champalimaud Neuroscience Programme, Champalimaud Centre for the Unknown, Lisbon, Portugal; 42 Department of Biology, Leipzig University, Leipzig, Germany; 43 RWTH Aachen University, Aachen, Germany; 44 Present Address: Neuroengineering Laboratory, Brain Mind Institute and Institute of Bioengineering, EPFL, Lausanne, Switzerland; 45 Present Address: Department of Molecular and Cellular Biology, Harvard University, Cambridge, MA USA; 46 Present Address: Center for Brain Science, Harvard University, Cambridge, MA USA; 47 Present Address: Department of Neurobiology, Harvard Medical School, Boston, MA USA; 48 Present Address: Psychology Department, Colorado College, Colorado Springs, CO USA; 49 Present Address: Interdisciplinary Graduate Program in Neuroscience, University of Iowa, Iowa City, IA USA

**Keywords:** Neural circuits, Reflexes, Reward

## Abstract

Just as genomes revolutionized molecular genetics, connectomes (maps of neurons and synapses) are transforming neuroscience. To date, the only organisms with complete connectomes are worms^1–3^, sea squirts^4^ and comb jellies^5^ (10^3^–10^4^ synapses). By contrast, the fruit fly is more complex (10^8^ synaptic connections), with a brain that supports learning and spatial memory^6,7^ and an intricate ventral nerve cord analogous to the vertebrate spinal cord^8–12^. Here we report a densely reconstructed adult fly connectome that unites the brain and ventral nerve cord, and we leverage this resource to investigate principles of neural control. We show that effector neurons (motor neurons, endocrine cells and efferent neurons targeting the viscera) are primarily influenced by sensory neurons in the same body part, forming local feedback loops. These local loops are linked by long-range circuits that involve ascending and descending neurons organized into behaviour-centric modules. Single ascending and descending neurons are often positioned to influence the voluntary movements of multiple body parts, together with the endocrine cells or visceral organs that support those movements. Brain regions involved in learning and navigation supervise these circuits. These results reveal an architecture that is distributed, parallelized and embodied, reminiscent of distributed control architectures in engineered systems^13,14^.

## Abstract (web summary)

A connectome of the brain and ventral nerve cord of an adult female fruit fly is reconstructed and annotated, providing an open resource that reveals neural control of behaviour to be distributed, parallel and embodied.

## Main

A coherent understanding of the embodied nervous system remains a central challenge of neurobiology. The fruit fly *Drosophila melanogaster* is the most complex organism for which this milestone is currently within reach. The *Drosophila* brain and ventral nerve cord (VNC) are analogous to the brain and spinal cord of vertebrates but contain fewer neurons (140,000 and 20,000 neurons, respectively), making them tractable for complete connectomes. The brain and VNC in the fly are connected by about 1,300 descending neurons (DNs) and around 1,800 ascending neurons (ANs)^15^. However, the existing connectomes of the fly brain^16–19^ and VNC^8–12^ were collected separately, and only a minority of DNs and ANs have been reconstructed via ‘bridging’ analyses^15^. A unified *Drosophila* connectome would enable us to trace the pathways that connect the brain, VNC and body.

Such a connectome would also shed light on the architecture of behavioural control. In principle, behavioural control might flow through a central pathway for perception, action selection and motor coordination. Alternatively, it might be decentralized and distributed across many feedback control modules that are loosely coupled in a hierarchical manner. These alternative scenarios are debated in the literature on vertebrate intelligence, insect intelligence and artificial intelligence^14,20–26^. A unified connectome would place important constraints on this debate.

In this study, we describe a unified and embodied brain-and-cord connectome of an adult fly. To analyse this dataset, we develop an ‘influence’ network metric to estimate the magnitude of the functional connection between any pair of cells, and we apply this at scale to the entire nervous system. We show that the strongest influences on effector neurons are generally local sensory signals, forming a distributed set of tight feedback loops. Long-range connections involving ANs and DNs coordinate these local loops. Many of these AN and/or DN (AN/DN) circuits can be linked to specific behaviours, such as escape, feeding, reproduction and locomotion. We describe the interactions between these circuits, and we explicitly link these circuits to supervisory brain regions involved in learning and navigation. Our results establish empirical support for theories of behavioural control organized around distributed sensory-motor modules, in which cognitive regions are supervisory but not essential for action.

## A unified open-source connectome

**Fig. 1:**
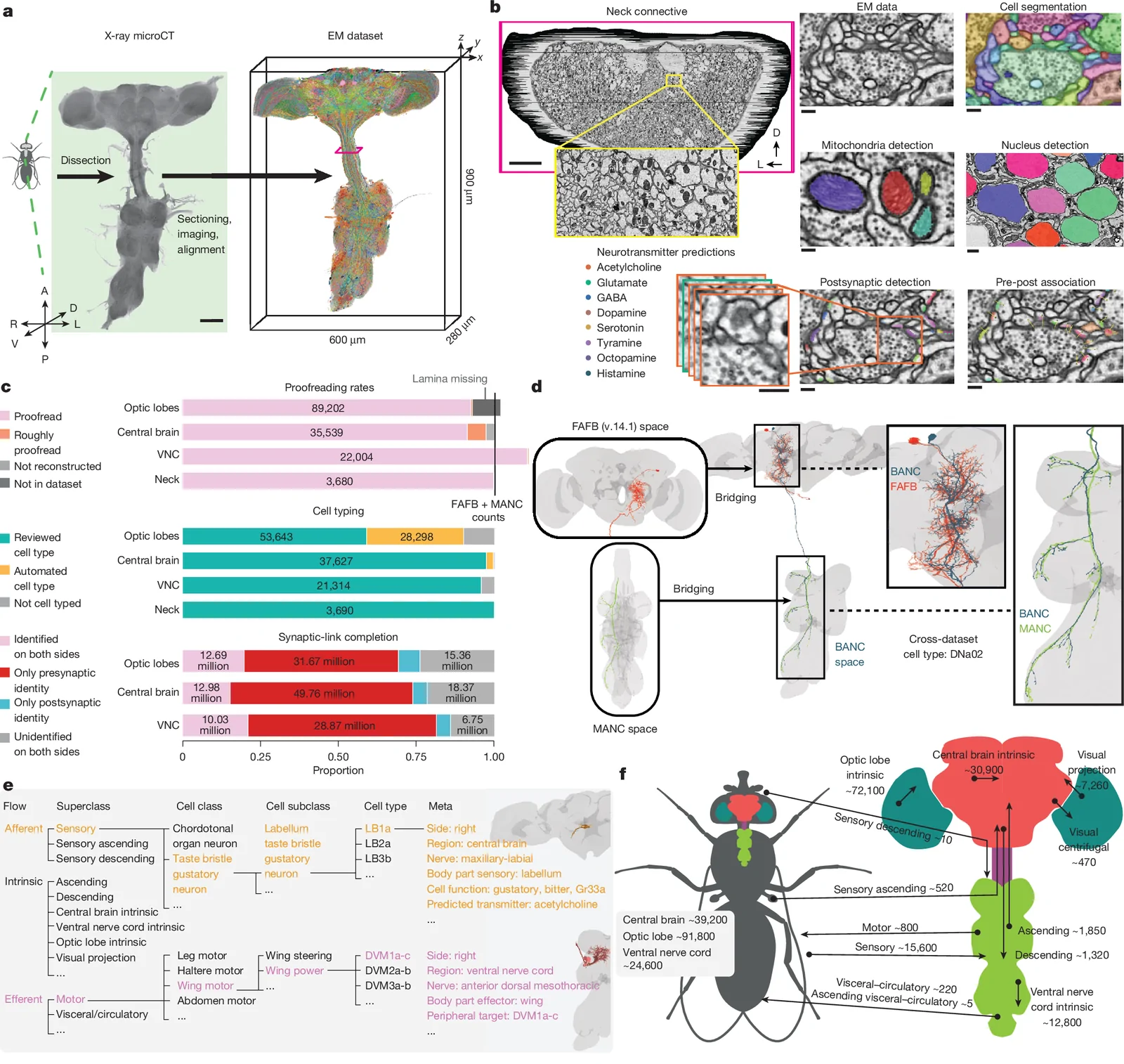
A brain-and-nerve-cord connectome. **a**, Left, X-ray micro-computed tomography (microCT) projection of the BANC sample, an adult female *D. melanogaster*. Right, rendering of the largest 500 cell types by volume. A, anterior; D, dorsal; L, left; P, posterior; R, right; V, ventral. **b**, Top left, aligned EM micrographs through a cross-section of the neck connective (magenta box in **a**), seeded at *y* = 92500 (https://ng.banc.community/2026a/neck-connective). Yellow box, zoom-in of the EM data. Right columns, example EM image data, cell segmentation, mitochondria detection (*x* = 137533, *y* = 35220, *z* = 2493; https://ng.banc.community/2026a/example-mitochondria), nucleus detection (*x* = 192977,* y* = 51679, *z* = 2493; https://ng.banc.community/2026a/example-nuclei), postsynaptic locations (*x* = 140988, *y *= 36705, *z* = 2498; https://ng.banc.community/2026a/example-synapse) and presynaptic locations (Extended Data Fig. 1). Bottom left, the type of neurotransmitter was predicted for each presynaptic site (Extended Data Fig. 3). **c**, Top, fraction of neurons across the CNS that have been proofread based on expected cell counts for the brain^17^, VNC^12^ and neck connective^15^, as indicated by the black line labelled FAFB + MANC counts. The R1–R6 photoreceptors and the lamina intrinsic cell type Lai (around 9,390 neurons) are missing from the BANC connectome because the lamina are not in the dataset. Middle, fraction of proofread and roughly proofread neurons in the BANC dataset. Neurons were matched to metadata from previous projects by transforming morphologies from other connectomes^9,11,12,16,17,19^ into BANC space. Reviewed cell type means manually evaluated, whereas automated cell type indicates a morphology-to-connectivity algorithm hit. Bottom, fraction of synapses with identified presynaptic and postsynaptic partners (171,512 accounted-for objects, including proofread, roughly proofread and neuronal fragments large enough to be cell typed or marked as glia or trachea). We used NBLAST^30^ to identify morphological matches. **d**, An example of cross-dataset matching using DNa02 (https://ng.banc.community/2026a/DNa02). **e**, Hierarchy of cell annotations (Supplementary Data 1). Examples are shown for LB1a (https://ng.banc.community/2026a/LB1a) and DVM1a-c (https://ng.banc.community/2026a/DVM1a-c). **f**, Number of proofread and roughly proofread neurons (totalling 155,916) in the BANC dataset by region and superclass. Scale bars, 100 µm (**a**, left), 10 µm (**b**, neck connective image), 1 µm (**b**, nucleus detection image) and 200 nm (**b**, all other images). Codex links for this figure are listed in the Supplementary Information.

**Extended Data Fig. 1:**
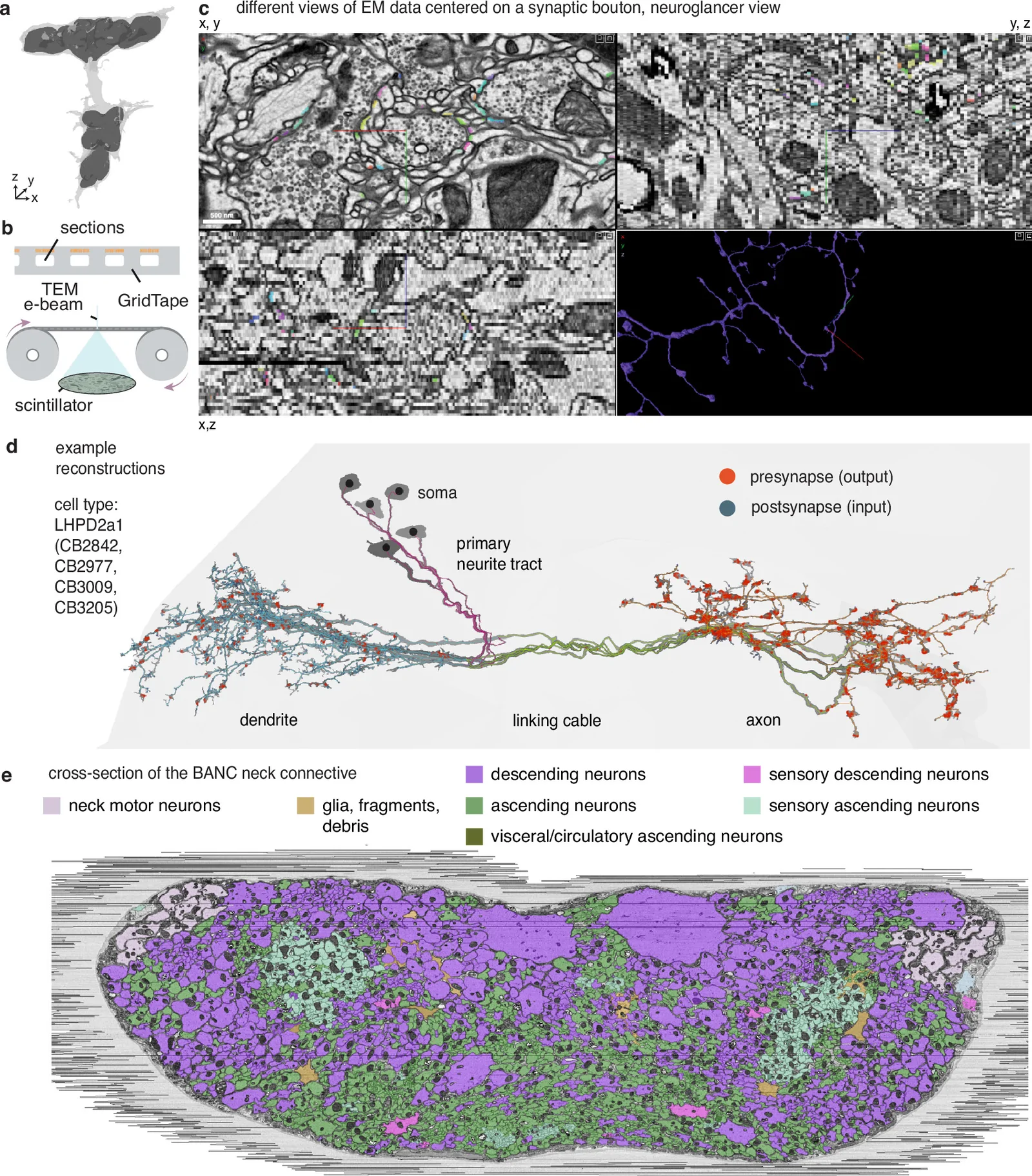
Central nervous system connectome generation and example reconstructions. **a**, Surface rendering of the BANC CNS (grey; includes neuropil as well as cell bodies, neck connective, and nerves); neuropil surface (black), from kernel density estimate of all predicted synapses. **b**, Overview of the GridTape^8^ imaging approach. Serial EM sections are automatically collected onto a tape-based substrate and subsequently imaged in a transmission electron microscope (TEM) with a custom reel-to-reel imaging stage. **c**, Different views of cross-sections around a synaptic bouton in neuroglancer, shown at 4 × 4 × 45 nm^3^, in xyz. Note that the public instance downscales data to 8 × 8 × 45 nm^3^ in xyz. (*Lower right*) lines point to the bouton location in the neuronal arbor. **d**, Example reconstructions of identified LHPD2a1^326^ neurons (grey meshes), with skeletonizations (lines), root points (black dot), synapse detections (blue = input, red = output) and compartment determinations^113^ shown. **e**, Cross-section of the neck connective from BANC.

We generated a serial-section electron microscopy (EM) volume of the connected brain and nerve cord from an adult female *D. melanogaster* at synapse-level resolution (4 × 4 × 45 nm^3^) (Fig. 1a and Extended Data Fig. 1a). Using our semi-automated sectioning and imaging platform (GridTape)^8^ (Extended Data Fig. 1b), we collected 7,010 serial sections onto film-coated tape compatible with transmission EM. This approach enabled visualization of fine neural processes (<200 nm), synaptic vesicles (around 40 nm) and synaptic clefts (around 10–20 nm) (Fig. 1b and Extended Data Fig. 1c,d). After imaging each section, we computationally reassembled the entire Brain And Nerve Cord (BANC) dataset into a 3D volume. We then used convolutional neural networks (CNNs) to automatically segment and reconstruct individual cells, nuclei and mitochondria (Fig. 1b). To proofread and annotate the expected^11,17^ approximately 160,000 neurons in the dataset, we followed the approach created by FlyWire for the whole-brain connectome of a full adult fly brain (FAFB)^17,27,28^. We used automatically identified nuclei to account for all neurons with cell bodies in the central nervous system (CNS). For neurons with cell bodies outside the CNS (for example, sensory neurons), we manually identified nerves leaving the dataset and verified that each axon in these nerves was associated with a segmented neuron. For neurons traversing the neck connective (Extended Data Fig. 1e), we verified that every axon at both anterior and posterior neck levels was associated with a segmented neuron. As in the independent maleCNS project^29^, both the lamina and the ocellar ganglion are missing; however, these regions are present in the FAFB dataset^18^. A team of 155 proofreaders corrected errors in the automatic segmentation over about 3.5 years, a total effort of around 38.6 person-years (Fig. 1c).

**Extended Data Fig. 2:**
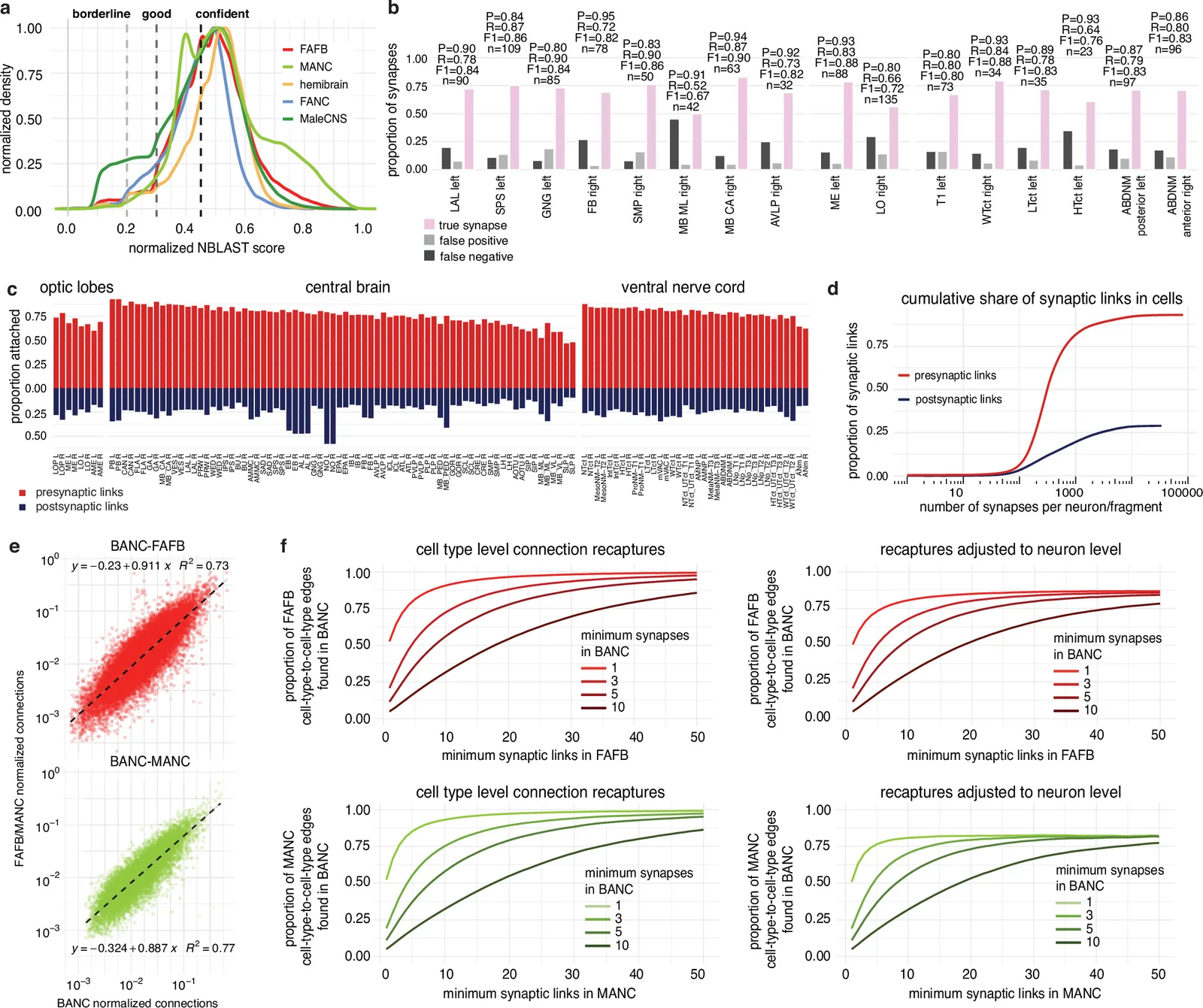
BANC connectome quality and neuron identification. **a**, Density of the normalized NBLAST scores^30^ of ‘backbone proofread’ neurons^17^ in the BANC against all neurons in other connectomic datasets (different colours). We consider normalized NBLAST scores > 0.3 as high and suggest score bins to help guide data users (dashed lines). Normalized NBLAST scores are ‘raw’ NBLAST scores divided by the self-match score. All density curves are normalized to their own peak. FAFB is a dense female brain reconstruction. MANC is a dense male VNC reconstruction. Hemibrain is a dense reconstruction of ~⅓ of the central brain. FANC is a sparse female VNC reconstruction. MaleCNS is a dense male brain and nerve cord reconstruction. **b**, Evaluation of synapse predictions by comparison of predicted synapses with those manually identified in 16 cutouts distributed across the CNS. (*P*, precision; *R*, recall; *F1*, *F*-score). **c**, Attachment rates for presynaptic (red) and postsynaptic (navy) links to an ‘identified’ neuron (171,512 neurons, including proofread, roughly proofread, and neuronal fragments large enough to be cell typed or marked as glia/trachea; note 13,108 cells are glia or trachea). We used the BANC synapse version: synapses_v2 and BANC segmentation and metadata version: v888. Note that a synapses_v3 is available for users and is reported in ref. ^31^. **d**, The cumulative share of pre- and postsynaptic links in identified neurons versus orphan fragments (not part of an identified neuron). Plot is by neuron/fragment size as inferred from the number of synaptic links on the neuron/fragment (v888). **e**, Scatter plots show the correlation between matched pairs of connected cell types in the BANC versus FAFB (red) and MANC (green). Each point is a cell-type-to-cell-type normalized connection (synaptic connections from source-to-target / total number of postsynaptic links on the target cell type). FAFB-BANC: 483,957 matched cell type connections, MANC-BANC: 434,357 matched cell type connections. **f**, Rates of connection recapture between the FAFB (*top*) and MANC (*bottom*) datasets and BANC. Both cell type level connections (*left*), and an adjustment for neuron-level connections (*right*) are shown. Member-weighted recovery: for each cell type pair with at least X synapses in FAFB, we compute the BANC-to-FAFB connection-density ratio scaled by the number of FAFB neuron-pairs in that pair (i.e. the expected number of BANC edges if BANC matched FAFB density × the number of FAFB neuron-pairs), capped at 1, and average across pairs. This is closer to the probability of finding any BANC edge in a high-volume FAFB pair than a proportion of FAFB edges recovered. Only cell types present bilaterally in both datasets and their common edges are evaluated.

Rather than typing most neurons de novo, as previous connectomes did, we assigned cell-type labels using multiple methods to automatically identify potential matches between neurons in the BANC dataset and earlier datasets^9–11,16,17,19^. We performed morphology matching with NBLAST^30^ followed by manual review, graph alignment based on connectivity^31^ or a new annealing algorithm that combines neuron morphology with connectivity data (Fig. 1c–e and Extended Data Fig. 2a). We linked 147,846 neurons to a cell-type label (93.1% in the central brain and VNC) used in another connectomic dataset or the literature. Remaining neurons were challenging to match owing to inter-dataset variability in cell morphology^32–34^. Inter-dataset variability can result from genetic variation, developmental noise and limitations in data quality or reconstruction. In making these cell-type assignments, we also generated a comprehensive accounting of DN and AN cell types across the neck connective, and we matched AN and DN cell-type labels across the FAFB-FlyWire whole-brain connectome^17^ and VNC connectomes^9–11^.

To automatically identify synapses in the BANC dataset, we trained two CNNs^35^ to predict presynaptic and postsynaptic locations with high accuracy (*F*-score = 0.83, precision = 0.87, recall = 0.78; Extended Data Fig. 2b). Overall, 74% of detected presynaptic links and 23% of postsynaptic links have been attached to identified (proofread and/or cell typed) cells; 18% of synapses have identified cells on both sides (Fig. 1c and Extended Data Fig. 2c,d). A comparison of the normalized synaptic count between all pairs of cross-matched, identified cell types in the CNS revealed strong concordance between the BANC dataset and other datasets (Extended Data Fig. 2e,f).

**Extended Data Fig. 3:**
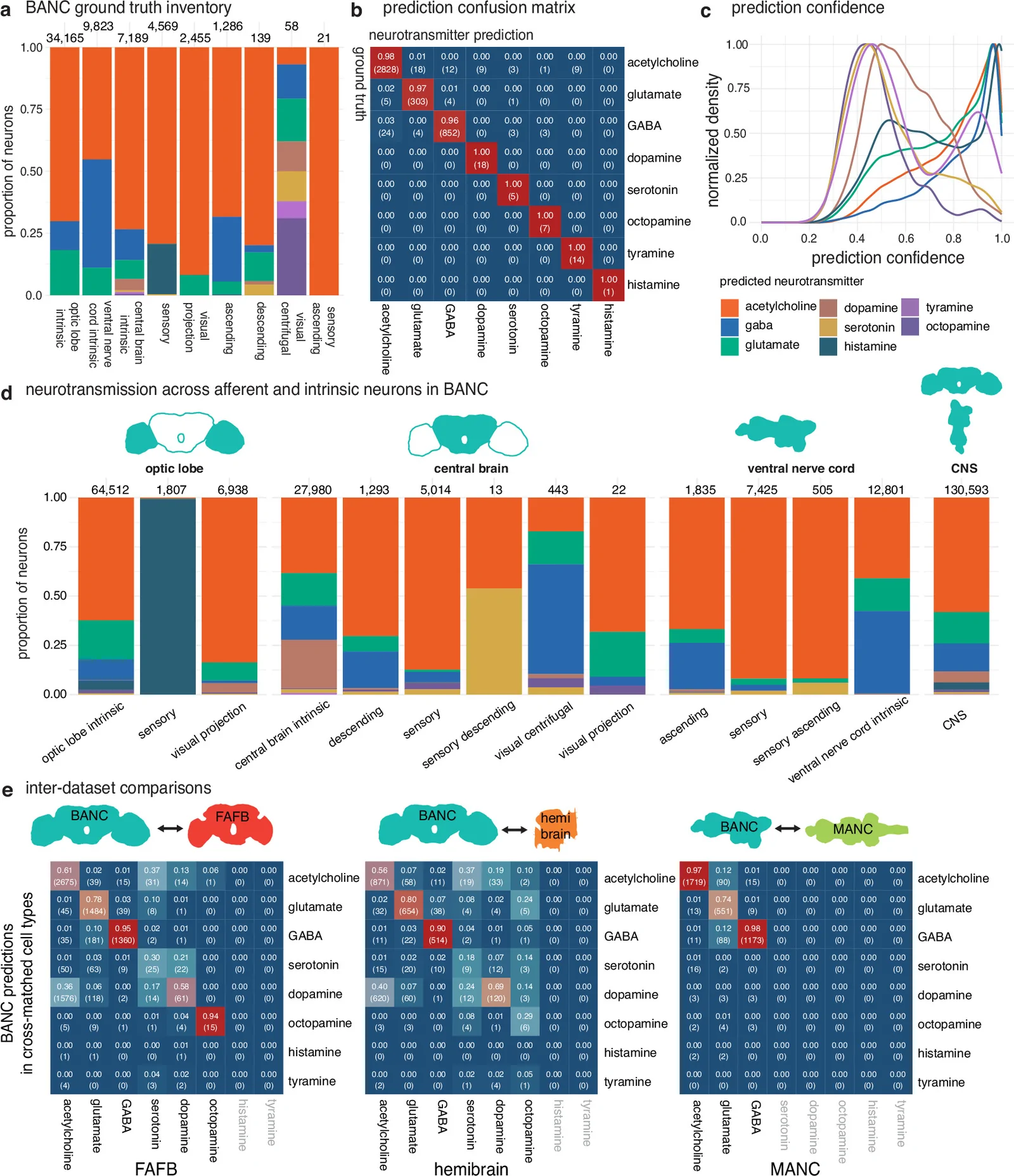
Expanded neurotransmitter predictions across the CNS. **a**, Stacked proportions of verified neurotransmitters for each super class (known co-releasing neurons not included). Effector neurons are removed, as their output synapses are mostly outside of the dataset. Numbers over the bars are total neuron counts. **b**, Confusion matrix of the neurotransmitter predictions evaluated at the level of whole neurons on the held-out test set. Whole neuron prediction is based on the summed classification probabilities across all presynaptic links, selecting the most confident class. The ground-truth included 60,394 neurons (from 3,379 cell types, see Methods), of which 16,448 were used for model training and 4,124 for testing. **c**, Normalized density of neuron-level prediction scores (the winning proportion of classified presynaptic links that determined the neuron-level prediction) across proofread, non-effector neurons. **d**, Stacked proportions of predicted neurotransmitters for each super class and region combination, plus a single CNS summary bar, for all BANC proofread neurons with at least 50 presynaptic links. Effector neurons are removed, as their presynaptic links are mostly outside of the dataset. Kenyon cells are mostly mispredicted as dopaminergic, as in FAFB and hemibrain^36^, and so are shown in a separate colour for clarity. Numbers over the bars are total neuron counts. **e**, Cell type modal neurotransmitter comparison between BANC (rows) and each external dataset (columns): FAFB^36^, hemibrain^36^, MANC^12^. Only cell types present in both datasets are included. Greyed out transmitters were not predicted in the given dataset.

We used another CNN to predict the neurotransmitter released by each neuron^36^ (Extended Data Fig. 3a–d). Our identifications of neurons releasing acetylcholine, glutamate, GABA, dopamine, serotonin and octopamine largely agree with previous predictions^12,36^ (Extended Data Fig. 3e). We also used this approach to identify cells that release tyramine and histamine, which have not been previously predicted by CNNs (Extended Data Fig. 3a–d).

Next, we identified the majority of cell types that link the CNS with the rest of the body (Fig. 1c–f). For example, we identified motor neurons targeting muscles of the legs, wings, halteres, antennae, eyes, neck, crop, pharynx, proboscis, salivary glands and uterus. We found sensory nociceptors from the abdomen, sensory neurons from the aorta, sensory neurons from the cibarium (the pre-oral food chamber), oxygen-sensing neurons in the abdomen and sensory neurons from the abdominal terminalia. We identified multiple distinct types of endocrine cells in the brain and VNC, many of which could be matched with the neuropeptides they release and their sites of action, including the ureter, digestive tract, reproductive tract and neurohemal release sites. We also identified chemosensory, tactile and proprioceptive afferents from the head, eyes, antennae, proboscis, legs, abdomen, wings and halteres. Taken together, these cell-type identifications make the BANC dataset a highly ‘embodied’ connectome, with explicit connections to specific muscles, sense organs and viscera throughout the body.

We were able to describe all these connections because the BANC connectome has been an open-science effort since July 2023, and the project involves many experts in the field. This endeavour continues to grow with ongoing community input. Users can visualize an archived version of our image data on BossDB (10.60533/boss-2025-941r), and the latest version is available at Neuroglancer (https://ng.banc.community/view). Users can add annotations through the Connectome Annotation Versioning Engine (CAVE)^28^. They can also browse metadata and connectivity via FlyWire Codex and CAVE and matching data via NeuronBridge^37^ as well as with our programmatic tools. Neuron morphologies, synapse detections, annotations, indirect connectivity scores, analysis code and more are available as direct downloads from the Harvard Dataverse, a digital object identifier minting repository. We have also modified typology annotations (for example, superclass, cell class, cell subclass, cell function and body part) for the FAFB-FlyWire and Male Adult Nerve Cord (MANC) connectomes^11,12,17–19^ to facilitate comparisons between these datasets and the BANC dataset (Supplementary Data 1–5).

## A metric of influence

To interpret a whole brain-and-cord connectome, we need a way to estimate the influence of neuron A on neuron B for any pair of neurons. So far, there has not been a computationally efficient method of estimating these influences. Efficiency is crucial, as there are billions of pairwise interactions between neurons in the entire CNS.

**Fig. 2:**
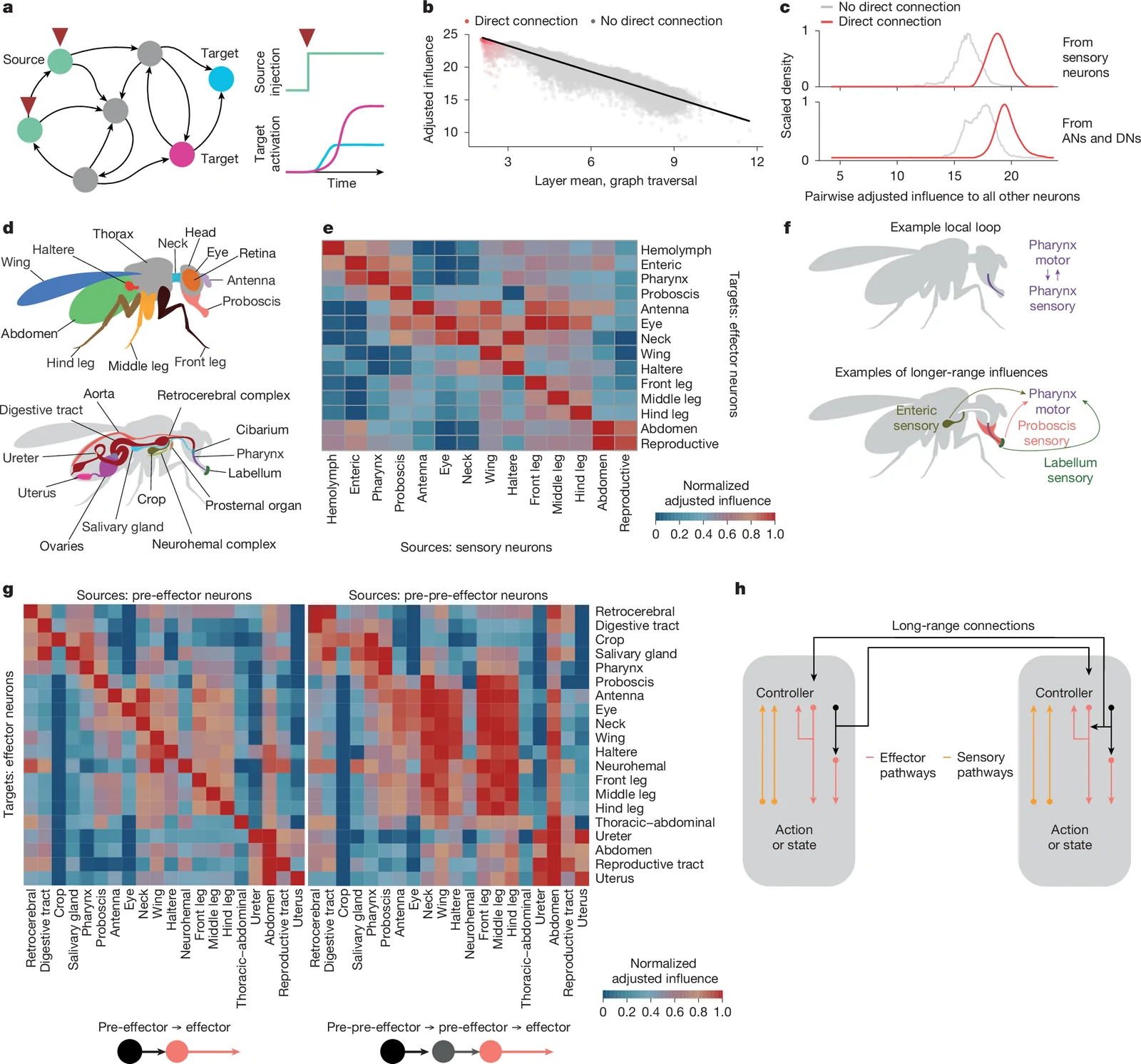
Linking sensors and effectors through local and long-range circuits. **a**, The influence of source cells on target cells is estimated via linear dynamical modelling. **b**, Adjusted influence scores are proportional to the number of network layers in a graph traversal model^32^. Here the source neurons are olfactory receptor neurons in the FAFB (v.783) dataset^19^, and adjusted influence is averaged over neurons in the source and target groups (for which targets are defined as all other FAFB neurons). The regression line is in black (*R*^2^ = 0.94, *n* = 94,278 neuron pairs). **c**, Distributions of adjusted influence from each sensory neuron onto all other neurons (top) or from each AN and DN onto all other neurons (bottom) (*n* = 171,512 target neurons). Each distribution is normalized to its own maximum. Note, the underlying graph used was thresholded at a synaptic count of ≥5. Overlap is proportionally small but numerically large: 149,139 indirect connections and 70,753 direct connections from sensory are over the 25th percentile of influence for direct connections from sensory neurons. At lower thresholds, these distributions overlap more. **d**, Body parts associated with effector neurons. Not all neurohemal organs are shown (https://ng.banc.community/2026a/efferent-body-parts). **e**, Adjusted influence of sensory neurons onto effector neurons by body part. Each row is normalized to a range of 0–1. Adjusted influence is significantly higher within a body part than across body parts (*W* = 2,535.5, *P* = 3.49 × 10^–10^, one-sided Wilcoxon rank-sum, *n* = 14 same-body-part versus *n* = 182 different-body-part 14 × 14 matrix entries, *n*_sensory_ = 16,140, *n*_effector_ = 1,033). **f**, Example local loop (top) that is also linked to specific sensors via long-range connections (bottom). **g**, Adjusted influence of pre-effector neurons on effector neurons (left) and pre-pre-effectors on effector neurons (right). A pre-effector is a neuron directly presynaptic to an effector neuron; a pre-pre-effector is defined analogously. Source × target interactions are significant for both (*P* < 1 × 10^–15^, two-way analysis of variance (ANOVA) (type III sum of squares), *n*_effector_ = 1,031, *n*_pre-effectors_ = 8,824, *n*_pre-pre-effectors_ = 16,838). **h**, Sensory neurons and effector neurons are connected within functional modules, largely via intrinsic neurons. Effector neuron pathways also influence each other, particularly within a module. Modules are weakly connected via long-range connections. Within each controller, the intrinsic circuitry of the CNS also shapes motor output (for example, via central pattern generators).

**Extended Data Fig. 4:**
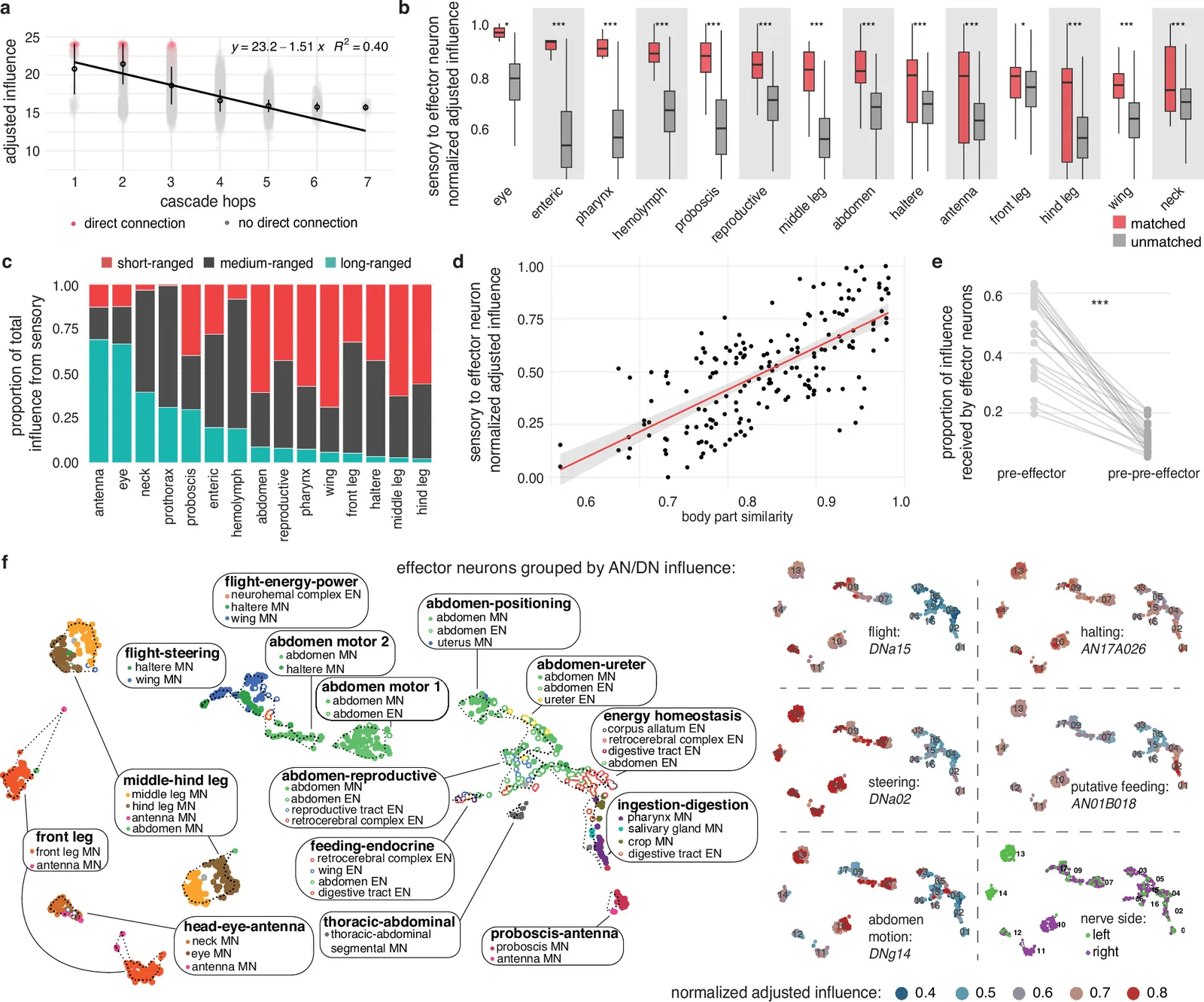
Extended analysis of influence. **a**, Adjusted influence is proportional to the output of the signal cascade algorithm^41^. As in Fig. 2b, the source neurons are olfactory receptor neurons. Mean ± 1 S.D. shown. **b**, Boxplots for each body part comparing adjusted influence (Eq. 10) values from sensory neurons to effector neurons, grouped by whether the sensor and effector body parts are the same (matched) or different (unmatched). Normalized by target, as in Fig. 2e. Matched sensor-effector pairs have significantly higher influence across all body parts (* *P* < 0.05, *** *P* < 0.001, one-sided Wilcoxon rank-sum with Holm correction, *n* = 14 body parts). Box plots: centre line, median; box, 25th–75th percentile (IQR); whiskers, 1.5× IQR; outliers not shown. **c**, Division of sensory influence onto effector neurons (grouped by body part). Sensory influence is categorized as short-ranged (same body part), medium-ranged (sensory and effector neurons are both in the brain or both in the VNC but have different body parts), or long-ranged (sensory neuron is in the brain and effector neuron is in the VNC or vice versa). **d**, Scatterplot shows normalized, adjusted influence values from Fig. 2e – i.e., between effector body part pairs – versus the mean cosine similarity between effector body parts – i.e., calculated between the columns in Fig. 2e. Same body part pairs have cosine similarity = 1 by definition and are omitted. We find a significant positive relationship between body part similarity and influence (*r* = 0.70, *R*^2^ = 0.497, *P* = 1.18 × 10^–28^, two-sided Pearson correlation, *n* = 182 pairs from 14 body parts). In grey, 95% confidence interval of the fitted linear regression. **e**, Proportion of influence effector neurons of a body part receive from pre- and pre-pre-effector neurons of their matched body part. ‘Pre’ indicates neurons directly upstream of an effector neuron (≥5 synaptic links); ‘pre-pre’ indicates neurons directly upstream of a pre-effector neuron (i.e., two connections upstream of an effector neuron). The proportion of influence effector neurons receive from pre-pre-effector neurons is significantly less than that from pre-effector neurons (*P* = 9.54 × 10^–7^, paired two-sided Wilcoxon signed-rank test, *n* = 21 body parts). **f**, DNs and ANs often co-regulate effector neurons in different body parts. (*Left*) Cosine similarity UMAP of effector neurons (Supplementary Data 7) based on the AN/DN influence that they receive. Note that DNs and ANs co-regulate pharynx motor neurons, salivary gland motor neurons, crop motor neurons, and endocrine neurons acting on the digestive tract; these effector neurons are labelled ‘ingestion-digestion’. Similarly, DNs and ANs coordinate wing power motor neurons, haltere motor neurons, and endocrine neurons of the neurohemal complex; these effector neurons are labelled ‘flight-energy-power’. (*Right*) the same effector neuron maps, coloured by normalized adjusted influence from example ANs and DNs (minmax normalized, rescales influence values to 0–1, across all AN/DNs). This illustrates the influence distribution rather than magnitude. (*Lower right*) the same effector cell map, coloured by the side of the CNS on which their efferent axon exists.

To tackle this problem, we developed an approach based on a linear dynamical systems description of signal propagation^38,39^. Specifically, to compute the influence of one or more source neurons on any target neuron or neurons, we determine the effect of injecting a sustained signal into the source neurons, taking the ‘activation’ of each downstream neuron as the weighted sum of its inputs. The weight is the number of synapses in that input connection^40^, as a fraction of the total synaptic input of the postsynaptic cell. For a target cell of interest, we take its steady-state response (Fig. 2a), log-transform it and add a constant to ensure that the result is non-negative. The metric (called adjusted influence) is inversely proportional to the network distance from the source to the target (Fig. 2b and Extended Data Fig. 4a). The adjusted influence is in close agreement with previous network distance metrics^17,32,41^, and like these metrics, adjusted influence is an unsigned quantity. However, unlike those metrics, it is deterministic, linear and computationally efficient. For example, our metric does not involve manually assigning layers of neurons for expensive connectivity matrix multiplications (unlike the effective connectivity metric)^7^. In essence, our metric is simply an efficient way to estimate the effective distance between any pair of cells, and because it is efficient, it is straightforward to scale up to quantify all pairwise distances in the connectome. This enabled us to precompute the pairwise adjusted influence between all BANC neurons, for a total of approximately 24 billion scores. Over the entire dataset, the modal pairwise adjusted influence score was 20 for direct connections and 5 for indirect connections, although the distribution will differ depending on the depth of the selected neurons within the connectome (Fig. 2c). All scores are available via the Harvard Dataverse (10.7910/DVN/7WTH1N), and the code^42^ to compute these scores is available online (10.5281/zenodo.15999929).

In the subsequent sections, we say A influences B as shorthand for a high adjusted influence score (A→B). These scores do not demonstrate functional connections, and they are no substitute for experiments. The value of these scores is that they enable us to make provisional inferences on a large scale. Below, we use influence scores to make inferences, and to support these inferences, we provide example circuit motifs showcasing instances of relatively direct connections.

## Modules for local feedback control

Other large-scale connectome analyses have focused mainly on cells deep in the CNS^6,7,43^. Here we take a complementary approach: we start by focusing on sensors and effectors. A sensor is a presumptive peripheral sensory neuron (either external or internal), whereas an effector is defined here as a presumptive motor neuron, endocrine cell or efferent neuron that targets the viscera (Fig. 2d). Note that sensors are distributed across the body and that effector neurons are also widely distributed. Specifically, the brain contains motor neurons^19^ of the eyes, antennae, mouth parts and foregut, and the VNC contains motor neurons^9^ of the legs, wings, halteres, abdomen, reproductive organs and hindgut^8^. Similarly, endocrine cells are found in both the brain and VNC^44^.

We found that most groups of effector neurons receive their strongest influence from sensors in the same body part (Fig. 2e and Extended Data Fig. 4b). For example, pharynx motor neurons are most strongly influenced by pharynx sensory cells. Naturally, pharynx movements will alter the activity of pharynx sensory neurons. Thus, our results imply that pharynx motor neurons and sensory neurons form a reciprocal feedback loop (Fig. 2f). We found similar loops in almost every body part (Fig. 2e and Extended Data Fig. 4b).

At the same time, each local loop is influenced by more distant sensors in functionally related body parts (Fig. 2e and Extended Data Fig. 4c,d). For example, pharynx motor neurons are influenced by sensors in the crop, labellum and proboscis (Fig. 2e). These longer-range connections can also be seen as forming feedback loops. For example, pharynx movements during feeding should trigger not only sensory signals in the pharynx but also sensory signals in the crop, which might then, for example, limit feeding if the crop is filling too quickly (Fig. 2f). Long-range loops can provide important feedback signals that local loops cannot directly access^45^.

We also found that many effector pathways are positioned to influence multiple effectors, and these patterns are also primarily local. Specifically, we identified all the neurons directly presynaptic to any effector neuron (we call these ‘pre-effectors’); we then computed the influence of each pre-effector onto all effectors. Pre-effectors tend to influence effectors distributed across multiple body parts, but these influences are selective and mainly local (Fig. 2g). We obtained a similar result when we stepped back one layer, starting with the neurons directly presynaptic to any pre-effector (‘pre-pre-effectors’; Fig. 2g). Thus, effector recruitment is coordinated across body parts, but the strongest effector pathways are local, with weaker coupling at longer spatial scales (Extended Data Fig. 4e).

In summary, these analyses support a view in which CNS output is primarily controlled by local modules. Within each module, sensory neurons influence effector neurons (largely indirectly via intrinsic neurons). Meanwhile, effector neurons drive changes in body state, which produces sensory feedback (Fig. 2h). Within each module, effector pathways also influence each other (Fig. 2h). Finally, different modules are weakly coupled via longer-range connections. Our findings argue that actions and sensations are tightly coupled, that behavioural control is highly parallelized and distributed, and that actions are controlled by a combination of sensory reafference and internal expectation signals.

## Linking DNs and ANs to behaviours

**Fig. 3:**
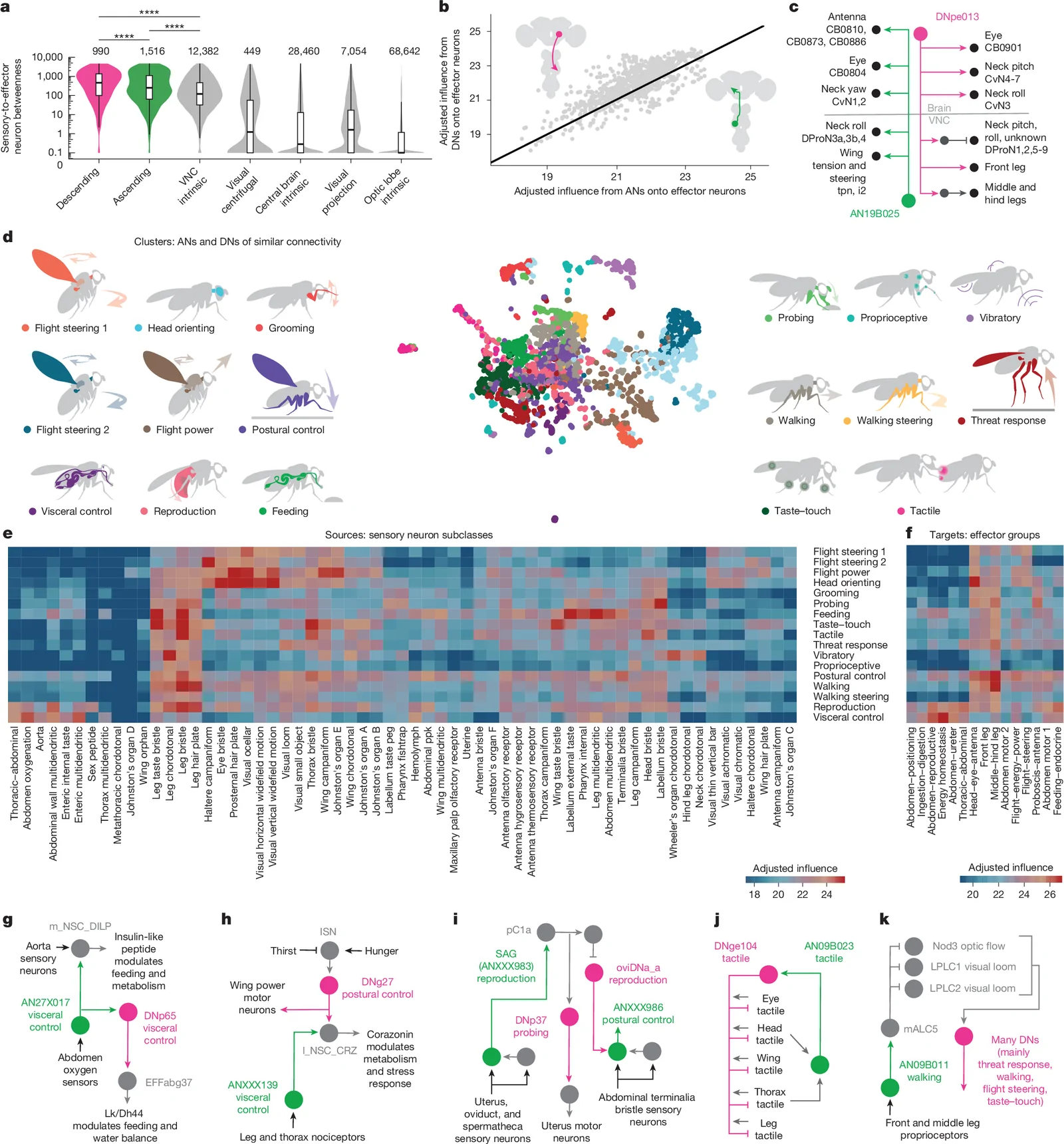
Clustering ANs and DNs into behaviour-centric modules. **a**, Betweenness of selected superclasses, quantifying participation in the number of shortest paths from all sensory neurons to all effector neurons. ANs, DNs and VNC intrinsic neurons have higher betweenness than all other superclasses (*P* < 2.2 × 10^–16^, Kruskal–Wallis, *n* = 77,581). VNC intrinsic neurons have high betweenness because the VNC is enriched in sensory and effector neurons (33% of VNC neurons are sensory and 3.4% are effectors, versus 16% and 0.47%, respectively, in the central brain). ANs, DNs and VNC intrinsic neurons are all significantly different (*P* = 5.41 × 10^–6^, pairwise two-sided Wilcoxon with Holm correction; *n* = 79,916). Box plots: centre line, median; box, 25th–75th percentile (interquartile range (IQR)); whiskers, 1.5× IQR; outliers not shown. Numbers are neuron counts. **b**, Adjusted influence on each effector neuron from DNs (inset, pink) versus ANs (inset, green). Black, unity line. **c**, An example of an AN and a DN with strong adjusted influence on effector neurons in multiple body parts (https://ng.banc.community/2026a/figure-3c). **d**, A uniform manifold approximation and projection (UMAP) embedding, based on the first 319 principal components (PCA) of AN/DN direct connectivity (Supplementary Data 6). Neurons are coloured by cluster membership: clusters (https://ng.banc.community/2026a/an-dn-super-clusters), ANs (https://ng.banc.community/2026a/ascending) and DNs (https://ng.banc.community/2026a/descending). **e**, Adjusted influence onto each AN/DN cluster from select groups of sensory neurons. Clusters are rows; sensory neurons are columns. Select visual projection neurons were used to determine processed visual streams from the optic lobes. **f**, Adjusted influence from each cluster onto co-controlled groups of effector neurons (Extended Data Fig. 4f). Clusters are rows; effector neuron groups are columns. **g**, Example circuit involving visceral control ANs and DNs (https://ng.banc.community/2026a/figure-3g). **h**, Example circuit involving the flight power cluster and the visceral control cluster (https://ng.banc.community/2026a/figure-3h). **i**, Example circuit for coordinated visceral sensing and reproductive control (https://ng.banc.community/2026a/figure-3i). **j**, Example circuit involving a DN and an AN in the tactile cluster (https://ng.banc.community/2026a/figure-3j). **k**, Example circuit involving an AN in the walking cluster (https://ng.banc.community/2026a/figure-3k). Codex links for this figure are listed in the Supplementary Information.

**Extended Data Fig. 5:**
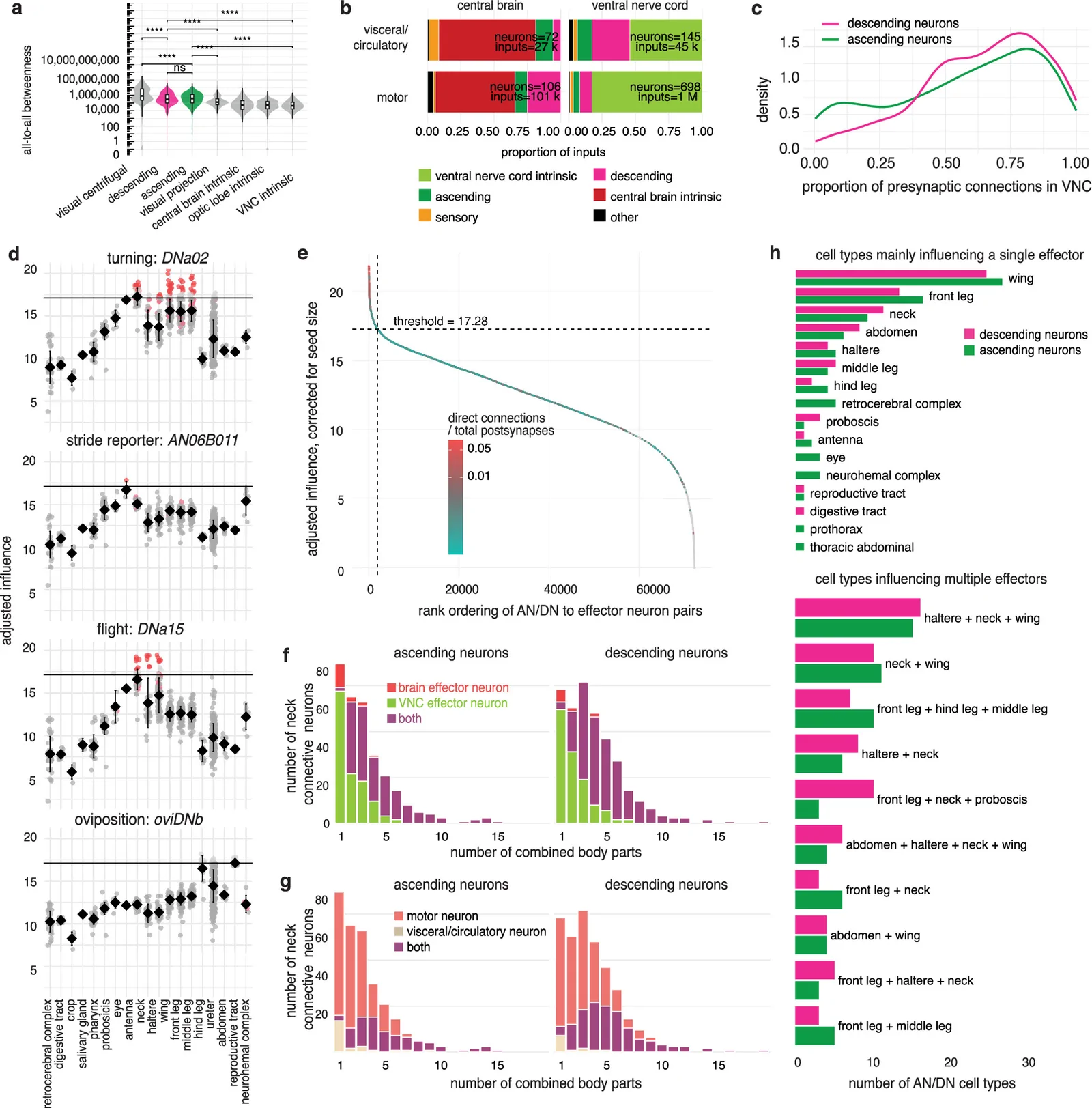
Individual DNs and ANs often influence effectors in multiple body parts. **a**, All-to-all betweenness by super class in the BANC dataset. A neuron’s betweenness is the number of shortest paths between all other proofread neurons in BANC v888 that pass through it. Critically, a neuron can have low degree but high betweenness if it sits between modules. This metric captures a neuron’s potential control over global flow, not just its local connectivity. All-to-all betweenness varies significantly with super class (*P* < 2.2 × 10^–16^, Kruskal–Wallis test, *n* = 119,494 neurons across 7 super classes). Visual centrifugal, ANs, and DNs have significantly higher all-to-all betweenness than other super classes (all *P* ≤ 2.83 × 10^–25^, two-sided Dunn’s post hoc tests with Holm correction, *n* = 119,494 neurons across 7 super classes). Box plots: centre line, median; box, 25th–75th percentile (IQR); whiskers, 1.5× IQR; outliers not shown. **b**, The proportion of input synapses to effector neurons by super class identity of the presynaptic neurons. (*Top*) visceral/circulatory neurons; (*bottom)* motor neurons. (*Left*) effector neurons in the central brain; (*right*) effector neurons in the VNC. **c**, Distribution of presynapses in the VNC versus the brain for all DNs (1,316 cells) and ANs (1,849 cells) in the BANC dataset. **d**, Examples of three DNs and one AN with known behavioural functions, plotting their adjusted influence on effector neurons in different body parts. Within each subplot, each point is an effector neuron, with direct connections in red. The horizontal line marks a value of 17.28, which we determined was a conservative cutoff for ‘high influence’ (see e). All four cells have some effector neuron influence above this cutoff. For each cell, the above-cutoff effector neuron influences are compatible with the cell’s function. Mean ± 1 S.D. shown. **e**, The adjusted influence cutoff as the ‘elbow’ in the cumulative distribution of AN/DN-to-effector neuron adjusted influences (Eq. 10) involving DNs and ANs with known behavioural functions; DNs and ANs used to identify this elbow were AN03A001^327^, AN06B011^328^, AN08B134^323^, AN17A026^289^, AN19A018^73^, AN27X004^10^, AN27X005^327^, AN27X008^10^, AN27X017^295^, ANXXX983^60^, DNa02^287^, DNa01^287^, DNa03^272^, DNa11^272^, DNa12^329^, DNa15^274^, DNae003^272^, DNb01^274^, DNb02^50^, DNb05^98^, DNb06^98^, DNb08^288^, DNg02^276^, DNg07^276^, DNg12^330^, DNg13^98^, DNg14^50^, DNg26^331^, DNg55^73^, DNg62^281^, DNg75^73^, DNg79^292^, DNg97^73^, DNg100^73^, DNge011^281^, DNge012^281^, DNge050^73^, DNge078^281^, DNge172^50^, DNp01^285^, DNp02^286^, DNp03^275^, DNp04^286^, DNp06^284^, DNp07^66^, DNp09^72,290^, DNp10^66^, DNp11^286^, DNp13^61^, DNp15^279^, DNp17^280^, DNp20^277^, DNp22^277^, DNp25^282^, DNp27^166^, DNp37^193^, DNp42^78^, DNp44^282^, DNp47^292^, MDN (DNp50)^291^, oviDNa^61^, oviDNb^61^. **f**, The number of AN and DN cell types that combine different numbers of body parts after indirect connections are binarized by the cutoff in (e). Gross CNS division for combined effector neurons shown in colour (‘both’ can appear when only one body part is targeted because neck motor neurons are found in both the brain and VNC^64^). **g**, Same as (f), but colour indicates whether the combinations incorporate motor neurons, visceral/circulatory neurons, or both. **h**, After discarding connections below the cutoff in (e), we counted the number of AN and DN cell types that influence effector neurons in single body parts (*top*) or multiple body parts (*bottom*). The bottom plot shows only the most common 20 combinations of body parts.

Thus far, we have seen evidence for strong local feedback loops linked by selective longer range sensor–effector connections. To better understand these long-range connections, we focused on the neurons that link the brain with the VNC, namely DNs and ANs. DNs and ANs participate in many of the shortest paths from sensory neurons to effector neurons (Fig. 3a and Extended Data Figs. 4f and 5a).

It is sometimes suggested that DNs send motor commands from the brain to the VNC, whereas ANs send sensory signals and predictive motor signals back from the VNC to the brain^46,47^. However, classical work in other insects has shown that some ANs can send signals from the brain to the VNC^48,49^. Furthermore, in *Drosophila*, more recent work has shown that ANs can form output synapses in the VNC^10,11^, and DNs can form output synapses in the brain^15,50^. The BANC dataset clearly showed that both DNs and ANs have substantial output in both the brain and the VNC (Extended Data Fig. 5b,c). Moreover, it showed that most effector neurons are influenced by both DNs and ANs (Fig. 3b). Most individual DNs exert influence over effector neurons in multiple body parts, and the same is true of ANs (Extended Data Fig. 5d–h). For example, DNpe013 influences motor neurons for the eyes, neck and legs, whereas AN19B025 influences motor neurons controlling the antennae, eyes, neck and wings (Fig. 3c). Together, these observations imply that DNs and ANs work together to coordinate motor patterns and internal organs in different body parts.

## AN/DN behaviour-centric modules

**Extended Data Fig. 6:**
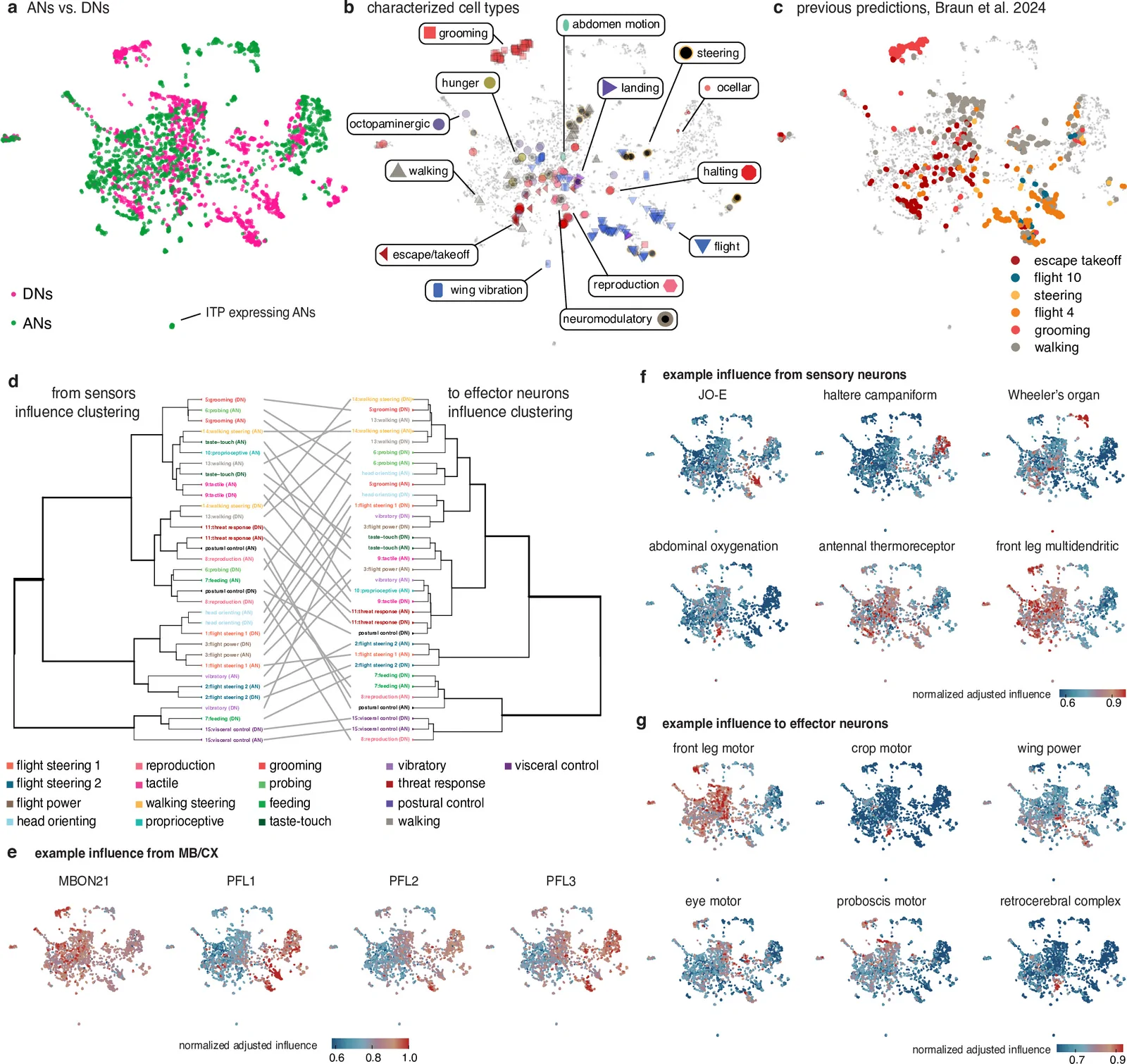
Influence streams to and from AN/DN clusters. **a**, The PCA-UMAP from Fig. 3d; each point is a single AN or DN, coloured accordingly. Near synapse-less ITP-expressing ANs (pers. comm. M.Z.) are highlighted as they form a discrete cluster that we combine with the ‘visceral control’ cluster. **b**, Behavioural functions for characterized cell types, mapped onto the same PCA-UMAP. **c**, The same PCA-UMAP with predicted functions from^50^, which were based on direct DN-DN connectivity in FAFB. **d**, Tanglegram showing the relationship between two methods of sorting AN/DN clusters, splitting ANs and DNs. The left dendrogram sorts clusters based on the similarity of their adjusted influences from sensory neuron sub classes. The right dendrogram sorts clusters based on the similarity of their adjusted influences to effector neuron sub classes. **e**, Examples of normalized, adjusted influence (Eq. 10, source size corrected) from neurons from more ‘cognitive’ brain regions, the mushroom body (MB) and central complex (CX), to ANs and DNs (displayed using the same PCA-UMAP). Minmax normalization rescales influence values to 0–1 across all AN/DNs. This illustrates the influence distribution rather than magnitude. **f**, Examples of normalized, adjusted influence (Eq. 10) from sensory neuron sub classes to ANs and DNs (displayed using the same PCA-UMAP). **g**, Examples of normalized, adjusted influence (Eq. 10) from ANs/DNs to effector neuron cell classes (displayed using the same PCA-UMAP).

**Extended Data Fig. 7:**
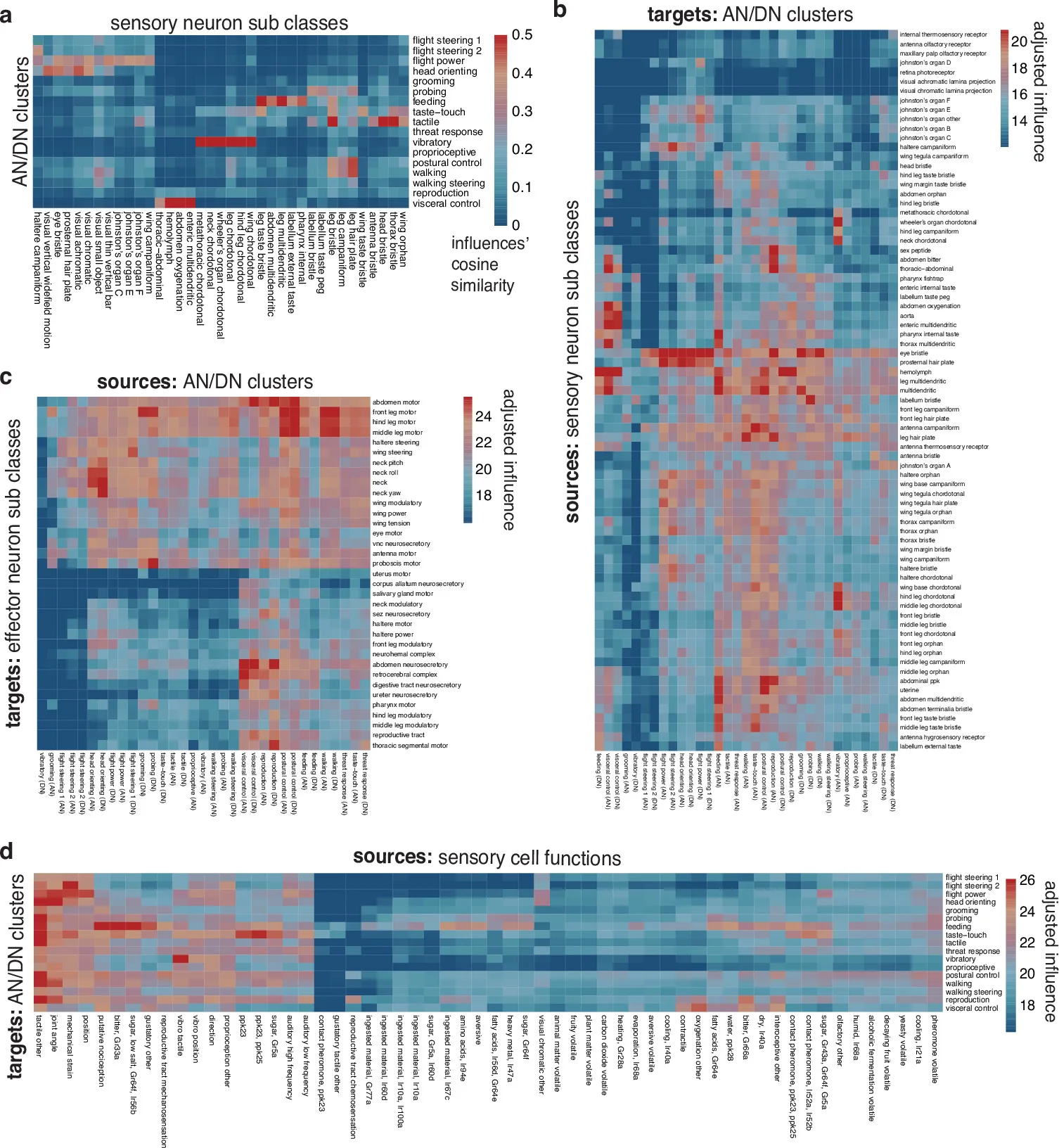
Evidence for AN/DN assignment to behavioural ‘clusters’. **a**, Cosine similarity between sensory neuron sub classes and AN/DN clusters. **b**, Adjusted influence (Eq. 10, source size corrected to control for large variation in the size of source classes) from all sensory neuron sub classes onto AN/DN clusters, splitting ANs and DNs. **c**, Adjusted influence (Eq. 10) from AN/DN clusters, splitting ANs and DNs, onto effector neuron sub classes. **d**, Adjusted influence (Eq. 9, not source-size normalized) from sensory neurons, grouped by cell function, onto effector neuron groups from Extended Data Fig. 4f. See the Harvard Dataverse repository for cell function to ID assignments.

**Extended Data Fig. 8:**
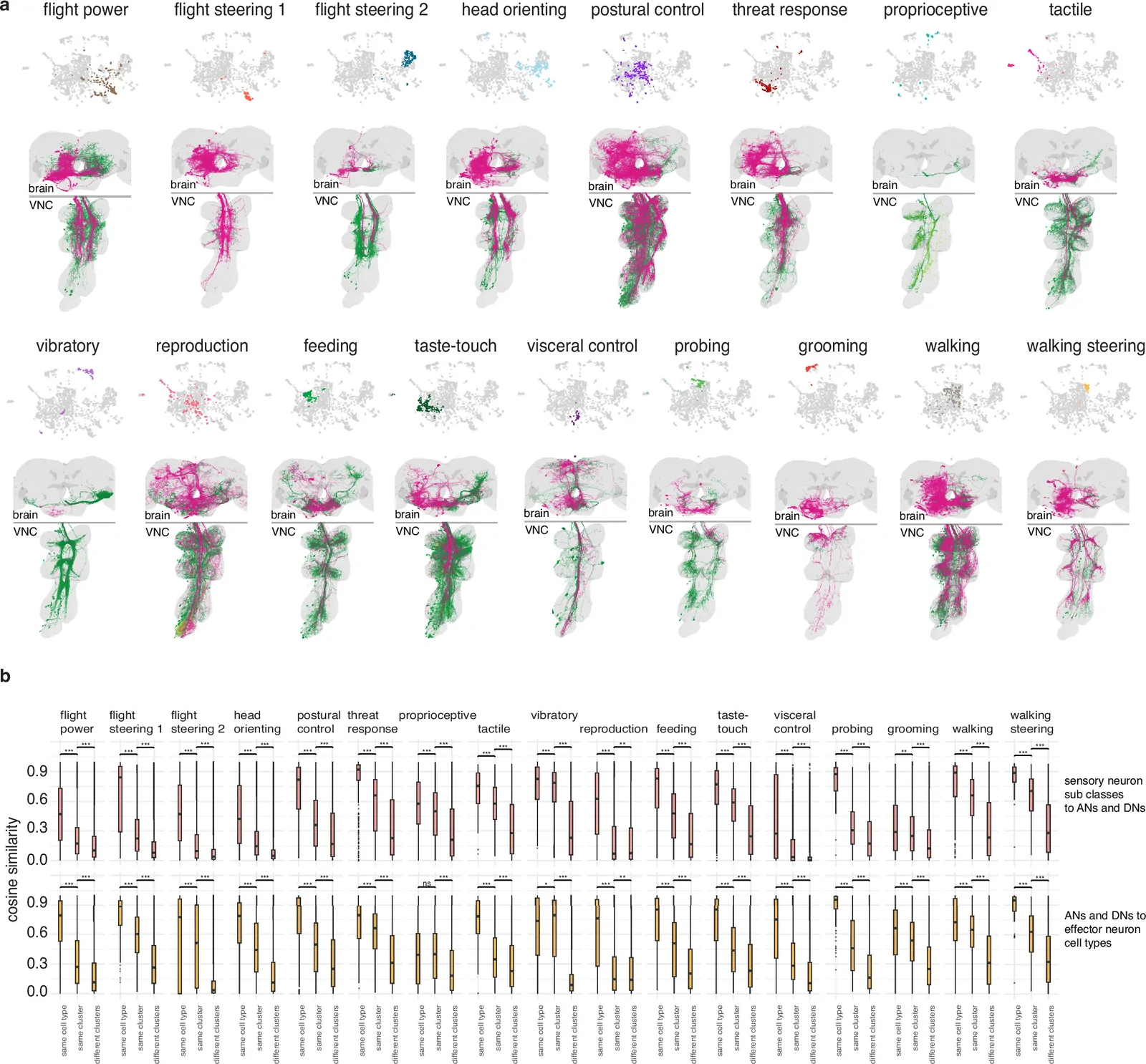
AN/DN morphologies by cluster. **a**, Each subpanel shows neurons from one AN/DN cluster (Fig. 3d), with all neurons of the cluster highlighted in colour in the PCA-UMAP above and the anatomy of neurons in the cluster (but only for cells with somas on the right side of the CNS) displayed below. DNs are in magenta and ANs are in green. See Supplementary Information for Neuroglancer links for flight power, flight steering 1, flight steering 2, head orienting, postural control, threat response, proprioceptive, tactile, vibratory, reproduction, feeding, taste-touch, visceralcontrol, probing, grooming, walking, and walking steering. **b**, Cosine similarity in ‘from sensory’ and ‘to effector’ raw influences for AN/DN clusters. Similarity distributions are shown for the same cell type, same cluster, or different clusters comparisons (***P ≤ 0.001, **P ≤ 0.01, *P ≤ 0.05, n.s. *P* > 0.05 pairwise two-sided Wilcoxon rank-sum tests with Holm correction, *n* = 8,438,583 cluster pairs). Box plots: centre line, median; box, 25th–75th percentile (IQR); whiskers, 1.5 × IQR; outliers not shown.

To identify functional divisions among DNs and ANs, we constructed a map of these neurons on the basis of their direct synaptic connections (both presynaptic and postsynaptic; Fig. 3d and Supplementary Data 6). DNs and ANs are intermingled in this map because, as it turns out, their connections can be similar (Extended Data Fig. 6a). We verified that cells with similar known functions (9.3% of all DNs and ANs) are generally colocalized on this map (Extended Data Fig. 6b,c and Supplementary Data 9). When we divided DNs and ANs into clusters on the basis of their direct presynaptic and postsynaptic connections (Extended Data Figs. 6d–g, 7 and 8a), we found that we could link many clusters with a behaviour (Fig. 3d).

For example, one cluster is most likely to be associated with threat-response behaviours because it contains most of the DNs and ANs known to have this function. Cells in this cluster are influenced by visual loom detectors, visual small-object detectors and mechanoreceptors (Fig. 3e). They output to endocrine neurons and to wing and leg motor neurons (Fig. 3f). This result is consistent with the idea that these DNs and ANs trigger evasive manoeuvres while also recruiting the energy stores needed to support these manoeuvres.

Another cluster is probably involved in reproductive behaviours. These cells are influenced by tactile sensors, taste sensors and nociceptors^51^ (Fig. 3e). They influence the uterus and reproductive tract as well as the neurohemal complexes, which release signals into the circulatory system (Fig. 3f).

Using a similar process of inference, we linked many clusters with putative behaviours—namely, walking, walking steering, flight steering, flight power, head orienting, grooming, threat response, postural control, visceral control, feeding and probing (Fig. 3d and Extended Data Fig. 7). The term ‘probing’ refers to tactile sampling before feeding initiation^52^. We propose that this behaviour is linked to the cluster that receives strong input from labellar tactile afferents and external taste sensors (Fig. 3e) and exerts influence over the forelegs, proboscis and pharynx (Fig. 3f). Meanwhile, we suggest that a distinct cluster is associated with feeding because it receives the highest influence from internal taste sensors (Fig. 3e). Moreover, it has strong influence over the pharynx, crop and salivary glands as well as endocrine neurons acting on the digestive tract (Fig. 3f).

The visceral control cluster contains ANs and DNs that seem to coordinate endocrine cells in different body parts (Fig. 3f). An example is AN27X017 (Fig. 3g). This AN relays signals from putative abdominal oxygen sensors (Y.L., manuscript in preparation) directly to brain endocrine cells that release insulin-like peptides (DILPs), which regulate feeding^53^. In the brain, these ANs converge with projections of aorta sensory neurons^54,55^. AN27X017 also synapses onto DNp65, which targets abdominal ganglion leucokinin neurons that regulate feeding and diuresis^56^. This circuit might regulate energy and water balance during physical stress.

Some DNs and ANs seem to have multiple functions. Consider DNg27 (Fig. 3h): this DN synapses onto wing power motor neurons and brain endocrine neurons that release corazonin, which mobilizes energy stores^57^. Therefore, DNg27 is positioned to increase flight power while also releasing energy needed to sustain flight. DNg27 is postsynaptic to thirst-sensitive interoceptive neurons in the brain^58^, and this connection may help control flight power based on water balance. This is because high flight power involves high metabolic demand and thus water loss via respiration^59^. Meanwhile, the same corazonin neurons are positioned to receive nociceptor signals from ANXXX139. This AN may respond to painful stimuli by recruiting energy reserves to prepare for escape.

ANs and DNs can form extended loops. For instance, sex peptide abdominal ganglion (SAG) ANs (ANXXX983)^15^ are downstream from sensory neurons in the uterus, oviduct and spermatheca (Fig. 3i), which is consistent with their known role as monitors of the reproductive tract^60^. SAG ANs signal to pC1 cells in the female brain^61^, which lie upstream from several DNs in the female reproduction cluster, including oviDNa_a and DNp37. DNp37 influences uterine motor neurons, whereas oviDNa_a influences ascending sensory signals from the uterus via interposed ANs (Fig. 3i). Together, these cells form an extended feedback loop that links uterus sensory signals with uterus motor neurons.

In addition to finding many behaviour-centric clusters, we found four sensory-centric clusters (tactile, proprioceptive, vibratory and taste–touch; Fig. 3e). These clusters may be involved in whole-body integration of sensory cues. For example, DNge104 is downstream from tactile sensors across the body (Fig. 3j) but also upstream from tactile sensors from these same body parts. Because DNge104 is inhibitory, this circuit could produce tactile contrast enhancement. For example, touching the head or thorax is predicted to excite AN09B023, which then excites DNge104, thereby decreasing tactile input to the rest of the body. In a feedback control system, modulation of a sensor feedback signal can cause the system to operate with a different effective set point^62–64^.

Even in the behaviour-centric clusters, we found DNs and ANs positioned to influence sensory processing. For example, AN09B011 in the walking cluster (Fig. 3k) makes a strong direct connection onto a visual centrifugal neuron (mALC5), which is positioned to suppress neurons with ventral visual fields, including visual optic flow detectors and loom detectors. This AN is postsynaptic to leg proprioceptors; therefore, it might relay leg movement information to mALC5, enabling this circuit to suppress visual responses to leg movement^65^ or anticipated ventral optic flow from walking.

In summary, we were able to assign a tentative behavioural or sensory role to all DNs and ANs on the basis of their connectivity. Our results suggest that DNs and ANs are conceptually analogous to the long-range connections that coordinate behaviour-centric control modules in robotic design^13,14^. Behaviour-centric control modules can be useful because they reduce the need for centralized planning and coordination. Complex behaviours can emerge from these modules even without centralized supervision because these modules essentially supervise each other.

## AN/DN specialization and coordination

**Fig. 4:**
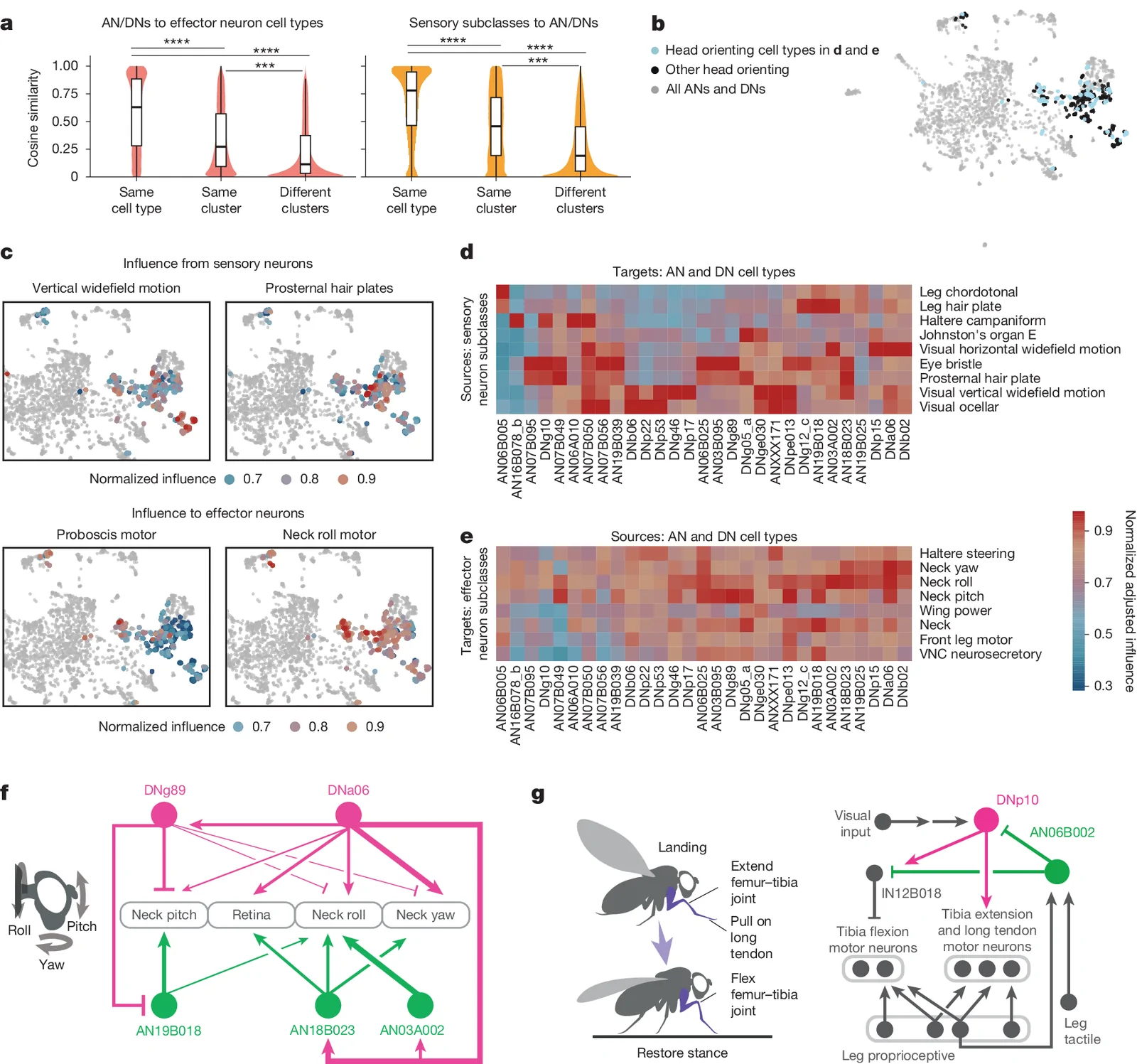
Specializations and coordination within a functional cluster. **a**, Cosine similarity of raw influence scores for ANs and DNs of the same cell type, same cluster or different clusters. We compared outgoing effector neuron influences and incoming sensory neuron influences for three different groups (pairwise two-sided Wilcoxon rank-sum with Holm correction), all *P* < 2.2 × 10^–16^; *n* = 4,947,085 sensory→AN/DN pairs, *n* = 4,054,128 AN/DN→effector pairs. Box plots: centre line, median; box, 25th–75th percentile (IQR); whiskers, 1.5× IQR; outliers not shown. **b**, PCA–UMAP embedding of ANs and DNs (Fig. 3d) with the head-orienting cluster and cell types from **d** and **e** highlighted (https://ng.banc.community/2026a/head-orienting). **c**, Top, neurons coloured by their incoming normalized (see below) adjusted influence from two example sensory sources. Bottom, neurons coloured by their outgoing normalized adjusted influence onto two example effector neuron groups. Values were min-max normalized (scaled to a bounded [0,1] range) across all ANs/DNs. **d**, Normalized adjusted influence from sensory neurons for selected cell types in the head-orienting cluster. Sensory neuron subclasses with adjusted influence over the 95th percentile across all sensory neuron subclasses are included. Values were min-max normalized across all sensory cell classes across the entire dataset onto the selected AN and DN cell types. **e**, Normalized adjusted influence onto effector neurons for these same ANs and DNs. Effector neuron subclasses with adjusted influence from AN and DN types over the 99th percentile across all effector neuron subclasses are included. Values were min-max normalized across AN and DN cell types in the entire dataset onto the selected effectors. **f**, Example circuit with neurons in the head-orienting cluster. Thick arrows indicate connections with >100 synapses; intermediate arrows indicate connections with 20–100 synapses; thin arrows indicate connections with 5–20 synapses. Note diverse but overlapping connectivity, and hierarchical interactions, within a cluster (https://ng.banc.community/2026a/figure-4f). **g**, Example circuit with neurons in the postural control cluster (DNp10 and AN06B002). This example shows that ANs and DNs in the same cluster can be organized into loops (https://ng.banc.community/2026a/figure-4g). Codex links for this figure are listed in the Supplementary Information.

Overall, DNs and ANs in different clusters have different influences, whereas cells in the same cluster have similar influences (Fig. 4a and Extended Data Fig. 8b). Nonetheless, cells in the same cluster are still diverse. For example, different ANs and DNs in the head-orienting cluster are influenced by distinct visual or mechanosensory signals, and they influence different combinations of motor neurons (Fig. 4b–e).

Moreover, some DNs and ANs are positioned to recruit (or suppress) many other cells in their home cluster^50^. Again, the head-orienting cluster provides examples of this. For instance, DNa06 is an excitatory DN with connections onto eye motor neurons as well as neck motor neurons that control all three axes of movement (roll, pitch and yaw; Fig. 4f). DNa06 also targets two ANs that are positioned to recruit either neck or eye motor neurons (or both). Meanwhile, DNa06 targets DNg89, which is positioned to inhibit neck-pitch neurons directly and indirectly through AN19B018 (Fig. 4f).

Within a cluster, specialized ANs and DNs can also be organized into feedback loops. For instance, DNp10 drives landing manoeuvres in response to looming visual stimuli^66^, and we found that this cell is positioned to excite tibial extensor motor neurons and inhibit tibial flexor motor neurons (Fig. 4g), implying that it drives tibia extension during landing. Meanwhile, AN06B002 is positioned to inhibit DNp10, thereby arresting tibia extension. AN06B002 is postsynaptic to leg sensory neurons (Fig. 4g); therefore, this circuit could arrest tibia extension when the leg has made contact with a surface during landing.

In summary, cells in the same cluster can have specialized connections to sensors and effectors. Often, these related cells are interconnected, sometimes in loops. These circuits of finely specialized cells should enable flexible behavioural control that can be rapidly fine-tuned to the current state of the body and the environment.

## Interactions among behaviour modules

**Fig. 5:**
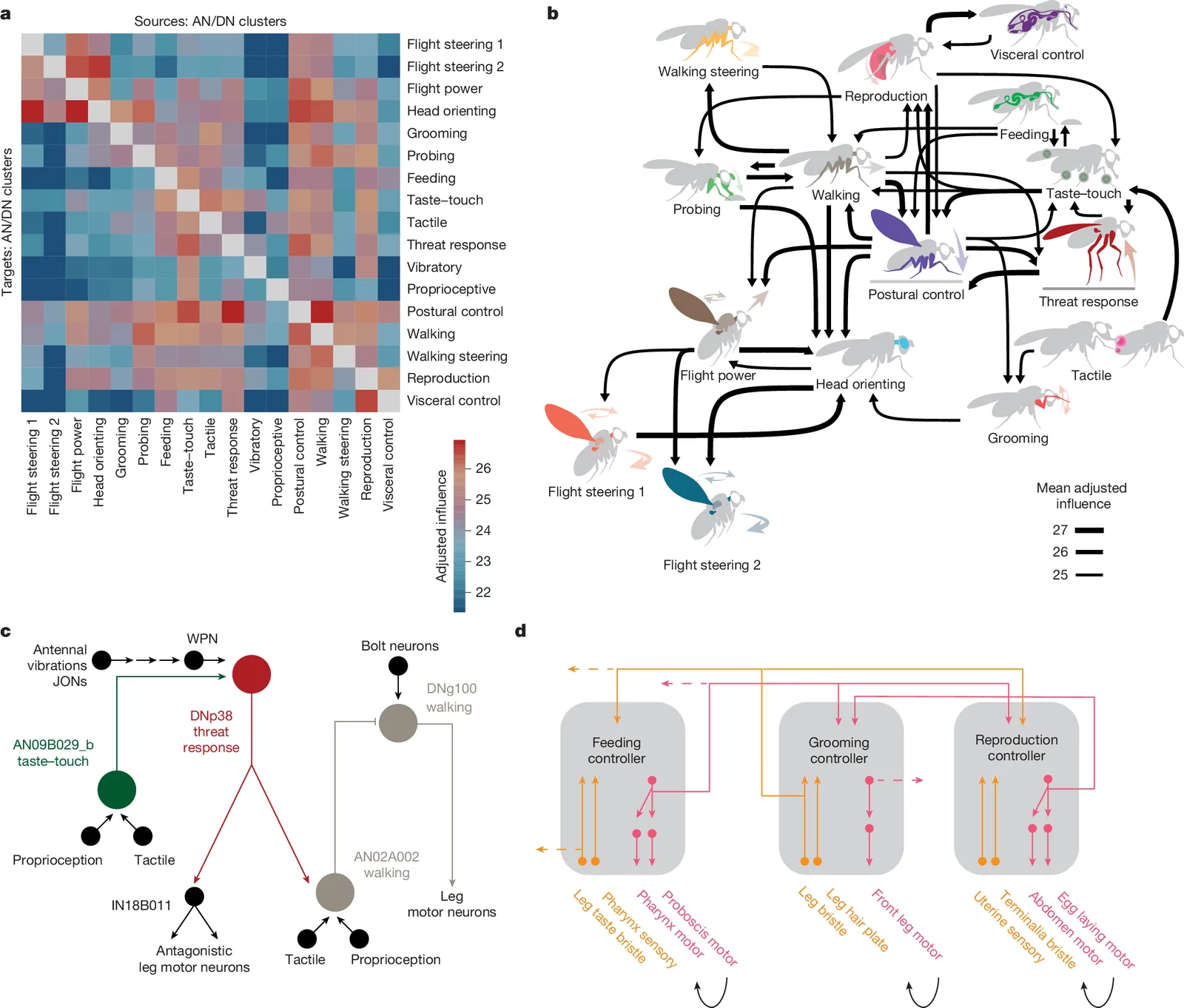
Interactions between behaviour-centric modules. **a**, Adjusted influence of each AN/DN cluster onto every other cluster demonstrated significant source × target interactions (*P* < 1 × 10^–15^, two-way ANOVA (type III), *n* = 9.5 million). Here, adjusted influence is source size corrected to control for cluster size (equation (10)). **b**, Summary of the strongest adjusted influences between clusters from **a**. For clarity, we depict only those influences with an adjusted influence ≥85th percentile; note that **a** shows all interactions, not just the strongest. Line weights are binned by adjusted influences in **a**. **c**, Example circuit illustrating cross-cluster interactions between ANs and DNs. This circuit links neurons in the proprioceptive, threat-response and walking clusters. Note the role of VNC interneurons in recruiting leg motor neurons. Wedge projection neurons (WPNs) here include WED092c, WED092d and LHPV6q1 (https://ng.banc.community/2026a/figure-5c). **d**, Our data suggest an architecture in which behavioural modules are linked by selective long-range pathways, creating hierarchical connections among behaviours. Through these connections, one module may recruit or suppress another (subsumption^13^). We schematize three conjectured behavioural modules, each constructed primarily from local sensory-motor loops; not all relevant sensors and effectors are shown. Black arrows, self-generated sensory feedback (reafference). Dashed lines denote connections to other modules not shown.

**Extended Data Fig. 9:**
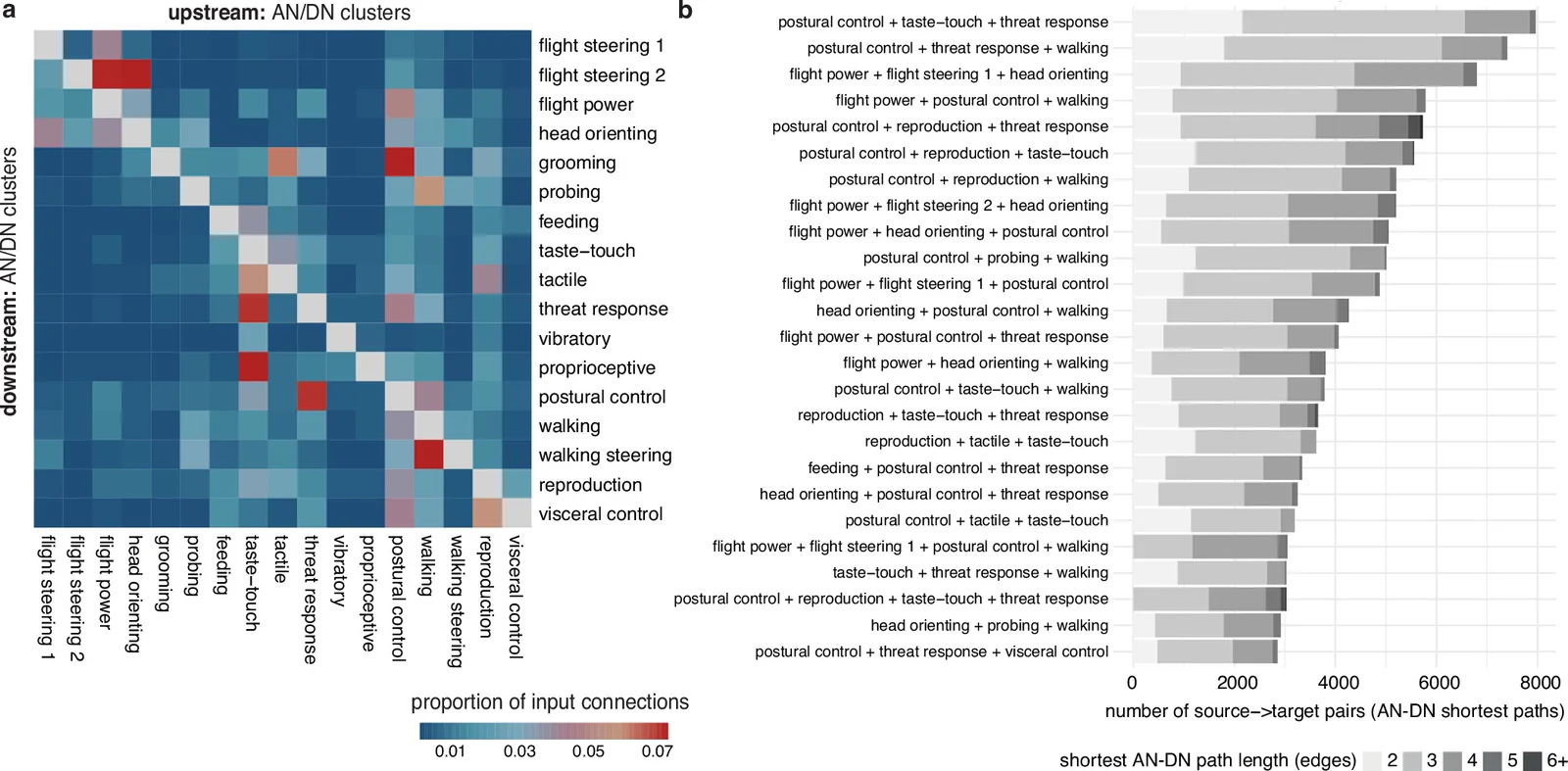
Interactions between AN/DN clusters. **a**, Direct connections between AN/DN clusters, shown as the proportion of each cluster’s total input supplied by the upstream cluster. **b**, The number of shortest paths between ANs/DNs in an AN–DN only network (i.e., the BANC synaptic connectivity edgelist limited to only ANs and DNs, threshold ≥ 10 synapses) that are between ≥ 3 clusters. Bars show the number of source→target cell type pairs whose shortest path traverses ≥ 3 clusters. Each bar corresponds to a unique cluster combination; paths are deduplicated and simplified (no node repeats). The 25 most common ≥ 3 cluster transits are shown. These short-path connections linking ≥ 3 clusters are akin to motifs reported in the larval *Drosophila* CNS^41^. Note that there are many two-hop paths connecting three different clusters. The vignette in Fig. 5c is one such example, from the second most common motif here.

In robotic design, interactions among behaviour-centric modules can prioritize behaviours, resolve conflicts and link behaviours into sequences^13,14^. The BANC dataset reveals a specific pattern of influence among behaviour-centric modules (AN/DN clusters; Fig. 5a). Focusing on the strongest of these influences, we can begin to reconstruct the logic of complex behavioural control (Fig. 5b and Extended Data Fig. 9).

For example, the walking cluster and the threat-response cluster strongly influence the postural control cluster (Fig. 5a,b). This observation is consistent with the idea that postural control should be subsumed by the need to walk or escape. Similarly, flight steering and head orienting strongly influence each other (Fig. 5a,b), reflecting the close coupling between head orientation and flight control^67,68^. Finally, reproduction strongly influences visceral control, consistent with the notion that multiple internal organs are primed to serve reproductive drives^69,70^.

Strong cross-cluster influences (Fig. 5b) are due to strong direct or indirect interactions between distinct groups of ANs/DNs. Consider the circuit (Fig. 5c) that involves cells from the taste–touch cluster (AN09B029_b), the threat-response cluster (DNp38) and the walking cluster. AN09B029_b sends mechanosensory signals to DNp38, which also receives antennal mechanosensory signals (via wedge projection neurons^71^). DNp38 is positioned to drive co-contraction of antagonistic muscles in all the legs, which should increase leg stiffness. Thus, this circuit motif might integrate whole-body mechanosensory signals to stabilize a defensive posture. Meanwhile, DNp38 is also positioned to recruit AN02A002, which inhibits DNg100, a cell downstream from pro-walking Bolt neurons^72^, which initiates walking^73^. In this manner, a mechanical threat could stabilize the resting stance while also suppressing walking.

Overall, the arrangement of influence between clusters (Fig. 5b and Extended Data Fig. 9) is analogous to subsumption architecture in robotics (Fig. 5d). In subsumption architecture, some behaviour-centric modules are positioned to influence (subsume) another module to exploit its functionality or override it^13,14^. A set of semi-autonomous modules, loosely linked in a subsumption hierarchy, can produce complex, emergent behaviours^14^.

## From behaviour modules to CNS networks

**Fig. 6:**
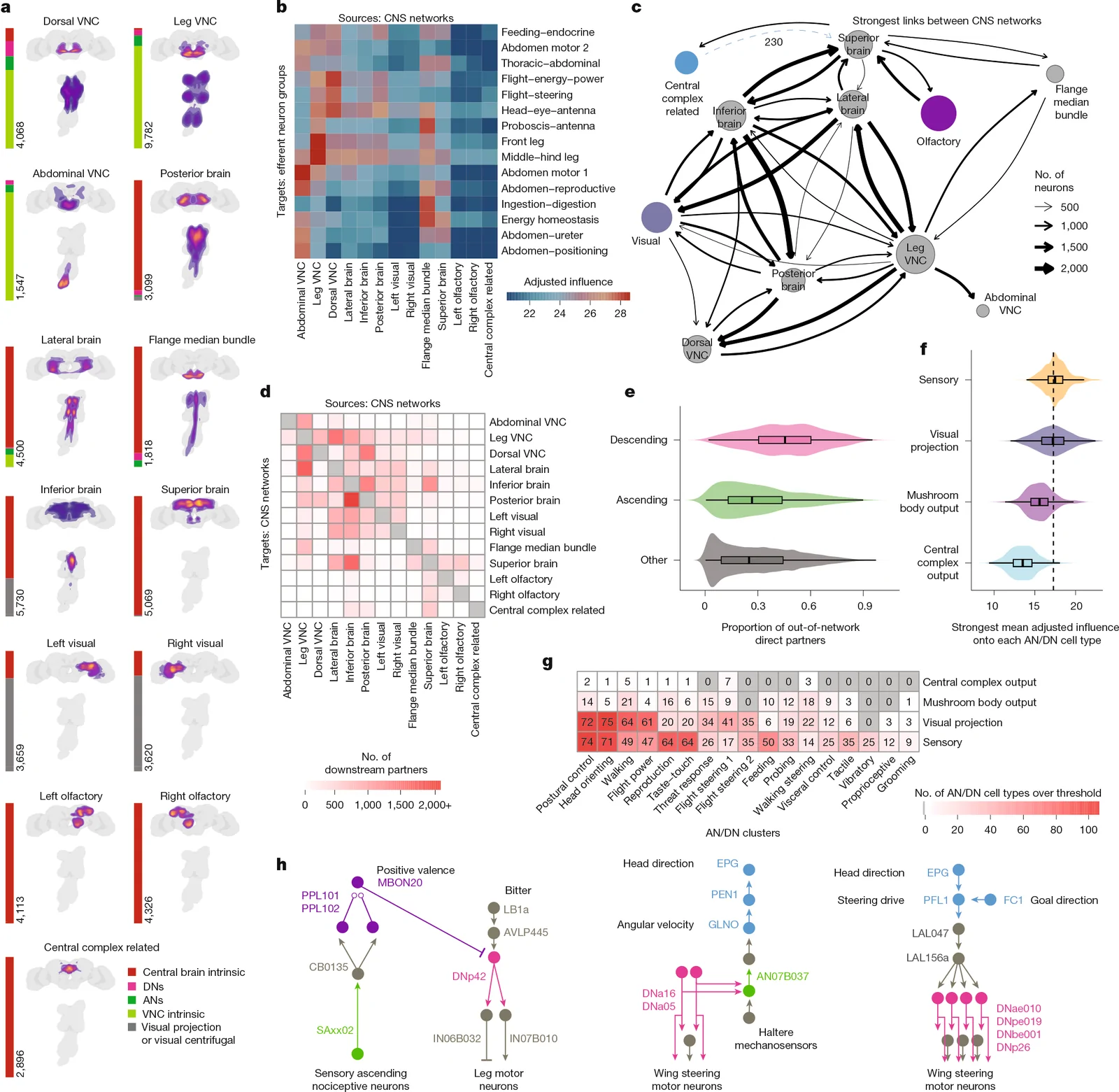
Linking CNS networks with AN and DN clusters. **a**, CNS networks from spectral clustering of 50,568 proofread neurons (excluding sensory, effector and optic lobe intrinsic neurons; neurons with low synapse counts were dropped). Each panel shows 2D kernel density estimates of synapse density (100,000 random synapses per network), inventory by superclass. Numbers are neurons in each network. **b**, Adjusted influence of each network on each effector group (Extended Data Fig. 4f). Source × target interaction was significant (*P* < 2.2 × 10^–16^, two-way ANOVA type III sum of squares, *n* = 522,148 observations over 528 sources × 15 effector groups). Overall, 10 out of 13 networks have greater than median influence on ≥1 effector group; the central complex related network and olfactory networks have greater than the median influence on no effector groups (combined, *P* = 9.3 × 10^–10^; two-sided binomial test, *P* = 0.019). **c**, Strongest connections between CNS networks. The size of each arrow represents the number of source neurons that connect with the target network (≥5 synapses), thresholded at 500 (except we show a below-threshold outgoing link from the central complex related network to show its strongest output). **d**, Matrix underlying **c**. The colour scale is capped at 2,000 neurons. Within-network connections are omitted (grey). **e**, Out-of-network connections, measured as the proportion of direct partners (upstream or downstream with ≥5 synapses) each neuron has in another network. ANs and DNs had significantly more out-of-network partners compared with other neurons in their respective CNS networks (DNs, *P* < 2.2 × 10^–16^; ANs, *P* = 3.53 × 10^–10^; two-sided Kolmogorov–Smirnov test, Holm-corrected; *n*_total_ = 50,568, *n*_DN_ = 1,239, *n*_AN_ = 1,810 and *n*_other_ = 47,519). Box plots: centre line, median; box, 25th–75th percentile (IQR); whiskers, 1.5× IQR; outliers not shown. **f**, Distribution of adjusted influence from four cell classes onto each of the 1,000 AN/DN cell types. Dashed line shows the adjusted influence threshold (17.28), as in Extended Data Fig. 5, which was also used to threshold cell-type selection in **g**. Visual projection, mushroom body output and central complex output cell types differ from sensory (visual projection, *P* = 3.54 × 10^–4^; mushroom body output and central complex output, *P* < 2.2 × 10^–16^; two-sample two-sided Kolmogorov–Smirnov tests versus sensory, Holm-corrected). Box plots: as in **e**. **g**, Counts for AN/DN cell types within each cluster above threshold (**f**) for each upstream cell class. **h**, Left, example circuit connecting mushroom-body neurons (purple) to ANs and DNs (https://ng.banc.community/2026a/figure-6h-mb). Middle and right, example circuits connecting central complex neurons (blue) to ANs and DNs (https://ng.banc.community/2026a/figure-6h-cx).

**Extended Data Fig. 10:**
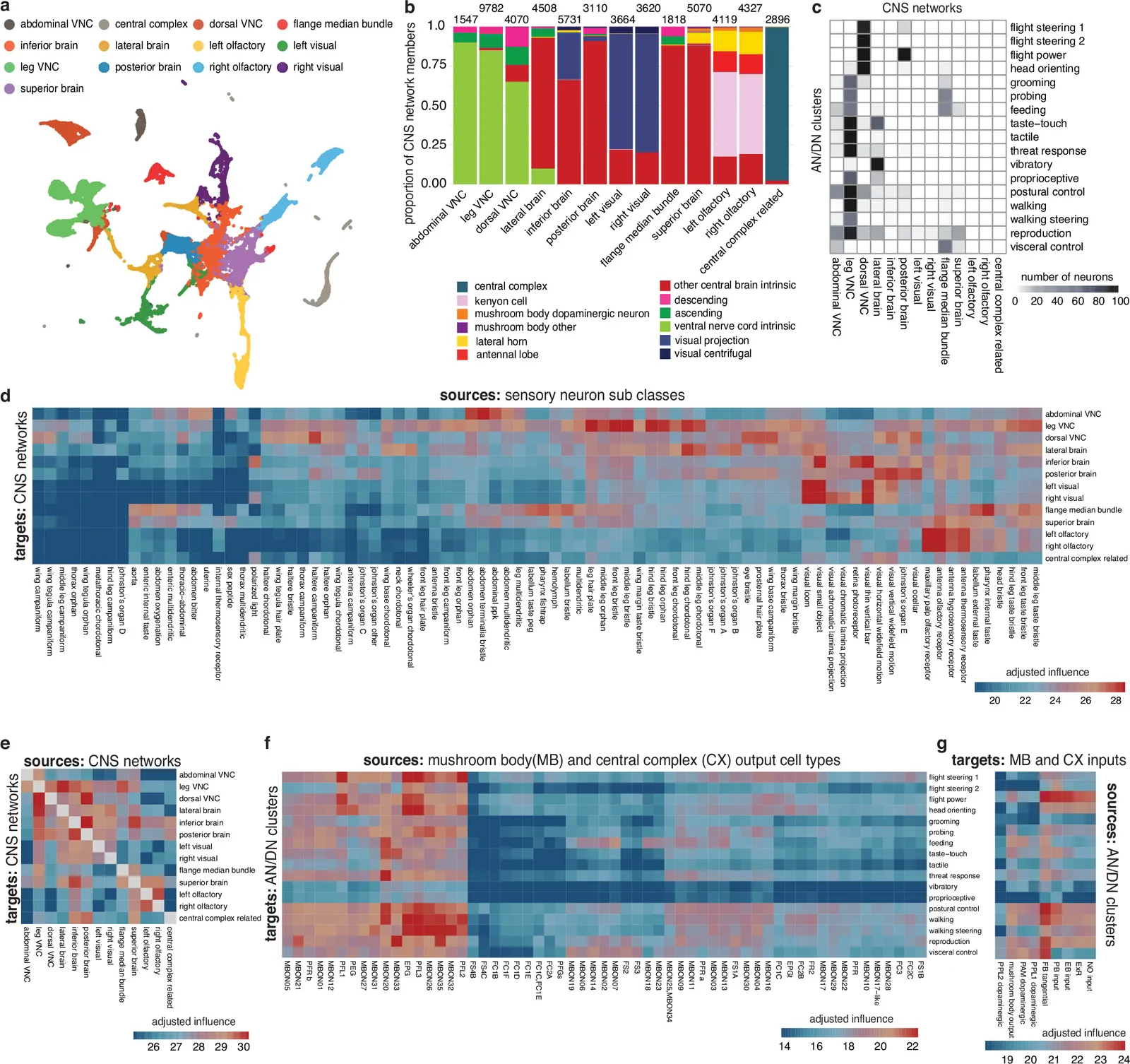
CNS networks’ cluster influence from sensors and to effectors. **a**, A PCA-UMAP embedding based on direct connectivity between BANC neurons (specifically, the eigenvectors of the graph Laplacian, derived from the undirected connectivity matrix), where each point is a neuron coloured by the CNS network it belongs to. This analysis uses all BANC neurons that meet the following criteria: they are proofread, they are not sensory or effector neurons or optic lobe intrinsic neurons, and they have > 100 incoming and outgoing connections. In total, 50,568 neurons were used for this analysis, corresponding to 100% of cell-typed central brain or VNC intrinsic neurons. **b**, Proportion of neurons of each CNS network belonging to select super classes/cell classes. Numbers above each bar indicate the total number of neurons in each CNS network. **c**, Number of ANs and DNs in each CNS network. ANs and DNs are grouped by cluster (Fig. 3d). **d**, Adjusted influence of sensory neuron sub classes onto CNS networks. Visual projection neuron cell types are also included, although they are not sensory neurons. **e**, Adjusted influence of each CNS network onto each other CNS network. **f**, Adjusted influence of mushroom body output neurons and central complex output neurons onto AN/DN clusters. **g**, Adjusted influence of AN/DN clusters onto input neurons of the mushroom body and central complex.

Finally, we asked how DNs and ANs are integrated with the rest of the brain and cord. We began by dividing the CNS into 13 networks, on the basis of the direct synaptic connections of each neuron, using a spectral clustering algorithm that maximizes within-network connectivity while minimizing across-network connectivity (Fig. 6a, Extended Data Fig. 10a and Supplementary Data 8). Our aim was to identify large groups of interconnected neurons, as these would be candidate functional divisions of the CNS.

Notably, many of these CNS networks span the brain and VNC (Fig. 6a), and many contain ANs and DNs (Extended Data Fig. 10b,c). Most CNS networks also have high influence on at least one group of effector neurons (Fig. 6b), and most networks receive strong sensory influence (Extended Data Fig. 10d). Together, these results suggest that behavioural control is distributed across CNS networks. The CNS networks with a high influence on effector neurons are directly linked in a dense pattern of reciprocal connectivity (Fig. 6c,d and Extended Data Fig. 10e). Notably, these links are disproportionately composed of DNs (Fig. 6e). Most AN/DN clusters are divided between two or three CNS networks (Extended Data Fig. 10c), consistent with the notion that DNs often form bridges between networks.

Interestingly, the central complex and the olfactory system emerged as networks with distinct properties. They have low influence on effector neurons (Fig. 6b), weak input from other networks (Fig. 6c,d) and low AN/DN membership (Extended Data Fig. 10b,c). Thus, these networks may ‘supervise’ actions. This supervision probably involves learning, as the olfactory network includes most of the mushroom body, which is a locus of associative learning^74^, and the central complex is the navigation centre of the brain and a locus of spatial learning^75^. Output neurons of the mushroom body and central complex exert high influence on only a small number of ANs and DNs (Fig. 6f). DNs and ANs with high influence from the mushroom body are mainly found in the walking, walking-steering and flight-steering clusters (Fig. 6g and Extended Data Fig. 10f). These results suggest that the central complex and mushroom body supervise specific actions rather than all actions.

Several example circuits illustrate how supervisory networks might communicate with lower networks via DNs and ANs (Fig. 6h and Extended Data Fig. 10f,g). For example, leg nociceptors (SAaxx02) project directly to the brain, where they are positioned to excite several mushroom-body dopamine neurons, including PPL101 and PPL102 (Fig. 6h). These dopamine neurons encode negative valence^76,77^, and they are positioned to instruct learning in several mushroom-body output neurons, including MBON20 (ref. ^6^). Given the synaptic rules that govern learning in the mushroom body, we anticipate that these dopamine neurons will ‘teach’ MBON20 to respond to odours that lack negative associations (that is, odours associated with safety). Notably, MBON20 is positioned to inhibit DNp42, a threat-response DN that drives backward walking in response to noxious stimuli^78^. Thus, odours associated with safety should excite MBON20, which is then positioned to suppress avoidance behaviour (Fig. 6h). This example illustrates how the olfactory network might supervise behaviour by interacting with ANs and DNs.

Another example supervisory circuit comes from the central complex. Here angular path integration is driven by an internal estimate of the fly’s rotational velocity, encoded by GLNO neurons^79^. The BANC dataset reveals that GLNO neurons receive a strong disynaptic excitatory input from a specific AN (Fig. 6h). This AN receives direct input from DNa16 and DNa05, which influence wing-steering motor neurons. Thus, this AN is positioned to send descending flight-steering signals back up to the central complex to update the head direction system in anticipation of an upcoming heading change. Meanwhile, the central complex continuously compares the fly’s estimated head direction against its internal goal. This comparison is performed by several cell types, including PFL1 (refs. ^7,80,81^), but the DN targets of PFL1 have not been fully identifiable until now. The BANC connectome shows that DNs downstream from PFL1 are likely flight-steering neurons (Fig. 6h). Thus, PFL1 is positioned to compare the fly’s head direction with its goal direction to generate corrective steering in flight when these directions are misaligned. Again, this example illustrates how the central complex can supervise behaviour by interacting with ANs and DNs.

## Discussion

The BANC connectome represents a substantial advance in scale compared with other connectomes (*Caenorhabditis elegans*^1,2^, *Ciona intestinalis*^4^, *Platynereis dumerilii*^3^ and *Mnemiopsis leidyi*^5^). Tackling a problem of this size required us to leverage new methods for semi-automated sectioning and transmission EM imaging, section alignment, cell segmentation, synapse identification, neurotransmitter assignment and cell-type matching. Because we could draw on the expertise of a large open-science community, we were able to assemble an embodied connectome with explicit connections to many organ systems.

An embodied connectome of this scale offers new clues about the logic of behavioural control. The classical theory is that actions are selected by a centralized executive brain function (sensing→cognition→action, the classical sandwich)^82^. The implementation of motor patterns could be delegated to circuits in the spinal cord or insect VNC^23,83,84^. However, in the classical view, these motor patterns are selected by descending commands from a centralized executive controller in the brain. Following this classical model, executive functions like action selection and attention have been ascribed to the insect central complex or mushroom body^20,24–26^. A radical alternative is the ecological theory, which dispenses with the middle layer of the classical sandwich. It posits direct bidirectional connections between sensing and action, thereby rejecting the need for internal representations^85^. More recently, theories of embodied cognition have argued that sensing↔action loops could be implemented by a modular, distributed control architecture that is tightly connected to the body, an idea that takes inspiration from robotic design^86^. Embodied cognition does not reject internal representations, but it argues that their role is limited. For example, local efference copy signals might be used to inform locomotor control, or memorized snapshots might be used to guide navigation. Models of embodied cognition predict that most behaviours engage distributed networks, consistent with recent data from mammals^87,88^.

Our analyses provide new insight into the implementation of distributed behavioural control. First, we found that the core elements of behavioural control are local feedback modules, in which effector neurons are influenced by the sensors positioned to monitor the relevant effectors. Local feedback loops have been identified in the vertebrate spinal cord and brainstem^89,90^, as well as the insect VNC^10,23,84,91–94^, gnathal ganglion^95^ and enteric nervous system^54^. Our study extends previous work to show that local loops are located throughout the CNS and that they are consistently the strongest influences on effector neurons. Second, our analyses draw attention to the ubiquitous recurrent connections between effector pathways, which again are primarily local. These recurrent connections are positioned to alert the CNS to upcoming movements, thereby reducing processing delays and helping to coordinate different effectors. The prominence of local feedback loops and recurrence supports the embodied cognition model of neural function.

At the same time, purposeful behaviour also requires long-range coordination among body parts (long-range loops^45^), which is partially mediated by ANs and DNs. We found that these cells can be divided into clusters, with each cluster connecting a specific set of sensory and effector neurons. We linked many clusters with behaviours, and we highlighted hierarchical connections among clusters^15,50^. This control architecture—typified by behaviourally specialized hierarchical connections—is reminiscent of subsumption architecture in robotic design^13,14^, which helped motivate theories of embodied cognition^86^. In these robots, control is divided among many modules, each devoted to a behavioural task or aptitude, and high-level modules have the ability to recruit or suppress lower-level modules via hierarchical connections.

According to the classical theory, descending signals are merely action commands, which in principle might be packaged into a small number of descending axons^15,50,96^. However, we found that the number of DNs (1,316) is larger than the number of effector neurons (1,031), and ANs are even more numerous (1,849). Indeed, within each AN/DN cluster, we identified many fine-grained variations on the same connection pattern, forming parallel pathways with slightly different inputs and/or outputs. This arrangement should promote flexibility by offering many available action patterns. It should also promote precision by pre-selecting the specialized action patterns that can result from particular sensory signals. These specializations could explain why different threat-response DNs can produce different escape takeoff manoeuvres^97^ and why different steering DNs can produce distinct changes in leg movement^98^. Notably, DNs and ANs typically influence effector neurons indirectly, via interneurons, and the circuitry of these interneurons plays a key role in behavioural dynamics^99,100^.

Finally, considering the network structure of the entire brain and cord, we found that many CNS networks have a high influence on effector neurons, further supporting the idea that behavioural control is distributed. A few CNS networks have notably low influence on effector neurons; this is particularly true of the central complex and the olfactory network (which includes most of the mushroom body). We propose that the central complex and mushroom body have a supervisory role and that they supervise a subset of behaviours, not all behaviours (consistent with the fact that disruptions to these circuits have limited and specific effects on behaviour^101,102^). This idea is compatible with embodied cognition (which posits a limited role for memories and other internal representations). It is also compatible with subsumption architecture, whereby high-level modules supervise low-level modules, but high-level modules are nonessential for many behaviours^13,14^.

This project illustrates how insight can arise from new technologies combined with the accumulation of many small biological facts. Just as early cartographers amalgamated the work of multiple map-makers, we have deliberately amalgamated typology and metadata from previous *Drosophila* connectomes. We anticipate that future work will amalgamate the BANC dataset with additional datasets, including the recently released maleCNS connectome^29^. The BANC connectome has been an open-science project since 2023 and, as a living public dataset, it will progressively improve. This open-science effort should generate even more testable experimental hypotheses and, ultimately, new theories.

## Methods

### Overview

To produce the BANC dataset, we dissected a CNS sample from an adult female fly, stained and fixed the tissue, sectioned the sample and imaged sections at synapse-level resolution. We aligned the image volume into a 3D dataset, and Zetta.ai (https://zetta.ai/) performed automated image segmentation and synapse detection. SixEleven and Aelysia performed the bulk of the manual proofreading to fix automated segmentation errors and to identify sufficiently complete neurons. Proofreading and annotation infrastructure was based in Neuroglancer^103^ (proofreading and visualization) and CAVE^28^ (key metadata annotation) and managed together with Yikes. We built custom R and Python tools and code for data analysis and to manage and assign metadata. These tools were built on open-source projects, including Navis^104^ (Python) and the Natverse^105^ (R). We used established frameworks for metadata assignments where possible, and a team of two to four experts manually reviewed all metadata. The wider research community was openly engaged to improve the BANC dataset, mainly via CAVE^28^ and FlyWire^27^. At the time of publication, the BANC is a living dataset: FlyWire Codex (https://codex.flywire.ai/banc) provides user-friendly access and reflects continued improvements to the dataset. We also share our data via the Harvard Dataverse (10.7910/DVN/7WTH1N, for static long-term storage) and a public Google Storage Bucket (console.cloud.google.com/storage/browser/lee-lab_brain-and-nerve-cord-fly-connectome/, for easier access and updates). Below, we call out specific data files at these two locations in square brackets. Thus, for example, [banc_888_meta.feather] refers to a file with this name in both the Harvard Dataverse and the Google Storage Bucket.

### Specimen

All animal work used the fruit fly *D. melanogaster*, an invertebrate species not subject to institutional animal care regulations. The BANC sample came from a female adult fly. To choose this specimen, we behaviourally screened 5–6-day post-eclosion wild-type *D. melanogaster* female flies (F_1_ progeny of a *w*^*1118*^×Canton-S cross)^106,107^. The fly used for the BANC dataset turned right 70% of the time over 582 choices when walking in an acrylic Y-maze [behavior.zip] for 2 h, placing the fly at the 97th percentile for handedness in the population (*n* = 1,095). We raised the flies on standard cornmeal–dextrose medium at room temperature (around 20 °C) in natural lighting conditions. We collected flies on the day after eclosion, housed them in vials with other flies for 4–5 days, behaviourally tested them and then subsequently housed them individually in vials for about 1 day at 25 °C until dissection.

To dissect the flies, we pinned them individually onto a dissection pad then submerged them in a drop of ice-cold Karnovsky’s fixative (2.5% formaldehyde and 2.5% glutaraldehyde in 0.1 M cacodylate buffer, pH 7.4) containing 0.04% CaCl_2_. We removed the legs and proboscis to allow fixative to access the nervous tissue. Next, we carefully removed the head capsule and the cuticle of the ventral thorax to expose the nervous tissue for dissection. Within 5 min, we completely dissected the brain and connected VNC, and we transferred these to an Eppendorf tube containing the same Karnovsky’s fixative. We fixed the sample at 4 °C overnight. On the subsequent day, we washed the sample with 0.02 M 3-amino-1,2,4-triazole (A-TRA) in cacodylate buffer (3 × 10 min) and then we stained it with 1% OsO_4_ in 0.1 M A-TRA for 90 min on ice. On the same day, we stained the sample with 1% thiocarbohydrazide for 8 min at 40 °C, 2% OsO_4_ (aqueous) at room temperature for 60 min, and 1% uranyl acetate in maleate buffer at 4 °C overnight. On the next day, the sample was stained with lead aspartate for 3 h at 60 °C, then dehydrated in a graded ethanol series, washed with propylene oxide and infiltrated with 2:1 then 1:2 propylene oxide:LX-112 resin, consecutively for 30 min each. The sample was then placed in pure LX-112 resin overnight at 4 °C and was embedded in fresh pure resin the following day and cured at 60 °C for 48 h.

The resin-embedded sample was scanned on a microCT X-ray scanner (Zeiss) before serial sectioning [banc_microCT.zip] to screen for obvious defects or damage, and to check that the neck connective appeared intact. Bounding boxes of known artefacts that affect reconstruction are provided in Supplementary Data 10. The specimen includes the central brain, neck connective and VNC, as well as the medulla, lobula and lobula plate of the optic lobes. It lacks the lamina (part of the optic lobes), the ocelli and the ocellar ganglion. Thus, the R1–R6 photoreceptors, the lamina intrinsic cell type Lai and the ocellar photoreceptors are missing from the BANC dataset (about 9,390 cells), and intrinsic neurons that arborize in the lamina and ocellar ganglion are incomplete (cell types L1, L2, L3, L4, L5, T1, C2, C3, Lat, Lawf1, Lawf2, OCG01, OCG02, OCC01, OCC02, DNp28 and the ocellar local neurons). There was also dissection damage to both the left and right antennal nerves, which resulted in damage to Johnston’s organ sensory neurons and central brain intrinsic neurons that pass near the nerve entry point, which means that their recovery and cell typing are suboptimal. These defects (of the lamina, ocellar ganglion and quality of Johnston’s organ neurons) are also true of the maleCNS project, a connectome of the whole brain and VNC of a male fly^29^. However, the Johnston’s organ sensory neurons in the BANC dataset are more complete. To our knowledge, the BANC dataset is the only available connectome dataset for which the full female abdominal neuromere is present.

### Serial sectioning

We cut serial 45–50-nm-thin sections and collected them on a 7500-slot reel of GridTape (Luxel) as previously described^8^.

### Transmission EM imaging

To acquire the dataset, we used a transmission electron microscope (JEOL 1200 EX) custom-modified with a custom vacuum extension and scintillator (Grant Scientific), a 2 × 2 array of sCMOS cameras (Andor, Zyla 4.2) and a reel-to-reel GridTape imaging stage, as previously described^8^. Imaging spanned 7.5 calendar months, but 96.5% of the images were acquired during the 4 months of November 2021 to February 2022.

### Missing data

Of the 7,010 sections, 6,970 (99.43%) were collected and imaged without data loss. Ten (0.14%) have no data owing to the section being lost (sections 856, 885, 3755, 5746, 5772, 5778, 5793, 5801, 5822 and 5869). Notably none of the losses are consecutive serial sections [banc_problem_regions.csv]. One of these losses (3755) was because the section was collected onto the wrong location on the GridTape (not over the slot) and so it could not be imaged by transmission EM. The other nine losses were due to the support film rupturing after section collection but before the section could be imaged. An additional 30 sections (0.43%) have partial data: 11 sections are missing all images for the brain (914, 1462, 5841, 5849, 5888, 5896, 5916, 6207, 6208, 6209 and 6210); 7 sections are missing all images for the VNC (874, 2784, 2822, 3064, 3102, 4566 and 5840); 12 sections are missing a fraction of the brain and/or VNC images (2828, 2860, 2912, 2986, 3054, 3080, 3586, 3605, 3833, 4648, 4768 and 5935). The large majority of these losses were also caused by partial rupturing of the support film before the tissue was imaged.

### Transmission EM dataset alignment and segmentation

We performed initial BANC image alignment with a custom software pipeline that deployed AlignTK alignment functions^108^ on a computing cluster^8^. We refined the alignment of the data using self-supervised CNNs and online optimization to produce displacement fields that were combined with a global relaxation^109,110^. We trained a CNN to identify regions that were damaged during serial sectioning. We used CNNs to segment the data into cells at 16 × 16 × 45 nm^3^ per voxel resolution, nuclei at 64 × 64 × 45 nm^3^ and mitochondria at 16 × 16 × 45 nm^3^, excluding regions that decreased segmentation performance (for example, excessive missing data or damage)^9,111^. We then ingested the automated cell segmentation into CAVE^28^ for collaborative proofreading.

### Nucleus and mitochondria detection

We used a CNN^9,111^ to automatically detect 153,493 nuclei. We filtered out predicted nuclei with a size less than 4 µm^3^ or greater than 250 µm^3^. We removed additional false-positive nucleus detections via manual proofreading. The list of the midpoints of these nuclei is available through CAVE as somas_v1a and somas_v1b, where somas_v1a is the full list and somas_v1b provides corrections to the midpoints of 651 nuclei [somas_v1.parquet]. A CNN^9,35,111^ trained on manual and semi-automatic labels collected from FAFB and BANC automatically detected 38,928,244 mitochondria.

### Synapse detection

We generated synapses in two steps: (1) postsynaptic terminal detection^112^ and (2) synaptic partner assignment^35^. We pre-trained both models with data from FAFB, and we tuned the detection model with an additional 721 (about 21% new) labels from the BANC dataset. The detection operated on 8 × 8 × 45 nm^3^ images, with an output at 16 × 16 × 45 nm^3^. We removed detection objects that were <3 voxels. Assignment operated at 16 × 16 × 45 nm^3^. We merged terminals with identical assignments that were within 200 nm of each other into a single terminal. Detected synapses are tabulated in the file synapses_v2, available through CAVE [banc_888_synapses_v2_enriched.parquet, banc_nt_prediction_w_sizethresh_5_11102025.parquet, synapse_neuropil_lookup_v2.parquet]. This file lists 218,460,852 synaptic links (pre-post connections), of which 74% of presynaptic ends and 23% of postsynaptic ends are connected to a proofread neuron. This is termed the synaptic ‘completeness’ of our reconstructions, that is, the completeness percentage^16^. Overall, 18% of synaptic links have an identified neuron on both sides. For other densely reconstructed datasets, this number is 41.9% for FAFB, 35.3% for hemibrain, 44.4% for MANC and 40.1% for connections within the neuropils of maleCNS. By calculating axon–dendrite splits^105,113^ among proofread neurons, we estimate that of the synaptic links in the BANC dataset, 48.1% are axo-dendritic, 19.3% are axo-axonic and 24.1% are dendro-dendritic. Note that a synapses_v3 with 259,409,001 synaptic links is available for users, which was made with more BANC-centric training data rather than using training data from FAFB, and is reported in ref. ^31^ [banc_888_synapses_v3_enriched.parquet, banc_nt_prediction_v3_w_sizethresh_10_05042026.parquet, synapse_neuropil_lookup_v3.parquet].

### Synapse detection evaluation

To determine the false-positive and false-negative rates of automatic synapse detection, we selected 16 cutouts of size 2 × 2 × 0.7 μm^3^ from across the dataset (in the medulla, lateral accessory lobe, superior posterior slope, gnathal ganglia, fan-shaped body, superior medial protocerebrum, mushroom-body medial lobe, mushroom-body calyx, anterior ventrolateral protocerebrum, lobula, T1 leg neuromere, wing tectulum, lower tectulum, haltere tectulum and an anterior and a posterior region of the abdominal neuromere) and we compared the predicted synapses with those manually identified by experts (Extended Data Fig. 2b). Specifically, experts identified all synaptic links within each cutout blind to the synapse prediction; two individuals checked each synaptic link. We then compared these manual detections with the predictions to identify true synapses, false positives and false negatives. Synaptic links for which either the presynaptic or the postsynaptic prediction was incorrect were considered errors. Finally, the experts examined the synapses in each category and corrected any errors or ambiguous synapses. We report the number of true, false-positive and false-negative synapses as well as the *F*-score, precision and recall for the individual cutouts and for the dataset as a whole (using the total number of synapses in each category summed across all 16 cutouts). Because our synapse detection method relies on identifying postsynaptic profiles, we may underdetect some classes of synaptic connection for which postsynaptic sites are less distinct. In particular, the average number of outgoing connections for Kenyon cells (36.6 synaptic links) is substantially smaller than in FAFB (around 200 synaptic links, cleft score threshold >50). Axo-axonic connections between peripheral sensory neurons may also be underdetected. Detection in the BANC dataset has an autapse rate of 2.1%, a majority of which we expect to be a misassignment of the presynaptic link from a correctly detected postsynaptic link. We recommend that users filter out autapses in their analyses. In addition to this evaluation on a small number of cubes subjected to dense manual review, we sparsely evaluated a sample of 4,648 synapses across the volume at different postsynapse sizes (number of voxels in the detected postsynapse) [2024-09-20_aelysia_synapse_sample_complete.csv]. Sampling around 7.1 postsynapses per neuropil + size bin, we noted a sharp drop in quality at size 5. Human review produced only 9.88% synapses under postsynapse size 5 as correct (75.9% false, 14.2% ambiguous). Above size 5, 89.3% were correct (9.55% false, 1.14% ambiguous). We therefore used a postsynapse size threshold of ≥5 for including a pre-post synaptic link when creating our neuron–neuron edgelists and for running our pre-synapse transmitter predictions. Previous projects^36^ have made use of a cleft score for quality control, but our synapse detection method does not provide a synaptic cleft size estimation.

### Neurotransmitter prediction

We used a recently described approach to predict the type of neurotransmitter released at each automatically predicted synapse^36^. In brief, we trained a 3D CNN to classify presynapses into one of eight neurotransmitter classes: acetylcholine, dopamine, GABA, glutamate, histamine, octopamine, serotonin or tyramine. We compiled ground-truth data for synaptic transmission from the literature^7,10,57,61,114–204^, totalling 3,379 identified cell types from FAFB, MANC and hemibrain (60,394 neurons). We removed motor neurons from the ground truth, as they have few presynapses within the CNS. The dataset was split by neuron into training and testing sets, with 80% of the data for training and 20% for testing. We used the following sampling strategy to ensure a balanced dataset across different neuron types. For neurons associated with the most common neurotransmitters (acetylcholine, GABA and glutamate), we randomly sampled a maximum of ten presynaptic sites from each neuron. For all other neurotransmitters, we included all identified presynaptic sites. This approach ensured that all cell types that had ground-truth were represented in both training and testing sets. The input data for the network consisted of 3D cutouts from the EM volume, each centred on a presynaptic site. These local cutouts had dimensions of 640 × 640 × 630 nm. We used a 3D CNN architecture based on the 18-layer residual network (ResNet-18)^205^. ResNet-18 includes 3D convolutional layers, batch normalization and ReLU activation functions, with the core of the architecture consisting of residual blocks that use skip connections to enable effective training. The model architecture was adapted for our task by modifying the initial convolutional block to accept single-channel greyscale input from EM data. Finally, we replaced the model’s original fully connected output layer with a linear layer that maps the learned features to our eight neurotransmitter classes, followed by a softmax activation to produce the final probability distribution. The network was trained using the Adam optimizer^206^ to minimize the focal loss function^207^. This loss function is a variant of the standard cross-entropy loss, which is effective for datasets with a substantial class imbalance as it downweights the loss assigned to well-classified samples, enabling the model to focus on difficult-to-classify samples. To further improve generalization of the model, we applied several data-augmentation techniques during training. These included random affine transformations, random noise and random gamma correction. The probability of applying these augmentations was increased for less frequent neurotransmitter classes to further mitigate class imbalance. We trained the model for 1,060,000 iterations using a batch size of 16 samples. The final model selected was the one that achieved the highest classification accuracy on the separate testing set. A neuron-level transmitter prediction [banc_888_neurotransmitter_prediction_v2.csv] was obtained by summing the classification probabilities for each predicted class across all presynaptic detections and selecting the class with the highest total confidence as the most likely neurotransmitter. Note that we assume that Dale’s law^208^ holds even though we know that some neurons in the CNS co-transmit with multiple fast-acting transmitters^36,128,130,209^. Serotonin predictions should be treated with caution because our CNN predicted that many peptidergic neurons are serotonergic (see also ref. ^36^). A fully cited compilation of ground-truth labels per cell type has been archived with Zenodo for neurotransmitters^210^ (10.5281/zenodo.20818141) and neuropeptides^211^ (10.5281/zenodo.20818143) [drosophila_neurotransmitters_archive.zip, drosophila_neuropeptides_archive.zip].

### BANC neuropil mesh generation

We built a closed CNS neuropil surface based on presynaptic sites. We sampled every 500th synapse from the BANC synapse table, and we converted the coordinates to nanometres. To suppress noise, we retained points only if they had ≥10 neighbours within 10 µm (*k*-NN radius filter). We then fit a 3D alpha-shape to the filtered cloud (*α* = 7.5 µm), converted it to a triangular mesh, decimated to around 25% of faces, Taubin-smoothed (10 iterations), cleaned degenerate and isolated facets and performed integrity checks. We split the resulting mesh at the neck to produce brain and VNC components. We made each component ‘integral’ (closed) by removing small isolated pieces and isotropically remeshing to fill small gaps; if regions contained multiple pieces, we merged them. We registered neuropil meshes defined in other work into the BANC space (see the section ‘Neuropils and template alignment’). We exported final surfaces as OBJ files [banc_neuropil_meshes.zip].

### Neuropils and template alignment

To transform the BANC data into a standard template space for analysis and inter-dataset comparisons, we computationally generated a virtual ‘neuropil stain’ based on predicted synapse locations^35^. To do this, we downsampled and Gaussian-blurred (*σ* = about 900 nm) the predicted synapse locations to produce a synapse density map at the approximate resolution of light microscopy data used in the *Drosophila* standard templates^212^. We then registered the synapse density map of the EM dataset to the JRC 2018 female brain and JRC 2018 female VNC templates^212^ [banc_template_spaces.zip] separately using elastix (https://elastix.lumc.nl/) [registration_brain_jrc2018f.zip, registration_vnc_jrc2018vncf.zip]. Leveraging this alignment, neuropils and neurons were transformed between different connectome datasets for visualization and quantitative comparison in the same coordinate system. Meshes for individual neuropils in the central brain^213^ and VNC^214^ were based on previous work. We generated a left–right registration for the BANC connectome based on a thinplate-spline warping registration built from matched points on identified pairs of around 30 DNs, available through the bancr R package (bancr::jrc2018f_to_banc_tpsreg, bancr::jrcvnc2018f_to_banc_tpsreg), enabling [banc_mirror_nblast.feather].

### Proofreading

We proofread neurons to correct automated cell segmentation errors as previously described^17^. This effort included academic neurobiologists, dedicated proofreading teams at Princeton, SixEleven and Aelysia, and a community of citizen scientists. We used a multipronged strategy. To capture neurons with cell bodies in the CNS, we proofread segments associated with automatically detected nuclei, which were then extended to reconstruct their full morphology and to remove false mergers. To include sensory neurons, whose cell bodies typically reside outside the CNS, we seeded every neuron profile [peripheral_nerves.parquet] in planes that cut a cross-section through a nerve (1 plane per nerve, except in cases when 1 plane could not capture the full cross-section of the nerve; 47 seed planes in total, corresponding to the nerves that are separable when they enter the CNS^213–215^) and then we performed reconstructions starting from those seeds. To capture all neurons in the neck connective, we seeded two planes that were cross-sections through the neck connective (*y* = 92,500 and *y* = 121,000) [neck_connective_y92500.parquet, neck_connective_y121000.parquet]. These transverse planes were positioned posterior to the central brain and anterior to the VNC. We also proofread orphan segments containing >100 presynaptic links in decreasing order of synapse count for the central brain and VNC. We considered a neuron ‘backbone proofread’ [backbone_proofread.parquet] when its primary neurites (if not sensory) or major microtubule-rich processes had undergone a thorough review^27^. In backbone proofread neurons, the overall morphology of the cell has been deemed to be correct, and we do not anticipate future proofreading to substantially alter the shape or identity of the neuron, although minor branches or a small number of synapses might require adjustment. Neurons were marked in CAVE from within the FlyWire Slack (https://codex.flywire.ai/faq) by a Slack Bot helper, ‘BANC bot’ [the-BANC-fly-connectome/slackbots/], to make adding these tags simple. We proofread 150,841 neurons to backbone proofread status. Moreover, we defined a further category, ‘roughly proofread’, to encompass neurons that were identifiable (comprising a large region of arbour) but may have some large omitted segments, which comprised 5,075 neurons [proofreading_notes.parquet]. Frequently, this was due to known dataset artefacts, that is, regions of the dataset where misalignment or data loss has made reconstruction more challenging. We provide these locations in Supplementary Data 10. Figure 1c includes roughly proofread neurons with nuclei and roughly proofread sensory neurons, so as not to count the same cell multiple times (that is, a roughly proofread orphan axon and its roughly proofread dendrite). In total, 155 people served as proofreaders for the project (defined as individuals who made ≥100 edits). Between our initial preprint^216^ and v.888, 62,773 proofread and roughly proofread neurons were updated, of which 42,270 are new additions. Note that some neurons are marked as proofread [banc_888_meta.feather] in excess of the strict v.888 snapshot [backbone_proofread.parquet], which were marked after the v.888 snapshot on 17 April 2026.

### Colour maximum intensity projections

We generated colour–depth maximum intensity projections (colourMIPs)^217^ of all proofread neurons using a BANC Python package (https://pypi.org/project/banc/). We also wrote an R equivalent (https://natverse.org/neuronbridger/). We registered neuronal reconstructions to JRC2018_Unisex_20x_HR (1,210 × 566 pixels) and/or JRC2018_VNC_Unisex_40x_DS (573 × 1119 pixels) for compatibility with NeuronBridge^37^ [banc_color_mips.zip].

### Cell-type matching and annotation

#### Overview

We follow previous work in our approach to defining cell types^218^. Previous studies have invested substantial effort in cell typing in both the brain^6,7,16,19,32^ and VNC^8,9,11^ using a combination of manual annotation and computational methods. Whenever possible, we aimed to re-use previously defined cell types. To achieve this, we developed a pipeline designed to match each of the around 160,000 neurons in the BANC dataset with an existing dataset by associating each neuron with a cell type in an existing dataset, namely FAFB-FlyWire (v.783)^17,29,219^ and/or MANC (v.1.2.1)^12^. Matching to maleCNS (v.0.9; mainly sensory neurons) and FANC (mainly sex-specific and dimorphic ANs and DNs) was also important to our workflow. Automatic matching was based on neuron morphology and position (using NBLAST^30^ after other connectomes were bridged into the BANC space; [banc_fafb_783_nblast.feather, banc_manc_v1.2.1_nblast.feather, banc_fanc_1116_nblast.feather, banc_hemibrain_v1.2.1_nblast.feather], see the section ‘Neuropils and template alignment’; Fig. 1c–e and Extended Data Fig. 2a) as well as connectivity (in the optic lobe, see below). Human annotators then manually reviewed cell-type matches. NBLAST alone is a key step in the matching process, but it is not sufficient to make accurate matches at a high rate; our manual review of all matched neurons revealed that 47% of the cell types differ from the type of the max-NBLAST match to MANC/FAFB (minimum normalized NBLAST score of 0.1). Specifically, in the VNC, cell-type assignments were more challenging because the reference dataset is of a male fly, and we had to account for sexual dimorphisms^31^. Nevertheless, we were able to assign cell-type labels [banc_888_meta.feather] to 94.4% of BANC neurons (147,846 neurons, 93.1% excluding the optic lobes), with an estimated error rate of about 7% based on sampling 1,000 matched neurons for more careful review, incorporating cosine similarity of their connectivity to compare neurons between datasets. To do this, connectivity vectors were collapsed by cross-matched cell types upstream or downstream of each query neuron, and then their similarity was calculated between all query neurons with a mid-to-high NBLAST score between BANC and FAFB/MANC. Most mismatches were due to incomplete proofreading. The mismatched neuron was almost always a similar cell type in the same hemilineage. For the remaining neurons that could not be confidently matched, we classified them on the basis of gross morphology and identified their closest associated neurons in other datasets with NBLAST. We estimate that around 10% of these unmatched neurons will prove unmatchable owing to reconstruction quality issues (in either the source or the target dataset) or developmental differences in neuron wiring. Although further work here is needed, we currently estimate that as many as about 2,316 neurons, or approximately 9%, of the female VNC may be sexually dimorphic (present in both sexes but morphologically different) and about 600 neurons, or approximately 2%, may be female-specific (present only in the female), and so cannot be matched well to MANC, which is a male VNC sample. These estimates span all VNC classes rather than intrinsic interneurons alone: of the 2,316 dimorphic neurons, there are: 1,439 intrinsic^31^, 359 ascending^15^, 430 sensory and 83 effector; of the 600 female-specific, there are 430 intrinsic^31^, 56 sensory, 38 ascending^15^ and 29 effector. For comparison, a previous study estimated that approximately 2.4%, of the female central brain is sexually dimorphic^29^.

Our annotation work enables direct comparisons with identified cell types in the field and facilitates integration with existing datasets. Excluding ANs and DNs (which are truncated in FAFB and MANC), the BANC dataset is missing 531 FAFB cell types (6.92%) and 171 MANC cell types (5.99%), but note that MANC is male and BANC is female, so some male cells have no female counterpart. Applying the same annotations to the male VNC, we estimate, on the basis of refs. ^15,31^, that as many as 763 MANC cells (approximately 3%) could be male-specific and 3,364 (approximately 14%) could be sexually dimorphic, which is greater than in the female VNC. To better build efferent and afferent types of the female abdominal neuromere (which, to our knowledge, has not been captured in any previous connectome dataset), we typed abdominal efferent and afferent neurons by cross-matching to the FAbG dataset, an in-progress connectome of the female abdominal neuromere (J.Z. et al., manuscript in preparation). We identified 13,000 bodies in the BANC dataset as potentially glia, trachea or cellular debris, and of these, we verified 413 as astrocytes. Note that the maleCNS project conducted a high-quality cell-type synthesis and re-typing to generate the maleCNS types, for which we suggest some cross-links and provide NBLAST results [banc_malecns_v0.9_nblast.feather]. We anticipate that future work will build on typologies between hemibrain, FAFB, MANC, FANC, BANC and maleCNS to produce a better consensus for the field. Note that additional annotations from the research community were added to CAVE by a Slack Bot helper, ‘BANC bot’ [the-BANC-fly-connectome/slackbots/], to make connectome annotation more accessible (https://codex.flywire.ai/faq).

#### Semi-automated cell typing by morphology with manual review

Using NBLAST^30^, which quantifies pairwise neuronal similarity by considering both the position and morphology of neuronal arbours and calculating similarity scores by comparing matched morphological segments, we automatically identified potential matched neurons. We matched BANC neurons to reconstructions in FAFB-FlyWire (v.783)^17,19^, MANC (v.1.2.1)^11^, FANC (v.1116)^8^ and hemibrain (v.1.2.1)^16^. FAFB is a dense reconstruction of the female brain. MANC is a dense reconstruction of the male VNC. FANC is a female VNC dataset, with part of the abdominal ganglion truncated, which has been sparsely reconstructed, and for which sparse annotation data are available, including sensory and effector neurons^91,220–223^, ANs and DNs^15^ and hemilineages^224^. Hemibrain is a dense reconstruction of around one-third of the female central brain, including a high-quality reconstruction of the mushroom body and central complex. Recently updated^29^ cell-type names from FAFB were the preferred names for brain matches, from MANC for VNC matches, FANC for known female-specific^15^ ANs and motor neurons^9^ (in which the annotation is better than in MANC), and hemibrain for the central complex^7^ (in which the annotation is better than in FAFB). Following automated NBLAST scoring, we manually reviewed candidate matches. For sensory neurons, ANs and DNs, and intrinsic neurons of the brain, this manual review involved co-visualizing the meshes of matched neurons in three orthogonal 2D projections and evaluating their correspondence. For ANs and DNs, we followed up this 2D comparison with co-visualization and manual evaluation in 3D using Neuroglancer. For intrinsic neurons of the VNC, we also used connectivity^225^ to automatically determine their similarity to MANC neurons^31^. When the top matched cell type agreed between NBLAST and connectivity, we assigned the neuron to that cell type. When these potential matches were in conflict, we co-visualized the BANC and MANC neurons in 3D in Neuroglancer and manually reviewed them to determine the correct cell type. High NBLAST scores (for example, above 0.3, self-match normalized) generally indicated a good likelihood of a correct match in the central brain. Iterative proofreading and matching increased the population of identified cells; this is because low NBLAST scores often indicate issues with neuron reconstruction, which can be improved with additional proofreading. The top NBLAST match was correct for 65.7% of central brain neurons (mean normalized NBLAST score of 0.48 for correct hits), 28.0% of VNC neurons (0.53) that are not sex-specific, and only 19.9% of optic lobe neurons (0.49). Overall, we matched 118,380 neurons by NBLAST followed by manual review. For the remainder, mostly of the optic lobes, we used wide-scale automated typing, as described below.

#### Expert manual annotation of sensory and effector neurons

Many afferent neurons (that is, sensory neurons) and efferent neurons (that is, effector neurons: motor neurons, endocrine neurons and neurons targeting the viscera) have been incompletely annotated in existing connectome datasets. Therefore, we used comparisons to the literature and the domain expertise of our authors to determine their cell types and functions.

Specifically, we identified leg and wing motor neurons by their morphology and connectivity, as previously described^9^. The key identifying features we used were the exit nerve of the axon, the relative trajectory of the primary neurite, the relative position of the soma and unique features of the dendritic morphology. Front, middle and hind limb neuropils differ in terms of specific morphology, yet the identifying motor neuron features largely retain their relationships, which enabled us to identify homologous motor neurons in each neuropil^10^. We confirmed morphological identification by comparing these motor neurons on the basis of the sources of common synaptic input^9^. We used morphological comparisons to the literature to identify the effector neurons of the antennae^226^, eyes^227^, neck^64^, crop^228,229^, haltere^8^, proboscis^95,229–231^, pharynx^95,229,230,232^, salivary glands^95,229^ and uterus^233^; octopaminergic effector neurons involved in ovulation^193,234,235^; endocrine neurons of the brain and VNC^131,201,236,237^; and chemosensory, tactile and proprioceptive sensory neurons from the head^229,238–240^, eyes^241^, antennae^241^, proboscis^229,238,239^, cibarium^242^, legs^223^, abdomen^233,243^, wings^244^ and halteres^221,245^. We used reports in the literature to identify the neuropeptides released by specific cell types^44,54,198,246^ and the sites of action of those neuropeptides, including the ureter^247,248^, the digestive tract^54,198,246^ and the reproductive tract^61,234,235^. For some sensory neurons of the head (tactile neurons of the head and some proboscis and pharynx tactile and gustatory neurons), we used the cell-type identification process to assign the neurons to a named nerve^238,241^ that was not separately named in our nerve seed planes (because the nerve only separates from the larger bundle in the periphery, outside the BANC dataset; for example, eye nerve or stomodeal nerve). We assigned effectors to 1 of 24 body part categories where possible (99.4%). Of these, based on the literature and the presence or absence of large dense core vesicles, we determined 805 to be motor neurons and 221 to be endocrine or neurons that target the viscera. Furthermore, we assigned sensors to 1 of 45 body part categories where possible (99.8%); we have 1,169 orphan sensory neurons without a nerve annotation. In some cases, we used data from the larval fly (putative leg nociceptive^249–251^, putative oxygenation^236,252,253^ and aorta sensory neurons^54^) to annotate presumptive homologous neurons. Adult nociceptors in the abdomen and labellum have been reported^51^, including direct mappings to BANC reconstructions. We performed additional expert review and annotation of chordotonal, campaniform and hair plate neurons of the VNC, including those of Wheeler’s organ, the prothoracic organ and the metathoracic organ^8,222,223,254,255^. One neuron whose axon leaves the dataset was demarcated as efferent but also sensory, SNpp54, which is thought to associate with a connective tissue strand^11,256^. We could not identify chordotonal and bristle neurons of the haltere based on the literature (these are a minority input from the halteres), or chordotonal neurons of the proboscis and eye. The majority of neurons that leave or enter the abdominal nerves are of unknown peripheral identity, and many are dimorphic or sex-specific. We typed them by cross-matching to the FAbG dataset, an in-progress connectome of the female abdominal neuromere/ganglion (J.Z. et al., manuscript in preparation). These cell types have ‘abg’ in the cell type name. We start their type names with ‘EFF’ for effector neurons, as we found it hard to discriminate putative striatal motor neurons (MN) from visceral/circulatory neurons (EN). Note that the rate of sensory neuron recovery is higher in BANC than in MANC, and this contributes to the higher cell count in the BANC VNC. The higher cell count in the BANC VNC may also be partially due to sexual dimorphism, as MANC is from a male fly.

#### Automated typing by morphology and connectivity

Cell-type identification in connectomes has largely relied on morphological similarity (typically quantified via NBLAST^30^) followed by human review, which has often depended heavily on specific features like primary neurite correspondence to hemilineages^218^. Although effective for morphologically distinctive neurons, this approach can be limiting in cases in which multiple cell types share similar arbour geometry but differ in their synaptic partners (for example, this approach cannot easily resolve different types of columnar optic lobe neurons and central brain interneurons). Connectivity-based methods such as NTAC^257^ address this problem by propagating labels within the graph of a single connectome, but these methods require examples from every cell type and struggle when the graph topology is only partially reconstructed. In practice, expert annotators tend to resolve such ambiguities by considering morphology first and then refining assignments using connectivity. We automated this process by creating an iterative cross-dataset alignment algorithm that transfers cell-type labels from FAFB to BANC. First, NBLAST morphological scores provide initial type hypotheses and constrain the search space; then, at each iteration, BANC connectivity profiles are compared with fixed FAFB type-level reference centroids (mean input–output connectivity at 1-hop and 2-hop levels, scored via a cosine-weighted Jaccard ensemble metric), and the resulting connectivity scores are merged with NBLAST scores using an adaptive weight (alpha) that ramps from 0.05 (NBLAST-dominant) to 0.95 (connectivity-dominant) over 80 iterations. Hard assignments were made via a cell-type assignment ‘capacity’-constrained procedure. That is, neurons with NBLAST data were first matched to individual FAFB neurons (each claimable by at most one BANC neuron), then remaining neurons were assigned to types by soft probability ranking, with per-type capacity limits derived from FAFB left–right count variability, with a +10% allowance. We excluded 1,125 NBLAST matches from the assignment pool on the basis of expert assessment of these matches as obvious mis-assignments. Neurons with a nucleus (that is, a soma in the dataset) were not permitted to match to FAFB cell types whose somata lie outside the dataset (for example, photoreceptors and lamina neurons). The best iteration by holdout accuracy was retained via early stopping.

We applied this algorithm to 133,120 proofread BANC neurons across 8,816 FAFB cell types (whole brain, bilateral). Of these BANC neurons, 81,110 (60.9%) had existing cell-type annotations and served as tier-1 anchors; 41,911 (31.5%) lacked annotations, but had NBLAST matches providing soft initialization (tier 2); and 10,100 (7.6%) had neither and began unassigned (tier 3). Coverage was asymmetric across hemispheres: on the right, 3,139 out of 61,175 neurons (5.1%) lacked cell-type annotations, whereas on the left, 11,660 out of 58,119 (20.1%) were untyped, which reflected the historical concentration of manual annotation effort on the right side. In the optic lobes specifically, 37,742 out of 91,698 proofread neurons (41.2%) lacked a human-verified cell type—10,410 out of 48,846 (21.3%) on the right and 27,332 out of 42,852 (63.8%) on the left—across 685 FAFB optic lobe types. Our subsequent alignment proposed cell types for 29,109 of these, leaving 8,633 of 91,698 neurons (9.4%) with no cell type of any kind. In the right optic lobe, the algorithm achieved 82.7% holdout accuracy (versus 43.9% for NBLAST alone and 65.0% for NTAC), with 90.4% accuracy on the benchmark type Mi1 and 0.938 Spearman correlation between BANC and FAFB per-type neuron count distributions. Our code can be found on our Harvard Dataverse repository (10.7910/DVN/7WTH1N); bancpipeline/banc/alignment/ in [bancpipeline_archive.zip].

#### Neurons of the neck connective

We reviewed all profiles in the two seed planes through the neck connective [neck_connective_y92500.parquet, neck_connective_y121000.parquet]. We proofread 3,686 of the neuronal profiles to backbone proofread status, out of a total of 3,730 seeded profiles. We then matched these neurons to cell types in FAFB and MANC, as described above, except for the 8 female-specific DN cell types and 31 female-specific AN cell types identified in a previous study^15^, which we instead matched to FAFB and FANC. Previous work bridged a proportion of ANs and DNs between FAFB and MANC using available experimental data^15^, which was a valuable resource for our matching efforts. We identified 1,849 ANs, of which we matched 1,768 (corresponding to 527 cell types), and 1,316 DNs, of which we matched 1,304 (corresponding to 473 cell types). We also identified 13 sensory DNs (afferent axons that enter through a brain nerve and project through the neck connective to the VNC, discussed in more detail in previous work^15^) corresponding to 5 cell types, 517 sensory ANs (afferent axons that enter a VNC nerve and project through the neck connective to the brain) corresponding to 42 cell types and 5 efferent ANs (ANs that project out of other nerves) corresponding to 3 cell types, including EAXXX079, which may be the leucokinin ANs in ref. ^258^. We identified all 16 of the female-specific DNs (corresponding to 8 cell types) and 38 of the expected 70 female-specific ANs (corresponding to 17 of the 31 cell types). For ANs, sensory ANs and efferent ANs, we used the MANC cell-type name except for the female-specific ANs, for which we used the FANC name; for DNs and sensory DNs, we used the FAFB name. When this resulted in the same cell-type name for neurons of clearly different morphological cell types (which became apparent when considering the full neuron rather than just the brain or VNC half), we appended an underscore and a letter to the FAFB or MANC name. We also identified and proofread 49 efferent neurons of the neck that leave through the cervical nerves. These are neck motor neurons, and we named them as in ref. ^64^. Because they do not traverse the entire extent of the neck connective, they are not included in our count of 3,686 backbone proofread neurons of the neck connective. We did not use sensory or efferent ANs and DNs in our analysis of ANs and DNs. In our review of the neck connective, we identified 18 ANs and DNs that seemed to have developed abnormally; in some of these cells, an ascending or descending arbour was observed only stochastically. For example, DNge079 on the left side (in MANC named DNxl080) has a mis-targeted dendrite located in the VNC rather than the central brain. Both the left and right copies of IN08B003 are ANs in the BANC dataset, but they are intrinsic neurons of the VNC in MANC and in FANC. We determined that the cell type DNg28 leaves the brain through the maxillary-labial nerve and after it re-enters through the same nerve, its processes remain outside of the glial sheath surrounding the CNS as it then traverses the neck to envelop the outside of the VNC and target neurohemal release sites; therefore, we re-classified it from a DN to an efferent cell type. In the BANC dataset, as in FAFB, DNg25 is not identifiable, and DNd01 is not a DN but rather a central brain intrinsic neuron^15^.

### Annotation taxonomy

We developed an annotation taxonomy by consensus in our consortium of authors. In this taxonomy, we annotated neurons hierarchically by flow relative to the CNS (afferent, inputs to the CNS; intrinsic, contained in the CNS; efferent, leaves the CNS), superclass (for example, sensory, motor, visceral/circulatory, ascending and descending), cell class (for example, chordotonal organ neuron, leg motor neuron and Kenyon cell), cell subclass (for example, wing-steering motor neuron, front-leg hair plate neuron and PPL1 dopaminergic neuron) and individual cell type. Cells were also tagged with associated metadata (region, side, nerve, body part sensory, body part associated with effector neurons, peripheral target type, cell function, cell function detailed, hemilineage, neurotransmitter verified, neuropeptide verified, FAFB (v.783) match ID, MANC (v.1.2.1) match ID, and other names). The full list of terms used in each category is listed in Supplementary Data 1. We imported annotations from cell-type matching to existing *Drosophila* connectomes^11,17,19^ as well as those that proofreaders and the community contributed through a custom Slackbot (https://github.com/jasper-tms/the-BANC-fly-connectome/blob/main/slackbots/annotation_bot.py) directly to CAVE, which facilitated real-time tagging and collaborative refinement. We updated annotations as proofreading progressed [banc_888_meta.feather]. Annotations are publicly available through FlyWire Codex and on CAVE [codex_annotations.parquet, cell_info.parquet, cell_representative_point.parquet]. We used cell_info for community annotations, and codex_annotations for centralized annotations federated by the core BANC team. We used SeaTable internally to collect, draft and stage our annotations before uploading to FlyWire Codex and CAVE.

Elsewhere in the literature, ‘effector’ is sometimes understood as denoting a muscle or gland. Here we use ‘effector neurons’ to describe motor neurons, endocrine cells and efferent neurons targeting the viscera. All these cells effectuate change outside the CNS; therefore, they are the relevant output units of the CNS.

### Influence

The influence score^42^ quantifies the influence of a neuron or group of neurons, called the source, on each of the other neurons in the network. It is a measure of steady-state signal activation, which results from continuous stimulation of source neurons. We computed steady-state activation assuming a linear dynamical model,

1 $$\tau \frac{dr(t)}{dt}=-r(t)+{Wr}(t)+s(t),$$

where *r* is the vector of neural activity, *W* is the connectivity matrix, *τ* is the network time constant, and *s* is the simulated neural stimulation. For each source, all elements in *s* corresponding to the source neurons are set to one, whereas the remaining elements are fixed at zero.

The weight of each connection is taken as the number of synapses in that connection, normalized by the total count of input synapses onto the postsynaptic cell in question. That is, if *c*_*ij*_ is the synapse count from presynaptic neuron *j* onto postsynaptic neuron *i*, then the total input count for neuron *i* is

2 $${N}_{i}=\sum _{j}{c}_{{ij}},$$

and the connectivity weights were set to

3 $${w}_{{ij}}={c}_{{ij}}/{N}_{i}.$$

This type of normalization follows previous work and has been shown to qualitatively capture experimental observations^32,259^. Connections with fewer than five synapses were filtered out from the analysis to reduce reconstruction or biological noise. All connectivity weights were treated as non-negative values. To ensure stable neural dynamics, we re-scaled *W* such that its largest real eigenvalue is 0.99.

We computed the steady-state solution for the assumed network dynamics by

4 $${r}_{\infty }=-{(W-I)}^{-1}s$$

separately for each source vector *s*. As *W* is a highly sparse matrix, we could compute this solution efficiently using the sparse matrix parallel computing libraries PETSc and SLEPc (https://petsc.org/ and https://slepc.upv.es/, respectively).

If the source is one cell, and we are interested in a single target cell, we simply take the steady-state activity of the target *r* in response to the source. We define *r*_*ij*_ as the steady-state response of target cell *j*, given stimulation of source cell *i*. To better understand the steady-state solution, note that using the von Neumann series, it can be rewritten as an infinite sum of powers of the connectivity matrix *W* multiplied by the source vector *s*,

5 $${r}_{\infty }=s+{Ws}+{W}^{2}s+{W}^{3}s+\ldots .$$

Here the first term is the immediate impact of the source (or sources), and the remaining terms quantify its impact across immediate synaptic connections—or one hop—across two hops, and so on. In other words, we are summing over source propagations across different hops until all direct and indirect pathways between source and target neurons are covered.

Often, we were interested in pools of related target cells (for example, a pool of related motor neurons). Thus, for a target pool *T* that contains the indices of the |*T* | = *N* target neurons, we took the average steady-state response of each cell in the target pool

6 $$\overline{{r}_{T}}=\frac{1}{N}\sum _{j\in T}{r}_{{ij}}.$$

Similarly, we were often interested in a pool *S* of related source cells, where *S* contains the indices of the source cells. Here we simulated activity in all sources individually and averaged the results. In this case, for a source pool of size |*S*| = *M*, the average response is

7 $$\overline{{r}_{T,S}}\,=\frac{1}{{NM}}\sum _{i\in S,j\in T}{r}_{{ij}}.$$

Alternatively, because the steady-state solution *r*_*∞*_ is linear in the source vector, it was sometimes more convenient to just simulate activity in all source cells simultaneously. In this case, if *r*_*j*_ is the response of the *j*th target cell to the simultaneous activity of all source cells, we took

8 $$\overline{{r}_{T}}=\frac{1}{{NM}}\sum _{j\in T}{r}_{j}.$$

In this type of simulated network, $\bar{r}$ will generally decay exponentially as the distance increases between the source and the target (in network space). To correct for this, we took the logarithm of $\bar{r}$; note that $\log (\bar{r})$ should be generally proportional to the number of hops separating the source and the target. Because $\log (\bar{r})$ is generally negative, we added a constant *c* that brought the values of $\log (\bar{r})$ into the non-negative range for ease of display. The resulting value is called the adjusted influence:

9 $$\mathrm{Adjusted}\,\mathrm{influence}=\log (\overline{{r}_{T}})+c.$$

In a few cases (only as noted in the corresponding figure legends), we calculated the adjusted influence corrected for the number of neurons in the source pool:

10 $$\mathrm{Adjusted}\,\mathrm{influence}=\log (\overline{{r}_{T,S}}\,)+c.$$

This is because, in these cases, we were interested in quantifying the total influence of a sensory cell subclass onto effector neurons. In all other cases, we were interested in quantifying the influence between the average source and target neuron of the given classes, and so used equation (9).

We used *c* = 24 because this ensured that all adjusted influence values are non-negative (given that −24 was approximately the minimum value of $\log (\bar{r})$ we observed). Across the entire CNS, a small and discrete group of cells had $\log (\bar{r})$ ≪−24 for any source; as these cells were not well-connected to the graph, we set these adjusted influence values to 0.

We confirmed that adjusted influence is approximately inversely proportional to the number of synaptic hops separating the source cells and target cells, as expected, and this was true for two different published metrics of hop length (Fig. 2b and Extended Data Fig. 4b; see below for details of these previous metrics). Thus, adjusted influence is essentially a computationally efficient and deterministic method of estimating a quantity that is proportional to the effective number of hops separating the source and the target. Because the number of hops is an unsigned quantity^19,41^, it is reasonable that adjusted influence is also unsigned. Moreover, we do not actually know the signs of all connections in the connectome, particularly concerning glutamatergic, dopaminergic, serotonergic, octopaminergic and tyraminergic synapses. Notably, even inhibitory cells can drive behaviour, for example, via disinhibition (the only output of the vertebrate cerebellar cortex consists of GABAergic Purkinje cells, which trigger motor output when they pause firing, thereby disinhibiting their downstream targets^260^).

Compared with previous metrics of hop number, adjusted influence has several advantages. First, we have an explicit expression for the steady-state solution, which made the computation more efficient relative to comparable activity propagation approaches^17,32,41^. Second, the steady-state solution is linear in the source vector, such that it can in principle be summed across different sources.

In our influence score calculations, the maximum number of hops taken from any source to target was relatively small: the median number of topological hops between a sensory neuron and each AN or DN was 3, the 99th percentile was at 5, and the max was at 6, with 99.7% reachable (the median hops to an effector from an AN or DN was also 3).

Rather than taking the steady-state activity as the basis for this influence metric, we also considered using the initial slope of the neural activity. However, the initial slope turned out to be directly proportional to the chosen source vector, which made it unsuitable as a measure to quantify network-wide influences. We also considered projections of the above dynamics into the top 1,000 eigencircuits, similar to previous work^38^, but we found that this truncation was unsuitable for our purposes to well approximate the full network dynamics.

Note that as with the synaptic count between two neurons, the adjusted influence between two neurons is best understood relative to adjusted influences calculated between comparable sources and targets. There is no value over which an important interaction is assured.

We computed the influence scores reported in this paper using Python (v.3.13.2), and we executed all computations using a MacBook Pro running macOS Monterey (v.12.6.9). The code used to compute the influence scores is available as a separate Python package^42^ (see the ‘Code availability’ section), and as [connectome_influence_calculator_archive.zip, influencer_archive.zip].

### Alternative metrics for indirect connectivity

For comparisons with our influence scores, we used two complementary probabilistic graph traversal algorithms to model information flow through the CNS. First, we applied the signal cascade approach^41^, in which activity propagates from a set of source neurons to downstream targets based on synapse counts, treated as proxies for synaptic strength. A key feature of this model is that neurons are activated only once and then enter a deactivated state, enabling assessment of potential temporal sequences of activation.

Second, we used an information flow model^17,32^, in which neurons are probabilistically recruited based on the fraction of synapses received from already recruited neurons. This model allows ongoing activation from previously active neurons and assigns each neuron a rank that reflects its integration point in the circuit. Although these ranks do not correspond to true physiological latency, this approach enables systematic inference of information flow directionality and network layering across the CNS.

A ‘max flow’ algorithm was used in a previous study^29^ to estimate the importance of ANs and DNs in sensory-to-effector paths. This method finds the pathway that supports the greatest information flow between ‘source’ and ‘sink’ neurons given a capacity-constrained network. In the max flow algorithm, the input-normalized synaptic input to any neuron is taken as a proxy for the edge-capacity linking any two nodes (that is, neurons) of the network. Although this method does not suffer from exponential attenuation when the signal propagates from source to target (unlike methods based on repeated multiplication of the connectivity matrix), it overlooks parallel pathways of lower and even similar information flow.

### Betweenness centrality

To quantify how strongly each neuron acts as a bottleneck in the BANC dataset, we computed each neuron’s betweenness centrality, a standard network metric that quantifies how often a given node lies on the shortest paths between pairs of other nodes (excluding the endpoints)^261–264^. Neurons with high betweenness centrality lie on many routes between other neurons and are positioned at putative bottlenecks in the network.

We calculated two different versions of betweenness centrality: all-to-all betweenness and sensory-to-effector betweenness. To calculate all-to-all betweenness, we computed the unnormalized, unweighted, directed betweenness centrality for all the neurons in the network using the Brandes algorithm^262^ as implemented in the Python interface of igraph^265^ (Fig. 3a and Extended Data Fig. 5a).

We represented the network as a directed graph *G* = (*V, E*), where *V* is the set of neurons and *E* is the set of directed edges representing connections between neurons. We included a directed edge only if the connection had at least five synapses. The all-to-all betweenness of a neuron *v* is defined as follows^262^:

11 $${\mathrm{Betweenness}}_{\mathrm{all}-\mathrm{to}-\mathrm{all}}(v)=\sum _{s,t\in V,s\ne t,s\ne v,t\ne v}\frac{{\sigma }_{{st}}(v)}{{\sigma }_{{st}}},$$

where *σ*_*st*_ is the number of shortest paths from *s* to *t* and *σ*_*st*_*(v)* is the number of shortest paths from *s* to *t* that include *v*. If there are multiple shortest paths, $\frac{{\sigma }_{{st}}(v)}{{\sigma }_{{st}}}$ indicates the fraction of such shortest paths that include neuron *v*. We then sum this fraction over all pairs of neurons *s,t ∈V* with *s* ≠ *t*, and the neuron *v* is neither the source nor the target (*s* ≠ *v, t* ≠ *v*). As our graph is directed, paths from *s* to *t* must follow edge directions.

To quantify how strongly each neuron acts as a bottleneck in the sensory to effector information flow, we calculated the source–target betweenness centrality^263^. Specifically, we used the same unnormalized, unweighted, directed betweenness centrality but restricted the source–target pairs to sensory neurons (sensory ascending, sensory descending and sensory) as sources *S* and effector neurons (motor, visceral circulatory and ascending visceral circulatory) as targets *T* when accumulating betweenness scores (Fig. 3a):

12 $${\mathrm{Betweenness}}_{\mathrm{sensory}-\mathrm{to}-\mathrm{effector}}(v)=\sum _{s\in S,\,t\in T,\,s\ne t,\,s\ne v,\,t\ne v}\frac{{\sigma }_{{st}}(v)}{{\sigma }_{{st}}}.$$

### Spectral clustering

We adapted a spectral clustering algorithm^266^ to partition the CNS into modules of highly interconnected cells. For this analysis, we focused on intrinsic neurons of the central brain and VNC, ANs, DNs, visual projection neurons and visual centrifugal neurons [banc_888_meta.feather]. We chose to exclude optic lobe intrinsic neurons because they are so numerous that they end up dominating the analysis. Starting with these 55,643 cells, we iteratively pruned cells that did not have at least one input and output partner among the remaining cells (for example, because all their input comes from sensory neurons, or all their output goes to motor neurons). This left 50,568 cells as the input to this analysis.

To apply spectral clustering, we first specified our population of *N* cells of interest and a desired number of clusters *k*. We then constructed a weighted, undirected graph whose nodes corresponded to these *N* cells and whose edge weights were derived from the connectome. More formally, edge {*i, j*} was assigned weight

13 $${a}_{{ij}}=\frac{1}{2}({w}_{{ij}}+{w}_{{ji}}),$$

where *w*_*ij*_ is the normalized synaptic input from presynaptic cell *j* to postsynaptic cell *i*, as defined above. We then computed the first *k* (*k* = the chosen cluster number) eigenvectors of the graph Laplacian, which resulted in a *k* × *N* matrix of unit-norm eigenvectors *X*. Each node then received a *k*-dimensional feature vector that was determined by its loadings onto the eigenvectors, which produced a *N* × *k* feature matrix *Y* with entries

14 $${y}_{{im}}=\frac{{x}_{{mi}}}{\sqrt{\sum _{m}{x}_{{mi}}^{2}}}.$$

Finally, we applied *k*-means clustering to these feature vectors to assign each node to a cluster. We decided to use 13 clusters because this produced a coarse-grained division at the approximate level of resolution we found relevant to our analysis and because the resulting cluster divisions largely corresponded to salient boundaries in the UMAP space of CNS neurons. Parameters used were as follows: cluster count = 13, embedding seed = 3, cluster seed = 10 and min connection strength = 1.

### Clustering influence by influence and connectivity

We clustered heatmaps using hierarchical clustering based on Ward’s distance using functions in R. In Fig. 6 and Extended Data Fig. 10, we used spectral clustering, as described above. For Extended Data Fig. 4f, we applied dynamic tree cut^267^ clustering (implemented as dynamicTreeCut::cutreeDynamic, using deepSplit = 4) to the efferent neuron cosine UMAP to delineate our well-grouped effector clusters. For Fig. 3, AN/DN clusters were obtained by hierarchical clustering on the connectivity PCA embedding of the typed input and output partners of each neuron. We demarcated putative functional clusters as follows. For each AN and DN (3,165), presynaptic and postsynaptic edges from the proofread synaptic edge list (no minimum-synapse threshold) were combined into a single profile and normalized to the total input of the postsynaptic neuron (input fraction). Query neurons and partner neurons were aggregated by cell type + specific neuromere innervation + nerve (if sensory or effector), if any. This step was important because many VNC types are serial homologues across related hemilineages in different neuromeres and entry or exit nerves. Pairwise cosine similarities between cell types were computed, PCA was performed on the standardized similarity matrix and the Marchenko–Pastur upper bound from random matrix theory (319 out of 1,228 components) was used to retain components. This 319-dimensional PCA representation was then hierarchically clustered with Ward’s minimum-variance linkage (ward.D2) on Euclidean distances, and clusters were extracted with dynamicTreeCut^267^. The selected parameters were deepSplit = 0, minClusterSize = 8. Each AN or DN inherited the cluster label of its cell_sub_type (cell type completeness = 1.000 by construction), which produced 17 clusters. For visualization, the 319-dimensional PCA representation was embedded in 2D with PCA–UMAP (Euclidean metric, n_neighbors = 100, min_dist = 0, spread = 10, n_epochs = 500, n_trees = 100, seed = 42). This PCA–UMAP, rather than a direct cosine UMAP on the partner matrix, was used as the primary embedding throughout Fig. 3 because it is aligned with the clustering feature space. The software used was R (v.4.2.1), with uwot^268^ (UMAP, v.0.1.14), prcomp^269^ (PCA, v.3.6.2), dynamicTreeCut^267^ (cluster extraction, v.1.63.1), cluster::silhouette^270^ (silhouette scoring, v.2.1.8.2) and mclust::adjustedRandIndex^271^ (ARI, v.6.0.1).

### Naming AN/DN clusters

We assigned names to clusters of ANs and DNs on the basis of their hypothesized functions. These clusters were defined automatically using connectivity, but their names were also based on the influence each cluster received from sensory neuron subclasses (Fig. 3e) and the influence they exerted onto effector cell subclasses (Fig. 3f), as well as against the documented behavioural roles of specific cells in individual clusters (Supplementary Data 9). Our reasoning was as follows. See super_class in [banc_888_meta.feather] for assignments.

The two flight-steering clusters are dominated by inputs from Johnston’s organ E neurons together with prosternal hair plates, wing-base campaniform sensilla and eye bristles, and they output predominantly to wing-steering motor neurons and the four neck-axis motor groups. The flight steering 1 cluster contains the major DNs with reported roles in flight steering: DNa03 (turning^272^), DNa04 (flight turning^273^), DNa15 (turning^274^), DNae003 (turning^272^), DNb01 (turning^274^) and DNp03 (turning^275^). Flight steering 2 is distinguished by strong haltere campaniform sensillum input and it also contains DNp28 (ocellar^17^). PCA–UMAP clustering separated these two clusters, but it seems probable that they both contribute to flight steering. The flight power cluster receives input from Johnston’s organ neurons and wing campaniform neurons, and its output is primarily towards wing-steering and wing-power motor neurons; it contains DNg07 (flight^276^), AN27X008 (flight^10^) and DNp20 (flight^277^).

The head-orienting cluster has all five top effectors in the neck-axis motor groups (roll, pitch, yaw, roll-pitch and neck) and its dominant sensors are head-mounted: eye bristles, prosternal hair plates and Johnston’s organ E neurons. It contains DNa06, DNb02 (turning^50^), DNb06 (turning^98^), DNg10 (reaching^278^), DNp15 (steering^279^), DNp17 (steering^280^), DNp22 (ocellar^277^) and DNpe013, all of which are probably involved in head-orienting or eye-orienting.

The grooming cluster is driven by labellum bristles, front-leg bristles, hair plates and front-leg taste pegs, with output to neck-axis motor neurons and front-leg motor neurons; it contains DNg62 (ref. ^281^), DNge011 (ref. ^281^), DNge012 (ref. ^281^) and DNge078 (ref. ^281^), all documented antennal-grooming DNs. The probing cluster is influenced by labellum bristles, front-leg hair plates and bristles as well as labellum external taste sensilla and outputs prominently to proboscis motor neurons and prothoracic neurosecretory cells. This connectivity profile suggests that the probing cluster mediates tactile sampling before feeding initiation, in line with the previous usage of ‘probing’ in the literature. The feeding cluster has the strongest taste-peg dominance of any cluster (front-leg, middle-leg and hind-leg taste pegs in the top five sensors) and proboscis motor neurons as the leading effector; it contains DNp44 (feeding^282^), DNp62 (hunger^183^) and DNg70 (neuromodulatory^168^). Some known nociceptive relay neurons also appear in the feeding cluster, for example, AN09B018 (ref. ^51^).

The taste–touch cluster combines mixed gustatory and tactile input from leg bristles, leg taste pegs and wing-margin bristles. It contains DNp29 (hunger^152^), DNg30 (memory and sexual activity^147^) and DNge138 (octopaminergic^283^). The tactile cluster receives mechanosensory bristle input from all four body parts (front-leg, middle-leg, hind-leg, thorax and wing-margin bristles) and routes broadly to motor systems with no single dominant effector; it contains DNge078 (antennal grooming^281^) and many uncharacterized cells probably involved in whole-body tactile integration (Fig. 3j). Other known nociceptive relay neurons also appear in the feeding cluster, for example, AN09B004 (ref. ^51^).

The threat-response cluster has body-surface bristle and taste-peg input, with multi-leg motor output, and it contains DNp06 (escape-takeoff^284^), DNp42 (backward walking^78^), DNg55 (steering^73^) and DNge053 (walking^73^). This connectivity profile suggests that this cluster coordinates avoidance behaviours. We manually assigned DNs related to the giant fibre system (DNp01 (ref. ^285^), DNp02 (ref. ^286^) and DNp04 (ref. ^286^)) because all these DNs have demonstrated roles in escape-takeoff, even though our PCA–UMAP clustering originally assigned these DNs to the taste–touch cluster. This result is probably because these DNs have unusual morphologies, which caused them to have a higher rate of false synaptic detections and registration-related image artefacts owing to their size. Therefore, we should be cautious in interpreting any automatic assignment of these cells to a functional cluster based on their connectivity. These were the only cell types we manually reassigned to a new AN/DN cluster.

The vibratory cluster is dominated by input from chordotonal organs (hind-leg, middle-leg, front-leg, neck and Wheeler’s chordotonal neurons), with broadly distributed motor output. The proprioceptive cluster is distinguished from the tactile and vibratory clusters by the fact that the proprioceptive cluster receives hair-plate input across multiple legs, together with input from leg bristles and leg orphan sensory neurons. It contains AN09B029_b, which was used as a proprioceptive example in Fig. 5c.

The walking cluster receives leg-bristle and leg-hair-plate input, and it outputs to all three leg motor groups together with neck motor neurons; it contains DNa01 (turning^287^) together with DNb08 (leg CPG^288^), DNg100 (leg CPG^288^, forward walking^73^), DNg97 (forward walking^73^), DNg13 (turning^98^), AN17A026 (backward walking^289^), DNp09 (halting^72,290^) and DNp07 (landing, backward walking^66^). The walking-steering cluster also receives leg-bristle and leg-hair-plate input, but its output is dominated by neck motor neurons (comprising all four neck-axis motor groups) and it is enriched in head-mounted sensors, including olfactory, thermosensory and hygrosensory antennal sensing. It contains DNa02 (turning^287^), DNa11 (turning^272^) and MDN (DNp50, backward walking and steering^291^).

The postural control cluster is the most heterogeneous of the AN/DN clusters. Overall, it receives strong leg-bristle and wing mechanosensory input, and it sends output to leg, abdomen and neck motor neurons. This cluster contains DNp10 (landing^66^), DNp47 (landing^292^), DNp11 (escape-takeoff^286^), DNg14 (abdomen curl^50^), DNb05 (turning^98^), DNg75 (forward walking^73^), DNge050 (walking^73^), DNp60 (wing pump^293^), DNpe050 (wing elevation^293^), DNp13 (oviposition^61^), DNp37 (vaginal plate opening^193^), AN27X004 (flight^10^) and several ANs reported to modulate wing vibration, at least in males^294^ (AN08B031, AN08B043, AN08B059 and AN08B069). The range of behaviours associated with this cluster (landing, takeoff, walking, abdomen and wing vibration) suggests a general function in linking mechanosensation with postural control rather than a specific role in a particular behaviour.

The reproduction cluster has a uniquely strong output to neurosecretory effector neurons (front-leg, middle-leg, hind-leg and neck neurosecretory cells are among its top five effectors), with leg-bristle and abdominal sensory input. It contains oviDNa_a, oviDNa_b and oviDNb (all oviposition^61^), DNg104, DNg34 and DNge149 (octopaminergic^124,283^), and DNc01 and DNc02 (reproduction, feeding and neuromodulatory^162^). The visceral control cluster receives input from visceral and interoceptive sensors (abdomen oxygenation, anterior digestive multidendritic neurons, hemolymph sensory neurons, pharyngeal taste and abdominal terminalia bristles) and it sends output to neurosecretory cells and thoracic–abdominal motor neurons; it contains DNp25 (ref. ^282^), DNge172 (oviposition^50^) and AN27X017 (sleep, halting^295^) (Fig. 3g).

### Naming effector groups

We assigned names to the 15 effector clusters using the same principles as for the AN/DN clusters. Cluster identity was set automatically from the UMAP of efferent neurons built from influence from ANs and DNs (Extended Data Fig. 4f and Supplementary Data 7 and 9). See super_class in [banc_888_meta.feather] for assignments.

Front leg and middle-hind leg groups together account for the bulk of leg-muscle motor neurons, separated by limb and body side. The front-leg cluster contains the tergotrochanter (jump-extending) motor neuron, the trochanter flexors and extensors, the sternal rotators and the tergopleural promotors, reaching the prothoracic leg through the prothoracic, accessory prothoracic and ventral prothoracic nerves. The middle-hind leg cluster contains the tibia flexors and extensors, femur reductors, tarsus levators and depressors, and long-tendon-muscle motor neurons of the mesothoracic and metathoracic legs^8,9^. Both receive their strongest sensor input from leg proprioceptors^223^ and descending influence from our walking, walking-steering and postural control DN clusters. The split between leg groups reflects differences between target nerve and front-leg versus middle and hind-leg DN input. The leg groups are also the only groups to be bilaterally split.

The flight-steering group is the direct wing-steering and haltere-steering motor pool. It contains the canonical wing-steering motor neurons b1, b2, b3, i1, i2, iii1, iii3, iv1–iv4 and tpn, together with the haltere motor neurons MNhm03, MNhm42 and MNhm43 (ref. ^276^). The flight-energy-power group combines the indirect flight-power motor neurons (DLM1-5 and DVM1a-c, DVM2a-b and DVM3a-b, with haltere power motor neurons hi1, hi2 and hiii2) alongside the wing-targeted FMRFamide-positive neurosecretory cells EN27X010 (FMRFamide is myomodulatory^296^). Both groups receive their strongest sensor input from haltere campaniform sensilla and Johnston’s organ afferents^221,244^, and they receive descending influence from both the flight-steering DN clusters.

The head–eye–antenna group contains the neck motor neurons CvN1–CvN11, CvNA1/A2, VCvN1–VCvN3 and DProN1–DProN9 (ref. ^64^), together with the retina motor neurons CB0901 and CB0804 and the antenna motor neurons CB0886 and CB0723, reaching head structures through the cervical, dorsal prothoracic, ventral cervical and antennal nerves, perhaps for gaze control^68^. The proboscis–antenna group is dominated by the proboscis motor neurons^95,232^ MN1–MN9, MN3L/M, MN2Da/Db, MN2V and MN4a/b, plus the antenna motor neuron CB0750, reaching their targets through the maxillary-labial and pharyngeal nerves. Both receive strong head-bristle and prosternal hair-plate input. Their strongest descending influences are the head-orienting and walking-steering DN clusters. The split between the head–eye–antenna and proboscis–antenna clusters probably reflects orienting versus proboscis-positioning roles.

The ingestion–digestion group combines the pharyngeal pumping motor neurons MN10, MN11D, MN11V and MN12D, the proboscis motor neuron MN5, the salivary motor neuron MN13, the crop-entry motor CEM^228^ and the efferent DNg28 (ref. ^198^) (in the BANC dataset, we discovered that this cell has an axon that does not descend into the VNC, but instead wraps around its outer surface, forming peripheral neurohemal sites, and is therefore not a canonical DN). Thus, the ingestion–digestion group includes the alimentary motor chain from pharynx to crop^54^. The feeding–endocrine group is composed of suboesophageal neurosecretory cells with Hugin expression (SEZ_NSC_Hugin) and secondary CCAP/Hugin co-expressing cells, including CB2748 (ref. ^297^). This finding is interesting, as Hugin couples a gustatory state to feeding suppression and locomotion^54,236^. Both effector groups receive pharyngeal-nerve, enteric-gustatory and cibarial mechanosensory input. They receive strong influence from the AN/DN clusters linked to feeding, visceral-control and taste–touch.

The energy homeostasis group comprises the canonical brain neurosecretory cells^175,201^. Exemplars include the insulin-producing m_NSC_DILP cells (DILPs 2, 3 and 5, also expressing drosulfakinin^183,292^), m_NSC_DH44 (metabolic and arousal control^298^), l_NSC_DH31 (diuresis^138,299^), l_NSC_CRZ (corazonin, stress and metabolic mobilization^57^), l_NSC_ITP (ion-transport peptide and hydration^144^), m_NSC_DMS (myosuppressin^201^) and SEZ_NSC_CAPA (capability, fluid balance^53^). Strong inputs include endocrine cells, the cibarial mechanosensory neuron, leg proprioceptors and clock neurons. The energy homeostasis group receives influence from AN/DN clusters devoted to visceral control and taste–touch.

Abdominal motor groups (abdomen motor 1, abdomen motor 2 and thoracic–abdominal) comprise abdominal muscle motor neurons reaching their targets through the abdominal nerve trunk and (probably) the second and fourth abdominal nerves. Abdomen motor 1 is the largest of the three groups. Abdomen motor 2 is a smaller group dominated by EFFabg28, which projects primarily through the second abdominal nerves. The thoracic–abdominal group contains a single cell type (ENXXX226), presumably innervating thoracic–abdominal muscles. These effector groups receive strong influence from abdominal-nerve chemosensors and dorsal-metathoracic proprioceptors. They also receive influence from the AN/DN clusters labelled as proprioceptive, postural control, flight steering 2 and grooming.

The abdomen-positioning group is dominated by motor neurons, including the uterus motor neurons^233^, and we predict that about two-thirds are female-specific. The cluster may be a female-dimorphic abdominal-bending pool that positions the abdomen for egg-laying (oviposition). Abdomen-reproductive is largely visceral–circulatory and is mainly comprised of Doublesex-positive octopaminergic neurons^193,234,235^ of the reproductive tract and Dh44–leucokinin co-expressing neurons that might be involved in diuresis or defecation and stress tolerance^53^. This group also contains a smaller set of Capa-expressing neurons, which are probably involved in controlling gut motility^300^. The abdomen–ureter group is visceral–circulatory, and it contains PDF-expressing neurons that innervate the renal system^247,248^. All three receive abdominal-trunk chemosensory and dorsal-metathoracic proprioceptive input, but they receive different influence from AN/DN clusters: the abdomen-positioning effector group is influenced most strongly by the grooming cluster together with the feeding and probing clusters, whereas the abdomen–reproductive and abdomen–ureter groups are mainly influenced by the visceral control and taste–touch clusters.

### Naming CNS networks

We gave the 13 CNS networks descriptive names by inspecting their constituents. Our reasoning was as follows. See cns_network in [banc_888_meta.feather] for assignments.

Three networks are primarily in the VNC, but each has an associated brain region through ANs and DNs. The abdominal VNC network covers the abdominal ganglion and consists of VNC intrinsic and ascending and serially convergent neurons. Leg VNC is the largest CNS network and comprises the single-leg-neuromere intrinsic neurons of the prothoracic, mesothoracic and metathoracic leg circuits. The dorsal VNC network has the largest brain contingent of the three owing to feedback to the brain from the haltere and wing campaniform sensilla and the flight control circuitry in the dorsal VNC neuropils.

Three networks are primarily in the brain. The lateral brain network straddles the central brain and the VNC, linking lateral central-brain neuropils to the VNC via ANs and containing lateral horn output neurons. The inferior brain network links the central brain and the optic lobe projections at the optic-glomeruli. The posterior brain network is centred on the WED and AMMC posterior-medial neuropils and contains WPNs and Johnston’s organ E neurons.

Four networks are denoted as left–right copies, because the connections between the left–right copies of these networks are relatively sparse, and so spectral clustering recovers them as separate networks. Specifically, the left and right visual networks span the optic lobe and central brain, and they are dominated by visual-projection neurons. The left and right olfactory networks include the mushroom body and antennal lobe of the central brain, dominated by Kenyon cells and antennal lobe projection neurons.

Two networks straddle the SEZ and superior protocerebrum. The flange median bundle network is named for the neuropil it occupies. It includes the interface between the gustatory system and ANs and DNs, with taste-bristle gustatory neurons, SEZ projection neurons and the mAL lineage; it gets information from the VNC through its group of ascending neurons. The superior brain network comprises lateral horn output neurons, circadian DN1p–DN3 neurons, mushroom body PAM–PPL1 dopaminergic cells, and large numbers of relatively uncharacterized superior brain cell types.

Finally, the central complex related network includes the fan-shaped-body system (for example, PFNp, PFNa, FC, FS, vDelta, hDelta, Delta7 and FB tangential cell types), which sits entirely in the central brain; and the ellipsoid-body system (for example, TuBu, EPG, ER1, PEN, PEG cell types), which reaches into the optic lobe through the MeTu input that feeds the ellipsoid-body ring. If greater than 13 clusters are set, the algorithm will separate FB from EB networks.

### Data analysis and visualization

Our analysis pool of cells [banc_888_meta.feather] is primarily neurons marked as proofread/roughly_proofread in CAVE [cell_representative_point.parquet]. However, we have included a number of optic lobe neurons marked as proofread/roughly_proofread since the v.888 snapshot on 17 April 2026, corresponding to v.890 of the dataset, but mapped to a v.888 identifier, and omitted some neurons later found to be cellular debris. All connectivity and influence analyses are based on their v.888 identifier (root_id) and v.888 v.2 synapse table [synapses_v2_human_readable.csv.gz].

For connectivity analyses, we did not apply a synaptic threshold, except when we analysed influence, for which we applied a threshold of ≥5, [influence/all_to_all/, influence_all_to_effector_subclass.parquet, influence_sensory_subclass_to_all.parquet].

In the example circuit diagrams depicting individual cells or cell types (‘circuit vignettes’), connections between nodes were all ≥10. In all these circuit vignettes, we depict only a small subset of the inputs and/or outputs of the neurons in question. Our vignettes focus mainly on direct connections, but it should be noted that indirect connections can also produce high influence values.

In Fig. 2 we compressed some sensory cell classes for display. Hemolymph includes aorta, neurohemal complex and retrocerebral complex; enteric includes digestive tract, crop and salivary glands; reproductive includes uterus and ovaries; neck includes prosternal organ; and pharynx includes pharynx and cibarium.

Visual projection neuron functions were used to name different visual information streams as sensors. This is an incomplete survey of visual functions bounded by the literature^65,194,200,284,301–314^. Visual projection neurons were assigned to functions as follows: visual chromatic, aMe12, MeTu3b, MeTu3c, MTe50; visual leg feedback, LT52; visual horizontal widefield motion, dCH, FD1, FD3, H1, LPT04_HST, LPT21, LPT22, LPT23, LPT26, LPT42_Nod4, Nod1, Nod2, Nod3 and vCH; visual large_objects and visual thin vertical bar, LC15; visual loom, LC16, LC4, LPLC1 and LPLC2; visual object and visual loom, LC12 and LC17; visual polarized light, MeMe_e10, MeTu2a, MeTu2b and MeTu3a; visual small_object, LC10a, LC10b, LC10c, LC10d, LC11, LC13, LC18 and LC21; visual small object and visual_loom, LC26, LC6, LC9 and LPLC2; visual thin vertical bar, LC25 and MeTu1; and visual vertical widefield motion, LPT27, LPT28, LPT30, LPT31, LPT45_dCal1, LPT47 vCal2, LPT48 vCal3, LPT49, LPT50, Nod5, V1, vCal1, VST1 and VST2.

From Fig. 6g, the top AN/DN cell types over threshold were: for central complex output, DNa03, DNpe016, DNb01, AN17B008, AN18B053, DNbe006, DNg04, AN07B045, DNa02, DNg36_a, DNb09, DNpe019, DNg01, DNg71, DNg97, DNa13, DNae001, DNg106, DNae002 and AN23B004; for mushroom body output, DNge150, DNg104, DNge138, DNge151, DNp42, DNp68, DNge149, DNge152, DNg66 and ANXXX977.

We conducted data analysis in R (v.4.2.1) using the uwot (v.0.1.14)^268^, tidyverse (v.1.3.2)^315^ and ggplot2 (v.4.0.0.9000)^316^ packages. We made the Kernel density estimates for Fig. 6a using MASS::kde2d (v.7.3.58.1), *n* = 100, cubes with densities above the first percentile coloured^317^. We calculated cosine similarity using the lsa (v.0.73.3) R package^318^, and we applied it to direct connectivity between BANC neurons to build the space used in Fig. 3. To perform the Kolmogorov–Smirnov, two-way ANOVA tests and Kruskal–Wallis tests, we used their respective statistical functions using the car (v.3.1.2) and stats (v.0.62.0) packages in R. *P* values for nonparametric Wilcoxon rank-sum and signed-rank tests were obtained using the wilcox.test function in base R. Holm correction alpha values were all set to 0.05. *P* values were as follows: ****P ≤ 0.0001, ***P ≤ 0.001, **P ≤ 0.01, *P ≤ 0.05 and not significant (NS, *P* > 0.05). Because the connectome has a large number of neurons and connections, standard statistical tests often report very small *P* values; we frequently report *P* < 2.2 × 10^–16^, which reflects the lower numerical bound of double-precision calculations in R. We used large language models (LLMs), including Claude Code (Opus v.4.7, 2.1.147) and the Harvard AI Sandbox (https://www.huit.harvard.edu/ai-sandbox, accessed 2025–2026), to review and recommend code as well as to write a first draft of the verbal code documentation for human review and editing. All LLM output was evaluated for accuracy, in compliance with the Harvard University Generative AI guidelines (https://www.huit.harvard.edu/ai/guidelines). The Harvard AI Sandbox provides a secure environment in which to use LLMs, and all queries are recorded. The majority of our codebase was not assisted by LLMs.

### Reporting summary

Further information on research design is available in the Nature Portfolio Reporting Summary linked to this article.

## Online content

Any methods, additional references, Nature Portfolio reporting summaries, source data, extended data, supplementary information, acknowledgements, peer review information; details of author contributions and competing interests; and statements of data and code availability are available at 10.1038/s41586-026-10735-w.

## Supplementary information

Supplementary Information Table of resources generated by this work, tables of neuroglancer (for neuroanatomy) and FlyWire Codex (for network diagrams and annotation) links, and detailed description of the columns of each of the Supplementary Data files.

Reporting Summary

Supplementary Data 1 Metadata categories and terms. Table of categories of annotations applied to BANC neurons and the list of terms used in each category.

Supplementary Data 2 BANC neuron metadata. Per-neuron metadata for the proofread/roughly proofread neurons in our BANC release, with cross-dataset match identifiers to FAFB, MANC, hemibrain, FANC and maleCNS where assigned. This is the reference table for every BANC analysis in the paper.

Supplementary Data 3 Updated FAFB-FlyWire neuron metadata. Per-neuron metadata for the FAFB-FlyWire v.783 neurons used in BANC's cross-dataset comparisons. We re-annotated several columns (super_class, cell_class, cell_sub_class, body_part_*, cell_function) to follow BANC conventions; cell_type names are unchanged from the FAFB project.

Supplementary Data 4 Updated MANC neuron metadata. Per-neuron metadata for the 49,052 VNC neurons from MANC v.1.2.1, with super_class / cell_class / cell_sub_class / body_part_* / cell_function aligned to BANC conventions. cell_type names are unchanged from the MANC project.

Supplementary Data 5 maleCNS neuron metadata. Per-neuron metadata for the neurons in the male CNS v.0.9 release. Cell typing is all from the maleCNS project, but annotations have been aligned to the conventions of BANC, to help users compare and co-analyse these datasets.

Supplementary Data 6 ANs and DNs with PCA–UMAP coordinates and cluster assignments. Per-neuron metadata for the ANs and DNs included in the connectivity–PCA–UMAP analysis (Fig. 3d and Extended Data Fig. 6), with cluster assignments.

Supplementary Data 7 Effector neurons with UMAP coordinates and functional cluster assignments. Per-neuron metadata for the efferent neurons (motor + visceral/circulatory) in the connectivity–UMAP analysis (Extended Data Fig. 4f), with cluster assignments.

Supplementary Data 8 CNS network analysis with spectral clustering and UMAP embedding. Per-neuron metadata for the 50,568 intrinsic neurons that entered the CNS-network spectral clustering analysis (Fig. 6a and Extended Data Fig. 10). Spectral clustering parameters: min connection strength = 1, cluster count = 13, cluster seed = 10, embedding seed = 3 (banc_888).

Supplementary Data 9 Literature review on cell function for ascending, descending and visual projection neurons. Curated list of literature-validated functional roles used to interpret the AN/DN clusters and visual-projection influences

Supplementary Data 10 Bounding boxes for known dataset artefacts that negatively affect neuronal reconstruction. In total, 22 bounding boxes delineating regions with known data-quality issues in the BANC dataset. Coordinates are in BANC raw-voxel space (1 voxel = 4 × 4 × 45 nm).

Peer Review File

## Data Availability

Data are freely accessible through multiple platforms. A general overview of the resource and links to these tools are available at the BANC portal (https://banc.community). FlyWire Codex^319^ (https://codex.flywire.ai/banc) provides an interactive web interface for exploring the BANC connectome, which enables users to search for neurons, visualize morphology, traverse synaptic pathways and download metadata such as cell-type annotations, neurotransmitter predictions and connectivity matrices. Volumetric EM data, including 3D neuron meshes and annotations, can be viewed at the BANC portal (https://ng.banc.community/view) or accessed programmatically via CAVE^28^. We used CAVE materialization v.626 (21 July 2025) for our preprint, and v.888 (17 April 2026) for the final print version. Static data and code dumps are also available for download from a DOI-minting repository, the Harvard Dataverse (10.7910/DVN/7WTH1N) and on a Google Storage Bucket (console.cloud.google.com/storage/browser/lee-lab_brain-and-nerve-cord-fly-connectome/). Note that this Harvard Dataverse version supersedes our previous Harvard Dataverse for the preprint^216^, v.626 (10.7910/DVN/8TFGGB). Direct downloads from the Harvard Dataverse and Google Storage Bucket include the following resources: the synaptic connectivity edgelist, NBLAST results of BANC neurons against hemibrain, FAFB, FANC and MANC as well as BANC all-by-all; neuronal L2 skeletons (made using: https://github.com/CAVEconnectome/pcg_skel); neuronal colorMIPs; influence scores; our aligned BANC metadata; template registrations; behavioural data for the BANC fly; and snapshots of our code. Ground-truth labels are available for neurotransmitters^210^ (10.5281/zenodo.20818141) and neuropeptides^211^ (10.5281/zenodo.20818143). Aligned image data and flat segmentation data for cells, nuclei and mitochondria are available on BossDB (DOI: 10.60533/boss-2025-941r, or URL: https://bossdb.org/project/bates_phelps_kim_yang2025). Schematics are available as vector graphics from GitHub (https://github.com/wilson-lab/schematics?tab=readme-ov-file). Please see Supplementary Information for a full list of the data and code resources we have made available, along with their web addresses.

## Code availability

A comprehensive collection of community tools and software packages for working with the BANC dataset can be found at the project hub (https://banc.community) and the FlyWire Apps portal (https://flywire.ai/apps). All code developed for this project is open-source, publicly available and archived on the Harvard Dataverse repository (10.7910/DVN/7WTH1N). This archive contains the following repositories, also hosted on GitHub or Zenodo: the specific code used to perform the analyses and generate the figures for this manuscript (BANC-Project, 10.5281/zenodo.20350642)^320^; our data pre-processing pipeline post-proofreading for BANC, including skeletonization, neuron matching pipeline, neuron matching algorithms, metadata management, axon–dendrite splitting, neuropil assignment to synapses, among others (bancpipeline, 10.5281/zenodo.20350572)^321^; the Python code for computing influence scores (Connectome Influence Calculator, 10.5281/zenodo.15999929)^42^; the R code for computing influence scores (influencer, 10.5281/zenodo.20350564)^322^; the code for training and running the neurotransmitter prediction model (synister_banc, 10.5281/zenodo.20350570)^323^; the Python client code for querying and analysing BANC (banc, https://pypi.org/project/banc/); an R client package compatible with the natverse^105^ for querying and analysing BANC (bancr, 10.5281/zenodo.20350648)^324^; an R package for neuroanatomical plots (nat.ggplot, 10.5281/zenodo.20350566)^325^; and a Python + R tutorial for fly connectome data analyses that brings many of these tools and data together (fly_connectome_data_tutorial, https://github.com/sjcabs/fly_connectome_data_tutorial). We have also made our association of published, verified neurotransmission with connectomic cell types available as citable resources (drosophila_neurotransmitters^210^, 10.5281/zenodo.20818141, and drosophila_neuropeptides^211^, 10.5281/zenodo.20818143).
