# Supplementary material for: Distributed control circuits across a brain-and-cord connectome
Source: Nature. 2026 Jun 8;656(8129):957–70. doi: 10.1038/s41586-026-10735-w (PMC13518251; doi:10.1038/s41586-026-10735-w)
Supplement: Supplementary file 1 [file 41586_2026_10735_MOESM13_ESM.pdf]

nature portfolio

Peer Review File

Distributed control circuits across a brain-and-cord connectome

Corresponding Author: Dr Wei-Chung Lee

Any redactions in this file are there to maintain patient confidentiality, the confidentiality of unpublished data, or to remove third-party material.

This file contains all reviewer reports in order by version, followed by all author rebuttals in order by version.

Attachments originally included by the reviewers as part of their assessment can be found at the end of this file.

Version 0:

Reviewer comments:

Referee #1

(Remarks to the Author)
Bates et al provide an exciting new connectomics dataset of an entire fruit fly brain and nerve cord. The authors make a strong effort to match the new dataset to existing connectomes and the wider literature on sensory and motor neurons external to the brain and VNC. They introduce a new algorithm to investigate long-range connectivity, which while not conceptually new, provides a new scalable alternative to existing algorithms. The data and analysis are clearly presented on Flywire and in their published code, making it easy to navigate and understand. The primary finding is that the CNS comprises local sensory-motor loops that are connected with weaker long-range connectivity. The role of ascending and descending neurons is proposed to be important for this long-range connectivity. This manuscript provides an extensive dataset with new conceptual findings of how nervous systems are structured. We have some concerns about quantification of various results and other minor issues, which will be mentioned below, but overall view the manuscript very favourably.

Comments:
Fig 1c: the fraction of proofread neurons is useful to know, but we also need to know the % of synapses contained in those proofread neurons, as seen on past connectome papers. The number of pre synapses are reported in the main text (~60%), however the much harder to reconstruct postsynaptic connections are not reported (~20%). This should also be mentioned in the main text and not just in Extended Data Fig 1e. Extended Data Fig 1d would also be useful to have in the main Figure 1, as it is more indicative of completeness of proofreading than Fig 1c.

The authors should show data to support this statement from line 144-146: “Our identifications of neurons releasing acetylcholine, glutamate, GABA, dopamine, serotonin and octopamine largely agree with previous predictions”. This could be something like the percentage overlap of known/cross-matched cells with the same predicted neurotransmitter type for example.

There is an issue with supplemental data table 1, the rows seem to be in the wrong order and missing data, e.g. in the fafb_match_id column, the only entry is [FAFB ID], and there is no column for the BANC root/body ID.

Why did you only normalise some of the influence scores? The deciding factors for this should be clearly outlined.

In line 214 you mention ‘influence’ being a shorthand for high adjusted influence score. Extended Data Fig 2d uses a cutoff of 17.18, Fig 5a seems to use a threshold around 20.5 for the associated diagrams (based on connections included in Fig 5b). The explanation for the Ext Fig 2d cutoff is given in a note in the figure legend of Ext Data Fig 2, however the elbow plot used to determine threshold is not shown. The adjusted influence metric threshold should be explicitly mentioned and justified each time a new threshold is used.

Lines 230-231: “effector cells receive their strongest influence from sensors in the same body part”. The evidence of this is a heat map (Fig2E), which does seem to agree with the statement. However, there is no quantification of this statement across

all sensory-motor pairings. Additionally, there are clearly some sensory modalities that do not form local feedback loops, but something more complex (abdomen, hind leg, hemolymph, body chordotonal…). These should be quantified and discussed as well.

Lines 238-239: “each local loop is influenced by a select group of more distant sensors”. This is again the same heatmap, additional quantification should be provided. It would be interesting to see how many long-range connections influence each local loop.

How do the sensory to effector long range connections relate to the sensory to DN/AN connectivity?

Related to lines 377-380, could the fact that most superclusters can be associated with a specific behavioural task be due to the cells having been clustered/divided based on their connectivity with known cells (most of which are involved in specific behaviours)?

Related to Figs. 3-4; Lines 382-383: “DNs and ANs can be divided into superclusters…[but] their inputs and outputs are specialized”. This concept needs quantification. The authors show an illustrative example using two heat maps showing the inputs and outputs of all ANs/DNs of that supercluster. But this needs conclusive quantification against some null model to demonstrate that these inputs and outputs are specialised. At a glance, they look quite similar overall (particularly the output, see Figure. 4C). Once some specialisation metric is developed, it should be applied to all superclusters to see if some are more or less specialised than others.

In Extended Data Fig 3a, how do the coloured groups relate to the DN/AN sensor and effector clusters? We also suspect there may be some neurons missing, as we could not identify any neurons in the ‘flight steering 1’ category based on their colour. Maybe rather than colours (or in addition), the supercluster groups could be numbered.

Fig.5A is a nice overview of the influence-connectivity of AN/DN superclusters, but what about direct connectivity? Do certain superclusters have direct connections to each other?

Fig.5D is a nice schematic of what might be happening, but there needs to be more conclusive evidence of such a structure than a few examples of similar architecture. It would be helpful to try quantifying examples of these types of subsumption structures in the data itself to determine whether this is indeed a general principle or something that happens to happen in a couple examples.

Fig. 6F: the membership of AN/DN clusters in CNS clusters does not demonstrate that ANs/DNs are bridging CNS networks. A more direct way of testing this would be to excise ANs/DNs from the network and see if influence scores are reduced between CNS networks. This would also help strengthen the argument presented on lines 589-590.

It would be useful to show data to support statements made in lines 865-868 regarding the error rate of cell type label assignment.

Stürner et al 2024 (doi: 10.1101/2024.06.04.596633) and Winding et al 2023 (doi: 10.1126/science.add9330) both report connectivity between ANs and DNs and suggest this connectivity may be important for behavioural control. How does this compare or relate to the findings in this manuscript?

References to unpublished work:

Lines 873-876: Claims about the significance/importance of a different manuscript in preparation should be removed, as they cannot be supported by the data presented here.

Line 129: What does this in prep manuscript contribute specifically? This should be clearly described.

(Remarks on code availability)
The GitHub repo was well structured and easy to navigate. We installed and ran the influence metric code without issues.

Referee #2

(Remarks to the Author)
I co-reviewed this manuscript with one of the reviewers who provided the listed reports.

(Remarks on code availability)
Repository on Github is well organised and easy to navigate. I only tried to run the adjusted information metric analysis but this worked well.

Referee #3

(Remarks to the Author)
A. Summary of the key results
This study provides the first full EM connectomics dataset that combines the brain and the ventral nerve cord (VNC) and thus

entails the whole CNS. Moreover, the CNS is not reconstructed in isolation, but it is embodied by having identified numerous sensory cells in the periphery with their projections and connections in the CNS, and by assigning the motor neurons to their target muscles as well as by identifying and annotating numerous endocrine cells in the CNS with their specific visceral targets and neurohemal release sites. Therefore, this new connectome provides a yet unprecedented resource of a full CNS connectome that is highly embodied. It is a prime example of open science and excellent user accessibility, and it is well integrated with the already existing connectomics datasets.

In a next step, a heroic effort is taken to create a matrix that estimates the network influence of any one cell to any other cell, for all cells in the connectome. It is a highly impressive accomplishment to provide a network distance metric (named adjusted influence) for all possible neuron pairs (which yields 24 billion scores freely available at codex.flywire.ai/banc), and the adjusted influence scores presented here match other measures of network distances, but it does not consider synapse type and other highly important network features (see below).

This adjusted influence score is then used to make predictions on principles of the neural control of behavior. Here the key results are that sensory cells from one body part feedback to motor control for the same body part (essentially meaning that wing sensors are used for wing control), that local feedback circuits are linked by long range circuits into behavior centric modules (long range flight control circuits are closer linked to short range flight than to walking circuits), that descending and ascending neurons often influence voluntary movements of multiple body parts, and that brain regions involved in cognition have less direct influence on motor control, and are thus named supervisory. In sum, these data confirm previous work showing a distributed and parallelized control architecture.

B. Originality and significance: if not novel, please include reference
This embodied full CNS connectome will be extremely useful for the scientific community. First, it really is the first connectome that connects the brain and the VNC networks and thus provides high quality novel tools for studies on information transfer between the brain and the VNC. Second, its embodiment provides novel approaches toward addressing the principles of the neural control of behavior. Third, given the efforts that have been done to include modifications of the typology annotations for the whole-brain and the VNC connectomes, comparisons between the existing Drosophila connectomics datasets and the new BANC presented here are strongly facilitated. This is a strong step towards allowing inter-animal comparisons in connectomics datasets, which is further supported by the superb availability of all Drosophila connectomes and the publicly available tools to study them.

Providing a matrix with 24 billion adjusted influence scores for all combinations of neuronal connections throughout CNS networks is indeed a novel scale and might be highly useful for many aspects of network analyses, but it has significant limitations.
Accordingly, the interpretations that are drawn from employing this tool to the BANC connectome seem not all that novel. For example, the conclusion of a distributed network architecture for insect locomotion control has been reviewed already nearly 20 years ago (Cruse et al., 2007, Insect walking is based on a decentralized architecture revealing a simple and robust controller, doi: 10.1098/rsta.2006.1913; Bueschges, 2005, Sensory control and organization of neural networks mediating coordination of multisegmental organs for locomotion doi: 10.1152/jn.00615.2004) as well as recently (Bueschges, Ache, 2025, Motor control on the move: from insights in insects to general mechanisms. Physiol Rev doi: 10.1152/physrev.00009.2024). Moreover, the manuscript repeatedly claims to have resolved an old debate as to whether behavioral control works in a top-down manner, where actions are selected centrally and then relayed to lower regions for implementation, or whether behavioral control is distributed. However, it appears clear that distributed control is the case across phyla, with local sensory feedback and reflexes as well as local motor control circuits interacting with higher centers. In fact, the only paper that is cited for a sole top-down model with centrally selected actions is Fiore et al., (2015), but this paper is rather an evolutionary comparison of the basal ganglia and the central complex, but not a pledge for centralized behavioral control. In fact, citing from this paper speaks for distributed control: 'In a cockroach, for example, forward walking can be triggered by touching its abdomen, even after its brain has been disconnected from its ventral nerve cord. But the animal is unable to respond to novel external cues and change its direction'. Similarly, the notion that motor neurons are influenced by local sensory cells in the same body part (local feedback loops) is not novel (see Mantziaris et al., 2017, doi: 10.1152/jn.00321.2017 and citations above) but rather somewhat trivial, isn't it clear that leg motor neurons receive direct or indirect feedback from the leg and wing motor neurons from the wings? In sum, it is recommended to down-tone the novelty of the findings on network architecture but to provide a more critical evaluation what the connectome and the adjusted influence score can really answer.

C. Data & methodology: validity of approach, quality of data, quality of presentation
Although the adjusted influence score matches the results of other network distance measures that have previously been used, it has significant limitations that need to be considered and explained in more detail. First, it does not account for synapse type, not even for inhibitory synapses along the way. Second, it does not consider time, which would be important for differently long network distances along multiple paths between two neurons. This is circumvented by looking only at steady state, but in reality, the timing of synaptic inputs is critical for their summation. Third, it does neither consider synaptic diversity or plasticity. Some synapses have high release probabilities and depress whereas others have low release probabilities and facilitate. Many synapse activations along a path may be sub-threshold, which would cancel out any influence along that network path. Fifth, there are numerous postsynaptic mechanisms how to compute and modify synaptic input, including amplification, multiplication, and summation, which again depend on timing. It must be clear to the reader of a broad audience that adjusted influence does not account for any of this and is thus a vast simplification that ignores numerous critical aspects of information transfer in neuronal networks. This does not mean that it cannot be used, but these limits must be pointed out in the text. It is stated in line 218 that the influences are merely predictions to generate testable hypotheses, but it should be clear that these predictions are based on very limited information and can also be false and

misguiding. This does not mean that adjusted influence may not be of high value for future research, but it should be presented more carefully. The credibility of the adjusted influence score could be increased by experiments (see below).
The voxel size of the connectome is 4x4x45 nm, meaning that the x and y resolution is great (twice as large as compared to the MANC), but the z resolution is 5-6 times lower than that of the MANC dataset, and thus exceeding the width of the synaptic cleft by far. How did this affect synapse detection and transmitter class prediction? The scheme used to train and use a 3D convoluted neural network is well described on page 26, and the authors have to be commended for making a fully cited compilation of ground truth labels for each cell type freely available (line 823), but it is important to have an estimate of the consequences of the relatively low z-resolution.
Although the combination of brain and VNC with fully intact head connectives plus mostly intact nerve roots that allow for embodiment are major accomplishments, and the annotation of this dataset must have been an incredible endeavor, there are some parts missing from the brain (such as the lamina and the retina are missing from the optic lobe). This is likely owed to the challenge of having entirely intact connectives to really map all DNs and ANs, and it is mentioned in the methods section, but it should also be made transparent in the main text (e.g. almost complete brain plus VNC connectome). Also, the preparation used looks bent and somewhat distorted (Fig. 1). This seems a pity considering that a total of roughly 30 working years have been invested by more than 150 people for proofreading. The reason for that choice should be explained in the text.
Data presentation: Overall, the figures are well organized and clearly presented. Given the nature of the data, it is clear that the lettering becomes very small and hard to read. If possible at all, it is suggested to increase letter size wherever possible. Some specific suggestions for the figures are:

Figure 1 should also contain a 3-D reconstructed neuron from the BANC dataset with synapses to give the reader a clear visual impression of the resolution in all 3 spatial dimensions.

Figure 2: panel a on the right should not refer to source and target activity. If I understood correctly, neuronal activity was neither recorded nor simulated as membrane potential, but relative input synapse numbers were counted per layer along a network. The current representation gives the reader the impression that each network path was really subject to computational modeling of neuronal activity. The legend to panel b must explain that the relation is linear because the y-axis shows log data to make it linear over layer number. It must also explain that the score starts at 24 in layer zero (at the source) because 24 was added to all values. Panel e should not be on a normalized scale but show real adjusted influence score, so that e can be directly related to panels a to c. Also, panels d and e (belonging together) take a lot of figure space just to show that sensors from specific body parts influence mainly the effectors of these body parts, such that wing sensors influence wing effectors whereas leg sensors influence leg effectors.

Figure 3 nicely explains the concept of DN and AN superclusters, though these results do not come as a surprise. The data presentation is difficult to follow due to the small size, but this again is likely owed to the type of analysis presented. Any way to increase font sizes would be nice but is not required. A problem with the figure and accompanying text that must be addressed is that the reader may get the impression that the main input partners of motor neurons are DNs and ANs, but this is not the case. For example, wing power motor neurons as shown in panel h receive most of their direct input from local (VNC intrinsic) neurons (nearly 70%), but not from DNg27 (less than 10%) as might be suggested by the figure plus text. Moreover, in the BANC dataset only 5% of DNg27 output is to the depressor flight motor neurons 1 to 4, but 15% of the DNg27output is to only two VNC intrinsic interneurons. This is important, because VNC intrinsic interneurons seem to be the major premotor neurons in insects and are mostly neglected in the description of functional circuit architecture presented here. This should be clarified in the text.

Figure 4 exemplifies specializations and coordination within functional superclusters. It would be great if some experimental testing for the functional predictions shown in d and in e could be provided. Can specific predictable head positions not be taken when identified DNs or ANs that are specialized for these positions are acutely silenced? Can the animal not get into its normal standing position when silencing the negative feedback loop via AN06B002 after landing?

Figure 5 proposes interactions between behavior centric modules. Panel c exemplifies interactions between the threat response, the walking, and the proprioceptive superclusters to draw a picture that threat signals are integrated to stabilize defense posture while inhibiting walking drive. This seems intuitive and can be observed behaviorally (unless escape is evoked), but can these circuit motives really fully explain the behavior? What are additional features of the neurons and the circuits that should be considered to fully understand the behavior.

However, it might be more informative to investigate supercluster interactions that must interact in highly defined ways to produce stereotypical sequences of complex motor behaviors. A great example would be courtship, where walking, singing, licking, and copulation must all be arranged in a well-coordinated sequential manner in the context of processing different streams of sensory signals.

D. Appropriate use of statistics and treatment of uncertainties
Despite the uncertainties related to a broad validity and a broad applicability of the adjusted influence score and some uncertainties about the consequences of the relatively low z-resolution, the data are generated and presented on an exceptionally high level. There are no other concerns about statistics or uncertainties.

E. Conclusions: robustness, validity, reliability
The connectome is fantastic, well explained, superbly annotated, and a true prime example of open science that is expected to act as a cornerstone of many future research projects and novel insight into network architecture for behavioral control. The metric of influence provides an amazing look-up table for estimates of network distance that can be highly useful to form

hypotheses and make predictions for future research directions, but its validity and applicability remains quite limited at this point. The conclusions drawn from this metric of influence on network architecture are not entirely novel and only partly convincing.

F. Suggested improvements: experiments, data for possible revision
The adjusted influence score requires validation if employed that broadly.

G. References: appropriate credit to previous work?
The recent advances in connectomics analyses and associated tools and techniques as well as recent work on Drosophila functional circuit analyses are well credited. The text would greatly benefit from referring at least exemplarily to the large body of old work on network architecture for behavioral control that has described a decentralized and parallelized network structure, ANs with information transfer from the brain to the VNC and many other aspects shown here already decades ago. Similarly, a comparison to the network architecture and information flow between brain and spinal cord as it is known from the vertebrate CNS would be extremely helpful to integrate the wonderful advances deriving from Drosophila connectomics in a broader perspective.

H. Clarity and context: lucidity of abstract/summary, appropriateness of abstract, introduction and conclusions
Clarity and context of the abstract are appropriate and comprehensible. The same is true for the intro and conclusions. However, it is recommended to take older work into account and downtone occasionally (see above and below).

Line 255 states that it is sometimes suggested that DNs send motor commands to the VNC whereas ANs send sensory signals and predictive motor signals back to the brain. Please clarify what is meant by predictive motor signals. Also, again this seems like a somewhat constructed debate. It is clear since more than 30 years that ascending neurons can also transfer sensory signals from the insect brain to the VNC (e.g. the locust A4I1 neuron with its cell body in the fourth abdominal ganglion transfers mechanosensory information from the head to the thoracic flight motor circuits, Burrows and Pflüger, 1992, Output connections of a wind sensitive interneurone with motor neurones innervating flight steering muscles in the locust. doi: 10.1007/BF00194576, or even older Pflueger, 1984, The large fourth abdominal intersegmental interneuron: a new type of wind-sensitive ventral cord interneuron in locusts, doi: 10.1002/cne.902220303). This does not at all decrease the great value that this paper now adds all ANs and DNs with their putative input and output synapses to a publicly available database, which is really fantastic. But the reader should not get the impression that this resolved a debate of whether ascending neurons send information only from the VNC to the brain or also in the opposite direction.

As already mentioned above, the reader may get the impression that DNs are a major direct input to motor neurons.

Discussion:
Third para: A major point in the discussion claims to have resolved an old debate as to whether behavioral control works in a top-down manner, where actions are selected centrally and then relayed to lower regions for implementation or whether behavioral control is distributed. This reads like an artificial debate because it appears clear that distributed control is the case across phyla, with local sensory feedback and reflexes as well as local motor control circuits interacting with higher centers. In fact, the only paper that is cited for a sole top-down model with centrally selected actions is Fiore et al., (2015), but this paper is rather an evolutionary comparison of the basal ganglia and the central complex, but not a pledge for centralized behavioral control. Citing from this paper speaks for distributed control: ‘In a cockroach, for example, forward walking can be triggered by touching its abdomen, even after its brain has been disconnected from its ventral nerve cord. But the animal is unable to respond to novel external cues and change its direction’. It is thus suggested to remove the claim that a decentralized architecture for behavioral control is a novel finding (mentioned this already above).

Similarly, the claim that effector cells, such as motor neurons are primarily controlled by local sensory feedback seems not quite correct and misleading. First, the direct synaptic input to motor neurons comes mostly from local interneurons (here named VNC intrinsic neurons, which are mostly dismissed throughout the manuscript) and not from sensory neurons. For example, for flight power motor neurons, 10 % of the synaptic input is from sensory neurons, but roughly 70% of the direct synaptic input is from VNC intrinsic neurons (according to the MANC EM dataset). Second, patterned motor neuron activity can be generated by local central pattern generating networks without sensory feedback. Of course, in insect walking, to coordinate activity across joints and segments sensory feedback provides critical feedback about load, position, and movement, thus shaping the timing and strength of motor neuron bursts and ensuring adaptive coordination between joints (in pat also already mentioned above).

Fourth para: Suggests that combinatorial codes (across fiber patterns) fine-tune action patterns. Although the example that different threat DNs mediate different threat responses nicely reflects different actions being mediated by different descending neurons, there is no proof in the current data that combinatorial codes of DN activity fine-tune behavior. Again, this notion dismisses the importance of local (VNC intrinsic) interneurons that are the main input partners to motor neurons and themselves integrate input from sensory neurons and DNs. The picture drawn gives a somewhat misleading impression of motor control. It is even suggested on page 22, lines 579-581 ‘to think of DNs and ANs as “wires” for actuating effector cells in different combinations’. This would be in strict contradiction of local central pattern generating networks that cause coordinated activity motor neuron firing for each joint, and sensory feedback and intersegmental communication are needed for walking coordination. In stick insects, for example, coordinated motor neuron firing for leg movement is primarily generated by local CPGs for each leg joint (Bidaye et al., 2018, doi: 10.1152/jn.00658.2017; Strohmer et al., 2022, doi: 10.3389/finsc.2022.818449; Ruthe et al., 2024, doi: 10.1016/j.cub.2024.01.026), with joint- and segment-specific modulation. However, robust, adaptive coordination - especially across multiple legs - depends critically on sensory feedback and intersegmental communication (Mantziaris et al., 2017, doi: 10.1152/jn.00321.2017). This modular, feedback-driven system

enables flexible and stable walking patterns.

(Remarks on code availability)
All code is very well documented and freely available, a wonderful example of open science

Referee #4

(Remarks to the Author)
This landmark paper by Bates and colleagues provide the most comprehensive volume electron microscope dataset of an adult female Drosophila melanogaster nervous system to date, unique including the brain and the ventral nerve cord in a single volume. The authors focus on connectome modularity and the connectivity of ascending and descending neurons, fully reconstructed for the first time. One of the many findings of the study include the prevalence of local feedback loops with higher brain centres having supervisory functions. The paper will be a central resource for fly neurobiology and is of general interest and importance for circuit neuroscience.

Strengths:

The dataset is unique, of high quality and extensively annotated and allows the study of the connectivity between the brain and the ventral nerve cord. The data are made accessible on an online platform and the detailed and well-organised code enables the exploration of the connectome. The circuit analyses and functional predictions are compelling and will stimulate further experimental work. Besides the mapping of the whole connectome annotation, the dataset is also annotated with neurotransmitters, allowing to assign signs to the connections. The data are put into the context of the extensive literature on fly connectomics and the function of individual neuron types, e.g. ascending and descending neurons.

Weaknesses:

Some additional steps will be essential to guarantee the long-term archiving of the code and analysis workflows.
Since the dataset comprises a dissected adult brain, the identity of some of the sensory nerves remains ambiguous. Some other parts of the NS (e.g. ocelli) are also missing.

Essential revisions:

While the paper is exquisitely annotated and is made accessible to the community in an exemplary manner, the computational resources are only provided on GitHub. GitHub is not a permanent code archive. The authors should also link all GitHub repositories and permanently archive them and provide the DOIs in a long-term code/data archive, for example Zenodo. https://help.zenodo.org/docs/github/enable-repository/

Minor comments:

- It is sometimes unclear which of the influences or actions of the discussed neurons have already been tested experimentally and which are predicted based on the new connectome resource (e.g., page 11, lines 392f: DNa06 also targets two ANs that are positioned to excite neck and/or eye motor neurons.)

- Abstract, lines 64-67: What is referred to by the authors as „worms“ are indeed two representatives from two completely different animal groups (Ecdysozoa and Lophotrochozoa) and may be better referred to as ‘an annelid and a nematode’, otherwise it is misleading.

- The authors define effector cells as “(motor neurons, endocrine cells and efferent neurons targeting the viscera)"
In the zoological literature, the term ‘effector cell’ more commonly refers to muscles, gland cells, multiciliated cells (not in flies obviously), pigment cells, maybe endocrine cells. These are effectors because ultimately these cells 'effectuate' movements. These cells are obviously missing from the CNS connectome but many of them can be inferred (e.g. MN innervation). It would be better to stick to the more general terminology of motor neuron, endocrine cells and not lump these neurons together under effector cells.

- Page 3, lines 131: If inter-individual variability exceeding 25% it will be hard to generalize findings. It would be important to elaborate more on this and e.g. report whether these unmatched neurons are in a particular part of the nervous system (e.g. mushroom bodies), are due to technical or other differences. In this context, it would also be important to mention what is know about inter-sex variability.

- Page 11, lines 391: If there is any reference testing that these motor muscles/neurons control all three axes of movement it would be great to add this here.

Typos:

- Page 25, line 717: „removed“ is added twice in one sentence - „We removed the legs and proboscis removed to allow …“

- Extended Data Figure 1f: Typo in axis label - connections instead of connections; Different for sizes are used in the

respective panels; Could you possibly shortly add to Figure 1b which parts of the nervous system are targeted in the respective projects (MANC and FAFB are mentioned in the text, but what is the difference to hemibrain and FANC?)

- Extended Data Figure 2e: there seems to be a mismatch in the alignment of the labels and the bars – there are only 14 bar-comparisons, but 21 labels

- Extended Data Figure 3b: should this be „functionally assessed"?

(Remarks on code availability)
The code is very well organised and documented but needs to be also archived on a permanent repository.

Referee #5

(Remarks to the Author)
I co-reviewed this manuscript with one of the reviewers who provided the listed reports.

(Remarks on code availability)

Version 1:

Reviewer comments:

Referee #1

(Remarks to the Author)
We thank the authors for their thorough clarification and manuscript changes in response to our comments. The additional analyses and figures have addressed many of our comments and improved the manuscript. We do have some remaining concerns outlined below. Note that we include some of the author's original comments from the rebuttal letter for clarity, followed by our responses.

1. Proofreading status.
The figure and text edits regarding proofreading status of BANC has made it easier to assess BANC as a resource for the community, and we are happy with the changes the authors have implemented. However, at multiple points during the manuscript, the authors refer to BANC as one of the first 'complete' connectomes. We appreciate 'complete' in this context is refers to it being a full central nervous system, as opposed to completeness of the connectome itself. However, in the text, especially in the Abstract and Discussion lines 638-639, it is not immediately apparent that complete refers to CNS. We think the authors should be more specific here in referring to this connectome as a CNS connectome to avoid confusion with completeness of reconstruction (with BANC being less complete in terms of reconstruction compared to other available connectomes).

2. Adjusted influence metric.

Author's original comment:
For some analyses, having a principled threshold is useful. For example, to analyze numbers of 'body parts' influenced by ANs and DNs, we used ANs/DNs which have demonstrated effects on specific body parts (Extended Data Figure 5). Based on established published experimental data (e.g. optogenetic stimulation), we determined an adjusted influence of 17.15 (17.18 in the previous version; the change is due to an updated cell type label among our selected ANs/DNs) as a threshold for individual ANs/DNs influencing individual body parts (Extended Data Figure 5d,e). We think this is a very stringent threshold. We have now added the previously held-back elbow plot as Extended Data Fig. 5e. For our analyses on influence between larger pools of intrinsic neurons, i.e. between superclusters of ANs/DNs, we do not have a similarly biologically principled way to choose a threshold. Therefore, for Fig. 5, we simply use an adjusted influence threshold of the 85th percentile of Fig. 5a to plot Fig. 5b, so that the diagram would be humanly interpretable: i.e. did not have too many lines, to avoid cognitively overloading the reader. The accompanying heatmap (Fig. 5a) provides the full data for the reader. We have now made our choice of threshold clear in the legend:

"Summary of the strongest adjusted influences between superclusters. Here, for clarity, we depict only those influences with an adjusted influence cut-off of at the 85th percentile, chosen for concise visual display, line weight binned by the adjusted influences fully shown in (a)."

For our new analysis of the relative influence of mushroom body and central complex on ANs and DNs (Fig. 6g), given sensory neurons provide a baseline for influence on intrinsic cells, we used a median influence value from sensory neurons (Fig. 6f) as a threshold for selecting cell types to include in Fig. 6g. The point of the analyses is to demonstrate that few cell types from these more "cognitive" regions have comparable influence to that from sensors, and so we feel here that this threshold is more appropriate.

Reviewer comment:

We appreciate that for each of these individual instances, the reason why each threshold was chosen is reasonable. However, adjusted influence is presented as a generally useful metric to enable comparison of relative influence in the paper. As such, we think it is confusing to choose 3 different ways of thresholding, while describing the reasons and thresholding metric is the figure legends. In addition, the methods section implies a general threshold was used, which is not the case.
We would recommend choosing a consistent method of thresholding across the manuscript.
Otherwise, it should be made clear to the reader that within context comparisons require comparing influence scores relatively. The methods used in the manuscript should be clearly described in text, explicitly mentioning the 3 different ways that threshold were determined for different analyses. This will also make the metric more useful for usage in different contexts and further analyses in the future.

3. Sensor-effector pairings based on Fig 2 and Ext. Fig. 4:

Author's original comment:
Thank you. Our statement only applies to body parts where sensor and effector pairing can be clearly made. For example, chordotonal organs of the body (Wheeler's organ, the metathoracic organ, etc) do not have any effector neurons local to their sensory neurons in the periphery. The claim we make here is that when sensors and effectors can be paired by gross body part, sensor influence flows mainly to its paired effector neurons. However, significant off-pair influence exists, which also motivates us to examine longer range connections.

We have updated Fig. 2e to make our point better, including making more explicit pairings, and to remove distracting information (making this clear in the legend). We now quantify and statistically test that the interaction between sensory neurons and effector neurons is significant with a two-way ANOVA. We have also added a new analysis in Fig. 2g, which shows that neurons upstream of effector neurons in the CNS form local modules by influence, but that one step further, influences become more distributed. We use this observation to help motivate our subsequent focus on long-ranged connections (Fig. 2h) that reach across these modules.

Reviewer comment:
While the 2-way ANOVA for data shown in Fig 2e is useful to provide evidence for the general statement, it is difficult to evaluate this statement from a single sentence providing one p-value for a comparison of 110 x 23 subclasses. The data used for the statistical comparison and a post-hoc comparison of each group should be shown to evaluate whether this is generally the case for all pairings or whether there is a more heterogeneous effect.

Data in Fig 2g is an interesting addition, however as for 2e it is difficult to evaluate this statement based on a heatmap and an overall ANOVA value. In addition, the statement made in text about influence becoming more distributed is not tested. The ANOVA result for Fig 2g only suggests there is a source x target interaction effect across the whole data set and does not specifically compare effectors to pre- or pre-pre-effectors. This comparison, and specifically the claim that influence becomes more distributed should be properly quantified.

Author's original comment:
Thank you for the suggestion. We feel that an intuitive way to do this would be to assign sensor-effector connections that must transit between ganglia (i.e. between the brain and VNC) as 'long-ranged', those within-ganglia (within either the brain or the VNC alone) as 'medium ranged', and those for which the sensors and the effectors are in the same body part as 'short ranged'. We have now provided this new analysis, Extended Data Fig. 4b, which quantifies the proportion of the total sensory influence onto each efferent neuron group (by body part) that falls into these three classes.

Reviewer comment:
This is a useful addition. However, the statement 'each local loop is influenced by a select group of more distant sensors in functionally related body parts (Fig. 2e, Extended Data Fig 4b)' is not sufficiently backed up by Fig 2e or Ext Data Fig 4b. Ext Data Figure 4b does not enable evaluation of whether more distant sensor are functionally related, and Fig 2e does not contain quantification. Especially given the size of the dataset and the fact that the heatmap shows an aggregate value across many sensors, it would be useful to see whether there is a statistically significant influence from more distant sensors onto some of the effectors.

Author original comment:
We show that the core elements of behavioral control are a set of local feedback modules, where effector neurons are strongly and specifically influenced by the sensors positioned to monitor the relevant effectors (Fig. 2e). We have now added a statistical test to the Fig. 2e legend to show that the pattern of sensor→effector influence is significantly different for each body part. This argues that sensory signals are unlikely to be routed through a centralized world representation. This result is compatible with both ecological theory and embodied cognition, but not with the classical theory. In a new analysis, we show that there are strong local connections between effector pathways (Fig. 2g). Note that the pattern of recurrent effector pathway influence is significantly different for each body part (Fig. 2g legend). The prominence of local effector connections further supports the embodied cognition model of neural function.

Reviewer comment:
As noted above, the statistics provided in the legend of Fig 2e and 2g are not sufficient to back up the statement that sensor

to effector influence is significantly different for each body part.

4. AN/DN betweenness shown in Fig 3a

Author original comment:
In order to address this, we have conducted a new analysis on the relationship between ANs/DNs and sensory neuron-to-effector neuron indirect connectivity. Essentially, we quantify "bottleneck-ness" of all neurons, including ANs/DNs with node betweenness centrality (Freeman, 1977; Brandes, 2001; Brandes, 2008; Newman, 2018): for a neuron, its score is the fraction of all shortest paths between designated sources and targets that pass through that node. In our use, sources are sensory neurons and targets are effector neurons, so the score reflects how much a neuron could mediate sensor-to-effector communication. High-betweenness neurons are bridges linking otherwise separate modules. Critically, a node can have low degree but high betweenness if it sits between communities. I.e. this metric captures a neuron's potential control over global flow, not just its local connectivity. Fig. 3a now shows the result at a super-class level suggesting ANs and DNs are bottlenecks for sensory-to-effector information flow. Extended Data Fig. 4c also shows this extended across all proofread neurons as sources and targets, for which ANs and DNs remain among the most substantial bottlenecks for information flow in the CNS. We describe these new analyses in the Results:

"Thus far, we have seen evidence for strong local feedback loops, linked by selective longer-range sensor-effector connections. To better understand these long-range connections, we focused on the neurons that link the brain with the VNC, namely DNs and ANs. DNs and ANs participate in many sensor-to-effector connections: we can see this by finding the shortest paths from each sensory neuron to each effector neuron, and then computing the proportion of those paths that contain a given cell of interest. This value is relatively high for DNs and ANs (Fig. 3a, Extended Data Fig. 4c), which implies that DNs and ANs play key roles in bridging the brain and VNC to connect sensory neurons and with effector neurons.

Reviewer comment:
The legend of Figure 3a implies ANs and DNs had higher betweenness than all other super-classes but no direct comparison is shown. It is not clear from this test whether ANs and DNs have higher betweenness than other VNC intrinsic cells and the plot shows that they are at least comparable in value. This statistical analysis should be added and shown here.
The evidence provided here gives me reasonable confidence that ANs and DNs participate in many sensor-to-effector connections, however it should be noted that this also appears to be the case for other VNC intrinsic cell types.

5. Superclusters in Fig 4d

Author original comment:
Thank you for the helpful suggestion. We have quantified the cosine similarity of influence to and from ANs and DNs and tested input and output specialization by comparing influence related to ANs and DNs in the same cell type (highly similar), the same supercluster (less similar), and in different superclusters (dissimilar) in Fig. 4d. These results underscore that cells in the same supercluster are indeed diverse – more diverse than cells of the same type. At the same time, these results support the conclusion that cells of the same supercluster are more similar than cells of different superclusters.

Reviewer comment:
The additional quantification is useful. However, it is difficult to evaluate how similar or dissimilar some superclusters are given one summary comparison of all superclusters. It would also be useful to also show this comparison for each supercluster to provide some data on whether some superclusters may constitute a specific subcluster with higher confidence.

6. CNS networks in Fig 6

Author original comment:
Considering the network structure of the entire CNS, we found that many CNS networks have a high influence on effector neurons (Fig. 6b), further supporting the idea that behavioral control is distributed, rather than centralized. We found that the central complex and mushroom body have low effector influence overall (Fig. 6b), and high influence on only a small subset of DNs and ANs (Fig. 6f,g). Together, these findings imply that the central complex and mushroom body have a supervisory role, and they supervise a subset of behaviors, not all behaviors. This conclusion is compatible with the idea of embodied cognition (which posits a limited role for memories and other internal representations); it is also compatible with subsumption architecture, where high-level modules supervise low-level modules, but high-level modules are inessential for many behaviors. In short, our study provides many lines of evidence to support the idea of embodied cognition and specifically to support an implementation resembling subsumption architecture. Of course, it is possible that there is another theoretical framework that better explains the organization of the Drosophila CNS. If so, however, we think that theoretical framework does not yet exist. If the referee would like us to discuss another specific alternative, we would be sincerely interested in hearing this suggestion.

Reviewer comment:
For the comparison in 6f, it would be useful to also include the other CNS networks considered in 6b, for which a statement is made that they have high influence on effectors based on the heatmap in 6b, but this is not tested using a statistical test.

Author original comment:
We did not intend to argue that the bridges between CNS networks are solely composed of ANs/DNs, and we apologize if our original text gave this impression. We have revised the relevant sections of the main text to clarify our findings. Specifically, in the revised manuscript we state that:

“many CNS networks … contain ANs and DNs (Extended Data Fig. 10b-c)”

“these links [between CNS networks] are disproportionately composed of DNs: when we counted each neuron’s synaptic partners outside its assigned network, we found DNs had a relatively high proportion of outside partners (Fig. 6e)”

“Most AN/DN superclusters are divided between two or three CNS networks (Extended Data Fig. 10c), consistent with the notion that DNs often form bridges between networks.”

We think the cited analyses provide clear support for our conclusions as stated here. We did consider excising ANs/DNs to see how this changes influence scores. The main result is that the VNC-dominated CNS networks lose much of their influence from brain-dominated CNS networks, and vice versa. However, this result is somewhat expected, in our view, and we are hesitant to make much of it in the manuscript.

Reviewer comment:
It is not clear to me what ‘other’ in Figure 6e represents, this should be added to the figure legend. The text implies that DNs have a higher proportion of out-of-network direct partners. A two-sample KS test is not an appropriate test to make this claim in this case, as the distributions of each cell type are clearly different. This is also demonstrated by the significant difference between ANs and ‘other’ despite the seemingly similar median and IQR values. The authors should add a non-parametric test that compares the central value of the distributions to make this claim.

(Remarks on code availability)
We have not reviewed the code beyond what was mentioned in the first review

Referee #2

(Remarks to the Author)
I co-reviewed this manuscript with one of the reviewers who provided the listed reports.

(Remarks on code availability)

Version 3:

Reviewer comments:

Referee #1

(Remarks to the Author)
We thank the authors for their changes in response to our comments. We have no additional concerns and recommend publication.

(Remarks on code availability)

Referee #2

(Remarks to the Author)
I co-reviewed this manuscript with one of the reviewers who provided the listed reports.

(Remarks on code availability)

Open Access This Peer Review File is licensed under a Creative Commons Attribution 4.0 International License, which permits use, sharing, adaptation, distribution and reproduction in any medium or format, as long as you give appropriate credit to the original author(s) and the source, provide a link to the Creative Commons license, and indicate if changes were made.
In cases where reviewers are anonymous, credit should be given to 'Anonymous Referee' and the source.
The images or other third party material in this Peer Review File are included in the article's Creative Commons license, unless indicated otherwise in a credit line to the material. If material is not included in the article's Creative Commons license and your intended use is not permitted by statutory regulation or exceeds the permitted use, you will need to obtain permission directly from the copyright holder.
To view a copy of this license, visit https://creativecommons.org/licenses/by/4.0/

Response to Referees

We thank the referees for their constructive comments on our manuscript, “Distributed control circuits across a brain-and-cord connectome." We appreciate the time and effort that went into evaluating our work. We appreciate the referees' positive review of the manuscript and recognition of the importance of the study and the resource for the field. Referees recognized the importance of this full central nervous system (CNS) connectome and how our efforts to link this connectome to sensory and effector neurons embody the connectome, enabling a unique conceptual and analytical framework. The referees recognized the scale and importance of quantifying the influence of every neuron on every other neuron across the CNS. Referees were also very positive on the open-science nature of the data and tools provided by this effort; the integration with other available connectomes; and that it was straightforward to access and use the data, code, and resources overall.

Referees also raised concerns, which we summarize below, followed by a point-by-point response to the referees' comments. We have revised the manuscript, providing additional analyses and data to address concerns raised by the referees.

Summary of main concerns:

1. Discussion of previous work on centralized and distributed behavioral control and the importance of intrinsic VNC circuitry: We recognize that the manuscript did not clearly present the alternative frameworks of behavioral control nor emphasize the importance of intrinsic cells and circuitry for behavioral control beyond descending and ascending neurons (DNs and ANs). Regarding behavioral control, we recognize our original submission did not adequately describe the top-down model of control and alternative theories, and we have now revised the Discussion to fix this problem. Briefly, according to the classical theory, actions are selected by a centralized executive brain function. Low-level implementation of motor patterns could be delegated to circuits in the spinal cord (or VNC), but in the classical view, these motor patterns are still selected by descending commands from a centralized executive controller in the brain. In the revised Discussion, we now cite more papers that consider the classical theory of behavioral control in insects. We do not argue that this is the dominant theory; we simply argue that it is <u>a mainstream theory</u>. In the revised Discussion, we also review alternative relevant theories of behavioral control in the literature, namely the ecological theory and the theory of embodied cognition. We point out that our results are overall most supportive of the theory of embodied cognition. At the same time, we make it very clear that this idea is not novel to our study. Please see the Response to Referee 3, point #1 for details.

   There is certainly a deep literature on intrinsic circuits such as central pattern generators and additionally on distributed sensorimotor control, particularly in insects. We focused on ANs and DNs as they were a uniquely reconstructable aspect of the data. However, as with other connectomes, we also find that the majority of direct inputs onto motor neurons are local intrinsic neurons. We have revised the text in many places to clarify this issue (see Response to Referee 3, point #24 for details). Importantly, because the majority of direct inputs onto effector neurons are from local neurons, this was a major motivation for understanding the influence of neurons through indirect connections, including ANs and DNs and the focus of this study. This led to developing a scalable method of quantifying a network metric for influence (also discussed further below). Revising the manuscript, we have more broadly introduced and discussed previous findings on distributed and local circuits of behavioral control and have further contextualized our findings.
2. Interpreting influence scores: We realized that our influence scores require additional clarification for interpretability. Our goal for developing an influence metric was to better understand the potential impact of any neuron on any other neuron, given connectivity across the dataset. This was not meant to directly reflect neuronal activity nor dynamics. As referees point out, it is challenging to determine the net effect of multiple layers of inhibition and excitation across many connections and the timing of such inputs. Instead, we developed an efficiently computable network-based metric, we refer to as influence, which can be interpreted as a network distance. Because distance is always positive, it is sensible to interpret influence when it’s computed unsigned; however, we do also provide the code to compute a signed version of the influence score alongside the code for computing the unsigned version. We have attempted to clarify this in the main text and methods and also clarify adjusted and normalized influence and when they are used.

3. Additional quantification: Referees pointed out that additional analysis and quantification would be useful for some results, including subsumption architecture, sensorimotor loops, and AN/DN supercluster organization. We have included new analyses and statistical tests to support these results (e.g. **Fig. 2g, 4d, 6e-g, Extended Data Fig. 4b, 5a, 9**). We also include a new analysis supporting the idea that the neck connective acts as an information bottleneck (**Fig. 3a, Extended Data Fig. 4c**).
4. Reporting and improving the dataset: Referee 1 suggested additional ways of presenting dataset completeness. In particular, presenting the synaptic capture rate, which is the fraction of predicted synapses attached to both pre- and post-synaptic neurons that have been proofread. Previously we reported the presynaptic and postsynaptic capture rate separately. See updates to **Fig. 1c** (*bottom*) and **Extended Data Fig. 1b-d, f-g.** Notably, the recovered connected pairs of neurons continue to be highly correlated between the BANC, FlyWire, and MANC (**Extended Data Fig. 1f**). A new analysis shows that medium to strongly connected neurons are recovered with high likelihood in the BANC (**Extended Data Fig. 1g**). Additionally, we have added a new figure detailing neurotransmitter predictions, including the newly covered neurotransmitters, and comparisons across datasets (**Extended Data Fig. 2**). Finally, since the initial submission, we have continued improving and enriching the BANC. We have improved our coverage of neuron type annotations (**Fig. 1c**) and finished proofreading and attaching the 'orphaned' neuron fragments with 100+ synapses to proofread neurons, where possible, within the central brain and ventral nerve cord and are nearly finished proofreading the left optic lobe. .
5. Data and resource access: Referee 4 suggested that code and data be archived in a permanent repository. We agree and now make it even clearer that the data and code are archived in the Harvard Dataverse, which is a permanent DOI-minting and version-controlled repository (https://doi.org/10.7910/DVN/8TFGGB). Additionally, we have archived the image data and associated code at an NIH-funded repository https://bossdb.org/ (DOI: https://doi.org/10.60533/boss-2025-941r, or URL: https://bossdb.org/project/bates_phelps_kim_yang2025).

Point-by-point responses (in blue below):
Referee #1:

Bates et al provide an exciting new connectomics dataset of an entire fruit fly brain and nerve cord. The authors make a strong effort to match the new dataset to existing connectomes and the wider literature on sensory and motor neurons external to the brain and VNC. They introduce a new algorithm to investigate long-range connectivity, which while not conceptually new, provides a new scalable alternative to existing algorithms. The data and analysis are clearly presented on Flywire and in their published code, making it easy to navigate and understand. The primary finding is that the CNS comprises local sensory-motor loops that are connected with weaker long-range connectivity. The role of ascending and descending neurons is proposed to be important for this long-range connectivity. This manuscript provides an extensive dataset with new conceptual findings of how nervous systems are structured. We have some concerns about quantification of various results and other minor issues, which will be mentioned below, but overall view the manuscript very favourably.

Comments:
Fig 1c: the fraction of proofread neurons is useful to know, but we also need to know the % of synapses contained in those proofread neurons, as seen on past connectome papers. The number of pre synapses are reported in the main text (~60%), however the much harder to reconstruct postsynaptic connections are not reported (~20%). This should also be mentioned in the main text and not just in Extended Data Fig 1e. Extended Data Fig 1d would also be useful to have in the main Figure 1, as it is more indicative of completeness of proofreading than Fig 1c.

Response: We agree and thank the referee for this suggestion.

To make this clearer for readers, we have updated the Results as follows:

- As suggested by the Referee, we revamped **Fig. 1c** to show the fraction of predicted pre- and post-synapses (i.e. synaptic-links) contained in proofread neurons.
- We now provide quantified evaluation of precision, recall, and F1-scores of the post-synaptic predictions across different regions of the dataset (**Extended Data Fig. 1b**).
- We added the following sentence in the main text:
  *"Overall, 68% of detected presynaptic links and 22% of postsynaptic links have been attached to identified (proofread and/or cell-typed) cells; 15% of synapses have identified cells on both sides (**Fig. 1c, Extended Data Fig. 1b,c**)."*
- In the Synapse detection section of the Methods, we have also added these sentences:
  "It [the synapse detection] comprises 218460852 synaptic links, of which 68% of presynaptic ends and 22% of postsynaptic ends are connected to a proofread neuron. 15% of synaptic links have an identified neuron on both sides. For other densely reconstructed datasets, this number is: 41.9% for FAFB, 35.3% for HemiBrain, 44.4% for MANC and 40.1% for MaleCNS"
- In order to address whether BANC reliably captures the connections present in FAFB and MANC, we include a new analysis of the probability of recovering a cell-type-to-cell-type connection in BANC, given its presence in FAFB/MANC, at differing synapse counts (**Extended Data Fig 1g**) . This analysis shows that medium to strong connections are highly recoverable in BANC, consistent with the high correlation of connected neuron types between datasets (**Extended Data Fig 1f**).

Finally, we have improved the dataset since submission, as can be seen in **Fig. 1c**, including substantially improving our cell typing coverage.

The authors should show data to support this statement from line 144-146: "Our identifications of neurons releasing acetylcholine, glutamate, GABA, dopamine, serotonin and octopamine largely agree with previous predictions". This could be something like the percentage overlap of known/cross-matched cells with the same predicted neurotransmitter type for example.

Response: We thank the referee for this suggestion. In order to address this thoroughly and highlight important new predictions (histamine and tyramine are neurotransmitter predictions not published before), we

have created a new supplemental figure focused on neurotransmitter prediction, **Extended Data Fig 3**. This figure summarises our ground truth, training, and test results and reports predictions among BANC neurons that are proofread and have at least 50 output synaptic links.

Note, that the off-diagonal cell types in **Extended Data Fig 3e**, the confusion matrices against FAFB, the HemiBrain and MANC, do not indicate which dataset contains an error. Additionally, neurotransmitter prediction has historically performed better on serial-section TEM than FIB-SEM data. For example, per-synapse transmitter prediction accuracy was 87% on FAFB-FlyWire and 78% on HemiBrain and average accuracy of 94% for transmitter prediction per neuron on FAFB-FlyWire and 91% on HemiBrain) (Eckstein, Bates, et al. 2024).

There is an issue with supplemental data table 1, the rows seem to be in the wrong order and missing data, e.g. in the fafb_match_id column, the only entry is [FAFB ID], and there is no column for the BANC root/body ID.

Response: Thank you for identifying this. The issue is that we did not adequately explain what supplemental data table 1 shows, and we have now updated the legend to be clearer. To clarify, the rows do not mean anything. Only the columns are important, and each column contains the possible entries under that named category. For example, the 3 possible options for a neuron's flow are "efferent", "intrinsic", and "afferent"; the 3 possible options for a neuron's side are "left", "midline", and "right". However, although "efferent" and "left" are in the same row in the table, a neuron could have one, both, or neither of these labels. Regarding [FAFB ID], we included the column because fafb_783_match_id is one category of metadata we provide, but because there are over 100k unique identifiers for FAFB neurons, we did not list all of the ones that appear in the BANC metadata and just put [FAFB ID] as a placeholder. We have changed this to [integer 64], which then tells the reader what sort of ID to expect in these fields. The purpose of this file is to provide a human-readable file to list the metadata categories and to list the possible options in each metadata category (when that list is human-parsable), to help the user interpret our other metadata files. The full set of all annotations for all neurons in the dataset is available through Codex and through CAVE (codex_annotations table) and is not a supplementary data file.

Why did you only normalise some of the influence scores? The deciding factors for this should be clearly outlined.

Response: Thank you for the opportunity to make this clearer for readers. We generally use the adjusted influence score. In a few cases, we use the minmax 'normalized' score (**Fig. 2e**, **Extended Data Fig. 4e**, **Extended Data Fig. 6e-g**). We use the minmax normalized score in **Fig. 2e**: the point is to ask, for each group of effector neurons (rows), which sensor is most influential? In this context, it makes sense to normalize over the columns (sensors). The figure legend now states:

> *"Each row is normalized to the same range (0-1), to make clear which sensor groups are most influential for each effector group (14410 sensory neurons, 1031 effector neurons)."*

For the UMAPs in the Extended Data Figures, we are emphasizing the *relative* distribution of influences across the UMAP, and thus, normalization is appropriate. We have updated our figure legend for each use of the normalized score, to make our choice clear. For example, for **Extended Data Figs. 4e, 6e**, we have added:

> *"Minmax normalization rescales influence values to 0-1, across all AN/DNs. This illustrates the influence distribution rather than magnitude."*

In line 214 you mention 'influence' being a shorthand for high adjusted influence score. Extended Data Fig 2d uses a cutoff of 17.18, Fig 5a seems to use a threshold around 20.5 for the associated diagrams (based on connections included in Fig 5b). The explanation for the Ext Fig 2d cutoff is given in a note in the figure legend of Ext Data Fig 2, however the elbow plot used to determine threshold is not shown. The adjusted influence metric threshold should be explicitly mentioned and justified each time a new threshold is used.

Response: For some analyses, having a principled threshold is useful. For example, to analyze numbers of 'body parts' influenced by ANs and DNs, we used ANs/DNs which have demonstrated effects on specific body parts (**Extended Data Figure 5**). Based on established published experimental data (e.g. optogenetic stimulation), we determined an adjusted influence of 17.15 (17.18 in the previous version; the change is due to an updated cell type label among our selected ANs/DNs) as a threshold for individual ANs/DNs influencing individual body parts (**Extended Data Figure 5d,e**). We think this is a very stringent threshold. We have now added the previously held-back elbow plot as **Extended Data Fig. 5e**.

For our analyses on influence between larger pools of intrinsic neurons, i.e. between superclusters of ANs/DNs, we do not have a similarly biologically principled way to choose a threshold. Therefore, for **Fig. 5**, we simply use an adjusted influence threshold of the 85th percentile of **Fig. 5a** to plot **Fig. 5b,** so that the diagram would be humanly interpretable: i.e. did not have too many lines, to avoid cognitively overloading the reader. The accompanying heatmap (**Fig. 5a**) provides the full data for the reader. We have now made our choice of threshold clear in the legend:

> *"Summary of the strongest adjusted influences between superclusters. Here, for clarity, we depict only those influences with an adjusted influence cut-off of at the 85th percentile, chosen for concise visual display, line weight binned by the adjusted influences fully shown in (a)."*

For our new analysis of the relative influence of mushroom body and central complex on ANs and DNs (**Fig. 6g**), given sensory neurons provide a baseline for influence on intrinsic cells, we used a median influence value from sensory neurons (**Fig. 6f**) as a threshold for selecting cell types to include in **Fig. 6g**. The point of the analyses is to demonstrate that few cell types from these more "cognitive" regions have comparable influence to that from sensors, and so we feel here that this threshold is more appropriate.

Lines 230-231: "effector cells receive their strongest influence from sensors in the same body part". The evidence of this is a heat map (Fig2E), which does seem to agree with the statement. However, there is no quantification of this statement across all sensory-motor pairings. Additionally, there are clearly some sensory modalities that do not form local feedback loops, but something more complex (abdomen, hind leg, hemolymph, body chordotonal…). These should be quantified and discussed as well.

Response: Thank you. Our statement only applies to body parts where sensor and effector pairing can be clearly made. For example, chordotonal organs of the body (Wheeler's organ, the metathoracic organ, etc) do not have any effector neurons local to their sensory neurons in the periphery. The claim we make here is that when sensors and effectors can be paired by gross body part, sensor influence flows mainly to its paired effector neurons. However, significant off-pair influence exists, which also motivates us to examine longer range connections.

We have updated **Fig. 2e** to make our point better, including making more explicit pairings, and to remove distracting information (making this clear in the legend). We now quantify and statistically test that the interaction between sensory neurons and effector neurons is significant with a two-way ANOVA. We have also added a new analysis in **Fig. 2g**, which shows that neurons upstream of effector neurons in the CNS form local modules by influence, but that one step further, influences become more distributed. We use this observation to help motivate our subsequent focus on long-ranged connections (**Fig. 2h**) that reach across these modules.

Lines 238-239: "each local loop is influenced by a select group of more distant sensors". This is again the same heatmap, additional quantification should be provided. It would be interesting to see how many long-range connections influence each local loop.

Response: Thank you for the suggestion. We feel that an intuitive way to do this would be to assign sensor-effector connections that must transit between ganglia (i.e. between the brain and VNC) as 'long-ranged', those within-ganglia (within either the brain or the VNC alone) as 'medium ranged', and those for which the sensors and the effectors are in the same body part as 'short ranged'. We have now provided this new analysis, **Extended Data Fig. 4b**, which quantifies the proportion of the total sensory influence onto each

efferent neuron group (by body part) that falls into these three classes.

How do the sensory to effector long-range connections relate to the sensory to DN/AN connectivity?

Response: In order to address this, we have conducted a new analysis on the relationship between ANs/DNs and sensory neuron-to-effector neuron indirect connectivity. Essentially, we quantify "bottleneck-ness" of all neurons, including ANs/DNs with node betweenness centrality (Freeman, 1977; Brandes, 2001; Brandes, 2008; Newman, 2018): for a neuron, its score is the fraction of all shortest paths between designated sources and targets that pass through that node. In our use, sources are sensory neurons and targets are effector neurons, so the score reflects how much a neuron could mediate sensor-to-effector communication. High-betweenness neurons are bridges linking otherwise separate modules. Critically, a node can have low degree but high betweenness if it sits between communities. I.e. this metric captures a neuron's potential control over global flow, not just its local connectivity. **Fig. 3a** now shows the result at a super-class level suggesting ANs and DNs are bottlenecks for sensory-to-effector information flow. **Extended Data Fig. 4c** also shows this extended across all proofread neurons as sources and targets, for which ANs and DNs remain among the most substantial bottlenecks for information flow in the CNS.

We describe these new analyses in the Results:

> *"Thus far, we have seen evidence for strong local feedback loops, linked by selective longer-range sensor-effector connections. To better understand these long-range connections, we focused on the neurons that link the brain with the VNC, namely DNs and ANs. DNs and ANs participate in many sensor-to-effector connections: we can see this by finding the shortest paths from each sensory neuron to each effector neuron, and then computing the proportion of those paths that contain a given cell of interest. This value is relatively high for DNs and ANs (**Fig. 3a, Extended Data Fig. 4c**), which implies that DNs and ANs play key roles in bridging the brain and VNC to connect sensory neurons and with effector neurons."*

Related to lines 377-380, could the fact that most superclusters can be associated with a specific behavioural task be due to the cells having been clustered/divided based on their connectivity with known cells (most of which are involved in specific behaviours)?

Response: We apologize for any confusion here. In **Fig 3d**, we cluster ANs and DNs based solely on their direct connectivity without clustering or division by known cell types *a priori*. Thus, the position of each cell on the UMAP depends only on its direct synaptic inputs and outputs. We then aggregate these clusters into superclusters, and we assign tentative behavioral functions to each UMAP supercluster, based on three types of metadata: (1) the sensory influences onto the cells in that supercluster, (2) the influences of the cells in that supercluster onto effector neurons, and (3) the membership in that supercluster of any functionally characterized ANs/DNs. Presently, only about 11% of all ANs/DNs have been functionally characterized based on experimental data (physiological and/or behavioral data). We effectively use them as behavioral indicators or markers; however, they did not unduly influence clustering, that is to say their connectivity drove the UMAP clustering as much as any other AN/DN. We present in detail the supporting evidence for our assignments in **Extended Data Fig. 6** and **Extended Data Fig. 7**.

For clarity, we have revised the Results section describing this procedure:

> *"To identify functional divisions among DNs and ANs, we constructed a map of these neurons based on their direct synaptic connections, both pre- and postsynaptic (**Fig. 3d**). DNs and ANs are intermingled in this map because, as it turns out, their connections are often similar (**Extended Data Fig. 6a**). We verified that cells with similar known functions (11% of the total) are frequently colocalized on this map (**Extended Data Fig. 6b,c**; DNs and ANs with known functions are taken from previous work[9,20,21,24,79,100,116–146]). We then grouped related clusters of DNs and ANs into superclusters, based on the influence they receive from sensory neurons[21], as well as the influence they exert onto effector neurons (**Extended Data Figs. 6d-g,7,8**). Based on the sensory and motor influences associated with*

*each supercluster, as well as the functions of known DNs and ANs, we were able to link each supercluster with a putative behavior (**Fig. 3d**).”*

Related to Figs. 3-4; Lines 382-383: “DNs and ANs can be divided into superclusters…[but] their inputs and outputs are specialized”. This concept needs quantification. The authors show an illustrative example using two heat maps showing the inputs and outputs of all ANs/DNs of that supercluster. But this needs conclusive quantification against some null model to demonstrate that these inputs and outputs are specialised. At a glance, they look quite similar overall (particularly the output, see Figure. 4C). Once some specialisation metric is developed, it should be applied to all superclusters to see if some are more or less specialised than others.

Response: Thank you for the helpful suggestion. We have quantified the cosine similarity of influence to and from ANs and DNs and tested input and output specialization by comparing influence related to ANs and DNs in the same cell type (highly similar), the same supercluster (less similar), and in different superclusters (dissimilar) in **Fig. 4d**. These results underscore that cells in the same supercluster are indeed diverse -- more diverse than cells of the same type. At the same time, these results support the conclusion that cells of the same supercluster are more similar than cells of different superclusters.

In Extended Data Fig 3a, how do the coloured groups relate to the DN/AN sensor and effector clusters? We also suspect there may be some neurons missing, as we could not identify any neurons in the ‘flight steering 1’ category based on their colour. Maybe rather than colours (or in addition), the supercluster groups could be numbered.

Response: Thank you for catching this. The plot is now **Extended Data Fig. 6d**. The original used an incorrect color mapping. We have updated the plot and have also taken the referee’s suggestion and used numbering to map between clusters and superclusters.

Fig.5A is a nice overview of the influence-connectivity of AN/DN superclusters, but what about direct connectivity? Do certain superclusters have direct connections to each other?

Response: We have now supplied this information as **Extended Data Fig. 9a**. We have also supplied **Extended Data Fig. 9b**, which details the extent of AN-DN connectivity by identifying non-recursive linear ‘chains’ of AN-DN connectivity, and whether they transit between superclusters. We observe many inter-supercluster connections, but the influence scores give a fuller picture of inter-supercluster interactions.

Fig.5D is a nice schematic of what might be happening, but there needs to be more conclusive evidence of such a structure than a few examples of similar architecture. It would be helpful to try quantifying examples of these types of subsumption structures in the data itself to determine whether this is indeed a general principle or something that happens to happen in a couple examples.

Response: This is helpful feedback. In response to this comment, we have made several changes. First, we revised **Fig. 5d** so that it is less abstract and better connected to our data. Specifically, instead of using abstract modules, we now show an illustrative subset of the behavioral modules that emerged from our analyses, as well as the strongest hierarchical connections between these modules.

Next, we have heavily revised the Discussion to provide a more explicit framework and present several alternative theories for behavioral control, namely:

- According to the “classical” theory, actions are selected by a centralized executive brain function (sensing→cognition→action). Following this classical model, executive functions like action selection and attention have been ascribed to the insect central complex or mushroom body.
- The “ecological” theory posits direct bidirectional connections between sensing and action (sensing↔action), rejecting the need for internal representations.
- The theory of “embodied cognition” also posits direct bidirectional connections between sensing and action, but further specifies that that sensing↔action loops are organized into a modular, distributed control architecture that is tightly and strategically connected to the body. Unlike “ecological” theory, the

idea of embodied cognition does not reject internal representations, but it argues their role is limited — e.g., local efference copy signals might be used to inform locomotor control, or memorized snapshots might be used to guide navigation. The theory of embodied cognition took inspiration from subsumption architecture in robotic design, and the two schools of thought are closely linked.

For more details on these theories please see Hurley (2001) and Pfeifer, R. & Bongard (2006), as cited in our Reference list. The latter book has a foreword by Rodney Brooks linking embodied cognition with subsumption architecture.

Then, in the revised Discussion, we outline the key results that lead us to favor the notion of embodied cognition (and subsumption architecture, which is a specific implementation of embodied cognition). Namely,

- We show that **the core elements of behavioral control are a set of local feedback modules**, where effector neurons are strongly and specifically influenced by the sensors positioned to monitor the relevant effectors (**Fig. 2e**). We have now added a statistical test to the **Fig. 2e** legend to show that the pattern of sensor→effector influence is significantly different for each body part. This argues that sensory signals are unlikely to be routed through a centralized world representation. This result is compatible with both ecological theory and embodied cognition, but not with the classical theory.
- In a new analysis, we show that **there are strong local connections between effector pathways** (**Fig. 2g**). Note that the pattern of recurrent effector pathway influence is significantly different for each body part (**Fig. 2g** legend). The prominence of local effector connections further supports the embodied cognition model of neural function.
- We link many AN/DN superclusters with putative behavioral functions (**Fig. 3**), and **we highlight significant hierarchical connections among AN/DN superclusters** (**Fig. 5**, **Extended Data Fig. 6**). The division of control circuits into behavior-specific modules, with hierarchical connections among modules, is the most characteristic feature of subsumption architecture.
- Considering the network structure of the entire CNS, we found that **many CNS networks have a high influence on effector neurons** (**Fig. 6b**), further supporting the idea that behavioral control is distributed, rather than centralized.
- We found that **the central complex and mushroom body have low effector influence overall (Fig. 6b), and high influence on only a small subset of DNs and ANs (Fig. 6f,g)**. Together, these findings imply that the central complex and mushroom body have a supervisory role, and they supervise a subset of behaviors, not all behaviors. This conclusion is compatible with the idea of embodied cognition (which posits a limited role for memories and other internal representations); it is also compatible with subsumption architecture, where high-level modules supervise low-level modules, but high-level modules are inessential for many behaviors.

In short, our study provides many lines of evidence to support the idea of embodied cognition and specifically to support an implementation resembling subsumption architecture. Of course, it is possible that there is another theoretical framework that better explains the organization of the *Drosophila* CNS. If so, however, we think that theoretical framework does not yet exist. If the referee would like us to discuss another specific alternative, we would be sincerely interested in hearing this suggestion.

Fig. 6F [now 6E]: the membership of AN/DN clusters in CNS clusters does not demonstrate that ANs/DNs are bridging CNS networks. A more direct way of testing this would be to excise ANs/DNs from the network and see if influence scores are reduced between CNS networks. This would also help strengthen the argument presented on lines 589-590.

Response: We did not intend to argue that the bridges between CNS networks are solely composed of ANs/DNs, and we apologize if our original text gave this impression. We have revised the relevant sections of the main text to clarify our findings. Specifically, in the revised manuscript we state that:

- *"many CNS networks … contain ANs and DNs (**Extended Data Fig. 10b-c**)"*
- *"these links [between CNS networks] are disproportionately composed of DNs: when we counted each neuron's synaptic partners outside its assigned network, we found DNs had a relatively high proportion of outside partners (**Fig. 6e**)"*
- *"Most AN/DN superclusters are divided between two or three CNS networks (**Extended Data Fig. 10c**), consistent with the notion that DNs often form bridges between networks."*

We think the cited analyses provide clear support for our conclusions as stated here.

We did consider excising ANs/DNs to see how this changes influence scores. The main result is that the VNC-dominated CNS networks lose much of their influence from brain-dominated CNS networks, and vice versa. However, this result is somewhat expected, in our view, and we are hesitant to make much of it in the manuscript.

It would be useful to show data to support statements made in lines 865-868 regarding the error rate of cell type label assignment.

Response: We have have updated the Methods to provide more information on this issue:

> *"Our approach leverages morphology and connectivity matching to cell type the ~160,000 neurons in the BANC dataset by associating them with published reconstructions, namely FlyWire-FAFB v783[15] and MANC v1.2.1[11]. We assigned cell type labels by automatically identifying potential matches between BANC neurons and earlier datasets, based on neuron morphology and position (using NBLAST, **Fig. 1c-e**, **Extended Data Fig. 1b**) as well as connectivity (using NTAC in the optic lobe and network alignment followed by expert review in the VNC). Human annotators then manually reviewed cell type matches. NBLAST alone is competent but not sufficient to make matches; our careful manual review of all neurons in the VNC (human assessment of 3D morphology matches and some integration of known connection information) revealed that 55% of the cell types differ from the type of the max-NBLAST match in MANC. Specifically in the VNC, cell type assignments were more challenging because the reference dataset is of a male fly, and we had to account for sexual dimorphisms, which will be described in A.M, C.K.S et al., in prep. Nevertheless, we have assigned cell type labels to 63% of BANC neurons (98182 neurons, 90% excluding the optic lobes), with an estimated error rate of ~7% based on sampling 1,000 matched neurons for more careful review, incorporating cosine similarity to compare neurons between datasets. To do so, connectivity vectors were collapsed by cross-matched cell types up/downstream of each query neuron, and then their cosine similarity was calculated between all query neurons with a mid-to-high NBLAST score between BANC and FAFB/MANC."*

Stürner et al 2024 (doi: 10.1101/2024.06.04.596633) and Winding et al 2023 (doi: 10.1126/science.add9330) both report connectivity between ANs and DNs and suggest this connectivity may be important for behavioural control. How does this compare or relate to the findings in this manuscript?

Response: The connectivity between ANs and DNs is an interesting topic that has been discussed in Stürner et al., 2025 and Winding et al., 2023. These studies had access to limited data on AN/DN connectivity, as ANs and DNs were only partially reconstructed in those datasets. However, we agree that some of the observations in these studies provide an important foundation for our work.

In particular, we cite Stürner et al., 2025 as a precedent for the important observation that DNs often make synapses onto other DNs. In the revised manuscript, we have reviewed and added citations for Stürner et al., 2025 as appropriate. Stürner et al., 2025 compiled data on the sensory inputs to many DNs, and we have now added a citation to this work in connection with our analyses of sensory influence onto AN/DN superclusters. We also drew on Stürner et al., 2025 as a source of information about the likely function of specific DNs, and we cite this work appropriately. A large section of the Stürner et al., 2025 study is devoted to the topic of sexual dimorphism, which is outside the scope of our study.

An important contribution of Winding et al., 2023 was to develop and apply a metric of network distance. We cite this paper as an important precedent, and we describe the similarities and differences in our metric (see Methods: Alternative metrics of polysynaptic connectivity). Importantly, we show that our adjusted influence metric linearly correlates with the cascade distance metric of Winding et al., 2023. A key difference is that our metric is computationally efficient, which allowed us to compute it for the billions of pairwise interactions in the full adult connectome.

To facilitate comparisons between our data set and the data analyzed by Stürner et al., 2025 and Winding et al., 2023, we have added a new analysis of direct connectivity between AN/DN superclusters (**Extended Data Fig. 9**). Specifically, **Extended Data Fig. 9a** summarizes direct connections from ANs/DNs onto other

ANs/DNs. **Extended Data Fig. 9b** is an analysis of the subnetwork composed of every AN and DN, and the connections in this subnetwork. It focuses on short paths that transit between 3 or more superclusters (akin to 'zig-zag' motifs in Winding et al., 2023). This analysis shows that there are many two-hop chains passing between three different superclusters. The vignette in **Figure 5c** is one such example. We have now added another citation to Winding et al., 2023 in the legend to this figure.

References to unpublished work:

Lines 873-876: Claims about the significance/importance of a different manuscript in preparation should be removed, as they cannot be supported by the data presented here.

Response: Thank you. We expect some of our authors to release a comparison of the MANC and BANC VNC connectomes in the near future, based partly on the results of a challenge for the community that FlyWire issued earlier this year: https://codex.flywire.ai/app/vnc_matching_challenge. We have updated the text here to read:

> *"Notably, we estimate that as many as ~892 neurons of the VNC may be sexually dimorphic and/or sex-specific (A.M., C.K.S. et al., in prep. drawing on the 2025 FlyWire VNC matching challenge: https://codex.flywire.ai/app/vnc_matching_challenge) and cannot be matched well to MANC (which is a VNC sample from a male fly). Our annotation work enables direct comparisons with cell types identified in other datasets."*

In our original submission, we also cited J. Jones *in prep* for our information on nociceptive neurons. This work has since been preprinted, and we have updated this citation to Jones et al., 2025.

Line 129: What does this in prep manuscript contribute specifically? This should be clearly described.

Response: We had previously planned a different division of information between this manuscript, and the comparative one in preparation, which will focus on sexual dimorphisms in the VNC and will be led by different lead authors. We have removed the reference to this in prep manuscript from the Results section and instead describe it in the Methods section:

> *"Specifically in the VNC cell type assignments were more challenging because the reference dataset is of a male fly, and we had to account for sexual dimorphisms, and will be accounted for in A.M., C.K.S. et al., in prep."*

The GitHub repo was well structured and easy to navigate. We installed and ran the influence metric code without issues.

Response: We are glad that the referee found our influence algorithm runnable. For the referee's interest, we also note that we have made an R implementation in a new github repository, to hopefully expand its use, which can be found here: https://github.com/natverse/influencer/tree/main. We will also archive this repository before publication. Also please note that a static version of the code is available on the Harvard Dataverse, meaning the exact code used for the paper is snapshotted, while the github repository can continue to receive updates and improvements.

Referee #2:

I co-reviewed this manuscript with one of the reviewers who provided the listed reports.

Repository on Github is well organised and easy to navigate. I only tried to run the adjusted information metric analysis but this worked well.

Response: We are glad that the referee found our influence algorithm runnable. See our final response to Referee 1.

Referee #3:

[We have added numbers to this Referee's specific and substantive points, to allow cross-referencing. These numbers are shown in blue.]

A. Summary of the key results

This study provides the first full EM connectomics dataset that combines the brain and the ventral nerve cord (VNC) and thus entails the whole CNS. Moreover, the CNS is not reconstructed in isolation, but it is embodied by having identified numerous sensory cells in the periphery with their projections and connections in the CNS, and by assigning the motor neurons to their target muscles as well as by identifying and annotating numerous endocrine cells in the CNS with their specific visceral targets and neurohemal release sites. Therefore, this new connectome provides a yet unprecedented resource of a full CNS connectome that is highly embodied. It is a prime example of open science and excellent user accessibility, and it is well integrated with the already existing connectomics datasets.

In a next step, a heroic effort is taken to create a matrix that estimates the network influence of any one cell to any other cell, for all cells in the connectome. It is a highly impressive accomplishment to provide a network distance metric (named adjusted influence) for all possible neuron pairs (which yields 24 billion scores freely available at codex.flywire.ai/banc), and the adjusted influence scores presented here match other measures of network distances, but it does not consider synapse type and other highly important network features (see below).

This adjusted influence score is then used to make predictions on principles of the neural control of behavior. Here the key results are that sensory cells from one body part feedback to motor control for the same body part (essentially meaning that wing sensors are used for wing control), that local feedback circuits are linked by long range circuits into behavior centric modules (long range flight control circuits are closer linked to short range flight than to walking circuits), that descending and ascending neurons often influence voluntary movements of multiple body parts, and that brain regions involved in cognition have less direct influence on motor control, and are thus named supervisory. In sum, these data confirm previous work showing a distributed and parallelized control architecture.

Response: We thank the referee for highlighting the heroic scale of our study, its open science commitment, and integration with existing connectomes.

B. Originality and significance: if not novel, please include reference

This embodied full CNS connectome will be extremely useful for the scientific community. First, it really is the first connectome that connects the brain and the VNC networks and thus provides high quality novel tools for studies on information transfer between the brain and the VNC. Second, its embodiment provides novel approaches toward addressing the principles of the neural control of behavior. Third, given the efforts that have been done to include modifications of the typology annotations for the whole-brain and the VNC connectomes, comparisons between the existing Drosophila connectomics datasets and the new BANC presented here are strongly facilitated. This is a strong step towards allowing inter-animal comparisons in connectomics datasets, which is further supported by the superb availability of all Drosophila connectomes and the publicly available tools to study them.

Response: We sincerely appreciate this feedback.

1. Providing a matrix with 24 billion adjusted influence scores for all combinations of neuronal connections throughout CNS networks is indeed a novel scale and might be highly useful for many aspects of network analyses, but it has significant limitations. Accordingly, the interpretations that are drawn from employing this tool to the BANC connectome seem not all that novel. For example, the conclusion of a distributed network architecture for insect locomotion control has been reviewed already nearly 20 years ago (Cruse et al., 2007,

Insect walking is based on a decentralized architecture revealing a simple and robust controller, doi: 10.1098/rsta.2006.1913; Bueschges, 2005, Sensory control and organization of neural networks mediating coordination of multisegmental organs for locomotion doi: 10.1152/jn.00615.2004) as well as recently (Bueschges, Ache, 2025, Motor control on the move: from insights in insects to general mechanisms. Physiol Rev doi: 10.1152/physrev.00009.2024). Moreover, the manuscript repeatedly claims to have resolved an old debate as to whether behavioral control works in a top-down manner, where actions are selected centrally and then relayed to lower regions for implementation, or whether behavioral control is distributed. However, it appears clear that distributed control is the case across phyla, with local sensory feedback and reflexes as well as local motor control circuits interacting with higher centers. In fact, the only paper that is cited for a sole top-down model with centrally selected actions is Fiore et al., (2015), but this paper is rather an evolutionary comparison of the basal ganglia and the central complex, but not a pledge for centralized behavioral control. In fact, citing from this paper speaks for distributed control: 'In a cockroach, for example, forward walking can be triggered by touching its abdomen, even after its brain has been disconnected from its ventral nerve cord. But the animal is unable to respond to novel external cues and change its direction'. Similarly, the notion that motor neurons are influenced by local sensory cells in the same body part (local feedback loops) is not novel (see Mantziaris et al., 2017, doi: 10.1152/jn.00321.2017 and citations above) but rather somewhat trivial, isn't it clear that leg motor neurons receive direct or indirect feedback from the leg and wing motor neurons from the wings? In sum, it is recommended to down-tone the novelty of the findings on network architecture but to provide a more critical evaluation what the connectome and the adjusted influence score can really answer.

Response: The referee raises several important issues:

i. Previous work on neural circuits for insect walking: We agree that we should have given more prominence to previous work on circuits in the insect VNC related to walking control. In the Discussion, we now prominently cite a larger body of previous work on this topic; this includes Büschges 2005, Cruse et al. 2007, Mantziaris et al., 2017; Cheong et al., 2024, Büschges & Ache 2025, Lesser et al., 2024. As we noted in the text, this body of literature provides many examples of local feedback loops where there is a tight coupling between sensation and action. In the revised Discussion, we also state more explicitly how our work builds upon this existing literature:

> *"Our study extends prior work to show that local loops are located <u>throughout</u> the CNS, and they are consistently the <u>strongest</u> influences on effector neurons"* [emphasis added].

ii. Top-down models of control: We agree that our original submission did not adequately describe the top-down model of control, and we did not provide enough supporting citations. We have now revised the Discussion to address this issue. Specifically, we cite an authoritative critical review (Hurley, 2001) that describes the "classical" theory of behavioral control; in this classical theory, actions are selected by a centralized executive brain function (sensing→cognition→action, which Hurley calls the "classical sandwich". Under the classical theory, low-level implementation of motor patterns could be delegated to circuits in the spinal cord (or VNC), but in the classical view, these motor patterns are still selected by descending commands from a centralized executive controller in the brain. Also, in the classical view, cognition is still in some sense "distributed", insofar as cognition could never reside in a single neuron; it would necessarily involve neural populations. The key elements of the classical view are that cognition is a necessary step between sensing and acting, cognition resides in the brain (not the body), and cognition is not distributed over most (or all) of the CNS. We certainly appreciate the referee's enthusiasm for non-classical models, where cognition is fully distributed and embodied ("embodied cognition"), but with all due respect, we would argue that the referee's position (which we share!) is probably not the majority position in the fields of neuroscience or cognitive science. The assumptions of the classical view "are so ingrained in our computational metaphors that they usually go unquestioned" (Brooks, 2006, in the forward to Pfeifer & Bongard, 2006).

iii. Top-models of insect behavioral control: The referee points out that we did not do an adequate job in documenting how top-down control (the "classical" theory) has been applied in insects. We now cite a much larger set of papers to support our contention that centralized executive function is a mainstream opinion among insect neurobiologists. (Whether it is the dominant opinion or a minority opinion in insect neurobiology is difficult to say, but it certainly seems to be a mainstream opinion.) In our revised Discussion, we now cite a

number of prior publications to support this claim. Here we summarize the key passages for the referee, in chronological order:

- **Heisenberg, 2003 proposes that the mushroom body (MB) implements decision-making, or else it forms a key input to a downstream decision-making brain region**: "Mushroom bodies… seem to be involved in 'decision making. [Mushroom body output neurons] report to the decision stage of the brain…. what is [the] common denominator [linking four various functions of the mushroom body]? It seems that decision-like processes are involved in all four situations…. If, indeed, ancient mushroom bodies had no calyces and no prominent olfactory input, one would like to assume that their involvement in decision making is their original function and that they acquired their contribution to olfactory processing as a secondary trait."
- **Matheson, 2008 contrasts two models of motor planning in insects, one model where the central complex (CX) is a centralized motor planning region, and the other model where motor planning is centralized and largely residing in the VNC**: "where in the central nervous system might [motor] plans be computed? In primates like ourselves, motor planning involves activity spanning several regions of the brain, including the prefrontal cortex, premotor cortex and the cerebellum. The brains of insects contain many fewer neurons than those of vertebrates but are nevertheless highly complex, and are similarly organised into specialised regions. One of these in particular, the central complex, has been implicated in aspects of motor control including limb coordination. In *Drosophila,* mutations that disrupt central body function lead to locomotor deficits such as an inability to properly regulate step length or to orient towards attractive landmarks…. On the other hand, a large body of work tells us that many aspects of insect limb motor control are devolved to the chain of thoracic ganglia that form part of the ventral nerve cord. For example, just one of these ganglia is sufficient to generate aimed scratching movements in a locust, and a headless fruit fly can walk and groom. A challenge for the future is to see if the genetically tractable *Drosophila* continues to give us new ways of identifying and dissecting apart the neuronal structures responsible for motor planning."
- **Fiore et al., 2015 proposes that the CX is a central locus of action selection and deliberation, or else this executive function is embodied by the targets of the CX in the lateral accessory lobe of the brain**: "The multiplicity of similarities between central complex and basal ganglia suggests evolutionarily conserved computational mechanisms for action selection…. At least three sensory modalities (visual, mechanosensory, temperature) are relayed to the PB [protocerebral bridge, part of the CX] and FB [fan-shaped body, part of the CX] in the form of highly structured codes. Each represents one of many presumably simultaneous sensory events as well as recollections that must be assessed for their most probable adaptive value. The computational role of the EB [ellipsoid body, part of the CX] is to determine what within this incoming stream of data is best translated as possible motor actions. It is proposed that outputs from the EB serve to gate those parts of the LAL [lateral accessory lobe] that have executive control of the activity of descending channels…."
- **de Bivort & van Swinderen 2016 proposes that the CX is the central locus of attention**, which they define as the ability to "focus on only essential information from the environment at any given time". Specifically, they suggest that the FB "provides a potential substrate for stimulus selection" and "a general arousal-setting center" with "the capacity to suppress information flow in the insect brain", while the EB presents "the most convincing evidence so far for a neural correlate of visual attention in the insect brain", in part because it is "'is required for maintaining fixation on temporarily invisible objects", suggesting it is locus for "selecting (and ignoring) competing objects". Meanwhile, the "MB appears to mediate both learning and attentional processes". These authors also discuss alternative interpretations, e.g., that perhaps "neural selection and suppression mechanisms characteristic of selective attention are not necessarily confined to central brain structures such as the EB, FB, or MB. They might already operate in the periphery, consistent with experiments showing that behavioral states modulate neural activity in the optic lobes". Nonetheless, the lean away from this view: "If selective attention involves the recruitment of disparate neurons into transient ensembles… then [to] focus on any one structure might not be really informative. Nevertheless, the most convincing evidence to date has been found in the central complex, and specifically the EB".
- **Baran et al. 2025 propose that insects are endowed with executive functions**, and they highlight the MB and CX as key loci for executive functions. These authors define executive functions as "a set of domain-general cognitive processes that enable flexible, goal-directed behavior and regulate internal states in response to environmental demands". They state that "MBs are indispensable for tasks

requiring the formation of stimulus–rewards contingencies and behavioral flexibility," likening it explicitly to the prefrontal cortex in mammals. Meanwhile, they state that the CX is "particularly important for action selection and goal-directed navigation, functioning through the integration of internal states and external cues to orchestrate context-appropriate responses. These authors feel that available data "suggest that the MBs and CX form part of a distributed control architecture in which executive-like functions may emerge".

Thus, we think it is reasonable to treat the classical theory as a mainstream view of insect neurobiologists. Many of the previous citations noted by the referee pertain to "low-level" motor control problems, like limb control during locomotion. The idea that locomotion patterns arise in the spinal cord (or VNC) is pretty much universal. What is more controversial is the idea that action selection is distributed *throughout* the CNS. "Such an inverted viewpoint is perhaps not so radically controversial when applied to "low-level details" like how an animal or robot might locomote. But it is truly an affront to the modern viewpoint when it is applied to perception and even more so to thought itself" (Brooks 2006, in his introduction to Pfeifer & Bongard 2006). Note also that Referee 1 was somewhat skeptical of this viewpoint.

iv. How our results extend previous work in favor of embodied cognition: In the revised Discussion, we outline all the key results that lead us to favor the notion of embodied cognition (and subsumption architecture, which is a specific implementation of embodied cognition). Namely,

- We show that **the core elements of behavioral control are a set of local feedback modules**, where effector neurons are strongly and specifically influenced by the sensors positioned to monitor the relevant effectors (**Fig. 2e**). We have now added a statistical test to the **Fig. 2e** legend to show that the pattern of sensory→effector influence is significantly different for each body part. This argues that sensory signals are unlikely to be routed through a centralized world representation. This result is compatible with both ecological theory and embodied cognition, but not with classical theory.
- In a new analysis, we show that **there are strong local connections between effector pathways** (**Fig. 2g**). Note that the pattern of recurrent effector pathway influence is significantly different for each body part (**Fig. 2g** legend). The prominence of local effector connections further supports the embodied cognition model of neural function.
- We link many AN/DN superclusters with putative behavioral functions (**Fig. 3**), and **we highlight the strongest connections among AN/DN superclusters** (**Fig. 5**). The division of control circuits into behavior-specific modules, with evident hierarchical connections among modules, is the most characteristic feature of subsumption architecture.
- Considering the network structure of the entire CNS, we found that **many CNS networks have a high influence on effector neurons** (**Fig. 6b**), further supporting the idea that behavioral control is distributed, rather than centralized.
- We found that **the central complex and mushroom body have low effector influence overall (Fig. 6b), and high influence on only a small subset of DNs and ANs (Fig. 6f,g)**. Together, these findings imply that the central complex and mushroom body have a supervisory role, and they supervise a subset of behaviors, not all behaviors. This conclusion is compatible with the idea of embodied cognition (which posits a limited role for memories and other internal representations); it is also compatible with subsumption architecture, where high-level modules supervise low-level modules, but high-level modules are inessential for many behaviors.

C. Data & methodology: validity of approach, quality of data, quality of presentation

2. Although the adjusted influence score matches the results of other network distance measures that have previously been used, it has significant limitations that need to be considered and explained in more detail. First, it does not account for synapse type, not even for inhibitory synapses along the way. Second, it does not consider time, which would be important for differently long network distances along multiple paths between two neurons. This is circumvented by looking only at steady state, but in reality, the timing of synaptic inputs is critical for their summation. Third, it does neither consider synaptic diversity or plasticity. Some synapses have high release probabilities and depress whereas others have low release probabilities and facilitate. Many synapse activations along a path may be sub-threshold, which would cancel out any influence along that network path. Fifth, there are numerous postsynaptic mechanisms how to compute and modify synaptic input, including amplification, multiplication, and summation, which again depend on timing. It must be clear to the reader of a broad audience that adjusted influence does not account for any of this and is thus a vast

simplification that ignores numerous critical aspects of information transfer in neuronal networks. This does not mean that it cannot be used, but these limits must be pointed out in the text. It is stated in line 218 that the influences are merely predictions to generate testable hypotheses, but it should be clear that these predictions are based on very limited information and can also be false and misguiding. This does not mean that adjusted influence may not be of high value for future research, but it should be presented more carefully. The credibility of the adjusted influence score could be increased by experiments (see below).

Response: We apologise for the confusion which may stem from our use of the term 'activity' (see below). The influence score is a coarse grain approximation of neurons' impact on one another, not a biophysical model for the nervous system. Its strength lies in it being a simple, fast to calculate, linear metric that allows exploration of all possible pathways between seed and target neurons. It is roughly inversely proportional to network distance metrics, and so, by definition, a positive quantity. Our goal was to build a framework that allows us to think about large numbers of uncharacterized neurons that can provide intuition for future experimental work. While more complicated models, such as that from Shiu et al. 2024, have incorporated both spiking and inhibition, we do not think they produce more interpretable results for this purpose.

To be clearer about our choices, we have updated this section of the text:

> *"To tackle this problem, we developed an approach based on a linear dynamical systems description of signal propagation[86–89] across the brain and ventral nerve cord. Specifically, to compute the influence of one or more source neurons on any target neuron(s), we determine the effect of injecting a sustained signal into the source neurons, taking every downstream neuron's 'activation' as the weighted sum of its inputs. The weight is the number of synapses in that input connection[90], as a fraction of the postsynaptic cell's total synaptic input. For a target cell of interest, we take its steady-state response (**Fig. 2a**), log-transform it, and add a constant to ensure that the result is nonnegative. The metric (called 'adjusted influence') is inversely proportional to network distance from source to target (**Fig. 2b**, **Extended Data Fig. 4a**). Indeed, adjusted influence is in close agreement with previous network distance metrics[15,22,45], and like previous distance metrics[15,22,45], adjusted influence is an unsigned quantity. However, unlike those metrics, our metric is deterministic, linear and computationally efficient. For example, our metric does not involve manually assigning 'layers' of neurons for expensive connectivity matrix multiplications (unlike the 'effective connectivity' metric)[6]. In essence, the adjusted influence metric is simply an efficient way to estimate the effective distance between any pair of cells, and because it is efficient, it is straightforward to scale it up to quantify all pairwise distances in the connectome. This allowed us to precompute the pairwise adjusted influence of all individual neurons in the entire CNS onto all other individual neurons, yielding ~20 billion scores in total. Across the CNS, the modal pairwise adjusted influence score is 14 for direct connections and 8 for indirect connections (**Fig. 2c**). All scores are available to users via codex.flywire.ai/banc; we also provide code that allows users to compute these scores on demand[91]."*

And we have added this note to the Methods section on influence:

> *"Thus, adjusted influence is essentially a computationally efficient and deterministic method of estimating a quantity that is proportional to the effective number of hops separating the seed and the target. Because the number of hops is an unsigned quantity[17,22], it is reasonable that adjusted influence is also unsigned. Moreover, we do not actually know the signs of all connections in the connectome, particularly concerning glutamatergic, dopaminergic, serotonerergic, octopaminergic, and tyraminergic synapses. Notably, even inhibitory cells can drive behavior, e.g. via disinhibition; the only output of the vertebrate cerebellar cortex consists of GABAergic Purkinje cells, which trigger motor output when they pause firing, thereby disinhibiting their downstream targets[291]."*

3. The voxel size of the connectome is 4x4x45 nm, meaning that the x and y resolution is great (twice as large as compared to the MANC), but the z resolution is 5-6 times lower than that of the MANC dataset, and thus exceeding the width of the synaptic cleft by far. How did this affect synapse detection and transmitter class prediction? The scheme used to train and use a 3D convoluted neural network is well described on page 26, and the authors have to be commended for making a fully cited compilation of ground truth labels for each cell

type freely available (line 823), but it is important to have an estimate of the consequences of the relatively low z-resolution.

Response: Despite the HemiBrain image data being 8 x 8 x 8 nm^3/voxel and the FAFB-FlyWire data being 4 x 4 x 40 nm^3/voxel, neurotransmitter prediction performed worse on HemiBrain than FAFB-FlyWire data (per-synapse transmitter prediction accuracy was 87% on FAFB-FlyWire and 78% on HemiBrain and average accuracy of 94% for transmitter prediction per neuron on FAFB-FlyWire and 91% on HemiBrain) (Eckstein, Bates, et al. 2024). This is likely because the network learned higher x-y resolution features (Adjavon, et al. 2024) to differentiate synapses of different neurotransmitter types, though this will likely be overcome in the future with ever increasing amounts of ground truth data.

We found the synapse detection network (Yu, et al. 2025) performed well (F-score: 0.83, precision: 0.87, recall: 0.78) on the BANC's 4 x 4 x 45 nm^3/voxel data, though 8 x 8 x 8 nm^3/voxel can sometimes detect more synapses depending on signal-to-noise ratio of the image data. This is likely because the network also learns features beyond the synaptic cleft, including vesicles, and vesicle clouds, postsynaptic densities, presynaptic active zones, and other ultrastructural features correlated with synaptic contacts. See **Extended Data Fig. 1b**.

A z-resolution of 40-50 nm has been a gold standard for synapse-resolution, serial-section transmission electron microscopy (TEM) for a half century. 4 x 4 x 45 nm^3/voxel provides sufficient detail to reliably visualize chemical synapses and their associated ultrastructure. Because a typical synaptic cleft is ~20 nm in width, this means that ~2.5 synaptic clefts could, in principle, be stacked atop one another in z and be indistinguishable. However, given the non-crystalline organization of biological neuropil, this is rare and individual synapses are further disambiguated by additional ultrastructural features (see above) provided by high x-y resolution. The net result is that in some cases, synapse counts can be higher in data with 8-10 nm/pixel, z-resolution; however, the identification of synapses, and moreover, unitary connections comprising on average >10 synapses per connected pair of neurons is readily achieved in 4 x 4 x 45 nm^3/voxel EM data (**Extended Data Fig. 1g**).

4. Although the combination of brain and VNC with fully intact head connectives plus mostly intact nerve roots that allow for embodiment are major accomplishments, and the annotation of this dataset must have been an incredible endeavor, some parts are missing from the brain (such as the lamina and the retina are missing from the optic lobe). This is likely owed to the challenge of having entirely intact connectives to really map all DNs and ANs, and it is mentioned in the methods section, but it should also be made transparent in the main text (e.g. almost complete brain plus VNC connectome). Also, the preparation used looks bent and somewhat distorted (Fig. 1). This seems a pity considering that a total of roughly 30 working years have been invested by more than 150 people for proofreading. The reason for that choice should be explained in the text.

Response: Thank you. We detail in the Methods our losses more specifically in terms of the cell types and total number of neurons we think are affected. We have updated the first paragraph of the Results to be clear about these losses, with this sentence:

> *"Both the lamina and the ocellar ganglion are missing (see Methods), but these regions are present in the existing full adult fly brain dataset[16]."*

On the distortion of the whole dataset, this has only a small effect. We can 'bridge' neurons out of BANC into the symmetric standard spaces JRC2018F and JRCVNC2018F using a registration built with Elastix. This is available in our R package bancr, and we have now also made the Elastix files that describe the registration available on our Harvard Dataverse repository. We have made this clearer by adding the following line to the Methods:

> *"We generated a left-right registration for BANC based on a thinplate-spline warping registration built from matched points on identified pairs of ~30 DNs, available through the bancr R package (bancr::jrc2018f_to_banc_tpsreg, bancr::jrcvnc2018f_to_banc_tpsreg)."*

Additionally, users can co-visualize neurons from FAFB, HemiBrain, FANC and MANC in the BANC space at https://ng.banc.community/view.

5. Data presentation: Overall, the figures are well organized and clearly presented. Given the nature of the data, it is clear that the lettering becomes very small and hard to read. If possible at all, it is suggested to increase letter size wherever possible.

Response: Agreed. We have increased the size of the smallest fonts in the figures. If and when the manuscript is accepted, we will work with the production team to ensure that all fonts are legible. At the moment, it is difficult to optimize this, as different journals have different policies for determining the final size of each figure on the page, and the ultimate page-space budget is subject to editorial discretion.

Some specific suggestions for the figures are:

6. Figure 1 should also contain a 3-D reconstructed neuron from the BANC dataset with synapses to give the reader a clear visual impression of the resolution in all 3 spatial dimensions.

Response: Thank you for the suggestion. We have now made a new figure, **Extended Data Fig. 2**, to showcase our EM image data and neuronal morphologies. We have included a high-resolution image of a cross-section of the neck connective, 3D neuronal morphologies (note that the Z-resolution is high enough to produce visually cohesive meshes; i.e. 'steps' are not visible), and an image in the EM data of a synaptic bouton.

7. Figure 2: panel a on the right should not refer to source and target activity. If I understood correctly, neuronal activity was neither recorded nor simulated as membrane potential, but relative input synapse numbers were counted per layer along a network. The current representation gives the reader the impression that each network path was really subject to computational modeling of neuronal activity.

Response: Thank you. So as to avoid the misimpression that we are predicting neural 'activity', we have replaced this with 'activation' in **Fig. 2a**. The term 'activation' is commonly used in modeling artificial neural networks to denote the output of a network node (https://en.wikipedia.org/wiki/Activation_function).

As described in the Methods (under 'Influence'), the influence of a seed *s* on a target *t* is the steady-state activation of *t,* given a steady activation of *s*. However, because the network is linear, we can compute influence directly from the weight matrix W by calculating $-(W-I)^{-1}s$, where I is the identity matrix and s is the seed vector. The reason why 'influence' can be computed so efficiently is that W is a highly sparse matrix, and this enables us to leverage sparse matrix parallel computing libraries in our code. Simulating the network and computing influence directly (by computing $-(W-I)^{-1}s$) are mathematically identical solutions. Therefore, the two ways of describing influence are interchangable. The interchangability of these two concepts is an important part of the rationale for this metric, and therefore we think it is important to retain this point in the manuscript.

The referee is correct that the number of synapses per connection was incorporated into this metric. As noted in the Methods, the weight of each connection is taken as the number of synapses in that connection, normalized by the total count of input synapses onto the postsynaptic cell in question.

8. The legend to panel b must explain that the relation is linear because the y-axis shows log data to make it linear over layer number. It must also explain that the score starts at 24 in layer zero (at the source) because 24 was added to all values.

Response: We apologize for any confusion here. First, we should clarify our motivation for developing this metric. As noted in the Results (section titled "A metric of influence"),

> *"To interpret a whole-CNS connectome, we need a way to estimate the influence of neuron A on neuron B, for any pair of neurons. To date, there has been no computationally efficient method of estimating these influences."*

Previous studies have of course grappled with this same problem, and these studies proposed several methods for estimating something akin to influence, but these methods were not computationally efficient, and so they were not applied to the entire connectome. Our motivation in devising this metric was simply to have a computationally efficient method of estimating how much any neuron A might contribute to the activity of neuron B.

Next, to avoid confusion, we have revised the legend to **Fig. 2b** to clarify now adjusted influence is calculated. Now, the legend states,

> *"Adjusted influence is the log(influence), plus a constant (to ensure non-negativity)."*

We have also revised the relevant Methods section ('Influence') to clarify the rationale for our method of calculating adjusted influence. As noted in the Methods, the calculation of adjusted influence does involve taking the logarithm of influence, and there is a good rationale for this:

> *"In this type of simulated network, $\bar{r}$ will generally decay exponentially as the distance increases between the seed and the target (in network space). To correct for this, we take the logarithm of $\bar{r}$; note that $log(\bar{r})$ should be generally proportional to the number of hops separating the seed and the target. And because $log(\bar{r})$ is generally negative, we add a constant c that brings the values of $log(\bar{r})$ into the nonnegative range, for ease of display. The resulting value is called the "adjusted influence":*
>
> $$\text{adjusted influence} = log(\overline{r_{T,S}}) + c$$
>
> *… We confirmed that adjusted influence is approximately inversely proportional to the number of synaptic 'hops' separating the seed cells and target cells, as expected, and this was true for two different published metrics of hop length (**Fig. 2b**. and **Extended Data Fig. 4a**; see below for details of these previous metrics). Thus, adjusted influence is essentially a computationally efficient and deterministic method of estimating a quantity that is proportional to the effective number of hops separating the seed and the target. Because the number of hops is an unsigned quantity[17,22], it is reasonable that adjusted influence is also unsigned."*

Thus, we have not taken the log of our influence values in an ad hoc manner. We have taken the log in order to generate a metric that scales with the number of hops separating the seed and the target. And adding a constant c simply makes influence non-negative; we felt this was more intuitive (as readers would likely struggle to understand how 'network hops' could be negative), but this constant has no effect on any of our conclusions.

9. Panel e should not be on a normalized scale but show real adjusted influence score, so that e can be directly related to panels a to c.

Response: We use normalization here and in other figure panels to facilitate comparisons across groups. We have now updated the legend to always clearly state when we normalize these values and to provide our rationale for normalization. For this figure panel, we are asking which sensors most strongly influence each group of effector neurons, so we normalize the influence scores. We have now updated the legend to explain our rationale:

> *"Each row is normalized to the same range (0-1), to make clear which sensor groups are most influential for each effector group (14410 sensory neurons, 1031 effector neurons)."*

10. Also, panels d and e (belonging together) take a lot of figure space just to show that sensors from specific body parts influence mainly the effectors of these body parts, such that wing sensors influence wing effectors whereas leg sensors influence leg effectors.

Response: We think this panel is important, and although it did not surprise this referee, it may surprise some readers. In particular, some readers may have the (implicit) assumption that action selection, decision-making, and/or executive function is centralized in the central complex and/or mushroom body. For more detail please see our response to this referee's point #1 (above).

11. Figure 3 nicely explains the concept of DN and AN superclusters, though these results do not come as a surprise. The data presentation is difficult to follow due to the small size, but this again is likely owed to the type of analysis presented. Any way to increase font sizes would be nice but is not required.

Response: We have reorganized this figure in an effort to improve legibility and avoid tiny fonts.

12. A problem with the figure and accompanying text that must be addressed is that the reader may get the impression that the main input partners of motor neurons are DNs and ANs, but this is not the case. For example, wing power motor neurons as shown in panel h receive most of their direct input from local (VNC intrinsic) neurons (nearly 70%), but not from DNg27 (less than 10%) as might be suggested by the figure plus text. Moreover, in the BANC dataset only 5% of DNg27 output is to the depressor flight motor neurons 1 to 4, but 15% of the DNg27output is to only two VNC intrinsic interneurons. This is important, because VNC intrinsic interneurons seem to be the major premotor neurons in insects and are mostly neglected in the description of functional circuit architecture presented here. This should be clarified in the text.

Response: To avoid this misunderstanding, the proportion of inputs from ANs, DNs and intrinsic neurons (and other super classes) onto effector neurons is now shown in **Extended Data Fig. 5a**. We recognize though, that the referee is making the point that VNC intrinsic interneurons are not sufficiently discussed in our study. As noted above, we focused on ANs and DNs as they were a uniquely reconstructable aspect of the data. By the same token, we do not explicitly discuss the intrinsic neurons of the central brain, or the intrinsic neurons of the optic lobes, except to indicate the number of cells in each category. (These numbers are now tabulated in a more vivid and readable form in the revised version of **Fig. 1f**.). VNC intrinsic interneurons are represented schematically in some vignettes (e.g., **Fig. 4f**), just as intrinsic neurons of the central brain are represented schematically in some vignettes. **We made the decision to focus our circuit vignettes on ANs and DNs, as these are the CNS superclasses that have never before been described systematically in any previous study, with their full morphology and connectivity.** Readers who are mainly interested in VNC circuitry may be more interested in the existing VNC connectome papers, whereas readers who are mainly interested in central brain circuitry may be more interested in the existing central brain connectome papers.

Our approach in this study is to try to complement our large-scale analyses (which are necessarily abstract) with small-scale illustrative examples of cells and connections ("circuit vignettes"). These circuit vignettes have the virtue that the reader can directly verify them by browsing the connectome. However, a typical neuron in the connectome has tens or hundreds of synaptic partners, and it would be impossible to depict all the partners of all neurons in any vignette. To make this clear, when we show the first such circuit vignette (**Fig. 3g**), we now note explicitly in the legend,

> *"Here are elsewhere, where we show circuit vignettes, it should be noted that we are depicting only a small subset of the inputs and/or outputs to the neurons in question."*

13. Figure 4 exemplifies specializations and coordination within functional superclusters. It would be great if some experimental testing for the functional predictions shown in d and in e could be provided. Can specific predictable head positions not be taken when identified DNs or ANs that are specialized for these positions are acutely silenced? Can the animal not get into its normal standing position when silencing the negative feedback loop via AN06B002 after landing?

Response: We would love to test these predictions! However, we feel this is beyond the scope of our manuscript. Our primary mission in this study was to document the first fully embodied CNS connectome so that interested biologists and theorists could make use of the data, with the confidence that this dataset had been properly peer-reviewed. Our secondary mission was to generate tools for the community to analyze this

dataset. Finally, our third mission was to demonstrate how this dataset and associated tools can be used to generate testable new hypotheses. As previous connectomes have not been so fully “embodied”, we were particularly motivated to illustrate how this type of embodied connectome could be used to generate testable new hypotheses about the links between the CNS and the body. With regard to our biological analyses, we state frankly that,

> *“These inferences are merely predictions, and their value is to generate testable hypotheses… This open science effort should generate even more testable experimental hypotheses and, ultimately, new theories.”*

14. Figure 5 proposes interactions between behavior centric modules. Panel c exemplifies interactions between the threat response, the walking, and the proprioceptive superclusters to draw a picture that threat signals are integrated to stabilize defense posture while inhibiting walking drive. This seems intuitive and can be observed behaviorally (unless escape is evoked), but can these circuit motives really fully explain the behavior? What are additional features of the neurons and the circuits that should be considered to fully understand the behavior.

Response: We agree that this architecture is intuitive (to us, at least), and it can explain some features of animal behavior. To make this point clearer, in the revised Discussion, we now point out explicitly that,

> *“This type of architecture can also potentially account for some hierarchical relationships among animal behaviors[179,180].”*

However, we certainly do not claim that patterns of influence among ANs and DNs could fully explain any behavioral pattern. After all, ANs and DNs do not themselves produce behavior; they only produce behavior by acting (directly or indirectly) on effector neurons. As we emphasize in the text, we think behavioral patterns are an emergent property arising from the interactions of many modules, with ANs and DNs simply providing some key long-range connections, including hierarchical connections. We have revised the schematic in **Fig. 5d** to clarify this point.

15. However, it might be more informative to investigate supercluster interactions that must interact in highly defined ways to produce stereotypical sequences of complex motor behaviors. A great example would be courtship, where walking, singing, licking, and copulation must all be arranged in a well-coordinated sequential manner in the context of processing different streams of sensory signals.

Response: The referee raises an interesting issue here. A connectome is a static picture of connectivity, and as such, it does not incorporate time as an explicit variable. We can certainly use the connectome to place constraints on a model of neural dynamics or behavioral dynamics. But any dynamical model (even if it is connectome-constrained) requires us to make additional assumptions that are not based on connectome data. For example, even the simplest (linear) model of the full CNS network would need to make an assumption about the magnitude of neural time constants. Moreover, as the referee notes, real neural dynamics and behavioral dynamics will be continuously influenced by the interactions between the CNS, the body, and the world (including the behaviors of other individuals -- as in courtship). In effect, CNS/body/world are in a continuous dynamical loop. In the future, it will be interesting to apply the BANC data set as a constraint in these models. Indeed, large research groups are currently currently working toward this sort of goal, by extending existing biophysically-constrained models (e.g., Vaxenburg et al., 2025 https://doi.org/10.1038/s41586-025-09029-4) to include constraints from connectomes. That said, this is also a difficult undertaking, and we feel it is beyond the scope of our manuscript.

D. Appropriate use of statistics and treatment of uncertainties

16. Despite the uncertainties related to a broad validity and a broad applicability of the adjusted influence score and some uncertainties about the consequences of the relatively low z-resolution, the data are generated and presented on an exceptionally high level. There are no other concerns about statistics or uncertainties.

Response: Point taken. As the referee did not state a specific concern or suggested remedy, please see our response below to the referee's subsequent more specific point.

E. Conclusions: robustness, validity, reliability

17. The connectome is fantastic, well explained, superbly annotated, and a true prime example of open science that is expected to act as a cornerstone of many future research projects and novel insight into network architecture for behavioral control. The metric of influence provides an amazing look-up table for estimates of network distance that can be highly useful to form hypotheses and make predictions for future research directions, but its validity and applicability remains quite limited at this point. The conclusions drawn from this metric of influence on network architecture are not entirely novel and only partly convincing.

Response: See our response below to the referee's subsequent more specific point (#18).

F. Suggested improvements: experiments, data for possible revision

18. The adjusted influence score requires validation if employed that broadly.

Response: We certainly agree on the importance of validation. As noted above in our response to this referee's points #2, #7, and #8, previous studies have recognized the need for some way to estimate how much any neuron A might contribute to the activity of neuron B, and several previous studies proposed methods for estimating something akin to influence. However, these methods were not computationally efficient, and so they were not applied to the entire connectome. Our motivation in devising the influence metric was simply to have a computationally efficient method of estimating how much any neuron A might contribute to the activity of neuron B. Thus, the relevant validation of this effort is to ask whether 'adjusted influence' is well-correlated with these previous metrics. Indeed, we confirmed that adjusted influence is strongly related (inversely proportional) to the number of synaptic 'hops' separating the seed cells and target cells, as expected, and this was true for two different published metrics of hop length (**Fig. 2b**. and **Extended Data Fig. 4a**). The Methods section titled "Alternative metrics of polysynaptic connectivity" provides more details on these previous methods.

As noted above in our response to this referee's point #7, simulating the network and computing influence directly (by computing $-(W-I)^{-1}s$) are mathematically identical solutions. The concept of a simulation was important to our rationale in developing this metric, but in order to efficiently compute the billions of influence scores in the entire connectome, we used the direct method based on the weight matrix W, not a simulation. Our influence metric is certainly not intended as a substitute for neurophysiological modelling. In the Results section where we introduce this metric, we state this clearly:

> *"In the following sections, we say A "influences" B, as shorthand for a high adjusted influence score (A→B). These scores do not demonstrate functional connections, and they are no substitute for experiments. The value of these scores is that they allow us to make provisional inferences on a large scale… These inferences are merely predictions, and their value is to generate testable hypotheses. "*

G. References: appropriate credit to previous work?

19. The recent advances in connectomics analyses and associated tools and techniques as well as recent work on Drosophila functional circuit analyses are well credited. The text would greatly benefit from referring at least exemplarily to the large body of old work on network architecture for behavioral control that has described a decentralized and parallelized network structure, ANs with information transfer from the brain to the VNC and many other aspects shown here already decades ago. Similarly, a comparison to the network architecture and information flow between brain and spinal cord as it is known from the vertebrate CNS would be extremely helpful to integrate the wonderful advances deriving from Drosophila connectomics in a broader perspective.

Response: As noted above in our response to this referee's point #1, we should have given more prominence to previous work on circuits in the insect VNC related to walking control. In the Discussion of our revised submission, we now prominently cite a larger body of previous work on this topic; this includes Büschges 2005, Cruse et al. 2007, Mantziaris et al., 2017; Cheong et al., 2024, Büschges & Ache 2025, Lesser et al., 2024. As we noted in the text, this body of literature provides many examples of local feedback loops where there is a

tight coupling between sensation and action. In the revised Discussion, we also state more explicitly how our work builds upon this existing literature.

In principle, we would like to include new Discussion material comparing brain-VNC circuits in insects with brain-spinal cord circuits in vertebrates. We have not included a comparative section like this for two reasons. First, Nature limits the space available for Discussion material. Second, and more fundamentally, our focus in this manuscript is on the large-scale analysis of all ANs and DNs, based on connectivity data with single-synapse resolution, and there is currently no available comparable data set in vertebrates, so a comparable analysis is not yet possible. We hope interested readers will consult Büschges & Ache 2025 for a topical summary of common general principles.

H. Clarity and context: lucidity of abstract/summary, appropriateness of abstract, introduction and conclusions
20. Clarity and context of the abstract are appropriate and comprehensible. The same is true for the intro and conclusions. However, it is recommended to take older work into account and downtone occasionally (see above and below).

Response: Please see our response to this referee's point #1 and #19, above.

21. Line 255 states that it is sometimes suggested that DNs send motor commands to the VNC whereas ANs send sensory signals and predictive motor signals back to the brain. Please clarify what is meant by predictive motor signals. Also, again this seems like a somewhat constructed debate. It is clear since more than 30 years that ascending neurons can also transfer sensory signals from the insect brain to the VNC (e.g. the locust A4I1 neuron with its cell body in the fourth abdominal ganglion transfers mechanosensory information from the head to the thoracic flight motor circuits, Burrows and Pflüger, 1992, Output connections of a wind sensitive interneurone with motor neurones innervating flight steering muscles in the locust. doi: 10.1007/BF00194576, or even older Pflueger, 1984, The large fourth abdominal intersegmental interneuron: a new type of wind-sensitive ventral cord interneuron in locusts, doi: 10.1002/cne.902220303). This does not at all decrease the great value that this paper now adds all ANs and DNs with their putative input and output synapses to a publicly available database, which is really fantastic. But the reader should not get the impression that this resolved a debate of whether ascending neurons send information only from the VNC to the brain or also in the opposite direction.

Response: We thank the referee for highlighting previous studies in other insects showing that ANs can transfer information from the brain to the VNC. In the relevant passage in the Results, we have now added a statement summarizing this work, together with citations to Burrows and Pflüger,1992 and also Pflueger,1984.

Regarding predictive motor signals, the sentence in question references Cheong et al., 2024. This study noted that, "To distinguish reafference from exafference, the central nervous system implements a broad class of feedforward circuits, commonly referred to as corollary discharge circuits (CDCs), which provide predictive motor information to sensory and motor pathways…. The fundamental importance of these predictive motor signals is highlighted by their failure, which results in attributional errors associated with nearly every form of sensory hallucination whether fatigue or disease induced. Thus, predictive motor signals from CDCs are essential for the animal to effectively use sensory information to make adaptive choices that optimize behavioral performance."

Regarding prevailing views in the literature on the functions of ANs: the sentence in question refers to Simpson 2024 (cited in connection with this sentence, also cited in Büschges & Ache 2025). Simpson 2024 exemplifies the view of DNs as cells that simply send motor commands to the VNC: "DNs are responsible for transmitting sensory and contextual information from the brain… to enable detailed execution of motor programs in the VNC… Descending neurons connecting the brain to the ventral nervous system sit at the convergence point, responding to sensory cues and initiating motor programs." This review mentions ANs only as the source of tactile sensory feedback to the brain. Cheong et al., 2024 points out that ANs can carry not only sensory feedback but also corollary discharge. Together, we think studies are representative of the prevailing view in the field of *Drosophila* neurobiology, which is that DNs drive motor programs, while ANs send information from the VNC back to the brain. In the revised manuscript, we have placed this view in the broader context of

classic insect neurobiology, which actually presents a more complex picture (consistent with our results in this manuscript).

22. As already mentioned above, the reader may get the impression that DNs are a major direct input to motor neurons.

Response: Agreed. To address this concern, we have now added a new analysis (**Extended Data Fig. 5a)** to show that a sizable minority of the inputs to motor neurons and endocrine neurons come directly from ANs and DNs. That said, this analysis also makes it clear that most inputs to motor neurons and endocrine neurons arise from neurons intrinsic to the VNC or brain.

The referee may be concerned that our vignettes focus specifically on relatively short pathways from ANs/DNs to effector neurons. We decided to highlight these short pathways simply because there is not sufficient space in the figures to illustrate the longer pathways, and we wanted the vignettes to show “identifiable neurons” with single cell/synapse resolution. To clarify this point, we have now revised the section of the Results where we explain our choice of circuit vignettes:

> *“we will use influence scores to make inferences, and to bolster these inferences, we will show example circuit motifs showcasing instances of relatively direct connections”* [emphasis added].

Moreover, in the legend to Fig. 3g, we have now inserted language to clarify that:

> *“Here are elsewhere, where we show circuit vignettes, it should be noted that we are depicting only a small subset of the inputs and/or outputs to the neurons in question. Our vignettes focus mainly on direct connections, but high influence values often reflect indirect connections.”*

Discussion:
23. Third para: A major point in the discussion claims to have resolved an old debate as to whether behavioral control works in a top-down manner, where actions are selected centrally and then relayed to lower regions for implementation or whether behavioral control is distributed. This reads like an artificial debate because it appears clear that distributed control is the case across phyla, with local sensory feedback and reflexes as well as local motor control circuits interacting with higher centers. In fact, the only paper that is cited for a sole top-down model with centrally selected actions is Fiore et al., (2015), but this paper is rather an evolutionary comparison of the basal ganglia and the central complex, but not a pledge for centralized behavioral control. Citing from this paper speaks for distributed control: ‘In a cockroach, for example, forward walking can be triggered by touching its abdomen, even after its brain has been disconnected from its ventral nerve cord. But the animal is unable to respond to novel external cues and change its direction’. It is thus suggested to remove the claim that a decentralized architecture for behavioral control is a novel finding (mentioned this already above).

Response: As noted above in our response to this referee’s point #1, previous literature is mixed on this issue. We agree that we should have given more prominence to previous work on circuits in the insect VNC related to walking control, and in the Discussion we now prominently cite a larger body of previous work on this topic; this includes Büschges 2005, Cruse et al. 2007, Mantziaris et al., 2017; Cheong et al., 2024, Büschges & Ache 2025, Lesser et al., 2024. We also agree that our original submission did not adequately describe the top-down model of control, and we have now revised the Discussion to fix this problem. Briefly, according to the classical theory, actions are selected by a centralized executive brain function. Low-level implementation of motor patterns could be delegated to circuits in the spinal cord (or VNC), but in the classical view, these motor patterns are still selected by descending commands from a centralized executive controller in the brain. Fiore et al., 2015 advocates for this classical theory; this review article does point out that a headless cockroach can walk, but the point of this passage is to stress that a headless cockroach cannot engage in purposeful behavior, which (in the view of Fiore et al.) is evidence that action selection resides in the brain. In the revised Discussion, we now cite many more papers that consider the classical theory of behavioral control in insects. We do not argue that this is the dominant theory; we simply argue that it is a mainstream theory. In the revised Discussion, we also review alternative relevant theories of behavioral control in the literature, namely the

ecological theory and the theory of embodied cognition. We point out that our results are overall most supportive of the theory of embodied cognition. At the same time, we make it very clear that this idea is not novel to our study.

24. Similarly, the claim that effector cells, such as motor neurons are primarily controlled by local sensory feedback seems not quite correct and misleading. First, the direct synaptic input to motor neurons comes mostly from local interneurons (here named VNC intrinsic neurons, which are mostly dismissed throughout the manuscript) and not from sensory neurons. For example, for flight power motor neurons, 10 % of the synaptic input is from sensory neurons, but roughly 70% of the direct synaptic input is from VNC intrinsic neurons (according to the MANC EM dataset).

Response: We regret that the referee felt that we dismissed the contributions of VNC intrinsic neurons (or any other intrinsic neurons), as this was not our intention. To address this issue, we have made several revisions.

- In the legend to **Fig. 2b**, we have added the following sentence: "Note that strong influence can come from either direct or <u>indirect</u> connectivity." [emphasis added]. This is intended to draw the reader's attention to the fact that strong influence does not necessarily mean direct connectivity.
- In **Fig. 2f**, where we first schematize sensory-to-motor interactions, we have added this sentence to the legend: "Connections from sensory neurons to effector neurons are generally indirect, mediated by intrinsic cells of the brain or VNC."
- In **Fig. 2h**, we have added a new schematic that depicts the connections from sensory neurons onto effector neurons as *indirect* connections. The legend to this new figure panel emphasizes the roles of intrinsic neurons in behavioral control: "Sensory neurons and effector neurons are connected within functional modules, <u>largely via intrinsic neurons</u>. Effector neuron pathways also influence each other, particularly within a module. Modules are weakly connected via long-range connections. Within each controller, <u>the intrinsic circuitry of the CNS also shapes motor output (e.g., via central pattern generators)</u>" [emphasis added].
- In the passage of the Results that summarizes Fig. 2, we have added new text to draw attention to the roles of intrinsic neurons: "Within each module, sensory neurons influence effector neurons (<u>largely indirectly, via intrinsic neurons</u>); meanwhile, effector neurons drive changes in body state, which produces sensory feedback (**Fig. 2h**)" [emphasis added].
- We have added a new analysis (**Extended Data Fig. 5a**) showing that most of the synaptic links onto effector neurons do in fact come from intrinsic neurons, either VNC intrinsic neurons or brain intrinsic neurons.
- In the legend to **Fig. 4f**, which depicts a VNC intrinsic cell type (IN12B018) in providing sensory feedback, we have added the following sentence: "Note a key role of VNC interneurons in processing sensory feedback."
- In the legend to **Fig. 5c**, which depicts a VNC intrinsic cell type (IN18B011) in mediating a connection from DNs to leg motor neurons, we have added the following sentence: "Note a key role of VNC interneurons in recruiting leg motor neurons."
- In the legend to **Fig. 6h**, which depicts two VNC intrinsic cell types (IN06B032 and IN07B010) in mediating a connection from DNs to leg motor neurons (left panel), we have added the following sentence: "Note a key role of VNC interneurons in recruiting leg motor neurons." (Note also that in the middle panel and right panel of **Fig. 6h**, additional VNC intrinsic neurons are depicted as mediating indirect connections from DNs to wing motor neurons; these intrinsic neurons are not identified by cell type name for reasons of space, but they are represented accurately in the circuit diagram.)
- As noted above, we have now revised the section of the Results where we explain our choice of circuit vignettes: "we will use influence scores to make inferences, and to bolster these inferences, we will show example circuit motifs <u>showcasing instances of relatively direct connections</u>" [emphasis added]. Moreover, in the legend to **Fig. 3g**, we have now inserted language to clarify that "Here are elsewhere, where we show circuit vignettes, it should be noted that we are depicting only a small subset of the inputs and/or outputs to the neurons in question. Our vignettes focus mainly on direct connections, but high influence values often reflect indirect connections."
- We have removed the sentence in the Discussion that described DNs and ANs as "wires" for actuating effector cells in different combinations, as the referee pointed out that this could be misconstrued as minimizing the roles of VNC intrinsic neurons and other intrinsic neurons.

- In the revised Discussion, we now explicitly draw attention to the fact that DNs and ANs act indirectly: "Importantly, DNs and ANs typically influence effector neurons indirectly, via interneurons, and the circuitry of these interneurons often plays a key role in structuring behavioral dynamics", citing here several relevant research articles and reviews.

Our study uses sensory neurons and efferent neurons as points of reference because these neurons are connected to body parts, and the focus of our analysis has been to show how the CNS is indeed "embodied", i.e. connected to body parts in concrete ways. Naturally, most neurons in the CNS are connected to the body indirectly (via intrinsic neurons), rather than directly. We apologize for creating the misimpression that we are dismissing the roles of interneurons.

25. Second, patterned motor neuron activity can be generated by local central pattern generating networks without sensory feedback. Of course, in insect walking, to coordinate activity across joints and segments sensory feedback provides critical feedback about load, position, and movement, thus shaping the timing and strength of motor neuron bursts and ensuring adaptive coordination between joints (in pat also already mentioned above).

Response: We agree that central pattern generators (CPGs) are crucial elements of locomotion, as well as other cyclical behavioral patterns (pharynx movements during feeding, leg movements during grooming, etc.). We have not suggested that CPG rhythms require sensory input. Our analyses of sensory→effector neuron influences are simply intended to highlight those pathways for sensory input that do exist in the connectome. As noted above in our response to this referee's point 24, we have now made several revisions to the text to draw attention to the roles of VNC intrinsic neurons (and other intrinsic neurons) in shaping behavior. In the future, it will be interesting to incorporate BANC data into dynamical models of CPGs. However, we feel this is beyond the scope of our manuscript.

26. Fourth para: Suggests that combinatorial codes (across fiber patterns) fine-tune action patterns. Although the example that different threat DNs mediate different threat responses nicely reflects different actions being mediated by different descending neurons, there is no proof in the current data that combinatorial codes of DN activity fine-tune behavior. Again, this notion dismisses the importance of local (VNC intrinsic) interneurons that are the main input partners to motor neurons and themselves integrate input from sensory neurons and DNs. The picture drawn gives a somewhat misleading impression of motor control. It is even suggested on page 22, lines 579-581 'to think of DNs and ANs as "wires" for actuating effector cells in different combinations'. This would be in strict contradiction of local central pattern generating networks that cause coordinated activity motor neuron firing for each joint, and sensory feedback and intersegmental communication are needed for walking coordination. In stick insects, for example, coordinated motor neuron firing for leg movement is primarily generated by local CPGs for each leg joint (Bidaye et al., 2018, doi: 10.1152/jn.00658.2017; Strohmer et al., 2022, doi: 10.3389/finsc.2022.818449; Ruthe et al., 2024, doi: 10.1016/j.cub.2024.01.026), with joint- and segment-specific modulation. However, robust, adaptive coordination - especially across multiple legs - depends critically on sensory feedback and intersegmental communication (Mantziaris et al., 2017, doi: 10.1152/jn.00321.2017). This modular, feedback-driven system enables flexible and stable walking patterns.

Response: Above, in our response to this referee's point 24, we list all the changes we have made to the manuscript to highlight the roles of VNC intrinsic neurons (and other intrinsic neurons). One of these revisions was to remove the sentence in the Discussion that described DNs and ANs as "wires" for actuating effector cells in different combinations, as we agree that this could be misconstrued as minimizing the role of VNC intrinsic neurons. In the same passage of the Discussion, we have now inserted citations to all the papers suggested by the referee, in connection with the statement that ANs and DNs typically influence effector neurons indirectly, via interneurons, and the circuitry of these interneurons often plays a key role in structuring behavioral dynamics.

27. All code is very well documented and freely available, a wonderful example of open science

Response: Thank you. This was a major area of focus for us. We want to encourage the community to use and evolve the resource as much as possible, and as such, we need to provide good data access and

documentation. We are also very happy to answer the community by email or similar, for help navigating the large amount of data we have made available.

Referee #4:

This landmark paper by Bates and colleagues provide the most comprehensive volume electron microscope dataset of an adult female Drosophila melanogaster nervous system to date, unique including the brain and the ventral nerve cord in a single volume. The authors focus on connectome modularity and the connectivity of ascending and descending neurons, fully reconstructed for the first time. One of the many findings of the study include the prevalence of local feedback loops with higher brain centres having supervisory functions. The paper will be a central resource for fly neurobiology and is of general interest and importance for circuit neuroscience.

Strengths:

The dataset is unique, of high quality and extensively annotated and allows the study of the connectivity between the brain and the ventral nerve cord. The data are made accessible on an online platform and the detailed and well-organised code enables the exploration of the connectome. The circuit analyses and functional predictions are compelling and will stimulate further experimental work. Besides the mapping of the whole connectome annotation, the dataset is also annotated with neurotransmitters, allowing to assign signs to the connections. The data are put into the context of the extensive literature on fly connectomics and the function of individual neuron types, e.g. ascending and descending neurons.

Weaknesses:

Some additional steps will be essential to guarantee the long-term archiving of the code and analysis workflows.
Since the dataset comprises a dissected adult brain, the identity of some of the sensory nerves remains ambiguous. Some other parts of the NS (e.g. ocelli) are also missing.

Response: We have added additional information on how we archive the code and data (see responses below for details). In addition to the description in the Methods, we have now updated the first paragraph of the results section to explicitly note the tissue loss, adding:

> *"Both the lamina and the ocellar ganglion are missing (see Methods), but these regions are present in the existing full adult fly brain dataset[16]."*

As this referee correctly points out, we initially identified nerves based on them being distinct as they entered/exited the CNS, but co-bundled nerves are distinct in the periphery. However, as we continue to improve the metadata annotations, we have added more detailed nerve annotations for specific sensory neurons, which can be assigned based on their cell type identity. For example, we separate out the eye nerve and the occipital nerves. Since the last version of this manuscript, we have also demarcated neurons belonging to the stomodeal, pharyngeal, and accessory pharyngeal nerve instead of the combined pharyngeal or accessory pharyngeal nerve. The current list of nerve annotations can be found in Supplementary Data 1.

Essential revisions:

While the paper is exquisitely annotated and is made accessible to the community in an exemplary manner, the computational resources are only provided on GitHub. GitHub is not a permanent code archive. The authors should also link all GitHub repositories and permanently archive them and provide the DOIs in a long-term code/data archive, for example Zenodo. https://help.zenodo.org/docs/github/enable-repository/

Response: Thank you for reviewing our code. While GitHub is the best source for our latest code in the evolving project, we agree with the referee that a permanent code archive is necessary, and we did archive all the code on a permanent, DOI-mining repository, the Harvard Dataverse. We apologize that this was not made

clear in the earlier version of our manuscript. We use the Harvard Dataverse rather than Zenodo. We did this because they offer more storage than Zenodo (2.5 TB vs. 200 GB), and our data could not all fit on a single Zenodo archive. The Harvard Dataverse DOI is *10.7910/DVN/8TFGGB*. In addition, we have now archived the image data with an NIH-funded data archive BossDB.org, and include this line in our methods section:

> *"Aligned image data is available on BossDB (DOI: https://doi.org/10.60533/boss-2025-941r, or URL: https://bossdb.org/project/bates_phelps_kim_yang2025)"*

At publication, we will update from v626 (note, v731 is used for **Fig. 1**, to show the reviewers the betterment of the dataset over the review process) to a new publication-version of the dataset, and this will be made clear for each file and for the update as a whole, as the Harvard Dataverse provides versioning. We will include the Dataverse version information in the published text. Because readers may be unfamiliar with the Dataverse, we have now added the following to the first section of the Results:

> *"Neuron morphologies, synapse detections, annotations, indirect connectivity scores, analysis code and more are available as direct downloads from the Harvard Dataverse, a DOI-minting repository (https://doi.org/10.7910/DVN/8TFGGB) (**Extended Data Fig. 1h**)."*

We have also updated the relevant portion of the Methods section to read:

> *"Static data and code dumps are also available for download from a DOI-minting repository, the Harvard Dataverse (DOI: https://doi.org/10.7910/DVN/8TFGGB). Direct downloads include: the synaptic connectivity edgelist, NBLAST results of BANC neurons against Hemibrain, FAFB, FANC and MANC as well as BANC all-by-all; neuronal L2 skeletons (made using: https://github.com/CAVEconnectome/pcg_skel); neuronal colorMIPs; influence scores; our aligned BANC metadata; template registrations; behavioral data for the BANC fly; and snapshots of our code."*

The GitHub repositories are spaces we also guide the user to, because BANC is a live project, and these repositories will continue to receive helpful updates for a while. At the time of preprinting, each GitHub repository was snapshot and put on the Dataverse. However, in reviewing this issue, we also noticed some missed data and some tools that can be improved: we have now added the behavioral data for the BANC fly, template registrations, an R version of the influence score calculation, and a new R package for plotting publication-quality neuroanatomy to the Dataverse.

Minor comments:

- It is sometimes unclear which of the influences or actions of the discussed neurons have already been tested experimentally and which are predicted based on the new connectome resource (e.g., page 11, lines 392f: DNa06 also targets two ANs that are positioned to excite neck and/or eye motor neurons.)

Response: The newly predicted influences or actions we discuss in our biological vignettes are based on our new resource, but have not yet been experimentally tested, which we feel is beyond the scope of this manuscript. Our primary mission in this study was to document the first fully embodied brain-and-cord connectome so that interested biologists and theorists could make use of the data, with the confidence that this dataset had been properly peer-reviewed. Our secondary mission was to generate tools for the community to analyze this dataset. Finally, our third mission was to demonstrate how this dataset and associated tools can be used to generate testable new hypotheses. As previous connectomes have not been so fully "embodied", we were particularly motivated to illustrate how this type of embodied connectome could be used to generate testable new hypotheses about the links between the CNS and the body. With regard to our biological analyses, we state that:

> *"These inferences are merely predictions, and their value is to generate testable hypotheses… This open science effort should generate even more testable experimental hypotheses and, ultimately, new theories."*

- Abstract, lines 64-67: What is referred to by the authors as „worms“ are indeed two representatives from two completely different animal groups (Ecdysozoa and Lophotrochozoa) and may be better referred to as ‘an annelid and a nematode’, otherwise it is misleading.

Response: While we agree with the referee that these animals belong to different clades, we would like to keep our wording here. Annelids are segmented worms, and nematodes are round worms. One of the key benefits to the BANC we have sought to point out is that it is the first ~full CNS for a limbed animal. The terms ‘annelid and nematode’ will be opaque to some, perhaps most, general readers.

- The authors define effector cells as “(motor neurons, endocrine cells and efferent neurons targeting the viscera)"
In the zoological literature, the term ‘effector cell’ more commonly refers to muscles, gland cells, multiciliated cells (not in flies obviously), pigment cells, maybe endocrine cells. These are effectors because ultimately these cells 'effectuate' movements. These cells are obviously missing from the CNS connectome but many of them can be inferred (e.g. MN innervation). It would be better to stick to the more general terminology of motor neuron, endocrine cells and not lump these neurons together under effector cells.

Response: We agree that this is a tricky issue, and also agree with the referee that our usage of the original terms was unclear. However, as we perform many analyses that consider all of these cells together as outputs of the CNS, we believe we need a single term to refer to them. Additionally, there are some neurons for which it is unknown whether they are motor neurons or endocrine neurons, where a more general term would be more appropriate. We have now gone with “effector neuron” instead of “effector” or “effector cell” when referring to motor neurons, endocrine neurons, and efferent neurons targeting the viscera. We have changed the terms throughout the manuscript and have added the following definition to the Results:

> *“[...] an ’effector neuron’ is defined here as a presumptive motor neuron, endocrine cell, or efferent neuron targeting the viscera, as these are the ‘effectors’ of the nervous system (**Fig. 2d**)”*

We have also added a section to the Methods (under ‘Annotation taxonomy’) to explain our rationale:

> *“Elsewhere in the literature, ‘effector’ is often understood as denoting a muscle or gland. Here we use ‘effector neurons’ to describe motor neurons, endocrine cells, and efferent neurons targeting the viscera. All these cells effectuate change outside the CNS, and thus they are the relevant output units of the CNS.”*

- Page 3, lines 131: If inter-individual variability exceeding 25% it will be hard to generalize findings. It would be important to elaborate more on this and e.g. report whether these unmatched neurons are in a particular part of the nervous system (e.g. mushroom bodies), are due to technical or other differences. In this context, it would also be important to mention what is know about inter-sex variability.

Response: We apologise for the misunderstanding. We actually think the CNS is highly stereotyped between individuals (Schlegel et al., 2024). Here, we think the referee is referring to this sentence from the previous version:

> *“Some neurons are still not cross-matched (26% of BANC neurons excluding the optic lobes), and some of these neurons likely cannot be matched even with more effort, due to inter-individual variability in cell morphology.”*

Our wording may have misrepresented the case. The primary reason that neurons are not matched is that in-depth human review is ongoing, as this is a labour intensive process. Indeed, since the previous version, the percentage of neurons that are not cross-matched has been reduced from 26% to 10%. We have updated the sentence to reflect this new percentage. We have also changed “inter-individual variability” to “inter-dataset variability”:

“Some neurons are still not cross-matched (10% of BANC neurons excluding the optic lobes), and some of these neurons likely cannot be matched even with more effort, due to inter-dataset variability in cell morphology[45–47]. Inter-dataset variability can result from genetic variation, developmental noise and limitations in data quality or reconstruction.”

We draw the referee's attention to the Methods, where we describe this issue in more detail:

“A majority of mismatches were due to incomplete proofreading; as proofreading improves, matches made improves. The mismatched neuron was almost always a similar cell type within the same hemilineage. For the remaining neurons that could not be confidently matched, we have classified them based on gross morphology and identified their closest associated neurons in other datasets with NBLAST. We estimate that ~10% of these unmatched neurons will prove unmatchable due to reconstruction quality issues or developmental differences in neuron wiring. Notably, we estimate that as many as ~862 neurons of the VNC may be sexually dimorphic and/or sex-specific (A.M, C.K.S et al., in prep, drawing on the 2025 FlyWire VNC matching challenge: https://codex.flywire.ai/app/vnc_matching_challenge) and cannot be matched well to MANC (which is a VNC sample from a male fly).”

We also note:

“*In our review of the neck connective, we identified 31 ANs and DNs that appeared to have developed abnormally or were stochastic in whether they had an ascending/descending arbour. For example, DNge079 on the right side (in MANC named DNxl080) has a mis-targeted dendrite located in the VNC, rather than the central brain. However, we note that both the left and right IN08B003 neurons are ANs in this dataset, but are intrinsic neurons of the VNC in MANC and in FANC.”*

- Page 11, lines 391: If there is any reference testing that these motor muscles/neurons control all three axes of movement it would be great to add this here.

Response: The reference is Gorko et al., 2024, which is cited elsewhere and was omitted here by accident. We have corrected the citation.

Typos:

- Page 25, line 717: „removed“ is added twice in one sentence - „We removed the legs and proboscis removed to allow …“

Response: Thank you; we have now corrected this sentence.

- Extended Data Figure 1f: Typo in axis label - connections instead of connections; Different for sizes are used in the respective panels; Could you possibly shortly add to Figure 1b which parts of the nervous system are targeted in the respective projects (MANC and FAFB are mentioned in the text, but what is the difference to hemibrain and FANC?)

Response: Thank you, we corrected the typo in the Extended Data Figure axis. To help clarify the parts of the nervous system encompassed by the other datasets, we have added the following to our Methods section on cell type matching:

“We matched BANC neurons to reconstructions in FlyWire-FAFB v783[15,17]MANC v1.2.1[10], FANC v1116[7] and HemiBrain v.1.2.1[14]. FAFB is a dense reconstruction of the female brain. MANC is a dense reconstruction of the male ventral nerve cord. FANC is a female ventral nerve cord dataset, with part of the abdominal ganglion truncated, which has been sparsely reconstructed. HemiBrain is a dense reconstruction of ~⅓ of the female central brain, including a high-quality reconstruction of the mushroom body and central complex. Cell type names from FAFB were preferred for brain matches, from MANC for VNC matches, FANC for known female-specific[21] ANs and motor neurons[8] (where the

annotation is better than in MANC) and HemiBrain for the central complex[6] (where the annotation is better than in FAFB).”

We have also added a shorted version to the legend for Extended Data Figure 1e (formerly Extended Data Figure 1b):

“FAFB is a dense female brain reconstruction. MANC is a dense male VNC reconstruction. FANC is a sparse female VNC reconstruction. HemiBrain is a dense reconstruction of ~⅓ of the central brain.”

- Extended Data Figure 2e: there seems to be a mismatch in the alignment of the labels and the bars – there are only 14 bar-comparisons, but 21 labels

Response: Thank you; this was a plotting error. We have fixed it (now **Extended Data Figure 5h**).

- Extended Data Figure 3b: should this be „functionally assessed“?

Response: Thank you; it should have been. We have now updated this heading to be “characterized cell types” and note that they have been functionally characterized in the figure legend (now **Extended Data Fig. 6b**).

Referee #4 (Remarks on code availability):

The code is very well organised and documented but needs to be also archived on a permanent repository.

Response: Thank you for reviewing our code. See our first response, in answer to ‘Essential Revisions’.

Referee #5:

I co-reviewed this manuscript with one of the reviewers who provided the listed reports.

Response: Thank you!

We thank the reviewers for their time, effort, and constructive feedback. We appreciate that our previous revision addressed many of their comments and concerns and improved the manuscript. Their remaining concerns are summarized and followed by point-by-point responses below:

1. Clarify the dataset's proofreading status. To avoid confusion, we have refined language regarding the completeness of the dataset. We have updated the text and refer to the dataset as a densely reconstructed connectome to further distinguish it from a complete connectome.
2. Using a global threshold of the influence metric for analysis and visualization. To address this, we now use a single threshold across analyses. Though we note that only two specific analyses use an influence metric threshold; all other quantitative analyses do not exclude any data based on the influence metric.
3. Additional statistical testing. We have provided additional statistical tests for Fig. 2e, 3a, 6b,e and Extended Data Fig. 4c as well as new analyses and statistical tests in Extended Data Fig. 4b,d,e and 8b.

Reviewer comments are shown in black upright font. When the Reviewer quotes our previous responses, this is shown in black italicized font. Our new responses are in green.

Referees' comments:

Referee #1 (Remarks to the Author):

We thank the authors for their thorough clarification and manuscript changes in response to our comments. The additional analyses and figures have addressed many of our comments and improved the manuscript. We do have some remaining concerns outlined below. Note that we include some of the author's original comments from the rebuttal letter for clarity, followed by our responses.

1. Proofreading status.

The figure and text edits regarding proofreading status of BANC has made it easier to assess BANC as a resource for the community, and we are happy with the changes the authors have implemented. However, at multiple points during the manuscript, the authors refer to BANC as one of the first 'complete' connectomes. We appreciate 'complete' in this context is refers to it being a full central nervous system, as opposed to completeness of the connectome itself. However, in the text, especially in the Abstract and Discussion lines 638-639, it is not immediately apparent that complete refers to CNS. We think the authors should be more specific here in referring to this connectome as a CNS connectome to avoid confusion with completeness of reconstruction (with BANC being less complete in terms of reconstruction compared to other available connectomes).

Response: We appreciate that this could be confusing, and we have removed the word "complete" in this context. Please also note that, in this revision, we have increased the rates of proofreading completion, synapse completion, and cell type identification; all updates have now been pushed to Codex.

2. Adjusted influence metric.

Author's original comment:
For some analyses, having a principled threshold is useful. For example, to analyze numbers of 'body parts' influenced by ANs and DNs, we used ANs/DNs which have demonstrated effects on specific body parts (Extended Data Figure 5). Based on established published experimental data (e.g. optogenetic stimulation), we determined an adjusted influence of 17.15 (17.18 in the previous version; the change is due to an updated cell type label among our selected ANs/DNs) as a threshold for individual ANs/DNs influencing individual body parts (Extended Data Figure 5d,e). We think this is a very stringent threshold. We have now added the previously held-back elbow plot as Extended Data Fig. 5e. For our analyses on influence between larger pools of intrinsic neurons, i.e. between superclusters of ANs/DNs, we do not have a similarly biologically principled way to choose a threshold. Therefore, for Fig. 5, we simply use an adjusted influence threshold of the 85th percentile of Fig. 5a to plot Fig. 5b, so that the

diagram would be humanly interpretable: i.e. did not have too many lines, to avoid cognitively overloading the reader. The accompanying heatmap (Fig. 5a) provides the full data for the reader. We have now made our choice of threshold clear in the legend:
"Summary of the strongest adjusted influences between superclusters. Here, for clarity, we depict only those influences with an adjusted influence cut-off of at the 85th percentile, chosen for concise visual display, line weight binned by the adjusted influences fully shown in (a)."
For our new analysis of the relative influence of mushroom body and central complex on ANs and DNs (Fig. 6g), given sensory neurons provide a baseline for influence on intrinsic cells, we used a median influence value from sensory neurons (Fig. 6f) as a threshold for selecting cell types to include in Fig. 6g. The point of the analyses is to demonstrate that few cell types from these more "cognitive" regions have comparable influence to that from sensors, and so we feel here that this threshold is more appropriate.

Reviewer comment:
We appreciate that for each of these individual instances, the reason why each threshold was chosen is reasonable. However, adjusted influence is presented as a generally useful metric to enable comparison of relative influence in the paper. As such, we think it is confusing to choose 3 different ways of thresholding, while describing the reasons and thresholding metric is the figure legends. In addition, the methods section implies a general threshold was used, which is not the case.
We would recommend choosing a consistent method of thresholding across the manuscript.
Otherwise, it should be made clear to the reader that within context comparisons require comparing influence scores relatively. The methods used in the manuscript should be clearly described in text, explicitly mentioning the 3 different ways that threshold were determined for different analyses. This will also make the metric more useful for usage in different contexts and further analyses in the future.

Response: We acknowledge that this situation was potentially confusing. To summarize, the three cases noted by the Referee are (1) Fig. 6f,g, (2) Extended Data Fig. 5, and (3) Fig. 5b.

In response to the Referee's suggestion, we have now modified Fig. 6f,g so that it uses the same threshold used in Extended Data Fig. 5.

In Fig. 5, we do not exclude any data. We show all values in panel 5a, with no thresholding. Panel 5b is a schematic summarizing the strongest values (at or above the 85th percentile) from panel 5a. We cannot show all the values from 5a in a schematic because 5a reports 210 pairwise influence scores between superclusters, and we cannot schematize 210 interactions in a readable way. We do not use the schematic in 5b to make any claims; it has purely illustrative value. The point is to illustrate the diversity of the strongest interactions.

The referee says that a "methods section implies a general threshold was used". We think they might mean the value of the constant *c* used in our adjustment of the influence score. This is not a threshold, just an adjustment to make all of our scores non-negative, which makes it easier to work with them in general data analysis, and avoids misinterpretation from the reader that a negative influence score could mean "inhibition". Data was not thrown out based on this value.

3. Sensor-effector pairings based on Fig 2 and Ext. Fig. 4:

Author's original comment:
Thank you. Our statement only applies to body parts where sensor and effector pairing can be clearly made. For example, chordotonal organs of the body (Wheeler's organ, the metathoracic organ, etc) do not have any effector neurons local to their sensory neurons in the periphery. The claim we make here is that when sensors and effectors can be paired by gross body part, sensor influence flows mainly to its paired effector neurons. However, significant off-pair influence exists, which also motivates us to examine longer range connections.
We have updated Fig. 2e to make our point better, including making more explicit pairings, and to remove distracting information (making this clear in the legend). We now quantify and statistically test that the interaction between sensory neurons and effector neurons is significant with a two-way ANOVA. We have also added a new analysis in Fig. 2g, which shows that neurons upstream of effector neurons in the CNS form local modules by influence, but that

one step further, influences become more distributed. We use this observation to help motivate our subsequent focus on long-ranged connections (Fig. 2h) that reach across these modules.

Reviewer comment:
While the 2-way ANOVA for data shown in Fig 2e is useful to provide evidence for the general statement, it is difficult to evaluate this statement from a single sentence providing one p-value for a comparison of 110 x 23 subclasses. The data used for the statistical comparison and a post-hoc comparison of each group should be shown to evaluate whether this is generally the case for all pairings or whether there is a more heterogeneous effect.

Response: We agree that the ANOVA was not the best way to evaluate this statement. The Results statement in question is the following: "We found that most groups of effector neurons receive their strongest influence from sensors in the same body part." To support this statement, we now include a Wilcoxon rank-sum test showing that adjusted influence from sensors to effectors is significantly stronger within-a-body-part than across-body-parts.This difference is highly significant ($p = 4.53x10^{-10}$); this is now described in the legend for Fig. 2e.

Moreover, to evaluate whether this statement holds true across each of the 14 body parts, we have added Extended Data Fig. 4b, where we plot the adjusted influence on the effectors of each body part from sensory neurons of the same body part and of different body parts. For each body part, we perform a Holm-corrected Wilcoxon rank-sum test comparing these two groups, and we find that the influence from the same body part is significantly stronger for all 14 body parts ($p < 0.05$).

Data in Fig 2g is an interesting addition, however as for 2e it is difficult to evaluate this statement based on a heatmap and an overall ANOVA value. In addition, the statement made in text about influence becoming more distributed is not tested. The ANOVA result for Fig 2g only suggests there is a source x target interaction effect across the whole data set and does not specifically compare effectors to pre- or pre-pre-effectors. This comparison, and specifically the claim that influence becomes more distributed should be properly quantified.

Response: We have now added Extended Data Fig. 4e to show that influence from matched sensors significantly decreases with "hops" back from effectors (from pre to pre-pre, paired Wilcoxon signed-rank test, $p = 0.954x10^{-6}$).

Author's original comment:
Thank you for the suggestion. We feel that an intuitive way to do this would be to assign sensor-effector connections that must transit between ganglia (i.e. between the brain and VNC) as 'long-ranged', those within-ganglia (within either the brain or the VNC alone) as 'medium ranged', and those for which the sensors and the effectors are in the same body part as 'short ranged'. We have now provided this new analysis, Extended Data Fig. 4b, which quantifies the proportion of the total sensory influence onto each efferent neuron group (by body part) that falls into these three classes.

Reviewer comment:
This is a useful addition. However, the statement 'each local loop is influenced by a select group of more distant sensors in functionally related body parts (Fig. 2e, Extended Data Fig 4b)' is not sufficiently backed up by Fig 2e or Ext Data Fig 4b. Ext Data Figure 4b does not enable evaluation of whether more distant sensor are functionally related, and Fig 2e does not contain quantification. Especially given the size of the dataset and the fact that the heatmap shows an aggregate value across many sensors, it would be useful to see whether there is a statistically significant influence from more distant sensors onto some of the effectors.

Response: Agreed. When we state that "each local loop is influenced by a select group of more distant sensors in functionally related body parts", we mean this: if we look at the influences across body parts, those patterns of influence are selective (not uniform or nonselective), and these influences are stronger from related body parts. To support this statement, we have generated a new figure panel (Extended Data Fig. 4d), which shows that across-part influence grows with relatedness between body parts. Here, relatedness is measured as the cosine similarity between the sensory influences onto a body part. As noted in the legend to this figure, this correlation is highly significant. This result supports the idea that across-body-part patterns of influence are selective (not uniform), and they are stronger from related body parts. All the datapoints in Extended Data Fig. 4d are across-body-part, so they

are all “distant” (relative to same-body-part influences); this demonstrates that distant interactions are indeed significant and selective. Finally, we note that all the data in this new figure panel also appears in Fig. 2e; here, the reader can examine any body part combination that interests them. Fig. 2f also highlights the interactions among some related specific body parts, as an illustrative example of this trend. Our intention in Extended Data Fig. 4d is to ask whether the *overall* relationship governing “distant” (across-body-part) influence is statistically significant, which was the referee’s question, as we understand it.

Author original comment:
We show that the core elements of behavioral control are a set of local feedback modules, where effector neurons are strongly and specifically influenced by the sensors positioned to monitor the relevant effectors (Fig. 2e). We have now added a statistical test to the Fig. 2e legend to show that the pattern of sensor→effector influence is significantly different for each body part. This argues that sensory signals are unlikely to be routed through a centralized world representation. This result is compatible with both ecological theory and embodied cognition, but not with the classical theory. In a new analysis, we show that there are strong local connections between effector pathways (Fig. 2g). Note that the pattern of recurrent effector pathway influence is significantly different for each body part (Fig. 2g legend). The prominence of local effector connections further supports the embodied cognition model of neural function.

Reviewer comment:
As noted above, the statistics provided in the legend of Fig 2e and 2g are not sufficient to back up the statement that sensor to effector influence is significantly different for each body part.

Response: Please see our answers above, detailing the added statistical tests for the relevant figure panels and the supplements that support them.

4. AN/DN betweenness shown in Fig 3a

Author original comment:
In order to address this, we have conducted a new analysis on the relationship between ANs/DNs and sensory neuron-to-effector neuron indirect connectivity. Essentially, we quantify “bottleneck-ness” of all neurons, including ANs/DNs with node betweenness centrality (Freeman, 1977; Brandes, 2001; Brandes, 2008; Newman, 2018): for a neuron, its score is the fraction of all shortest paths between designated sources and targets that pass through that node. In our use, sources are sensory neurons and targets are effector neurons, so the score reflects how much a neuron could mediate sensor-to-effector communication. High‑betweenness neurons are bridges linking otherwise separate modules. Critically, a node can have low degree but high betweenness if it sits between communities. I.e. this metric captures a neuron’s potential control over global flow, not just its local connectivity. Fig. 3a now shows the result at a super-class level suggesting ANs and DNs are bottlenecks for sensory-to-effector information flow. Extended Data Fig. 4c also shows this extended across all proofread neurons as sources and targets, for which ANs and DNs remain among the most substantial bottlenecks for information flow in the CNS. We describe these new analyses in the Results:
“Thus far, we have seen evidence for strong local feedback loops, linked by selective longer-range sensor-effector connections. To better understand these long-range connections, we focused on the neurons that link the brain with the VNC, namely DNs and ANs. DNs and ANs participate in many sensor-to-effector connections: we can see this by finding the shortest paths from each sensory neuron to each effector neuron, and then computing the proportion of those paths that contain a given cell of interest. This value is relatively high for DNs and ANs (Fig. 3a, Extended Data Fig. 4c), which implies that DNs and ANs play key roles in bridging the brain and VNC to connect sensory neurons and with effector neurons.

Reviewer comment:
The legend of Figure 3a implies ANs and DNs had higher betweenness than all other super-classes but no direct comparison is shown. It is not clear from this test whether ANs and DNs have higher betweenness than other VNC intrinsic cells and the plot shows that they are at least comparable in value. This statistical analysis should be added and shown here.

The evidence provided here gives me reasonable confidence that ANs and DNs participate in many sensor-to-effector connections, however it should be noted that this also appears to be the case for other VNC intrinsic cell types.

Response: To support the statement that ANs and DNs have higher betweenness than VNC intrinsic neurons, we have added a Wilcoxon ranked-sum test comparing ANs and VNC intrinsic neurons ($p=1.68\times10^{-29}$) and another test comparing DNs and VNC intrinsic neurons ($p=2.09\times10^{-24}$). This now appears in the legend of Fig. 3a. We have also added Wilcoxon ranked-sum tests to the legend of Extended Data Fig. 7a (formerly, Extended Data Fig. 4c).

The referee correctly notes that VNC intrinsic neurons also have high betweenness. To make this more explicit for readers, we have added VNC intrinsic neurons to the post-hoc Dunn tests, which indeed show that they have higher betweenness than the remaining four super classes ($p \leq 1.5\times10^{-3}$). We now also explicitly comment on this in the Figure 3a legend:

> "VNC intrinsic neurons also have high betweenness because the VNC is enriched with sensory and effector neurons (35% of VNC neurons are sensory and 4% are effectors, compared with 16% and 0.5% in the central brain)."

5. Superclusters in Fig 4d

Author original comment:
Thank you for the helpful suggestion. We have quantified the cosine similarity of influence to and from ANs and DNs and tested input and output specialization by comparing influence related to ANs and DNs in the same cell type (highly similar), the same supercluster (less similar), and in different superclusters (dissimilar) in Fig. 4d. These results underscore that cells in the same supercluster are indeed diverse – more diverse than cells of the same type. At the same time, these results support the conclusion that cells of the same supercluster are more similar than cells of different superclusters.

Reviewer comment:
The additional quantification is useful. However, it is difficult to evaluate how similar or dissimilar some superclusters are given one summary comparison of all superclusters. It would also be useful to also show this comparison for each supercluster to provide some data on whether some superclusters may constitute a specific subcluster with higher confidence.

Response: We have now done the comparisons for each supercluster and present them in Extended Data Fig. 8b. This new analysis shows that for each supercluster, the similarity is highest for the same cell type, lower for the same supercluster, and lowest for different superclusters (with each difference being statistically significant, $p \leq 0.0001$). This confirms what we show for the combined comparison in Fig. 4d.

6. CNS networks in Fig 6

Author original comment:
Considering the network structure of the entire CNS, we found that many CNS networks have a high influence on effector neurons (Fig. 6b), further supporting the idea that behavioral control is distributed, rather than centralized. We found that the central complex and mushroom body have low effector influence overall (Fig. 6b), and high influence on only a small subset of DNs and ANs (Fig. 6f,g). Together, these findings imply that the central complex and mushroom body have a supervisory role, and they supervise a subset of behaviors, not all behaviors. This conclusion is compatible with the idea of embodied cognition (which posits a limited role for memories and other internal representations); it is also compatible with subsumption architecture, where high-level modules supervise low-level modules, but high-level modules are inessential for many behaviors. In short, our study provides many lines of evidence to support the idea of embodied cognition and specifically to support an implementation resembling subsumption architecture. Of course, it is possible that there is another theoretical framework that better explains the organization of the Drosophila CNS. If so, however, we think that theoretical framework does not yet exist. If the

referee would like us to discuss another specific alternative, we would be sincerely interested in hearing this suggestion.

Reviewer comment:
For the comparison in 6f, it would be useful to also include the other CNS networks considered in 6b, for which a statement is made that they have high influence on effectors based on the heatmap in 6b, but this is not tested using a statistical test.

Response: Fig. 6f does not use CNS networks from Fig. 6b. Rather, the CNS network analysis in Fig. 6b suggested that the central complex and mushroom body had distinctive properties. Therefore, to further analyze the outputs of these well-studied brain regions, we quantified their influence onto ANs and DNs (Fig. 6f). Specifically, we focused on how the output neurons of these brain regions influenced ANs and DNs, and we used sensory neurons and visual projection neurons for comparison, as they also influence ANs and DNs. We have revised the figure legend as follows to make it clearer that this panel does not directly analyze the CNS networks:

> "We took adjusted influence scores from four cell classes (sensory, visual projection, central complex output neuron, and mushroom body output neuron) onto each of the 1018 AN/DN cell types."

Regarding Fig. 6b, we have now included an additional statistical test supporting the claim that the central complex and olfactory networks have low influence on effectors:

> "Most CNS networks (10/13, 77%) have high influence (>median) on at least one effector group. Central complex, right olfactory, and left olfactory networks have zero connections above median (combined probability by chance: $p=1.8\times10^{-12}$, binomial test: $p=0.046$)."

Author original comment:
We did not intend to argue that the bridges between CNS networks are solely composed of ANs/DNs, and we apologize if our original text gave this impression. We have revised the relevant sections of the main text to clarify our findings. Specifically, in the revised manuscript we state that:
"many CNS networks … contain ANs and DNs (Extended Data Fig. 10b-c)"
"these links [between CNS networks] are disproportionately composed of DNs: when we counted each neuron's synaptic partners outside its assigned network, we found DNs had a relatively high proportion of outside partners (Fig. 6e)"
"Most AN/DN superclusters are divided between two or three CNS networks (Extended Data Fig. 10c), consistent with the notion that DNs often form bridges between networks."
We think the cited analyses provide clear support for our conclusions as stated here. We did consider excising ANs/DNs to see how this changes influence scores. The main result is that the VNC-dominated CNS networks lose much of their influence from brain-dominated CNS networks, and vice versa. However, this result is somewhat expected, in our view, and we are hesitant to make much of it in the manuscript.

Reviewer comment:

It is not clear to me what 'other' in Figure 6e represents; this should be added to the figure legend. The text implies that DNs have a higher proportion of out-of-network direct partners. A two-sample KS test is not an appropriate test to make this claim in this case, as the distributions of each cell type are clearly different. This is also demonstrated by the significant difference between ANs and 'other' despite the seemingly similar median and IQR values. The authors should add a non-parametric test that compares the central value of the distributions to make this claim.

Response: "Other" refers to any other neuron in a CNS network that is neither an AN nor a DN. We have now added this to the legend: "'Other' refers to any other neuron in a CNS network that is neither an AN nor DN."
Additionally, as the referee suggests, we have added the non-parametric Wilcoxon rank–sum test to the legend. Both DNs and ANs have statistically significantly higher proportions of out-of-network partners compared to "other" ($p<6.2\times10^{-90}$ for DNs and $p<3.6\times10^{-4}$ for ANs, Wilcoxon rank-sum test, Holm-adjusted $\alpha=0.05$).

Response to Referees

Referee #1 (Remarks to the Author):

We thank the authors for their changes in response to our comments. We have no additional concerns and recommend publication.

Referee #2 (Remarks to the Author):

I co-reviewed this manuscript with one of the reviewers who provided the listed reports.

We thank the Referees for the time and effort that went into evaluating our work. There are no further concerns to address.
