# Supplementary material for: Distributed control circuits across a brain-and-cord connectome
Source: Nature. 2026 Jun 8;656(8129):957–70. doi: 10.1038/s41586-026-10735-w (PMC13518251; doi:10.1038/s41586-026-10735-w)
Supplement: Supplementary file 2 [file 41586_2026_10735_MOESM1_ESM.pdf]

nature portfolio

https://doi.org/10.1038/s41586-026-10735-w

Supplementary information

Distributed control circuits across a brain-and-cord connectome

In the format provided by the authors and unedited

Supplementary Notes

Below please find (i) a table of resources generated by this work, (ii) tables of neuroglancer (for neuroanatomy) and FlyWire Codex (for network diagrams and annotation) links, related to our figures, and (iii) a detailed description of the columns of each of the Supplementary Data files.

(i)

General resources introduced by this work

Panel	Description	Links
**Codex**	Browse all BANC neurons, connectivity and annotations (circuit)	https://codex.flywire.ai/banc
**Neuroglancer**	Public-access BANC dataset view (neuroanatomy)	https://ng.banc.community/view
**Neuroglancer**	Live data — authenticated access; email corresponding authors for access (neuroanatomy)	https://ng.banc.community
**Harvard Dataverse**	Stable archival source for all data and code: morphologies, synapses, annotations, influence scores	https://doi.org/10.7910/DVN/7WTH1N
**Google Storage Bucket**	Live master copy of all bulk project data (gs://lee-lab_brain-and-nerve-cord-fly-connectome): cell annotations; synapse tables, connectivity and neurotransmitter predictions (v2/v3); NBLAST results; neuron meshes and L2 skeletons; colour-MIPs; influence scores including the	https://console.cloud.google.com/storage/browser/lee-lab_brain-and-nerve-cord-fly-connectome

	all-to-all matrix; template registrations; Neuroglancer states; behavioural data; and microCT. Continuously updated as the project evolves — the Harvard Dataverse holds the stable, citable archival copy.	
**BossDB**	Archived aligned image and flat segmentation data (DOI: 10.60533/boss-2025-941r)	https://bossdb.org/project/bates_phelps_kim_yang2025
**BANC-Project**	Analysis and figure-generation code for this manuscript — GitHub and Zenodo	GitHub: https://github.com/htem/BANC-project<br>Concept DOI (cite — latest): https://doi.org/10.5281/zenodo.20350641<br>Version DOI (v2.0.5): https://doi.org/10.5281/zenodo.20350642
**ConnectomeInfluenceCalculator**	Python code for computing influence scores — GitHub and Zenodo	GitHub: https://github.com/DrugowitschLab/ConnectomeInfluenceCalculator<br>Concept DOI (cite — latest): https://doi.org/10.5281/zenodo.15999929<br>Version DOI (v0.4): https://doi.org/10.5281/zenodo.17693838
**influencer**	R code for computing influence scores — GitHub and Zenodo	GitHub: https://github.com/natverse/influencer<br>Concept DOI (cite — latest): https://doi.org/10.5281/zenodo.20350563<br>Version DOI (v1.0.2): https://doi.org/10.5281/zenodo.20350564
**synister_banc**	Code for training and running the neurotransmitter prediction model — GitHub and Zenodo	GitHub: https://github.com/htem/synister_banc<br>Concept DOI (cite — latest): https://doi.org/10.5281/zenodo.20350569<br>Version DOI (v1.0.4): https://doi.org/10.5281/zenodo.20350570
**bancpipeline**	Data pre-processing pipeline post-proofreading: skeletonization, neuron matching, metadata,	GitHub: https://github.com/htem/bancpipeline<br>Concept DOI (cite — latest): https://doi.org/10.5281/zenodo.20350571<br>Version DOI (v2.0.5): https://doi.org/10.5281/zenodo.20350572

	axon–dendrite splitting, neuropil assignment — GitHub and Zenodo	
**bancr**	R client package (natverse-compatible) for querying and analysing the BANC — GitHub and Zenodo	GitHub: https://github.com/natverse/bancr<br>Concept DOI (cite — latest): https://doi.org/10.5281/zenodo.20350647<br>Version DOI (v0.3.5): https://doi.org/10.5281/zenodo.20350648
**nat.ggplot**	R package for neuroanatomical plots — GitHub and Zenodo	GitHub: https://github.com/natverse/nat.ggplot<br>Concept DOI (cite — latest): https://doi.org/10.5281/zenodo.20350565<br>Version DOI (v1.0.2): https://doi.org/10.5281/zenodo.20350566
**banc**	Python client package for querying and analysing the BANC — PyPI	https://pypi.org/project/banc/
**drosophila_neurotransmitters**	Ground-truth Drosophila neurotransmitter labels per cell type — GitHub and Zenodo (v1.1.0)	GitHub: https://github.com/flyconnectome/drosophila_neurotransmitters<br>Concept DOI (cite — latest): https://doi.org/10.5281/zenodo.20818141<br>Version DOI (v1.1.0): https://doi.org/10.5281/zenodo.20818142
**drosophila_neuropeptides**	Ground-truth Drosophila neuropeptide labels per cell type — GitHub and Zenodo (v1.1.0)	GitHub: https://github.com/flyconnectome/drosophila_neuropeptides<br>Concept DOI (cite — latest): https://doi.org/10.5281/zenodo.20818143<br>Version DOI (v1.1.0): https://doi.org/10.5281/zenodo.20818144
**the-BANC-fly-connectome**	Project code repository (annotation Slack bots and tools) — GitHub	https://github.com/jasper-tms/the-BANC-fly-connectome
**fly_connectome_data_tutorial**	Python + R tutorial bringing these tools and data together — GitHub	https://github.com/sjcabs/fly_connectome_data_tutorial
**Schematics**	Figure schematics as vector graphics (Wilson lab) — GitHub	https://github.com/wilson-lab/schematics

(ii)

Figure 1

Panel	Description	Links
**1b**	EM neck connective cross-section (neuroanatomy)	https://ng.banc.community/2026a/neck-connective
**1b**	Example mitochondria detection (neuroanatomy)	https://ng.banc.community/2026a/example-mitochondria
**1b**	Example nucleus detection (neuroanatomy)	https://ng.banc.community/2026a/example-nuclei
**1b**	Example postsynaptic detection (neuroanatomy)	https://ng.banc.community/2026a/example-synapse
**1d**	Cross-dataset matching, DNa02 (neuroanatomy + Codex search)	https://ng.banc.community/2026a/DNa02<br>Codex link
**1e**	Annotation hierarchy, LB1a (neuroanatomy + Codex search)	https://ng.banc.community/2026a/LB1a<br>Codex link
**1e**	Annotation hierarchy, DVM1a-c (neuroanatomy + Codex search)	https://ng.banc.community/2026a/DVM1a-c<br>Codex link

Figure 2

Panel	Description	Links
**2d**	Effector body parts (neuroanatomy + Codex browser)	https://ng.banc.community/2026a/efferent-body-parts<br>Codex link

Figure 3

Panel	Description	Links
**3c**	Example AN and DN with strong influence (neuroanatomy)	https://ng.banc.community/2026a/figure-3c<br>Codex link
**3d**	AN/DN super-clusters embedding (neuroanatomy)	https://ng.banc.community/2026a/an-dn-super-clusters
**3d**	Ascending neurons (neuroanatomy)	https://ng.banc.community/2026a/ascending
**3d**	Descending neurons (neuroanatomy)	https://ng.banc.community/2026a/descending
**3g**	Visceral-control circuit (neuroanatomy + Codex circuit)	https://ng.banc.community/2026a/figure-3g<br>Codex link
**3h**	Flight-power × visceral-control circuit (neuroanatomy + Codex circuit)	https://ng.banc.community/2026a/figure-3h<br>Codex link
**3i**	Visceral-sensing / reproduction circuit (neuroanatomy + Codex circuit)	https://ng.banc.community/2026a/figure-3i<br>Codex link
**3j**	Tactile-cluster circuit (neuroanatomy + Codex circuit)	https://ng.banc.community/2026a/figure-3j<br>Codex link
**3k**	Walking-cluster circuit (neuroanatomy + Codex circuit)	https://ng.banc.community/2026a/figure-3k<br>Codex link

Figure 4

Panel	Description	Links
**4b/4c**	Head-orienting cluster (neuroanatomy)	https://ng.banc.community/2026a/head-orienting Codex link
**4f**	Head-orienting example circuit (neuroanatomy + Codex circuit)	https://ng.banc.community/2026a/figure-4f Codex link
**4g**	Postural-control loop circuit (neuroanatomy + Codex circuit)	https://ng.banc.community/2026a/figure-4g Codex link

Figure 5

Panel	Description	Links
**5c**	Cross-cluster interaction circuit (neuroanatomy + Codex circuit)	https://ng.banc.community/2026a/figure-5c Codex link

Figure 6

Panel	Description	Links
**6h**	Mushroom-body → AN/DN circuit (neuroanatomy + Codex circuit)	https://ng.banc.community/2026a/figure-6h-mb Codex link
**6h**	Central-complex → AN/DN circuit (neuroanatomy + Codex circuit)	https://ng.banc.community/2026a/figure-6h-cx Codex link

Extended Data Fig. 8a (AN/DN cluster morphologies — Neuroglancer scenes)

Panel	Description	Links
**flight power**	Cluster morphology (neuroanatomy)	https://ng.banc.community/2026a/flight-power
**flight steering 1**	Cluster morphology (neuroanatomy)	https://ng.banc.community/2026a/flight-steering-1
**flight steering 2**	Cluster morphology (neuroanatomy)	https://ng.banc.community/2026a/flight-steering-2
**head orienting**	Cluster morphology (neuroanatomy)	https://ng.banc.community/2026a/head-orienting
**postural control**	Cluster morphology (neuroanatomy)	https://ng.banc.community/2026a/postural
**threat response**	Cluster morphology (neuroanatomy)	https://ng.banc.community/2026a/threat-response
**proprioceptive**	Cluster morphology (neuroanatomy)	https://ng.banc.community/2026a/proprioceptive-perception
**tactile**	Cluster morphology (neuroanatomy)	https://ng.banc.community/2026a/tactile-perception
**reproduction**	Cluster morphology (neuroanatomy)	https://ng.banc.community/2026a/mating-and-reproduction
**feeding**	Cluster morphology (neuroanatomy)	https://ng.banc.community/2026a/feeding
**taste-touch**	Cluster morphology (neuroanatomy)	https://ng.banc.community/2026a/taste-touch
**visceral control**	Cluster morphology (neuroanatomy)	https://ng.banc.community/2026a/visceral-state
**probing**	Cluster morphology (neuroanatomy)	https://ng.banc.community/2026a/probing

**grooming**	Cluster morphology (neuroanatomy)	https://ng.banc.community/2026a/grooming
**walking**	Cluster morphology (neuroanatomy)	https://ng.banc.community/2026a/walking
**walking steering**	Cluster morphology (neuroanatomy)	https://ng.banc.community/2026a/walking-steering

(iii)

Supplementary Data 1: Metadata categories and terms
Table of categories of annotations applied to BANC neurons and the list of terms used in each category. For region, side, flow, super_class, cell_class, cell_sub_class, cell_type, and hemilineage, only one term applies per neuron. For the other categories, neurons can be labeled with more than one term. We do not include in this table all the possible options for cell_type, other_names, fafb_783_cell_type, manc_121_cell_type, fanc_1116_cell_type, hemibrain_121_cell_type, malecns_09_cell_type, fafb_783_match_id, manc_121_match_id, fanc_1116_match_id, hemibrain_121_match_id and malecns_09_match_id because there are too many possible options, but we include the categories and their descriptions to document them. For metadata annotations for each BANC neuron, see the data sources described in Data Availability.

- flow — from the perspective of the whole CNS, whether the neuron is afferent, efferent, or intrinsic
- super_class — coarse division, hierarchical below flow
- cell_class — hierarchical below super_class
- cell_sub_class — hierarchical below cell_class
- cell_type — the name of the matched neuron from FAFB if it is a brain neuron or a DN or the name of the matched neuron from MANC if it is a VNC neuron or an AN. There are a few exceptions where those names did not define single cell types and were further split. This is hierarchical below cell_sub_class
- region — region of the CNS–optic lobe, central brain or ventral nerve cord–based on soma or entry/exit nerve location
- side — from the fly's perspective, the side on which the cell body is located or for afferent neurons, the side of the entry nerve.
- cell_function — term briefly describing the function of the neuron, applied largely to afferent and efferent neurons
- cell_function_detailed — more detailed information for the function of the neuron than cell_function, also applied largely to afferent and efferent neurons
- peripheral_target_type — the sensor or effector structure/organ targeted by an afferent/efferent neuron.
- body_part_sensory — the part of the body innervated by an afferent neuron
- body_part_effector — the part of the body targeted by an effector neuron. If known, this is the site of action when it is different from the body part innervated (e.g., wing power motor neurons innervate muscles located in the thorax but move the wing)
- nerve — peripheral nerve (if applicable)
- hemilineage — developmental lineage (NA for many neurons)
- sexually_dimorphic — isomorphic, dimorphic, or female-specific for whether the neuron is sexually dimorphic/sex-specific
- neurotransmitter_verified/neuropeptide_verified — neurotransmitter/neuropeptide of neuron, as reported in the literature
- neurotransmitter_predicted — CNN-predicted primary neurotransmitter
- other_names — names given to the neuron that are not the cell_type name
- alignment_cell_type — cell types from our NBLAST-to-connectivity annealing algorithm
- fafb_783_cell_type/manc_121_cell_type/fanc_1116_cell_type/hemibrain_121_cell_type/malecns_09_cell_type — cell type of neuron from FAFB v783/MANC v1.2.1/FANC v1116/hemibrain v1.2.1/maleCNS v0.9 that matches the BANC neuron
- fafb_783_match_id/manc_121_match_id/fanc_1116_match_id/hemibrain_121_match_id/malecns_09_match_id — segment ID of neuron from FAFB v783/MANC v1.2.1/FANC v1116/hemibrain v1.2.1/maleCNS v0.9 that matches the BANC neuron

Supplementary Data 2: BANC neuron metadata
Per-neuron metadata for the proofread/roughly proofread neurons in our BANC release, with cross-dataset match identifiers to FAFB, MANC, hemibrain, FANC, and maleCNS where assigned. This is the reference table for every BANC analysis in the paper.

- root_id — BANC v888 neuron identifier
- dataset — "BANC" (constant; included so this table can be concatenated with Supplementary Data 3-5)
- Columns defined in Supplementary Data 1:
    - flow — from the perspective of the whole CNS, whether the neuron is afferent, efferent or intrinsic
    - super_class — coarse division, hierarchical below flow
    - cell_class — hierarchical below super_class
    - cell_sub_class — hierarchical below cell_class
    - cell_type — the name of the matched neuron from FAFB if it is a brain neuron or a DN or the name of the matched neuron from MANC if it is a VNC neuron or an AN. There are a few exceptions where those names did not define single cell types and were further split. This is hierarchical below cell_sub_class
    - alignment_cell_type — cell types from our NBLAST-to-connectivity annealing algorithm
    - region — region of the CNS–optic lobe, central brain or ventral nerve cord–based on soma or entry/exit nerve location
    - side — from the fly's perspective, the side on which the cell body is located or for afferent neurons, the side of the entry nerve.
    - cell_function — term briefly describing the function of the neuron, applied largely to afferent and efferent neurons
    - cell_function_detailed — more detailed information for the function of the neuron than cell_function, also applied largely to afferent and efferent neurons
    - peripheral_target_type — the sensor or effector structure/organ targeted by an afferent/efferent neuron.
    - body_part_sensory — the part of the body innervated by an afferent neuron
    - body_part_effector — the part of the body targeted by an effector neuron. If known, this is the site of action when it is different

from the body part innervated (e.g., wing power motor neurons innervate muscles located in the thorax but move the wing)
    - nerve — peripheral nerve (if applicable)
    - hemilineage — developmental lineage (NA for many neurons)
    - sexually_dimorphic — isomorphic, dimorphic, or female-specific for whether the neuron is sexually dimorphic/sex-specific
    - neurotransmitter_verified/neuropeptide_verified — neurotransmitter/neuropeptide of neuron, as reported in the literature
    - neurotransmitter_predicted — CNN-predicted primary neurotransmitter
    - other_names — names given to the neuron that are not the cell_type name
    - fafb_783_match_id/manc_121_match_id/fanc_1116_match_id/hemibrain_121_match_id/malecns_09_match_id — segment ID of neuron from FAFB v783/MANC v1.2.1/FANC v1116/hemibrain v1.2.1/maleCNS v0.9 that matches the BANC neuron

Supplementary Data 3: Updated FAFB-FlyWire neuron metadata
Per-neuron metadata for the FAFB-FlyWire v783 neurons used in BANC's cross-dataset comparisons. We re-annotated several columns (super_class, cell_class, cell_sub_class, body_part_*, cell_function) to follow BANC conventions; cell_type names are unchanged from the FAFB project.

- root_783 — FlyWire neuron ID (FAFB v783)
- dataset — "FAFB"
- Columns defined in Supplementary Data 1:
    - flow — from the perspective of the whole CNS, whether the neuron is afferent, efferent or intrinsic
    - super_class — coarse division, hierarchical below flow
    - cell_class — hierarchical below super_class
    - cell_sub_class — hierarchical below cell_class
    - cell_type — the name of the matched neuron from FAFB if it is a brain neuron or a DN or the name of the matched neuron from MANC if it is a VNC neuron or an AN. There are a few exceptions where those names did not define single cell types and were further split. This is hierarchical below cell_sub_class
    - alignment_cell_type — cell types from our NBLAST-to-connectivity annealing algorithm
    - region — region of the CNS–optic lobe, central brain or ventral nerve cord–based on soma or entry/exit nerve location
    - side — from the fly’s perspective, the side on which the cell body is located or for afferent neurons, the side of the entry nerve.
    - cell_function — term briefly describing the function of the neuron, applied largely to afferent and efferent neurons
    - cell_function_detailed — more detailed information for the function of the neuron than cell_function, also applied largely to afferent and efferent neurons
    - body_part_sensory — the part of the body innervated by an afferent neuron
    - body_part_effector — the part of the body targeted by an effector neuron. If known, this is the site of action when it is different from the body part innervated (e.g., wing power motor neurons innervate muscles located in the thorax but move the wing)
    - nerve — peripheral nerve (if applicable)
    - hemilineage — developmental lineage (NA for many neurons)
    - neurotransmitter_verified/neuropeptide_verified — neurotransmitter/neuropeptide of neuron, as reported in the literature
    - neurotransmitter_predicted — CNN-predicted primary neurotransmitter
    - other_names — names given to the neuron that are not the cell_type name

Supplementary Data 4: Updated MANC neuron metadata
Per-neuron metadata for the 49,052 VNC neurons from MANC v1.2.1, with super_class / cell_class / cell_sub_class / body_part_* / cell_function aligned to BANC conventions. cell_type names are unchanged from the MANC project.

- bodyid — MANC neuron ID (v1.2.1)
- dataset — "MANC"
- Columns defined in Supplementary Data 1:
    - flow — from the perspective of the whole CNS, whether the neuron is afferent, efferent or intrinsic
    - super_class — coarse division, hierarchical below flow
    - cell_class — hierarchical below super_class
    - cell_sub_class — hierarchical below cell_class
    - cell_type — the name of the matched neuron from FAFB if it is a brain neuron or a DN or the name of the matched neuron from MANC if it is a VNC neuron or an AN. There are a few exceptions where those names did not define single cell types and were further split. This is hierarchical below cell_sub_class
    - alignment_cell_type — cell types from our NBLAST-to-connectivity annealing algorithm
    - region — region of the CNS–optic lobe, central brain or ventral nerve cord–based on soma or entry/exit nerve location
    - side — from the fly’s perspective, the side on which the cell body is located or for afferent neurons, the side of the entry nerve.
    - cell_function — term briefly describing the function of the neuron, applied largely to afferent and efferent neurons
    - cell_function_detailed — more detailed information for the function of the neuron than cell_function, also applied largely to afferent and efferent neurons
    - body_part_sensory — the part of the body innervated by an afferent neuron
    - body_part_effector — the part of the body targeted by an effector neuron. If known, this is the site of action when it is different from the body part innervated (e.g., wing power motor neurons innervate muscles located in the thorax but move the wing)
    - nerve — peripheral nerve (if applicable)
    - hemilineage — developmental lineage (NA for many neurons)
    - neurotransmitter_verified/neuropeptide_verified — neurotransmitter/neuropeptide of neuron, as reported in the literature
    - neurotransmitter_predicted — CNN-predicted primary neurotransmitter
    - other_names — names given to the neuron that are not the cell_type name

Supplementary Data 5: maleCNS neuron metadata
Per-neuron metadata for the neurons in the male CNS v0.9 release. Cell typing is all from the maleCNS project, but annotations have been aligned to the conventions of the BANC, to help users compare and co-analyze these datasets.

- malecns_09_id — maleCNS neuron ID (v0.9)
- dataset — "maleCNS"
- Columns defined in Supplementary Data 1:
  - flow — from the perspective of the whole CNS, whether the neuron is afferent, efferent or intrinsic
  - super_class — coarse division, hierarchical below flow
  - cell_class — hierarchical below super_class
  - cell_sub_class — hierarchical below cell_class
  - cell_type — the name of the matched neuron from FAFB if it is a brain neuron or a DN or the name of the matched neuron from MANC if it is a VNC neuron or an AN. There are a few exceptions where those names did not define single cell types and were further split. This is hierarchical below cell_sub_class
  - alignment_cell_type — cell types from our NBLAST-to-connectivity annealing algorithm
  - region — region of the CNS–optic lobe, central brain or ventral nerve cord–based on soma or entry/exit nerve location
  - side — from the fly's perspective, the side on which the cell body is located or for afferent neurons, the side of the entry nerve.
  - cell_function — term briefly describing the function of the neuron, applied largely to afferent and efferent neurons
  - cell_function_detailed — more detailed information for the function of the neuron than cell_function, also applied largely to afferent and efferent neurons
  - body_part_sensory — the part of the body innervated by an afferent neuron
  - body_part_effector — the part of the body targeted by an effector neuron. If known, this is the site of action when it is different from the body part innervated (e.g., wing power motor neurons innervate muscles located in the thorax but move the wing)
  - nerve — peripheral nerve (if applicable)
  - hemilineage — developmental lineage (NA for many neurons)
  - neurotransmitter_verified/neuropeptide_verified — neurotransmitter/neuropeptide of neuron, as reported in the literature
  - neurotransmitter_predicted — CNN-predicted primary neurotransmitter
  - other_names — names given to the neuron that are not the cell_type name

Supplementary Data 6: ANs and DNs with PCA-UMAP coordinates and cluster assignments
Per-neuron metadata for the ANs and DNs included in the connectivity-PCA-UMAP analysis (Fig. 3d, Extended Data Fig. 6), with cluster assignments.

- id — BANC v888 root_id
- UMAP1, UMAP2 — 2D embedding coordinates from connectivity-based PCA-UMAP analysis
- supervoxel_id, position — supervoxel ID and 3D coordinates (BANC raw voxel space)
- side — soma side (or entry-nerve side for afferents)
- region — region of the CNS–optic lobe, central brain or ventral nerve cord–based on soma or entry/exit nerve location
- super_class — ascending or descending (intrinsic neurons only)
- hemilineage — developmental lineage
- cell_function — functional role (literature review)
- nerve — peripheral nerve (if applicable)
- cell_type — the name of the matched neuron from FAFB if it is a brain neuron or a DN or the name of the matched neuron from MANC if it is a VNC neuron or an AN. There are a few exceptions where those names did not define single cell types and were further split. This is hierarchical below cell_sub_class
- fafb_783_cell_type/manc_121_cell_type — cell type of neuron from FAFB v783/MANC v1.2.1 that matches the BANC neuron
- fafb_cell_type, manc_cell_type — corresponding cell types in FAFB / MANC
- super_cluster — named AN/DN cluster used in figures (e.g., "head orienting", "walking", "postural control")
- cns_network — CNS network from the spectral clustering (where applicable)

Supplementary Data 7: Effector neurons with UMAP coordinates and functional cluster assignments
Per-neuron metadata for the efferent neurons (motor + visceral/circulatory) in the connectivity-UMAP analysis (Extended Data Fig. 4g), with cluster assignments.

- id — BANC v888 root_id
- UMAP1, UMAP2 — 2D embedding coordinates from connectivity-based UMAP analysis
- supervoxel_id, position — supervoxel ID and 3D coordinates (BANC raw voxel space)
- side — soma side (or entry-nerve side for afferents)
- region — region of the CNS–optic lobe, central brain or ventral nerve cord–based on soma or entry/exit nerve location
- flow — efferent
- super_class — motor / visceral_circulatory
- hemilineage — developmental lineage
- cell_function — functional role (e.g., leg_motor, antenna_motor, neck_motor)
- nerve — peripheral nerve
- cell_type, fafb_cell_type, manc_cell_type — BANC and cross-dataset cell types
- cluster — cluster ID with EFF_ prefix (e.g., EFF_01)
- super_cluster — named effector group used in figures (e.g., "front leg", "flight-steering", "abdomen-ureter")

Supplementary Data 8: CNS network analysis with spectral clustering and UMAP embedding
Per-neuron metadata for the 50,568 intrinsic neurons that entered the CNS-network spectral clustering analysis (Fig. 6a, Extended Data Fig. 10). Spectral clustering parameters: min connection strength = 1, cluster count = 13, cluster seed = 10, embedding seed = 3 (banc_888).

- root_id — BANC v888 root_id
- UMAP1, UMAP2 — 2D embedding coordinates (UMAP on the connectivity Laplacian)
- supervoxel_id, position — supervoxel ID and 3D coordinates (BANC raw voxel space)
- region — region of the CNS–optic lobe, central brain or ventral nerve cord–based on soma or entry/exit nerve location

- side — from the fly’s perspective, the side on which the cell body is located or for afferent neurons, the side of the entry nerve.
- super_class — coarse division, hierarchical below flow
- hemilineage — developmental lineage
- cell_function — term briefly describing the function of the neuron (where known)
- cell_type, fafb_cell_type, manc_cell_type — BANC and cross-dataset cell types
- cluster — AN/DN cluster, splitting ANs from DNs, or effector cluster (EFF_ prefix) where applicable
- super_cluster — named AN/DN cluster or effector group (where applicable)
- cns_network — assigned CNS network from spectral clustering (e.g., "central complex related", "abdominal VNC", "left olfactory")

Supplementary Data 9: Literature review on cell function for ascending, descending and visual projection neurons
Curated list of literature-validated functional roles used to interpret the AN/DN clusters and visual-projection influences

- cell_type — BANC cell type
- other_names — alternative names used in the literature
- super_class — ascending / descending / visual_projection
- cell_function — short functional label
- citations — short-hand citation key(s) for the work that established the function
- doi — DOI of the citation (where available)

Supplementary Data 10: Bounding boxes for known dataset artifacts that negatively impact neuronal reconstruction
22 bounding boxes delineating regions with known data-quality issues in the BANC dataset. Coordinates are in BANC raw-voxel space (1 voxel = 4 × 4 × 45 nm).

- issue — short informal label naming the issue type (e.g., "tunnel of death", "T2 blowout", "T1 soup", "champagne patch", "left VLP blowout", "dorsal CB wavy patch", "dorsal esophageal crush", "butt wiggle")
- min_x, min_y, min_z — lower corner of the bounding box (voxels)
- max_x, max_y, max_z — upper corner of the bounding box (voxels)
