# Supplementary material for: Distributed control circuits across a brain-and-cord connectome
Source: Nature. 2026 Jun 8;656(8129):957–70. doi: 10.1038/s41586-026-10735-w (PMC13518251; doi:10.1038/s41586-026-10735-w)
Supplement: Supplementary file 3 [file 41586_2026_10735_MOESM2_ESM.pdf]

nature portfolio

Corresponding author(s): Alexander Bates, Jasper Phelps, Mala Murthy, Jan Drugowitsch, Rachel Wilson, Wei-Chung Allen Lee

Last updated by author(s): May 22, 2026

Reporting Summary

Nature Portfolio wishes to improve the reproducibility of the work that we publish. This form provides structure for consistency and transparency in reporting. For further information on Nature Portfolio policies, see our Editorial Policies and the Editorial Policy Checklist.

Statistics

For all statistical analyses, confirm that the following items are present in the figure legend, table legend, main text, or Methods section.

n/a	Confirmed	
☐	☒	The exact sample size (*n*) for each experimental group/condition, given as a discrete number and unit of measurement
☐	☒	A statement on whether measurements were taken from distinct samples or whether the same sample was measured repeatedly
☐	☒	The statistical test(s) used AND whether they are one- or two-sided<br>*Only common tests should be described solely by name; describe more complex techniques in the Methods section.*
☒	☐	A description of all covariates tested
☐	☒	A description of any assumptions or corrections, such as tests of normality and adjustment for multiple comparisons
☐	☒	A full description of the statistical parameters including central tendency (e.g. means) or other basic estimates (e.g. regression coefficient) AND variation (e.g. standard deviation) or associated estimates of uncertainty (e.g. confidence intervals)
☐	☒	For null hypothesis testing, the test statistic (e.g. F, t, r) with confidence intervals, effect sizes, degrees of freedom and P value noted<br>*Give P values as exact values whenever suitable.*
☒	☐	For Bayesian analysis, information on the choice of priors and Markov chain Monte Carlo settings
☒	☐	For hierarchical and complex designs, identification of the appropriate level for tests and full reporting of outcomes
☐	☒	Estimates of effect sizes (e.g. Cohen's *d*, Pearson's *r*), indicating how they were calculated

Our web collection on statistics for biologists contains articles on many of the points above.

Software and code

Policy information about availability of computer code

Data collection

EM images were acquired using custom code for TEM imaging (https://github.com/htem/GridTapeStage).

Data analysis

EM data processing: EM images were first stitched and aligned using AlignTK v1.0.2 (https://mmbios.pitt.edu/aligntk-home). Alignment was further refined using custom code (https://github.com/seung-lab/corgie; https://github.com/seung-lab/metroem; https://github.com/seung-lab/SEAMLeSS).

Cell and organelle segmentation: We used custom code to segment the EM dataset into cells and fragments of cells (https://github.com/seung-lab/DeepEM; https://github.com/seung-lab/chunkflow; https://github.com/seung-lab/abiss; https://github.com/seung-lab/seuron).

Synapse prediction: We used custom code to predict synapse locations (https://github.com/seung-lab/Synaptor/).

Template registration: We registered the synapse density map of the EM dataset to the JRC 2018 Female brain and JRC 2018 Female VNC templates separately using elastix v5.2 (https://elastix.lumc.nl/).

Proofreading: We used Neuroglancer and CAVE to perform cell segmentation proofreading, annotation, and pulling data for analysis (https://github.com/google/neuroglancer; https://caveconnectome.github.io/)

Morphological cell-type matching: We used NBLAST v1.6.7 for quantitative cell type matching (https://natverse.org/nat.nblast/).

A comprehensive collection of community tools and software packages for working with the BANC dataset can be found at the project hub (https://banc.community) and the FlyWire Apps portal (https://flywire.ai/apps). All code developed for this project is open-source, publicly

available and archived on the Harvard Dataverse (DOI: https://doi.org/10.7910/DVN/7WTH1N). This archive contains the following repos, also hosted on Github/Zenodo: The specific code used to perform the analyses and generate the figures for this manuscript (BANC-Project, https://doi.org/10.5281/zenodo.20350642); our data pre-processing pipeline post-proofreading for the BANC, including skeletonization, neuron matching pipeline, neuron matching algorithms, meta management, axon-dendrite splitting, neuropil assignment to synapses, etc (bancpipeline, https://doi.org/10.5281/zenodo.20350572); python code for computing influence scores (ConnectomeInfluenceCalculator, https://doi.org/10.5281/zenodo.15999929); R code for computing influence scores (influencer, https://doi.org/10.5281/zenodo.20350564); code for training and running the neurotransmitter prediction model (synister_banc, https://doi.org/10.5281/zenodo.20350570); python client code for querying/analysing the BANC (banc, https://pypi.org/project/banc/); an R client package compatible with the natverse for querying/analyzing the BANC (bancr, https://doi.org/10.5281/zenodo.20350648); an R package for neuroanatomical plots (nat.ggplot, https://doi.org/10.5281/zenodo.20350566); a python + R tutorial for fly connectome data analyses that brings many of these tools and data together (fly_connectome_data_tutorial, https://github.com/sjcabs/fly_connectome_data_tutorial)

ColorMIP generation: We generated color-depth maximum intensity projections (colorMIPs) of all proofread neurons using the BANC python package (banc, https://pypi.org/project/banc/)

Spectral clustering: We adapted an algorithm for spectral clustering (/python/spectral_clustering in BANC-Project https://doi.org/10.5281/zenodo.20350642)

Additional data analysis: Through this manuscript, we clustered heatmaps using hierarchical clustering based on Ward's distance using functions from base R v4.2.1. We applied dynamic tree cut (implemented as dynamicTreeCut::cutreeDynamic, using deepSplit = 4) clustering to UMAPs to delineate effector and AN/DN clusters, other than in Fig. 6 and Extended Data Fig. 10, in which spectral clustering was used, see above. We conducted data analysis in R using the uwot, tidyverse, igraph and ggplot2 packages (see versions below). We made the Kernel density estimates for Fig. 6a using MASS::kde2d (see version below), n=100, cubes with densities above the first percentile colored. We calculated cosine similarity using the lsa R package (see version below), and we applied it to direct connectivity between BANC neurons to build the space used in Fig. 3. To perform the Kolmogorov-Smirnov test in Fig. 6e,f, the two-way ANOVA tests in Fig. 2g, 5a, 6b, and Kruskal-Wallis tests in Fig. 3a and Extended Data Fig. 5a, we used their respective statistical functions using the car and stats packages in R. P-values for non-parametric Wilcoxon rank-sum and signed-rank tests (Fig. 2e, 3a, 4a, Extended Data Fig. 4c, 4f, and 8a) were obtained using the wilcox.test function in base R. (https://github.com/htem/BANC-project/; https://doi.org/10.7910/DVN/7WTH1N).

List of external software and package versions below:
R v4.2.1
#R packages
arrow v16.1.0
bit64 v4.6.0.1
data.table v1.16.2
DBI v1.3.0
RSQLite v 2.4.6
readr v2.1.5
readxl v1.4.1
vroom v1.6.5
jsonlite v2.0.0
dplyr v1.2.1
forcats v0.5.2
glue v1.8.0
plyr v1.8.9
purrr v1.2.1
reshape2 v1.4.4
rlang v1.1.7
snakecase v0.11.1
stringr v1.6.0
tibble v3.3.1
tidyr v1.3.1
tidytext v0.3.4
tidyverse v1.3.2
ggplot2 v4.0.0.9000
ggimage v0.3.3
ggnewscale v0.5.2.9000
ggpubr v0.6.0.999
ggraph v2.2.1
ggrepel v0.9.5
ggsignif v0.6.4
ggsvg v0.1.13
gridExtra v2.3
nat.ggplot v0.1.0
pheatmap v1.0.12
plotly v4.11.0
scales v1.4.0
RColorBrewer v1.1.3
extrafont v0.18
Rttf2pt1 v1.3.11
flextable v0.8.2
gt v0.7.0
knitr v1.51
officer v0.4.4
openxlsx v4.2.5.1
car v3.1.2

carData v3.0.5
dendextend v1.17.1
dynamicTreeCut v1.63.1
effectsize v0.8.1
emmeans v1.8.2
kernlab v0.9.31
lsa v0.73.3
MASS v7.3.58.1
mclust v6.0.1
Matrix v1.6.1.1
matrixStats v0.62.0
proxy v0.4.27
rstatix v0.7.2
seriation v1.4.0
uwot v0.1.14
vegan v2.6.4
igraph v1.5.1
RSpectra v0.16.1
tidygraph v1.2.3
alphashape3d v1.3.1
concaveman v1.1.0
misc3d v0.9.1
nabor v0.5.0
Rvcg v0.22.2
nat v1.11.0
nat.flybrains v1.8.2
nat.nblast v1.6.7
nat.templatebrains v1.2.1
fafbseg v0.15.5
malevnc v0.3.3
bancr v0.3.0
influencer v0.1.0
reticulate v1.34.0
curl v7.0.0
googledrive v2.1.1
googlesheets4 v1.1.1
crosstalk v1.2.0
DT v0.26
htmltools v0.5.9
htmlwidgets v1.6.4
magick v2.7.3
webshot2 v0.1.1
callr v3.7.6
checkmate v2.3.4
devtools v2.4.5
parallel v4.2.1
pbapply v1.7.4
Progress v1.2.3
R.utils v2.12.2
usethis v3.2.1

Python v3.12.2
Python packages
numpy v2.0.2
pandas v2.2.3
scipy v1.17.0
matplotlib v3.10.8
scikit-learn v1.8.0
python-igraph v1.0.0
umap-learn v0.5.11
caveclient v8.0.1
cloud-volume v12.9.2
gcsfs v2025.12.0

For manuscripts utilizing custom algorithms or software that are central to the research but not yet described in published literature, software must be made available to editors and reviewers. We strongly encourage code deposition in a community repository (e.g. GitHub). See the Nature Portfolio guidelines for submitting code & software for further information.

Data

Policy information about availability of data

All manuscripts must include a data availability statement. This statement should provide the following information, where applicable:

- Accession codes, unique identifiers, or web links for publicly available datasets
- A description of any restrictions on data availability
- For clinical datasets or third party data, please ensure that the statement adheres to our policy

Data is freely accessible through multiple platforms. A general overview of the resource and links to these tools are available at the BANC portal (https://banc.community). FlyWire Codex (https://codex.flywire.ai/banc) provides an interactive web interface for exploring the BANC connectome, enabling users to search for neurons, visualize morphology, traverse synaptic pathways and download metadata such as cell type annotations, neurotransmitter predictions, and connectivity matrices. Volumetric EM data, including 3D neuron meshes and annotations, can be viewed at https://ng.banc.community/view or accessed programmatically via CAVE28. We used CAVE materialization v626 (July 21, 2025) for our preprint, and v888 (April 17, 2026) for the final print version. Static data and code dumps are also available for download from a DOI-minting repository, the Harvard Dataverse (DOI: https://doi.org/10.7910/DVN/7WTH1N) and on a Google Storage Bucket (console.cloud.google.com/storage/browser/lee-lab_brain-and-nerve-cord-fly-connectome/). Note that this Harvard Dataverse version supersedes our previous Harvard Dataverse for the preprint211, v626 (https://doi.org/10.7910/DVN/8TFGGB). The Google Storage Bucket can be updated as the BANC project evolves. Direct downloads from the Harvard Dataverse and Google Storage Bucket include: the synaptic connectivity edgelist, NBLAST results of BANC neurons against hemibrain, FAFB, FANC, and MANC as well as BANC all-by-all; neuronal L2 skeletons (made using: https://github.com/CAVEconnectome/pcg_skel); neuronal colorMIPs; influence scores; our aligned BANC metadata; template registrations; behavioral data for the BANC fly; and snapshots of our code. Aligned image data is available on BossDB (DOI: https://doi.org/10.60533/boss-2025-941r, or URL: https://bossdb.org/project/bates_phelps_kim_yang2025). Schematics are available here as vector graphics: https://github.com/wilson-lab/schematics?tab=readme-ov-file.

Research involving human participants, their data, or biological material

Policy information about studies with human participants or human data. See also policy information about sex, gender (identity/presentation), and sexual orientation and race, ethnicity and racism.

Reporting on sex and gender	N/A
Reporting on race, ethnicity, or other socially relevant groupings	N/A
Population characteristics	N/A
Recruitment	N/A
Ethics oversight	N/A

Note that full information on the approval of the study protocol must also be provided in the manuscript.

Field-specific reporting

Please select the one below that is the best fit for your research. If you are not sure, read the appropriate sections before making your selection.

☒ Life sciences ☐ Behavioural & social sciences ☐ Ecological, evolutionary & environmental sciences

For a reference copy of the document with all sections, see nature.com/documents/nr-reporting-summary-flat.pdf

Life sciences study design

All studies must disclose on these points even when the disclosure is negative.

Sample size	This manuscript was based on the image dataset from one fly brain and nerve cord. The sample size is standard for the field and was determined by the technical, computational, and manual proofreading constraints inherent to dense, electron microscopy-based connectomics. It is sufficient for analysis due to high stereotypy of the model system and large numbers of mapped neurons (n = ~160000) and hundreds of millions of synapses.
Data exclusions	No data was excluded
Replication	We did not replicate this study on other animals; however, we did successfully compare this animal's data to 3 other animals.
Randomization	Randomization is not applicable with one connectome.
Blinding	Blinding is not applicable with one connectome.

Reporting for specific materials, systems and methods

We require information from authors about some types of materials, experimental systems and methods used in many studies. Here, indicate whether each material, system or method listed is relevant to your study. If you are not sure if a list item applies to your research, read the appropriate section before selecting a response.

Materials & experimental systems

n/a	Involved in the study
☒	☐ Antibodies
☒	☐ Eukaryotic cell lines
☒	☐ Palaeontology and archaeology
☐	☒ Animals and other organisms
☒	☐ Clinical data
☒	☐ Dual use research of concern
☒	☐ Plants

Methods

n/a	Involved in the study
☒	☐ ChIP-seq
☒	☐ Flow cytometry
☒	☐ MRI-based neuroimaging

Animals and other research organisms

Policy information about studies involving animals; ARRIVE guidelines recommended for reporting animal research, and Sex and Gender in Research

Laboratory animals	5-6 day post-eclosion wild-type Drosophila melanogaster (F1 progeny of a w1118 × Canton-S cross)
Wild animals	No wild animals were used in this study
Reporting on sex	Female
Field-collected samples	No field-collected animals were used in this study
Ethics oversight	No ethics oversight required.

Note that full information on the approval of the study protocol must also be provided in the manuscript.

Plants

Seed stocks	*Report on the source of all seed stocks or other plant material used. If applicable, state the seed stock centre and catalogue number. If plant specimens were collected from the field, describe the collection location, date and sampling procedures.*
Novel plant genotypes	*Describe the methods by which all novel plant genotypes were produced. This includes those generated by transgenic approaches, gene editing, chemical/radiation-based mutagenesis and hybridization. For transgenic lines, describe the transformation method, the number of independent lines analyzed and the generation upon which experiments were performed. For gene-edited lines, describe the editor used, the endogenous sequence targeted for editing, the targeting guide RNA sequence (if applicable) and how the editor was applied.*
Authentication	*Describe any authentication procedures for each seed stock used or novel genotype generated. Describe any experiments used to assess the effect of a mutation and, where applicable, how potential secondary effects (e.g. second site T-DNA insertions, mosiacism, off-target gene editing) were examined.*
